# Efficacy of Corticosteroid Therapy for HTLV-1-Associated Myelopathy: A Randomized Controlled Trial (HAMLET-P)

**DOI:** 10.3390/v14010136

**Published:** 2022-01-12

**Authors:** Junji Yamauchi, Kenichiro Tanabe, Tomoo Sato, Masanori Nakagawa, Eiji Matsuura, Yoshio Tsuboi, Keiko Tamaki, Hirokuni Sakima, Satoshi Ishihara, Yuki Ohta, Naoki Matsumoto, Kenichi Kono, Naoko Yagishita, Natsumi Araya, Katsunori Takahashi, Yasuo Kunitomo, Misako Nagasaka, Ariella Coler-Reilly, Yasuhiro Hasegawa, Abelardo Araujo, Steven Jacobson, Maria Fernanda Rios Grassi, Bernardo Galvão-Castro, Martin Bland, Graham P. Taylor, Fabiola Martin, Yoshihisa Yamano

**Affiliations:** 1 Department of Rare Diseases Research, Institute of Medical Science, St. Marianna University School of Medicine, Kawasaki 216-8512, Japan; junji.yamauchi@marianna-u.ac.jp (J.Y.); tomoo@marianna-u.ac.jp (T.S.); yagi@marianna-u.ac.jp (N.Y.); araya@marianna-u.ac.jp (N.A.); takahashi@marianna-u.ac.jp (K.T.); y-kunitomo@marianna-u.ac.jp (Y.K.); acoler-reilly@wustl.edu (A.C.-R.); 2 Division of Neurology, Department of Internal Medicine, St. Marianna University School of Medicine, Kawasaki 216-8511, Japan; hasegawa-neuro1@marianna-u.ac.jp; 3 Department of Frontier Medicine, Institute of Medical Science, St. Marianna University School of Medicine, Kawasaki 216-8511, Japan; kenichiro.tanabe@marianna-u.ac.jp; 4 Department of Neurology, Kyoto Prefectural University of Medicine Graduate School of Medical Science, Kyoto 602-8566, Japan; mnakagaw@koto.kpu-m.ac.jp; 5 Department of Neurology and Geriatrics, Kagoshima University Graduate School of Medical and Dental Sciences, Kagoshima 890-8544, Japan; eiji.matsuura@gmail.com; 6 Department of Neurology, Fukuoka University, Fukuoka 814-0180, Japan; tsuboi@cis.fukuoka-u.ac.jp (Y.T.); cerisier.pommier.kei@gmail.com (K.T.); 7 Department of Cardiovascular Medicine, Nephrology and Neurology, Graduate School of Medicine, University of the Ryukyus, Okinawa 903-0215, Japan; sakima51@gmail.com (H.S.); satoshi430@gmail.com (S.I.); 8 Department of Pharmacology, St. Marianna University School of Medicine, Kawasaki 216-8511, Japan; yuki-o@marianna-u.ac.jp (Y.O.); matsumoto@marianna-u.ac.jp (N.M.); 9 Translational Research Center for Medical Innovation, Foundation for Biomedical Research and Innovation at Kobe, Kobe 650-0047, Japan; kono@tri-kobe.org; 10 Department of Advanced Medical Innovation, St. Marianna University Graduate School of Medicine, Kawasaki 216-8511, Japan; misakonjp@yahoo.co.jp; 11 Division of Hematology and Oncology, Department of Medicine, University of California Irvine School of Medicine, Irvine, CA 92617, USA; 12 Department of Internal Medicine, Division of Bone and Mineral Diseases, Washington University School of Medicine, St. Louis, MO 63110, USA; 13 Division of Neurology, Department of Internal Medicine, SHIN-YURIGAOKA General Hospital, Kanagawa 215-0026, Japan; 14 Laboratory for Clinical Research in Neuroinfections, Evandro Chagas National Institute of Infectious Diseases, Oswaldo Cruz Foundation (FIOCRUZ), Rio de Janeiro 21040-900, Brazil; abelardo@ufrj.br; 15 Viral immunology Section, Neuroimmunology Branch, National Institute of Neurological Disorders and Stroke, National Institutes of Health, Bethesda, MD 20892, USA; jacobsons@ninds.nih.gov; 16 Escola Bahiana de Medicina e Saúde Pública, Salvador 40290-000, BA, Brazil; fernanda.grassi@fiocruz.br (M.F.R.G.); bgalvao@bahiana.edu.br (B.G.-C.); 17 Gonçalo Moniz Institute, Oswaldo Cruz Foundation, Salvador 40296-710, BA, Brazil; 18 Department of Health Sciences, Seebohm Rowntree Building, University of York, York YO10 5DD, UK; martin.bland@york.ac.uk; 19 Section of Virology, Department of Infectious Disease, Imperial College London, London W2 1PG, UK; g.p.taylor@imperial.ac.uk; 20 School of Public Health, Faculty of Medicine, University of Queensland, 266 Herston Road, Herston, QLD 4006, Australia; fabiola.az.martin@gmail.com

**Keywords:** human T-lymphotropic virus type 1, HTLV-1-associated myelopathy, prednisolone, methylprednisolone, randomized controlled trial

## Abstract

Corticosteroids are most commonly used to treat HTLV-1-associated myelopathy (HAM); however, their clinical efficacy has not been tested in randomized clinical trials. This randomized controlled trial included 8 and 30 HAM patients with rapidly and slowly progressing walking disabilities, respectively. Rapid progressors were assigned (1:1) to receive or not receive a 3-day course of intravenous methylprednisolone in addition to oral prednisolone therapy. Meanwhile, slow progressors were assigned (1:1) to receive oral prednisolone or placebo. The primary outcomes were a composite of ≥1-grade improvement in the Osame Motor Disability Score or ≥30% improvement in the 10 m walking time (10 mWT) at week 2 for rapid progressors and changes from baseline in 10 mWT at week 24 for slow progressors. In the rapid progressor trial, all four patients with but only one of four without intravenous methylprednisolone achieved the primary outcome (*p* = 0.14). In the slow progressor trial, the median changes in 10 mWT were −13.8% (95% CI: −20.1–−7.1; *p* < 0.001) and −6.0% (95% CI: −12.8–1.3; *p* = 0.10) with prednisolone and placebo, respectively (*p* for between-group difference = 0.12). Whereas statistical significance was not reached for the primary endpoints, the overall data indicated the benefit of corticosteroid therapy. (Registration number: UMIN000023798, UMIN000024085)

## 1. Introduction

Human T-lymphotropic virus type 1 (HTLV-1) infects at least 5–10 million people globally. Moreover, it causes rare but devastating diseases, including HTLV-1-associated myelopathy (HAM) and adult T-cell leukemia/lymphoma (ATL) in a small proportion of infected individuals [1,2,3]. HAM is characterized by chronic spinal cord inflammation, particularly at the thoracic level, resulting in neurological disorders such as spastic paraparesis, sensory disturbance in the legs, and bladder and bowel dysfunction [4]. To date, there is no treatment for HAM. Interferon-α is the only drug that demonstrated clinical efficacy in a randomized controlled trial (RCT) [5]. However, this medication is seldom used because it is not highly efficient [6]. Although data are limited, corticosteroids are most commonly used to maintain motor function by suppressing inflammation [4,6,7].

Typically, neurological deterioration progresses slowly in HAM. However, the rate of progression or the disease activity varies among patients, ranging from minor walking abnormalities for more than a decade to the inability to walk within years [8,9,10,11]. The international HAM guidelines (2019) stated that patients should be classified according to the three types of progression (rapid, slow, and very slow) [12]. Low-dose steroid therapy with or without high-dose induction may be recommended based on the category. However, these recommendations are based on retrospective observational studies and clinical experiences [13,14,15]. Therefore, to our knowledge, this multicenter RCT is the first to assess the efficacy of intravenous methylprednisolone induction therapy for patients with rapidly progressing HAM (rapid progressors) and oral prednisolone treatment for those with slowly progressing HAM (slow progressors).

## 2. Materials and Methods

### 2.1. Standard Protocol Approvals, Registrations, and Patient Consents

This phase 2 RCT (HAMLET-P) was conducted from August 2016 to June 2020 at five sites in Japan in accordance with the International Conference on Harmonization Guidelines for Good Clinical Practice and the Declaration of Helsinki (clinical trial registration identifiers UMIN000023798 and UMIN000024085). The protocol was approved by the ethics committee/institutional review board of each institution (St. Marianna University Group Institutional Review Board, University Hospital, Kyoto Prefectural University of Medicine Institutional Review Board, Institutional Review Board of Fukuoka University Hospital, Institutional Review Board, Kagoshima University Hospital, and University of the Ryukyus Hospital Institutional Review Board; project code A2202 (DAIGAKU); date of approval 20 July 2016). Written informed consent was obtained from all participants before enrollment. The study protocol, statistical analysis plan, and CONSORT checklist are available in Files S1–S4.

### 2.2. Participants

Patients aged ≥18 years and diagnosed with definite HAM based on the Belem criteria were eligible for this study [16]. Upon enrollment, to assess motor ability, the participants had to walk for ≥10 m with or without walking aids. The exclusion criteria included those with complications such as neurological diseases other than HAM, comorbidities affecting motor function (e.g., osteoarthritis, rheumatoid arthritis), severe organ dysfunction, cancer, and contraindications to corticosteroids; those receiving corticosteroids or drugs targeting HAM (e.g., interferon-alpha, immunosuppressive agents, antiretroviral agents, and valproic acid) within 12 or 48 weeks prior to providing consent in rapid and slow progressors, respectively; and those treated with drugs strongly affecting CYP3A4 metabolism.

### 2.3. Study Design

This study comprised two trial arms: the prospective, randomized, open, blinded endpoint trial to assess the efficacy of intravenous methylprednisolone induction therapy for rapid progressors; and the prospective, randomized, double-blind, placebo-controlled trial to evaluate the efficacy of oral prednisolone therapy for slow progressors (Figure S1). Study visits were performed for progression rate assessment at −12, −8, −4, and 0 weeks (last assessment) (progression assessment period) as well as at day 1 (baseline) and 2, 4, 8, 12, 24, 28, 32, 36, and 48 weeks (treatment period). After eligibility screening, patients were registered for the progression rate assessment based on changes in motor function. Then, they were classified as rapid progressors at the time of registration if the clinical history within 12 weeks prior to registration met one of the following rapid progressor criteria: ≥30% worsening in the time taken to walk 10 m (10 m walking time [10 mWT]) or ≥1-grade deterioration in the Osame Motor Disability Score (OMDS), which is a specific scale for HAM, ranging from 0 to 13, with higher scores indicating greater disability [17]. For other patients, motor function was assessed during the 12-week progression assessment period and was classified as follows: rapid progressors, those who fulfilled the aforementioned rapid progressor criteria; slow progressors, those who experienced 10–<30% worsening in 10 mWT at the last assessment; and non-progressors, those who experienced <10% worsening. After the progression assessment period, non-progressors were followed-up without the trial drug for 48 weeks and were reclassified as rapid or slow progressors if their 10 mWT worsened by ≥30% or 10–<30% compared with the best time recorded during the progression assessment period. After the participants were classified into the progression groups, they were assigned to a specific treatment and followed up for 48 weeks.

### 2.4. Trial Intervention

Rapid progressors were randomly assigned (1:1) to receive a 3-day course of intravenous methylprednisolone at a dose of 1 g/day (Pfizer Japan Inc., Tokyo, Japan) along with oral prednisolone once per day (Nipro Pharma Corporation, Osaka, Japan) (pulse group), or oral prednisolone alone (non-pulse group; Figure S2). The non-pulse group did not receive placebo. In both groups, oral prednisolone was administered at a dose of 0.5 mg/kg body weight (BW) until week 2 and was then tapered to 20 mg by week 4 and to 5 mg by week 24. After alternate-day administration of 5 mg prednisolone for 14 days (at week 26), all patients discontinued prednisolone, except those who required a prespecified additional treatment for deterioration. Then, they were followed up until week 48. If patients experienced motor function deterioration after week 4, additional treatment (oral prednisolone with or without intravenous methylprednisolone) was administered according to the protocol (Figure S2).

Slow progressors were randomly assigned (1:1) to receive oral prednisolone or matching placebo once daily (Figure S3). In the prednisolone group, prednisolone was administered at a dose of 0.5 mg/kg BW until day 7, tapered to 5 mg/day by week 24, and then continued at 5 mg/day until week 48. In the placebo group, 5 mg/day prednisolone was initiated after the 24 week placebo period and was continued until week 48. If patients experienced ≥10% worsening in 10 mWT twice or ≥1-grade deterioration in OMDS after week 24, prednisolone was increased according to the protocol.

Medications for urinary symptoms and spasticity that had been started prior to enrollment were continued without changes throughout the trial.

### 2.5. Randomization and Blinding

We used web-based interactive response technology for randomization. Rapid progressors were stratified according to the use of walking aids (none or unilateral/bilateral) and randomized with permuted blocks of two patients. Slow progressors were randomized using the minimization method, adjusting for sex (male/female), use of walking aids (none or unilateral/bilateral), and trial site. Only physicians who performed clinical evaluations were blinded to the treatment allocation of rapid progressors. Therefore, patients and treating physicians knew their treatment. For slow progressors, all patients and study staff were blinded, and study drugs in numbered containers were supplied by an external vendor to ensure blinding.

### 2.6. Disease Evaluation

In clinical evaluations, we assessed mobility, dysuria, sensory dysfunction of the lower extremities, and the patient’s self-assessment of HAM-related symptoms. Mobility was evaluated using the OMDS, 10 mWT, 2 and 6 min walk tests (distance walked in 2 and 6 min, 2 MWD, and 6 MWD), and the timed up-and-go test (time required to stand up from a chair, walk 3 m away, turn, walk back, and sit down again). Spasticity of the knee extensor and flexor muscles was evaluated using the Modified Ashworth Scale (MAS; grades: 0, 1, 1+, 2, 3, and 4), with higher grades indicating more severe spasticity [18]. Dysuria was assessed using the International Prostate Symptom Score (IPSS), with higher scores (range: 0–35) indicating more difficulty in urinating [19]; the Overactive Bladder Symptom Score (OABSS), with higher scores (range: 0–15) indicating more severe urinary urgency [20]; the International Consultation on Incontinence Questionnaire-Short Form (ICIQ-SF), with higher scores (range: 0–21) indicating more severe incontinence [21]; and the Nocturia–Quality of Life Questionnaire (N-QOL), with lower scores (range: 0–100) indicating a lesser quality of life [22]. The patient’s subjective assessment of the global condition of HAM, mobility, and sensory dysfunction was performed using a 100 mm visual analog scale (VAS), in which higher values indicate more severe conditions. The Insituto de Pesquisa Clinica Evandro Chagas disability score (IPEC) 1 was used to comprehensively evaluate HAM-related symptoms [23].

Regarding laboratory analyses, we performed complete blood count, blood chemistry test, and cerebrospinal fluid (CSF) analysis. We also evaluated the CSF concentrations of neopterin (a marker of immune system activation) and CXCL10 (a chemokine mainly induced by interferon gamma), as well as HTLV-1 proviral loads in the peripheral blood mononuclear cells (PBMCs) and CSF cells, as described in the Supplementary Materials [24]. For safety evaluation, adverse events (AEs) were assessed according to the National Cancer Institute Common Terminology Criteria for Adverse Events, version 4.0. Serious AEs were defined as life-threatening conditions, congenital abnormalities or birth defects, diseases considered fatal by the investigator, and conditions resulting in hospitalization or prolonged hospital stay, persistent or clinically serious disability or incapacity, and death. Whether AEs were attributed to the treatment regimen was confirmed by the investigators.

### 2.7. Outcomes

In rapid progressors, the primary outcome was a composite of ≥1-grade improvement in OMDS or ≥30% improvement in 10 mWT at week 2 compared with baseline. The secondary outcomes included: each component of the primary outcome; changes in 10 mWT, 2 MWD, and 6 MWD, and CSF neopterin and CXCL10 concentrations; motor function deterioration requiring intravenous methylprednisolone therapy between week 4 and week 24; and safety.

In slow progressors, the primary outcome was a change in 10 mWT at week 24 from baseline. The secondary outcomes included: changes in 2 MWD, 6 MWD, and CSF markers; differences in changes in 10 mWT, 2 MWD, 6 MWD, and CSF markers between the placebo and active drug periods (from baseline to week 24 and from week 24 to week 48) in the placebo group; and safety.

### 2.8. Statistical Analysis

We analyzed the treatment efficacy using the intention-to-treat population (full analysis set, FAS), which comprised all eligible patients who received at least one dose of treatment. Moreover, the per-protocol set (PPS), in which patients with serious protocol violations were excluded from the FAS, was evaluated. All patients who received at least one dose of the trial drug underwent safety analysis.

The proportions of rapid progressors who met the primary outcome, with 95% confidence intervals (CIs), were analyzed. Next, between-group differences were evaluated using Fisher’s exact test. Because this study arm was underpowered for detecting even large differences due to its small sample size, we hypothesized that intravenous methylprednisolone therapy is effective if the proportion of patients who meet the primary endpoint in the pulse group is higher than that in the non-pulse group.

In slow progressors, the primary endpoint was analyzed using the mixed-effect model with the repeated-measures (MMRM) approach. The model included the fixed effects of treatment (prednisolone or placebo), 10 mWT at baseline, time points (week 4, 12, and 24), and treatment-by-time-point interactions. We calculated the least-squares (LS) means with 95% CIs at each time point and assessed the between-group differences at week 24. Of the key secondary outcomes, 2 MWD and 6 MWD were analyzed using MMRM. The CSF neopterin and CXCL10 concentrations at week 24 were analyzed by calculating the LS means with 95% CIs using analysis of covariance (ANCOVA). A natural logarithmic transformation was applied to produce a normal distribution in 10 mWT and CSF marker values. To interpret data, the results were expressed as median percent change with 95% CI, which were calculated from the exponential of the LS mean estimates. Further details are provided in the Supplementary Materials.

Statistical analysis was performed using SAS version 9.4 (SAS Institute Inc., Cary, NC, USA). *p*-values were two-sided, and a significance threshold of 0.05 was applied for all tests.

### 2.9. Sample Size Calculation

It was challenging to recruit rapid progressors because the annual incidence of rapidly progressing walking disability is low in Japan. Therefore, we set a minimum sample size of only four patients per group. One more patient was recruited if five of eight were assigned to the non-pulse group. Assuming that gait function will improve in ≥50% of patients receiving intravenous methylprednisolone and none among patients receiving oral prednisolone alone, the probability that more patients in the pulse group can achieve the primary endpoint compared with those in the non-pulse group was >80%.

Based on our clinical experience, we assumed that the 10 mWT of slow progressors could improve by at least 15% (0.165 in log) with treatment and could worsen by at least 6% without treatment (0.058 in log). Therefore, the sample size was set at 20 patients per group based on the estimation that this could provide 90% power for detecting a between-group difference of 21% (0.223 in log) using ANCOVA with a 5% significance threshold, assuming that the standard deviation was 0.21 (in log). Under the same assumption, 15 patients per group were estimated to provide a power of 80%.

## 3. Results

### 3.1. Study Population

**Figure 1:**
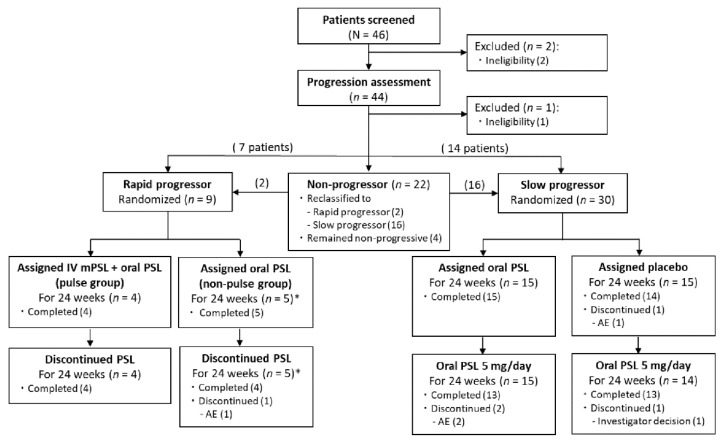
Trial flow chart. * One of five rapid progressors in the non-pulse group was excluded from the full and per-protocol sets due to ineligibility associated with treatment with carbamazepine (a strong CYP3A4 inducer) throughout the trial. AE, adverse event; IV mPSL, intravenous methylprednisolone; PSL, prednisolone.

From 31 August 2016 to 24 June 2019, 46 Japanese patients across 5 trial sites were enrolled, and 44 patients underwent the progression assessment (Figure 1). After the evaluation, patients were classified as rapid (*n* = 7), slow (*n* = 14), and non-progressors (*n* = 22). Several of the 22 non-progressors later experienced deterioration, and they were subsequently reclassified as rapid (*n* = 2) and slow progressors (*n* = 16) accordingly. Thus, 9 rapid and 30 slow progressors were randomized. Among them, 8 and 26 completed the trial, respectively. Due to the rarity of HAM, the target number of slow progressors (*n* = 40) was not achieved.

All randomized patients were included in the FAS, except for one rapid progressor who was taking the strong CYP3A4 inducer carbamazepine. All rapid progressors in the FAS were included in the PPS, whereas three slow progressors were excluded from the PPS due to lack of gait function assessment data at week 24 (*n* = 2) and low treatment adherence (≤80%, *n* = 1). The mean rates of adherence to the trial regimen (the proportion of administered doses per planned doses) were 99.8% and 99.2% in rapid and slow progressors, respectively.

Demographic characteristics were balanced in each progressor group between the treatment arms (Table 1 and Table 2). The median age of rapid and slow progressors was 62.0 and 64.0 years, respectively. All rapid (*n* = 8) and 23 (76.7%) of 30 slow progressors were women. The two progressor groups commonly presented with grade 5 OMDS (which requires unilateral support for walking) (5 rapid [62.5%] and 12 slow [40.0%] progressors). However, slow progressors in the prednisolone arm had more severe motor disability than those in the placebo arm (median OMDS grade, 5 vs. 4; median 10 mWT, 12.5 vs. 8.1 sec, respectively).

### 3.2. Efficacy Analysis of Rapid Progressors

**Figure 2:**
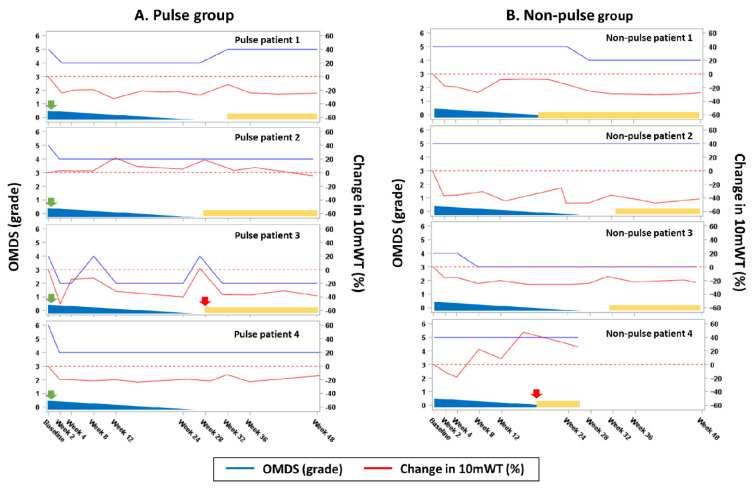
Primary endpoint in rapid progressors. (**A**,**B**) OMDS and percent changes in the 10 mWT from baseline are presented. The red dotted lines indicate the baseline 10 mWT (0% line). The green arrows (intravenous methylprednisolone) and blue triangles (oral prednisolone) represent steroid treatment. The red arrows (intravenous methylprednisolone) and yellow rectangles (oral prednisolone) represent additional treatment for worsening gait function. OMDS, Osame Motor Disability Score; 10 mWT, 10 m walking time.

At week 2, the OMDS improved by ≥1 grade in all 4 patients in the pulse group but none in the non-pulse group (*p* = 0.029; Figure 2). Meanwhile, the 10 mWT improved in 3 of 4 patients from each group after initiating steroid treatment, and 1 patient from each group experienced ≥30% improvement (*p* = 1.00). Thus, the primary outcome (improvement in OMDS or 10 mWT at week 2) was achieved by 4 patients with and 1 without intravenous methylprednisolone therapy (100% and 25%, respectively; difference: 75% [95% CI: −5.3–99.4; *p* = 0.14]; Table 3). At week 24, all patients in the pulse group maintained the OMDS improvement. In the non-pulse group, 1 patient experienced improvement in the OMDS at week 8; however, 2 patients met the deterioration criteria and required additional steroid therapy. At week 26, prednisolone treatment was discontinued in 4 and 2 patients in the pulse and non-pulse arms, respectively, according to the protocol. However, 3 and 2 patients resumed treatment due to deterioration.

**Figure 3:**
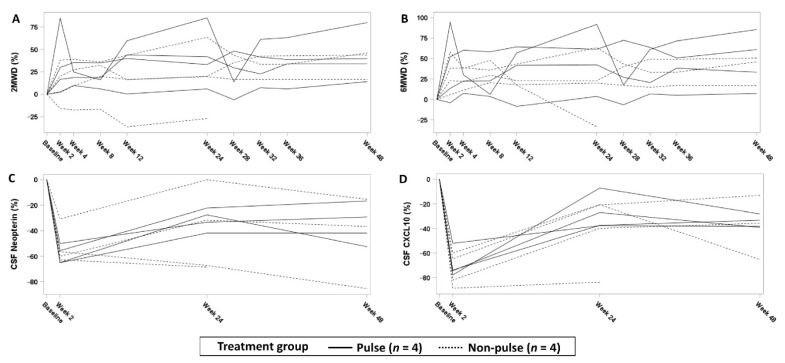
Changes in motor function and CSF marker concentrations from baseline in rapid progressors. (**A**–**D**) Percent changes in the 10 mWT, 2 MWD, 6 MWD, and CSF marker concentrations from baseline are shown. CSF, cerebrospinal fluid; 2 MWD, 2 min walk distance; 6 MWD, 6 min walk distance; 10 mWT, 10 m walking time.

A total of 3 of 4 patients in each group experienced an improvement in the 2 MWD and 6 MWD (Table 3, Figure 3 and Figure S4). Meanwhile, the CSF neopterin and CXCL10 concentrations decreased at week 2, and then they increased after the drug doses were tapered in both arms. Other disease evaluation parameters, including the timed up-and-go test, MAS, IPEC1, VAS, and urinary symptom scores, and HTLV-1 proviral loads, varied between patients (Figure S5).

### 3.3. Efficacy Analysis of Slow Progressors

**Figure 4:**
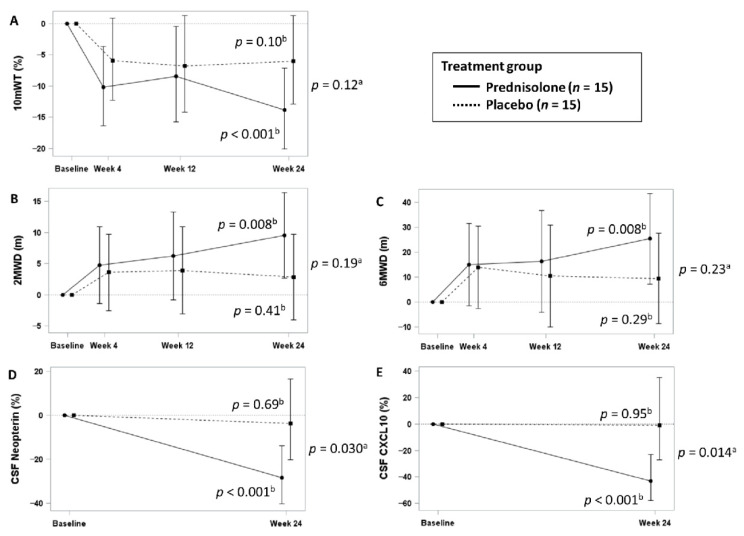
Least-squares mean estimates of changes in motor function and CSF marker concentrations from baseline in slow progressors. Least-squares mean changes from baseline and 95% CIs were calculated using MMRM (**A**–**C**) or ANCOVA (**D**,**E**). To interpret data, the 10 mWT (**A**), CSF neopterin concentration (**D**), and CXCL10 concentration (**E**) are expressed using median percent change and 95% CI, which were calculated from the exponential of the LS mean estimates of the log-transformed data. ^a^ *p*-value for between-group differences in changes at week 24. ^b^ *p*-value for comparison between baseline and week 24 values of each group CSF, cerebrospinal fluid; 2 MWD, 2 min walk distance; 6 MWD, 6 min walk distance; 10 mWT, 10 m walking time.

The median changes from baseline in 10 mWT at week 24 were −13.8% (95% CI: −20.1–−7.1; *p* < 0.001) and −6.0% (95% CI: −12.8–1.3; *p* = 0.10) in the prednisolone and placebo groups, respectively (*p* for between-group difference = 0.12; Figure 4 and Table 4). Although the 10 mWT at week 24 significantly improved from baseline only in the prednisolone group, the changes did not significantly differ between the prednisolone and placebo groups. The PPS analysis results were similar to those of the FAS.

Next, we analyzed changes in the 2 MWD, 6 MWD, and CSF marker concentrations, which are the secondary outcomes (Figure 4 and Table 4). The 2 MWD and 6 MWD at week 24 significantly improved from baseline with prednisolone. However, the changes did not significantly differ between the placebo and prednisolone groups. The LS mean changes in the 2 MWD and 6 MWD (meters) were 9.6 (95% CI: 2.7–16.4; *p* = 0.008) and 25.3 (95% CI: 7.3–43.4; *p* = 0.008) in the prednisolone group and 2.8 (95% CI: −4.0–9.7; *p* = 0.41) and 9.5 (95% CI: −8.6–27.5; *p* = 0.29) in the placebo group, respectively. Hence, the differences in the 2 MWD and 6 MWD were 6.7 (95% CI: −3.4–16.8; *p* = 0.19) and 15.9 (95% CI: −10.5–42.2; *p* = 0.25), respectively. Prednisolone significantly decreased CSF marker concentrations compared with placebo: the median changes in neopterin concentrations at week 24 from baseline were −28.3% (95% CI: −40.2–−13.8; *p* < 0.001) in the prednisolone group and −3.6% (95% CI: −20.3–16.5; *p* = 0.69) in the placebo group (*p* for between-group difference = 0.030); the changes in CXCL10 levels were −43.0% (95% CI: −57.7–−23.1; *p* < 0.001) and −0.9% (95% CI: −27.3–35.0; *p* = 0.95) in the prednisolone and placebo groups, respectively (*p* for between-group difference = 0.014). The placebo group received prednisolone at a dose of 5 mg/day after week 24. Gait function and CSF marker concentrations significantly improved at week 48 compared with week 24. (Table S1 and Figure S6). The change in CSF CXCL10 concentration significantly differed during the placebo and prednisolone periods (−0.9% vs. −54.1%; *p* = 0.006).

The results of other disease evaluations were analyzed via post hoc analysis (Tables S2 and S3 and Figure S7). Of 15 patients in the prednisolone group, 2 and 5 experienced ≥1-grade improvement in the OMDS from baseline at week 24 and week 48, respectively. No patients in the placebo group showed improvement in the OMDS while on placebo, but 7 of 13 patients had improvements after receiving prednisolone. The total IPEC1 score at week 24 significantly improved with prednisolone compared with placebo (median [IQR] changes from baseline: −2.0 [−5.0–−1.0] and 0.0 [0.0–0.0]; *p* = 0.002). There were no remarkable differences in other parameters.

### 3.4. Safety Analysis

None of the participants died during the trial. All rapid progressors developed AEs, and both treatment arms presented with steroid-related AEs (Table 5). The only serious AE, which resulted in trial discontinuation, was urinary tract infection in the non-pulse group.

During the first 24 weeks, 11 and 3 slow progressors in the prednisolone and placebo groups, respectively, developed AEs that were considered to be related to the treatment regimen; however, there were no serious AEs (Table 5). Between week 25 and week 48, during which both treatment arms received oral prednisolone, 8 and 5 patients in the prednisolone and placebo groups experienced steroid-related AEs, respectively. In addition, the prednisolone group presented with 2 serious AEs (ATL and herpes zoster). Meanwhile, the serious AE in the placebo group was facial palsy. During the 48 week treatment period, 2 patients in the prednisolone group developed herpes zoster. Moreover, urinary tract infection was observed in 4 and 2 patients in the prednisolone and placebo groups, respectively.

## 4. Discussion

We investigated the efficacy of corticosteroid therapy for HAM according to disease activity. The primary endpoint was achieved in rapid progressors. That is, the proportion of patients receiving intravenous methylprednisolone pulse therapy who achieved improvement in the OMDS or 10 mWT at week 2 was higher than that of those receiving non-pulsed steroid alone (Table 3 and Figure 2). All patients with pulse therapy but none of those without experienced improvement in the OMDS (*p* < 0.05). In addition, two patients in the non-pulse group required additional steroid therapy due to disease progression. Hence, oral prednisolone alone is insufficient among rapid progressors, and intravenous methylprednisolone is more effective for the rapid improvement and maintenance of motor function.

Observational studies reported that the motor function of most patients deteriorated in the long term despite corticosteroid treatment [6,25,26]. Thus, whether continuous corticosteroid therapy is beneficial for HAM has been debated. Therefore, treatment with prednisolone was discontinued at week 26 in rapid progressors. After discontinuation, five of six patients experienced deterioration and resumed prednisolone treatment, thereby indicating that the premature discontinuation of steroids could cause deterioration. Our findings support a recent retrospective study showing that continuous treatment was more effective in maintaining motor function than short-term therapy [27].

In slow progressors, we used 10 mWT as the primary outcome because it is a sensitive and objective functional measure with small intra-patient variability, and it can worsen by an average of 5.74% per 6 months in untreated patients with HAM [28]. Based on our clinical experience, there was an improvement of approximately 15% per 6 months with prednisolone (unpublished data), which is roughly equivalent to 2 years worth of gait deterioration. Because 1-grade OMDS deterioration can develop at an average of approximately 5 years, this improvement may be equivalent to 0.4 grade [6].

In this study, 10 mWT at week 24 significantly improved from baseline only in the prednisolone group, and the change was similar to our clinical experience. However, the results between the prednisolone and placebo groups did not significantly differ (Table 4 and Figure 4). Other mobility measures, including 2 MWD and 6 MWD, had similar tendencies. A statistically significant difference was not detected, presumably due to the small sample size. Nevertheless, prednisolone might be effective against HAM because the prednisolone group had better results in multiple mobility measures. Moreover, the results were consistent with significant findings in CSF markers, which are indicative of activation and inflammation [29].

This study provided important information for future clinical trials on HAM. The changes in 10 mWT, 2 MWD, and 6 MWD in the prednisolone group were consistent with the improvement in 10 mWT observed in earlier clinical observations. Therefore, 10 mWT is a simple and reliable outcome measure for HAM with a minimal burden on patients. Next, the 10 mWT of the placebo group improved, which was contrary to the results of clinical observations [28]. Thus, the placebo effect is nonnegligible in clinical trials on HAM. Since there was no data regarding the change in 10 mWT in clinical trial settings, we calculated the sample size of slow progressors using data obtained in clinical practice. If the sample size is calculated based on the current study, 100 participants (*n* = 50 in each group) are required to show the superiority over placebo of any therapy with similar efficacy to oral prednisolone. Recruiting such a large sample with rare diseases, including HAM, is not easy. Hence, it might be necessary to discuss whether applying general statistical thresholds to rare diseases is adequate. Moreover, developing reliable surrogate endpoints is essential to facilitate clinical trials.

CSF markers are advantageous because they can be measured objectively and do not exhibit any placebo effects. In the current research, we assessed CSF neopterin and CXCL10 concentrations. Their baseline concentrations were higher in rapid progressors than in slow progressors, well reflecting disease activity according to the classification criteria [30,31]. These markers significantly decreased in the prednisolone group, while there was minimal change in the placebo group (Figure 4). A retrospective study showed that changes in the concentrations of these markers are correlated with those in gait function in patients treated with corticosteroids [27]. Furthermore, in another retrospective study with a median follow-up of 4.1 years, patients whose CSF marker concentrations decreased during steroid therapy were at lower risk of deterioration [32]. Hence, this finding indicated a lower risk of deterioration in the future. Long-term maintenance of gait function is the gold standard endpoint for HAM. However, it is difficult to generate statistically significant outcomes if the sample size of clinical trials is limited. Nevertheless, CSF neopterin and CXCL10 can be a promising surrogate endpoint, with lower values indicating a better therapeutic efficacy.

In this study, the gait function and CSF marker concentrations of patients who received a placebo did not change. However, these parameters substantially improved after prednisolone therapy (Table S1 and Figure S6). These findings supported the aforementioned results regarding the treatment efficacy of prednisolone and the use of CSF markers.

Corticosteroid therapy was well tolerated, although steroid-related AEs were common. The overall incidence of serious AEs was similar among rapid and slow progressors between the treatment arms. Infectious disease is a common and, occasionally, serious complication of steroid therapy. Moreover, urinary tract infection is frequently observed in HAM due to neurogenic bladder issues. In this study, some patients presented with herpes zoster and urinary tract infections. Individuals infected with HTLV-1, including those with HAM, are at risk of developing ATL [33]. Those who have clonal expansion of HTLV-1-infected lymphocytes with ATL-related somatic mutations are at significantly high risk [34,35,36,37]. In this study, one slow progressor taking prednisolone developed ATL, and we retrospectively confirmed that this patient had such clones upon trial enrollment. Therefore, clonality examination should be included in eligibility screening in future trials. Whether immunosuppressive therapy can be recommended to high-risk patients with HAM and whether it affects ATL development should be investigated in future studies. When monitoring patients with HAM, special attention should be paid to infectious diseases and ATL.

The current study had several limitations. First and foremost, the sample size was extremely small. Thus, randomization might not have been significantly effective, and the baseline 10 mWT differed among slow progressors between the treatment groups. Rapid progressors did not receive placebo and were aware of their treatment, which might have affected the symptoms; moreover, some of them received additional treatment for deterioration after week 4. Meanwhile, all slow progressors received oral prednisolone after week 24. Therefore, long-term treatment efficacy was not completely validated. Lastly, all participants were Japanese, and most of them were women. Hence, the generalizability of the results might be limited.

In conclusion, to our knowledge, this is the first RCT on corticosteroid therapy for HAM. Although the primary endpoints did not significantly differ due to the small sample size, this study indicated that corticosteroid therapy was safe and beneficial for patients with HAM. Therefore, larger trials must be conducted to confirm treatment efficacy.

## Figures and Tables

**Table 1:** Characteristics of rapid progressors at baseline.

Characteristic		All Patients (*n* = 8)	Pulse (*n* = 4)	Non-Pulse (*n* = 4)
Age (years)		62.0 (55.5–65.5)	61.0 (55.5–65.5)	62.0 (56.0–69.5)
Sex	Female	8 (100.0%)	4 (100.0%)	4 (100.0%)
Race	Asian	8 (100.0%)	4 (100.0%)	4 (100.0%)
Disease duration of HAM (months)		7.0 (2.5–62.0)	7.0 (3.5–36.0)	32.0 (2.5–63.5)
Prior corticosteroid treatment (yes)		2 (25.0%)	1 (25.0%)	1 (25.0%)
OMDS	4 (needs handrail to climb stairs)	2 (25.0%)	1 (25.0%)	1 (25.0%)
	5 (needs unilateral support to walk)	5 (62.5%)	2 (50.0%)	3 (75.0%)
	6 (needs bilateral support to walk)	1 (12.5%)	1 (25.0%)	0 (0.0%)
10 mWT (seconds)		14.5 (10.5–30.4)	14.3 (10.5–18.5)	26.4 (10.0–44.0)
2 MWD (m)		66.9 (37.3–84.1)	66.9 (48.4–83.2)	56.6 (26.5–101.6)
6 MWD (m)		187.1 (67.9–241.1)	188.8 (125.1–241.2)	129.9 (31.1–288.8)
Timed up-and-go test (seconds)		12.7 (10.0–18.6)	12.7 (11.4–15.0)	14.9 (8.9–30.9)
MAS	1	3 (37.5%)	1 (25.0%)	2 (50.0%)
	1+	2 (25.0%)	0 (0.0%)	2 (50.0%)
	2	3 (37.5%)	3 (75.0%)	0 (0.0%)
IPEC1 score		13.0 (10.5–15.5)	13.0 (10.5–18.0)	13.5 (9.5–15.5)
CSF neopterin concentration (pmol/mL)		37.5 (21.0–49.0)	32.0 (21.0–41.5)	45.0 (24.0–57.5)
CSF CXCL10 concentration (pg/mL)		5099.2 (3608.8–5671.8)	4979.4 (3608.8–5344.9)	5426.1 (3297.2–6319.3)
HTLV-1 proviral load in PBMCs (copies/100 PBMCs)		6.6 (3.5–13.1)	5.0 (3.5–10.0)	9.78 (4.0–17.0)
HTLV-1 proviral load in CSF cells (copies/100 CSF cells)		9.7 (4.2–12.9)	7.1 (4.2–11.6)	10.7 (5.6–16.7)
HTLV-1 proviral load in CSF (copies/mL CSF)		538.2 (90.2–1002.7)	288.8 (90.2–1214.1)	754.4 (317.2–1002.7)
OABSS score		6.5 (3.5–7.5)	5.5 (2.0–7.5)	6.5 (4.5–8.0)
IPSS score		13.5 (5.0–24.0)	13.5 (5.0–24.0)	13.5 (9.0–23.5)
ICIQ-SF score		7.0 (3.5–12.0)	8.5 (1.5–15.0)	7.0 (5.0–9.0)
N-QOL score		77.1 (54.2–92.7)	92.7 (74.0–99.0)	62.5 (34.2–77.1)
VAS score for global condition of HAM (mm)		15.0 (2.5 –25.0)	10.5 (1.0–25.0)	15.0 (7.0–25.5)
VAS score for walking (mm)		11.0 (1.5–28.0)	6.5 (0.0–25.5)	17.0 (6.0–28.0)
VAS score for pain (mm)		80.0 (26.0–100.0)	98.5 (70.0–100.0)	66.5 (32.0–92.0)

Baseline characteristics are expressed as median (interquartile range) or number (percentage). CSF, cerebrospinal fluid; ICIQ-SF, International Consultation on Incontinence Questionnaire—Short Form; IPEC 1, Insituto de Pesquisa Clinica Evandro Chagas disability score 1; IPSS, International Prostate Symptom Score; MAS, Modified Ashworth Scale; N-QOL, Nocturia—Quality of Life Questionnaire; OABSS, Overactive Bladder Symptom Score; OMDS, Osame Motor Disability Score; PBMCs, peripheral blood mononuclear cells; VAS, visual analog scale; 2 MWD, 2 min walk distance; 6 MWD, 6 min walk distance; 10 mWT, 10 m walking time.

**Table 2:** Characteristics of slow progressors at baseline.

Characteristic		All Patients (*n* = 30)	Prednisolone (*n* = 15)	Placebo (*n* = 15)
Age		64.0 (58.0–68.0)	65.0 (63.0–67.0)	63.0 (55.0–69.0)
Sex	Female	23 (76.7%)	11 (73.3%)	12 (80.0%)
Race	Asian	30 (100.0%)	15 (100.0%)	15 (100.0%)
Disease duration of HAM (months)		31.0 (6.0–85.0)	24.0 (4.0–81.0)	37.0 (14.0–89.0)
Prior corticosteroid treatment (yes)		11 (36.7%)	6 (40.0%)	5 (33.3%)
OMDS	2 (abnormal gait, stumbling, stiffness)	2 (6.7%)	1 (6.7%)	1 (6.7%)
	3 (unable to run)	5 (16.7%)	0 (0.0%)	5 (33.3%)
	4 (needs handrail to climb stairs)	9 (30.0%)	4 (26.7%)	5 (33.3%)
	5 (needs unilateral support to walk)	12 (40.0%)	8 (53.3%)	4 (26.7%)
	6 (needs bilateral support to walk)	2 (6.7%)	2 (13.3%)	0 (0.0%)
10 mWT (seconds)		9.4 (8.0–13.7)	12.5 (8.8–19.4)	8.1 (7.3–10.2)
2 MWD (m)		98.8 (75.8–124.7)	81.0 (50.0–103.5)	120.0 (81.8–131.9)
6 MWD (m)		278.0 (201.2–370.0)	240.0 (130.0–298.5)	340.0 (234.6–381.7)
Timed up-and-go test (seconds)		9.6 (7.5–12.5)	10.6 (8.4–17.7)	8.1 (7.1–10.7)
MAS	0	2 (6.7%)	1 (6.7%)	1 (6.7%)
	1	15 (50.0%)	9 (60.0%)	6 (40.0%)
	1+	8 (26.7%)	5 (33.3%)	3 (20.0%)
	2	5 (16.7%)	0 (0.0%)	5 (33.3%)
IPEC1 (points)		13.0 (10.0–15.0)	15.0 (12.0–17.0)	13.0 (10.0–13.0)
CSF neopterin concentration (pmol/mL)		12.0 (7.0–19.0)	12.0 (6.0–24.0)	13.0 (7.0–18.0)
CSF CXCL10 concentration (pg/mL)		2275.8 (1269.3–3536.9)	1854.0 (1030.0–4539.5)	2306.4 (1350.2–3192.8)
HTLV-1 proviral load in PBMCs (copies/100 PBMCs)		4.9 (3.4–7.2)	5.3 (3.8–7.2)	4.7 (3.0–7.2)
HTLV-1 proviral load in CSF cells (copies/100 CSF cells)		8.9 (5.9–11.3)	8.9 (4.9–10.6)	8.9 (6.5–11.4)
HTLV-1 proviral load in CSF (copies/mL CSF)		312.2 (178.6–518.5)	297.0 (96.6–527.0)	323.0 (226.6–518.5)
OABSS score		5.5 (4.0–9.0)	5.0 (3.0–9.0)	6.0 (4.0–11.0)
IPSS score		15.0 (9.0–27.0)	13.0 (9.0–25.0)	16.0 (9.0–28.0)
ICIQ-SF score		6.0 (0.0–10.0)	6.0 (0.0–10.0)	6.0 (0.0–12.0)
N-QOL score		67.4 (58.3–81.3)	64.6 (45.8–83.3)	68.8 (65.9–81.3)
VAS score for global condition of HAM (mm)		31.5 (18.0–47.0)	23.0 (10.0–46.0)	32.0 (20.0–48.0)
VAS score for walking (mm)		38.0 (13.0–52.0)	27.0 (9.0–53.0)	39.0 (27.0–52.0)
VAS score for pain (mm)		51.0 (41.0–97.0)	45.0 (21.0–97.0)	64.0 (50.0–100.0)

Baseline characteristics are expressed as median (interquartile range) or number (percentage).CSF, cerebrospinal fluid; ICIQ-SF, International Consultation on Incontinence Questionnaire—Short Form; IPEC 1, Insituto de Pesquisa Clinica Evandro Chagas disability score 1; IPSS, International Prostate Symptom Score; MAS, Modified Ashworth Scale; N-QOL, Nocturia—Quality of Life Questionnaire; OABSS, Overactive Bladder Symptom Score; OMDS, Osame Motor Disability Score; PBMCs, peripheral blood mononuclear cells; VAS, visual analog scale; 2 MWD, 2 min walk distance; 6 MWD, 6 min walk distance; 10 mWT, 10 m walking time.

**Table 3:** Main outcomes in rapid progressors.

Measurement		Pulse Group (*n* = 4)	Non-Pulse Group (*n* = 4)	*p* Value
Primary endpoint				
Improvement in OMDS (≥1 grade) or 10 mWT (≥30%) at week 2		4 (100.0%; 95% CI: 39.8 to 100.0)	1 (25.0%; 95% CI: 0.6 to 80.6)	0.14
Secondary endpoints				
Improvement in OMDS (≥1 grade) at week 2		4 (100.0%; 95% CI: 39.8 to 100.0)	0 (0.0%; 95% CI: 0.0 to 60.2)	0.029
Improvement in 10 mWT (≥30%) at week 2		1 (25.0%; 95% CI: 0.6–80.6)	1 (25.0%; 95% CI: 0.6 to 80.6)	1.00
Changes in 10 mWT (%)	Week 2	−21.6 (−50.1to2.8)	−16.8 (−36.7 to −11.4)	0.56
	Week 4	−17.0 (−19.8 to 2.3)	−18.7 (−35.9 to −15.7)	0.56
	Week 12	−25.8 (−32.3 to 21.5)	−14.3 (−44.5 to 8.6)	1.00
	Week 24	−20.4 (−40.1 to 5.2)	−20.5 (−47.7 to 30.7)	1.00
Changes in 2 MWD (%)	Week 2	23.3 (2.7 to 84.7)	11.1 (−15.7 to 38.0)	0.39
	Week 4	21.8 (9.8 to 35.3)	18.5 (−17.4 to 38.7)	0.77
	Week 12	41.8 (0.4 to 59.5)	16.4 (−36.1 to 43.3)	0.25
	Week 24	37.5 (6.0 to 85.0)	20.0 (−27.0 to 63.3)	0.39
Changes in 6 MWD (%)	Week 2	32.8 (−4.1 to 94.2)	30.5 (5.7 to 58.1)	1.00
	Week 4	26.4 (7.4 to 60.3)	30.0 (11.4 to 38.7)	1.00
	Week 12	49.4 (−8.5 to 64.4)	20.6 (17.4 to 43.3)	0.39
	Week 24	51.8 (3.7 to 91.9)	21.4 (−33.5 to 63.3)	0.39
Changes in CSF neopterin concentrations (%)	Week 2	−60.3 (−65.1 to −50.0)	−58.2 (−62.9 to −30.8)	0.56
	Week 24	−30.4 (−41.9 to −22.2)	−49.5 (−68.6 to 0.0)	0.56
Changes in CSF CXCL10 concentrations (%)	Week 2	−74.2 (−77.9 to −52.0)	−73.4 (−88.7 to −59.9)	0.56
	Week 24	–32.2 (−37.8 to −7.0)	−30.4 (−83.7 to −20.6)	0.56
Patients who received intravenous methylprednisolone therapy between week 4 and 24		0 (0.0%; 95% CI: 0.0 to 60.2)	1 (25.0%; 95% CI: 0.6 to 80.6)	1.00
Patients in whom the 10 mWT worsened by ≥ 100% compared with week 4		0 (0.0%; 95% CI: 0.0 to 60.2)	0 (0.0%; 95% CI: 0.0 to 60.2)	1.00
Patients who could not stop treatment with prednisolone at week 26		0 (0.0%; 95% CI: 0.0 to 60.2)	2 (50.0%; 95% CI: 6.8 to 93.2)	0.43
Patients who resumed prednisolone treatment after week 26		3/4 (75.0%; 95% CI: 19.4 to 99.4)	2/2 (100.0%; 95% CI: 15.8 to 100.0)	1.00

Data are presented as number (percentage; 95% CI) or median (range). The Fisher’s exact test or the two-sample Wilcoxon test (exact method) was used to evaluate between-group differences. CSF, cerebrospinal fluid; OMDS, Osame Motor Disability Score; 2 MWD, 2 min walk distance; 6 MWD, 6 min walk distance; 10 mWT, 10 m walking time.

**Table 4:** Main outcomes in slow progressors.

Measurement		Prednisolone Group (*n* = 15)	Placebo Group (*n* = 15)	*p* Value ^a^
Primary endpoint				
Changes in 10 mWT (%)	Week 4	−10.2 (−16.3 to −3.6)	−6.0 (−12.3 to 0.8)	
	Week 12	−8.4 (−15.8 to −0.5)	−6.8 (−14.2 to 1.3)	
	Week 24	−13.8 (−20.1 to −7.1)	−6.0 (−12.8 to 1.3)	0.12
	*p* value ^b^	<0.001	0.10	
Secondary endpoints				
Changes in 2 MWD (m)	Week 4	4.8 (−1.4 to 11.0)	3.6 (−2.5 to 9.8)	
	Week 12	6.2 (−0.8 to 13.3)	3.9 (−3.1 to 10.9)	
	Week 24	9.6 (2.7 to 16.4)	2.8 (−4.0 to 9.7)	0.19
	*p* value ^b^	0.008	0.41	
Changes in 6 MWD (m)	Week 4	15.0 (−1.6 to 31.6)	14.0 (−2.5 to 30.5)	
	Week 12	16.3 (−4.1 to 36.7)	10.5 (−9.8 to 30.8)	
	Week 24	25.3 (7.3 to 43.4)	9.5 (−8.6 to 27.5)	0.23
	*p* value ^b^	0.008	0.29	
Changes in CSF neopterin concentrations (%)	Week 24	−28.3 (−40.2 to −13.8)	−3.6 (−20.3 to 16.5)	0.030
	*p* value ^b^	<0.001	0.69	
Changes in CSF CXCL10 concentrations (%)	Week 24	−43.0 (−57.7 to −23.1)	−0.9 (−27.3 to 35.0)	0.014
	*p* value ^b^	<0.001	0.95	

Least-squares (LS) mean changes from baseline and 95% CIs were calculated using MMRM (10 mWT, 2 MWD, and 6 MWD) or ANCOVA (CSF neopterin and CXCL10 concentrations). To interpret the data, the 10 mWT and CSF neopterin and CXCL10 concentrations are expressed as a median percent change, and 95% CI calculated from the exponential of the LS mean estimates because log-transformed values were used in the analysis as follows. LS mean changes in the 10 mWT (in natural logarithm) at week 24 from baseline were as follows: −0.149 (95% CI: −0.225 to −0.074; *p* < 0.001) in the prednisolone group and −0.087 (95% CI: −0.196 to 0.023; *p* = 0.10) in the placebo group. Difference: −0.087 (95% CI: −0.196 to 0.023; *p* = 0.12)). LS mean changes in neopterin concentrations (in natural logarithm) at week 24 were as follows: −0.332 (95% CI: −0.515 to −0.149; *p* < 0.001) in the prednisolone group and −0.087 (95% CI: −0.227 to 0.153; *p* = 0.69) in the placebo group. Difference: −0.295 (95% CI: −0.558 to −0.031; *p* = 0.030). LS mean changes in CXCL10 concentrations (in natural logarithm) at week 24 were as follows: −0.562 (95% CI: −0.861 to −0.263; *p* < 0.001) in the prednisolone group and −0.009 (95% CI: −0.319 to 0.300; *p* = 0.95) in the placebo group. Difference: −0.209 (95% CI: −0.983 to −0.122; *p* = 0.014). ^a^ *p*-value for comparison between the prednisolone and placebo groups at week 24. ^b^ *p*-value for comparison between baseline and week 24 in each group. CSF, cerebrospinal fluid; 2 MWD, 2 min walk distance; 6 MWD, 6 min walk distance; 10 mWT, 10 m walking time.

**Table 5:** Safety profile of rapid and slow progressors.

		Rapid Progressors		Slow Progressors	
Event		Pulse (*n* = 4)	Non-Pulse (*n* = 5)	Prednisolone (*n* = 15)	Placebo (*n* = 15)
Week 0 to 24	Any AEs ^a^	4 (25)	5 (25)	14 (38)	13 (19)
	AEs related to trial regimen ^b^	4 (18)	5 (13)	11 (23)	3 (3)
	Serious AEs	0	0	0	0
	Discontinuation due to AEs ^c^	0	0	0	1 (1)
Week 25 to 48	Any AEs ^d^	4 (9)	5 (22)	12 (36)	9 (23)
	AEs related to trial regimen ^e^	3 (4)	2 (3)	8 (13)	5 (6)
	Serious AEs ^f^	0	1 (1)	2 (2)	1 (1)
	Discontinuation due to AEs ^f^	0	1 (1)	1 (1)	1 (1)

Shown are the numbers of patients who experienced adverse events (AEs) with the numbers of events in parentheses. ^a^ In this study, ≥2 rapid progressors (pulse vs. non-pulse group) experienced AEs including oral mucositis (1 vs. 1), limb edema (1 vs. 1), pharyngitis (0 vs. 2), ligament sprain (1 vs. 1), hypercholesterolemia (2 vs. 2), weight gain (1 vs. 1), increased white blood cell count (1 vs. 3), hyperglycemia (2 vs. 0), insomnia (1 vs. 1), and urinary frequency (2 vs. 0). Moreover, ≥2 slow progressors (prednisolone vs. placebo group) experienced AEs such as Cushingoid (2 vs. 0), cystitis (1 vs. 1), pharyngitis (4 vs. 1), hypercholesterolemia (3 vs. 0), weight gain (2 vs. 0), arthralgia (1 vs. 1), back pain (0 vs. 2), headache (0 vs. 2), and insomnia (3 vs. 0). ^b^ In this study, ≥2 rapid progressors (pulse vs. non-pulse) had AEs correlated with the trial regimen, including limb edema (1 vs. 1), hypercholesterolemia (2 vs. 2), weight gain (1 vs. 1), high white blood cell count (1 vs. 3), hyperglycemia (2 vs. 0), insomnia (1 vs. 1), and urinary frequency (2 vs. 0). Moreover, ≥2 slow progressors (prednisolone vs. placebo) developed AEs correlated with the trial regimen, including Cushingoid (2 vs. 0), hypercholesterolemia (3 vs. 0), and insomnia (3 vs. 0). ^c^ In terms of AE resulting in the discontinuation, lumbar disc hernia (trial regimen-unrelated) was observed in the placebo group. ^d^ In this study, ≥2 rapid progressors (pulse vs. non-pulse) presented with dental caries (1 vs. 1), pharyngitis (0 vs. 2), hypercholesterolemia (2 vs. 0), arthralgia (1 vs. 1), and insomnia (1 vs. 1). Moreover, ≥2 slow progressors (prednisolone vs. placebo) presented with dental caries (2 vs. 0), pharyngitis (4 vs. 3), urinary tract infection (2 vs. 0), hypercholesterolemia (2 vs. 1), lymphocytopenia (2 vs. 0), weight gain (3 vs. 1), and headache (2 vs. 1). ^e^ In terms of AE correlated with the trial regimen, ≥2 rapid progressors (pulse vs. non-pulse) had hypercholesterolemia (2 vs. 0). In terms of AEs correlated with the trial regimen, ≥2 slow progressors (prednisolone vs. placebo) presented with hypercholesterolemia (2 vs. 1), lymphocytopenia (2 vs. 0), and weight gain (3 vs. 1). ^f^ The serious AEs were urinary tract infection (trial regimen-related) among rapid progressors in the non-pulse group, adult T-cell leukemia/lymphoma (trial regimen-unrelated) and herpes zoster (trial regimen-related) among slow progressors in the prednisolone group, and facial palsy (trial regimen-unrelated) among slow progressors in the placebo group. These AEs, except herpes zoster, resulted in the trial discontinuation.

## Data Availability

The data presented in this study are available in the manuscript, Supplementary Materials.

## Supplementary Materials

The following are available online at https://www.mdpi.com/article/10.3390/v14010136/s1, Supplementary Methods, Supplementary Tables (Table S1: Changes in motor function and CSF marker concentrations in the placebo group during the placebo and prednisolone periods, Table S2: Summary of exploratory assessment in slow progressors, Table S3: Changes in OMDS and the Modified Ashworth Scale score at 24 and 48 weeks from baseline in slow progressors), Supplementary Figures (Figure S1: Trial overview, Figure S2: Treatment flow chart of rapid progressors, Figure S3: Treatment flow chart of slow progressors, Figure S4: Changes in motor function and CSF marker concentrations in rapid progressors, Figure S5: Exploratory assessment in rapid progressors, Figure S6: Changes in motor function and CSF marker concentrations in slow progressors, Figure S7: Exploratory assessment in slow progressors), and Supplementary Files (File S1: Trial protocol, File S2: Statistical analysis plan for rapid progressors, File S3: Statistical analysis plan for rapid progressors, Figure S4: CONSORT Checklist).
