# Supplementary material for: Efficacy of Corticosteroid Therapy for HTLV-1-Associated Myelopathy: A Randomized Controlled Trial (HAMLET-P)
Source: Viruses. 2022 Jan 12;14(1):136. doi: 10.3390/v14010136 (PMC8780460; doi:10.3390/v14010136)
Supplement: Supplementary file 1 [file viruses-14-00136-s001.zip › Appendix A_TrialProtocol.pdf]

Phase IIb clinical trial of steroid therapy in patients with HAM/TSP

Protocol

Protocol Version 1.0 （2016/07/04）
Protocol Version 1.01 （2016/08/03）
Protocol Version 1.1 （2016/09/16）
Protocol Version 1.2 （2016/10/07）
Protocol Version 1.3 （2017/01/31）
Protocol Version 1.4 （2017/06/05）
Protocol Version 1.5 （2017/08/10）
Protocol Version 1.6 （2018/04/02）
Protocol Version 1.7 （2018/07/05）
Protocol Version 1.8 （2018/10/25）
Protocol Version 1.9 （2019/10/17）
Protocol Version 1.10 （2020/04/24）
Protocol Version 1.11 （2020/07/14）
Protocol Version 1.12 （2020/10/08）
Protocol Version 1.13 （2021/01/04）

Protocol Adherence

This study will be conducted in compliance with the study protocol. To prove this, I sign or affix my name and seal below and store a copy.

＜Principal Investigator＞

Institution ______________________________

Name ____________________________

Date ______________________________

CONFIDENTIAL

The information in this document will be provided only to the parties immediately concerned in the study, such as the coordinating investigator, the principal investigator and sub-investigators (including the study collaborators and other staff), the head of the medical institution, the study drug administrator, the HAMLET-P Coordinating Center, and the IRB.

【List of Abbreviations】

【Table of abbreviations】

Abbreviation	Unabbreviated term
ALT	Alanine Aminotranferase
AST	Aspartate Aminotransferase
ATLL	Adult T-cell Leukemia Lymphoma
BUN	Blood Urea Nitrogen
CTCAE v4.0	Common Terminology Criteria for Adverse Events v4.0
Ca	Calcium
Cl	Chlorine
CXCL10	C-X-C motif chemokine 10
eCB	eClinical Base
GCP	Good Clinical Practice
HAM-TSP	HTLV-1 Associated Myelopathy Tropical Spastic Paraparesis
HBs	Hepatitis B Surface
HBc	Hepatitis B Core
HBV-DNA	Heapatitis B Virus-Deoxyribonucleic Acid
HCV	Hepatitis C Virus
HIV	Human Immunodeficiency Virus
HTLV-1	Human T cell leukemia Virus type 1
IL	Interleukin
K	Potassium
LDH	Lactate Dehydrogenase
Na	Sodium
OMDS	Osame’s Motor Disability Score
PBMC	Peripheral Blood Mononuclear Cells
PCR	Polymerase Chain Reaction
SpO_2	Oxygen Saturation<br>Measured by Pulse Oximeter
YAM	Young Adult Mean

【Definition in this study】

<Final Assessment>

This refers to the assessment at the time of the last progressor assessment.

However,

・If a medical history assessment was performed in a rapid progressor, this refers to the final assessment before the second enrollment.

・For slow progressor and non-progressor, this refers to the assessment at the end of Week 12 since the progressor assessment period will last for 12 weeks.

<Baseline value>

As a general rule, this refers to the value in the final assessment.

If there is no indication of (rapid progressor) or (slow progressor) or (non progressor) in this protocol, the description shall be applicable in common to all types of progressor.

0. Overview

0.1 Study Plan

＜Overview of the study＞

The primary enrolment will be performed only for eligible patients with HAM. In principle, progressor assessment will be performed based on the 12-week observation. The secondary enrollment will be performed for subjects determined as rapid or slow progressor, and these subjects will be treated according to the protocol (Rapid: Clinical trial①, Slow: Clinical trial). Subjects who do not fulfil either progressor definitions will be followed up as non-progressor (Accompanying study).

Figure 1: Overview of the study

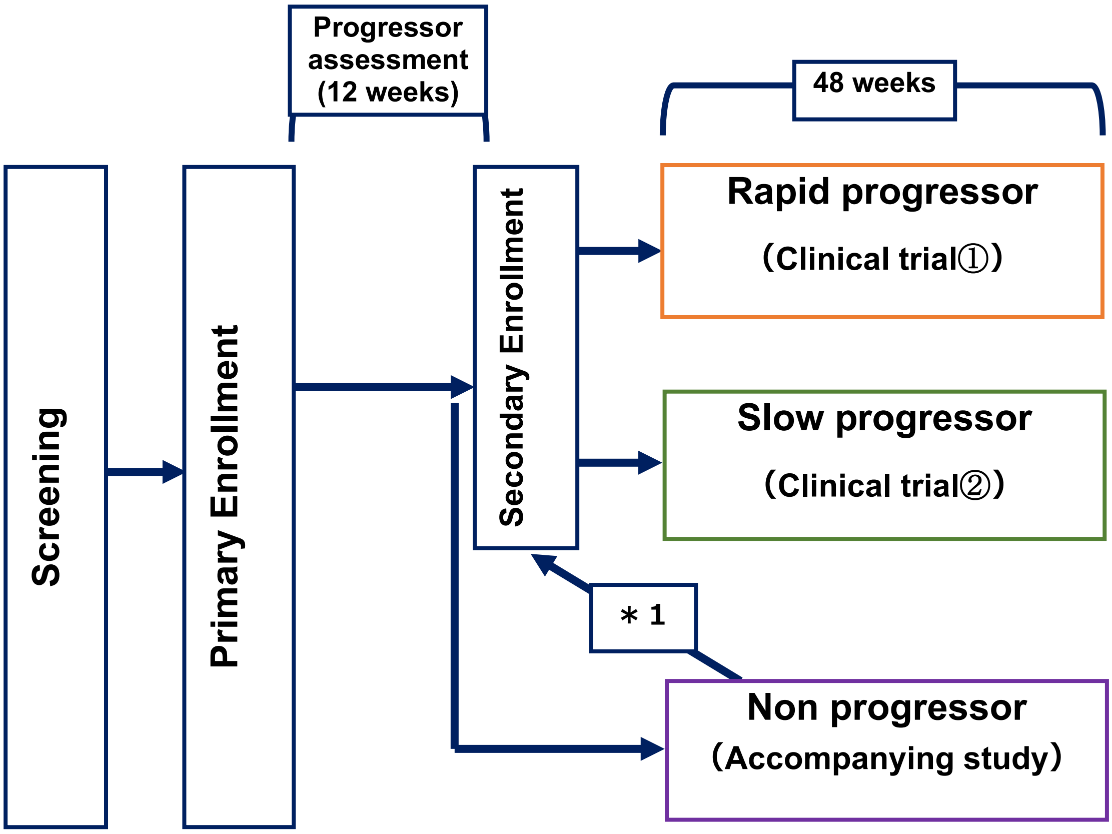

＊1: If a subject experiences worsening of symptoms during the observation period up to Week 48 and meets the definition of the rapid or slow progressor, the subject will finish the non-progressor study and start the secondary enrollment as a rapid or slow progressor, with the study treatment + observation according to the protocol for respective progressor.

Figure 2: Study flow chart

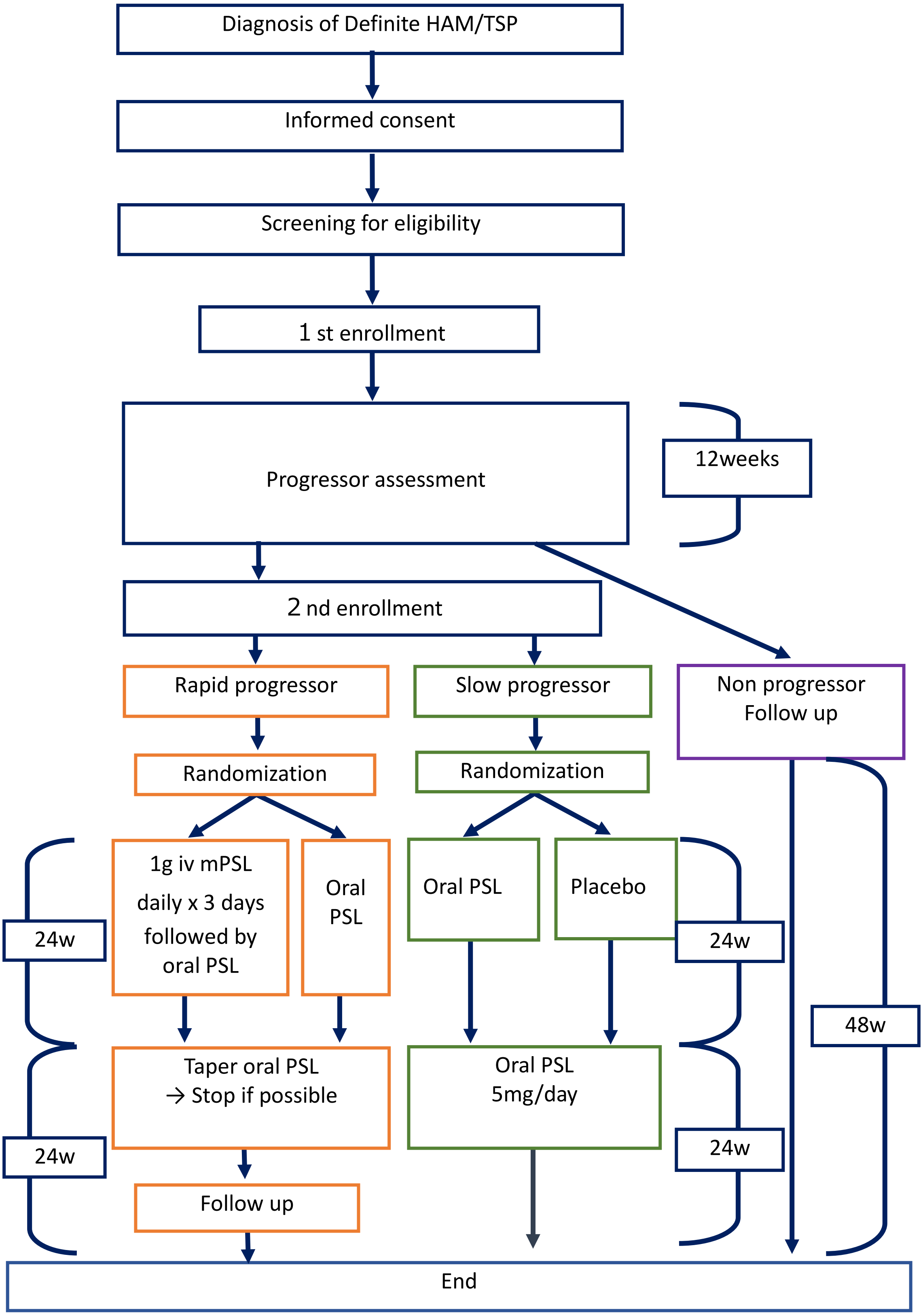

Rapid progressor（Clinical Trial①）

＜Explanation for Figure 2＞

Definite HAM/TSP

Patients with definite HAM/TSP who can walk 10 meter or more by themselves are potentially eligible for this study. The use of walking sticks but not wheelchairs or frames permitted.

Informed Consent

The principal investigator and subinvestigator provide the patient with information of the study and obtain written informed consent from the patients

Screening for Eligibility

Patients will be screened for eligibility by blood test, urinalysis, CSF test, and MRI.

MRI：MRI should be performed in the screening period. However, if it is difficult, data from previous tests may be used for eligibility screening.

If data obtained within 12 weeks prior to the date of informed consent at another hospital is available, a retest is not required.

Primary enrollment

Eligible subjects will be enrolled by web-based centralized enrollment.

Progressor assessment

During the period of baseline assessments (12 weeks), participants are recruited into one of three study sub-groups: rapid progressors, slow progressors or non-progressors depending on walking test and the evaluation of clinical history.

Patients with rapidly progressing disease are treated as soon as identified and do not have to complete the 12 weeks assessment period.

Slow and non progressors must complete the 12 weeks assessment period.

Secondary enrollment

The secondary enrollment and treatment assignment will be performed on the web for subjects determined as rapid or slow progressor. The secondary enrollment will not be performed for non progressor.

Treatmernt/Follow up

・ Rapid progressor will be treated with the study drug until Week 24, and followed up until Week 48.

Rapid progressor（Clinical Trial①）

- Slow progressor will be treated with the study drug until Week 24, and with prednisolone until Week 48.
- Non progressor will be followed up without treatment until Week 48.

For, details, refer to '10. Treatment Plan (After Secondary Enrollment).

0.2 Eligibility Criteria

0.2.1 Indication for Study

HTLV-1-associated myelopathy/tropical spastic paraparesis (HAM/TSP)

Diagnosis of HAM/TSP to Belem Criteria（2006）_Definite HAM/TSP

1. A non-remitting progressive spastic paraparesis with sufficiently impaired gait to be perceived by the patient. Sensory symptoms or signs may or may not be present. When present, they remain subtle and without a clear-cut sensory level. Urinary and anal sphincter signs or symptoms may or may not be present.
2. Presence of HTLV-I antibodies in serum and CSF confirmed by <u>Western blot*[1] or Line blot</u> and/or a positive PCR for HTLV-1 in blood and/or CSF
3. Exclusion of other disorders* that can resemble HAM/TSP

   *: multiple sclerosis; carcinomatous meningitis; familial spastic paraparesis; transverse myelitis; primary lateral sclerosis; syringomyelia; Lyme disease; B 12 and folate deficiency; Behçet disease; neurosyphilis; neurotuberculosis; sarcoidosis; HIV vacuolar myelopathy; collagen vascular diseases; autoimmune myelopathies; Sjögren's syndrome; toxic myelopathies; amyotrophic lateral sclerosis; fungal myelopathy; spinal arteriovenous fistula; hepatic myelopathy; parasitic myelopathy (visceral larva migrans of *Toxocara canis* and *Ascaris suum*); spinal cord compression (spinal tumor, cervical spondylosis, brain parasagittal tumor etc.); endemic regional myelopathies with similar clinical manifestations (including schistosomiasis and neurocysticercosis

0.2.2 Inclusion criteria

(1) Patients diagnosed with HAM/TSP according to the diagnostic guidelines described in Section 0.2.1.

(2) Patients who are 18 years of age or older at the time when informed consent is obtained

(3) Patients who are capable of walking at least 10 meters (regardless of whether a walking aid is used or not) at the time when informed consent is obtained

(4) Patients whose primary organ functions are stable

(According to the latest laboratory results from within a maximum of 28 days prior to the

Rapid progressor(Clinical Trial①)

date of enrolment)

① Neutrophil count: ≥1,500/mm^3

② Platelets: ≥100,000/mm^3

③ Hemoglobin: ≥9.0 g/dL

④ AST: ≤3-times the upper limit of normal (ULN)

⑤ ALT: ≤3-times the ULN

⑥ Serum creatinine: ≤1.5-times the UL

⑦ HbA1c(NGSP): ≤6.5%

(5) Patients who have given written consent to participate in the study of their own free will

(6) Patient must be willing and able to comply with all the aspects of trial design and follow-up

0.2.3 Exclusion criteria

(1) Patients who have received corticosteroids or other immune-modulating or anti-viral agent that alter the immune response to HTLV-1 within 12 weeks of entering the study if rapidly progressing

(2) Patients who have received corticosteroids or other immune-modulating or anti-viral agent that alter the immune response to HTLV-1 within 48 weeks if slowly progressing or not progressing

(3) Patients who have undergone highly invasive surgery under general anesthesia within 24 weeks prior to giving informed consent

(4) Patients who have received other study drugs within 16 weeks prior to giving informed consent

(5) Patients who have undergone live or attenuated/inactivated vaccinations within 4 weeks prior to the date of enrollment, or who plan on being vaccinated during the study period

(6) Patients who have taken ascorbic acid (≥1.5 g/day), prosultiamine, or pentosan polysulfate within 2 weeks prior to the date of enrollment

(7) Patients with a history of myocardial infarction

(8) Patients with a history of tuberculosis or with active tuberculosis

(9) Patients with serious complications (heart failure, lung disease, renal failure, hepatic failure, uncontrolled diabetes mellitus, etc.)

(10) Patients with uncontrolled high blood pressure

(11) Patients with uncontrolled electrolyte disorder

(12) Patients with thrombotic disease

(13) Patients who have a history of cancer, or those complicated with cancer

However, patients with radically resected solid tumor which has not recurred for at least 3 years before enrollment will be able to enroll in the study. Patients with radically

resected basal cell carcinoma of the skin, squamous cell carcinoma (except malignant melanoma), non-invasive cervix carcinoma, and carcinoma in situ in the gastrointestinal tract or corpus of the uterus will be able to enroll in the study if they are determined to be completely cured even if within 3 years of enrollment

(14) Patients with peptic ulcer
(15) Patients complicated with adult T-cell leukemia/lymphoma (ATL)
(16) Patients with uncontrolled eye disease
(17) Patients with a history of steroid-induced glaucoma
(18) Patients who are pregnant, breastfeeding, who may be pregnant, or wish to bear children
(19) Patients with complications of spinal cord compressive lesions such as spondylitis, ossification of the posterior longitudinal ligament, and ossification of the yellow ligament, or articular diseases such as rheumatoid arthritis and osteoarthritis
(20) Patients with neurological disorder or MRI findings attributable to other disease
(21) Patiens with ahistory of spine compression fracture
However, patients with traumatic fracture or those with ≥70% of lumber spine bone mineral density (YAM) will be able to enroll in the study.
(22) Patients with psychiatric disorder, epilepsy, or dementia
However, patients with epilepsy who have experienced no seizures for 3 years before enrollment will be able to enroll in the study.
(23) Patients who test positive for HBs antigen or HBV-DNA (using real-time PCR
(24) Patients who test positive for HIV antibody
(25) Patiens with a history of strongyloidis stercoralis infection
(26) Patients with systemic bacterial or fungal infection
(27) Patients who need medications that strongly induce or inhibit CYP3A4.
(28) Patients with a history of allergic reaction to the study drug
(29) Patients considered unqualified to participate in the study by the principal investigator or subinvestigator

0.3 Definition of the progressor caterogy

Subjects will be classified into three clinical subgroups (rapid-progressor, slow-progressor, or non-progressor) based on clinical history and the change in the 10 meter timed walk (10mWT) during the 12 week run-in. Clinical history will be assessed using OMDS. Grade 1 and Grade 2 of the original OMDS will be treated as Grade 2 in the study. The average of two measuments of 10mWT (rounded to two decimal place) will be used for judgement.

- Rapid progressor：

Rapid progressor（Clinical Trial①）

Subjects who fulfil the criteria based on clinical history will be categorized as rapid progressors without 12-week assessment. Where patients cannot be categorized as rapid progressors based on clinical history, they are followed up during the baseline assessment. The baseline assessment can be shortened if rapid clinical deterioration is confirmed.

[Assessment based on clinical history]

Patients display at least one of the following:

- Deterioration in the 10mTW ≥ 30% within the previous 12 weeks
- Deterioration in OMDS ≥ 1 Grade within the previous 12 weeks

[12-week Progressor assessment]

Patients display at least one of the following:

- Deterioration in the 10mTW of ≥ 30% during the 12 weeks of assessment
- Deterioration in OMDS of ≥ 1 Grade during the 12 weeks of assessment
- Non progressors who display, during the 48 weeks of follow up, deterioration in the 10mTW of ≥ 30% compared with the best record in the baseline assessment period.

➢ Slow progressor:

Patients display at least one of the following:

- Deterioration in the 10mTW of ≥ 10% but < 30% at the end of the 12 weeks of assessment
- Non progressors who display, during the 48 weeks of follow up, deterioration in the 10mTW of ≥ 10% but < 30% compared with the best record in the baseline assessment period.

➢ Non progressor:

These patients do not display, at the end of the 12 weeks of observations, any deterioration in the 10mTW that is more than or equal to 10%.

Rapid progressor（Clinical Trial①）

＜*1 Osame Motor Disability Scale＞

Grade	Motor disability
0	Normal gait and running
1	Normal gait but runs slowly } ＊1
2	Abnormal gait (staggering or spastic) } ＊1
3	Abnormal gait and unable to run
4	Needs support while using stairs but walks without assistance
5	Needs one hand support in walking
6	Needs two hands support in walking (can walk more than 10 meter)
7	Needs two hands support in walking (can walk less than 10 meter)
8	Needs two hands support in walking (can walk less than 5 meter)
9	Unable to walk but can walk on all fours
10	Unable to walk on all fours but can crawl with hands
11	Unable to crawl with hands but can turn sideways in bed
12	Unable to turn sideways but can move the toes
13	Completely bedridden (unable to move the toes)

[Notes for the OMDS assessment in this study]

*1: To be rated as "Grade2"

The outline of Rapid progressor (clinical trial①), Slow progressor (clinical trial②), and Non progressor (accompanying study) is described below.

Rapid progressor（Clinical Trial①）

Rapid progressor

0.4 Objectves (Rapid)

- Primary objective
  To test the efficacy of i.v. methylprednisolone followed by oral prednisolone therapy in patients with rapidly progressive HAM/TSP compared to oral prednisolone alone.
- Secondary objectives
  To test the safety of i.v. methylprednisolone and oral prednisolone therapy in patients with rapidly progressive HAM/TSP.
  To test the efficacy of oral prednisolone therapy in patients with rapidly progressive HAM/TSP.

0.5 Study design and evaluation（Rapid）

- Study design ： Prospective, Randomized, Open, Blinded-Endopoint (PROBE)
- Primary outcome measures
  - ◆ Efficacy
    Presence or absence of "≥30% improvement in the 10-meter timed walk" or "≥1 improvement in the OMDS" at Day 15 compared to the baseline value
- Secondary outcome measures
  - ◆ Efficacy
    - ・ 10mWT
      - ✧ Presence or absence of "≥30% improvement in the 10-meter timed walk" at Day 15 compared to the baseline value
      - ✧ Area under the curve (Baseline, Day15, Day29 (Week4))
      - ✧ Change at Day15 / Day29 (Week4) compared to baseline
    - ・ 2-minute walk distance and 6-minute walk distance
      - ✧ Area under the curve (Baseline, Day15, Day29 (Week4))
      - ✧ Change at Day15 / Day29 (Week4) compared to baseline
    - ・ OMDS
      Presence or absence of "≥1 improvement in the OMDS" at Day 15 compared to the baseline value
    - ・ CSF neopterin concentration
      Change at Day15 compared to baseline
    - ・ Proportion of subjects who received i.v. methylprednisolone between Day29（Week 4）and Day169（Week 24）
  - ◆ Safety

Rapid progressor（Clinical Trial①）

Adverse events（frequency, severity）

- Exprolatory outcomes
  - Efficacy
    - 10mWT
      Change at Day85 (Week12) / Day169 (Week24) compared to baseline
    - 2-minute walk distance and 6-minute walk distance
      Change at Day85 (Week12) / Day169 (Week24) compared to baseline
    - CSF neopterin concentration
      Change at Day169 (Week24) compared to baselin
    - Proportion of subjects who discontinue the study due to clinical exacerbation of HAM (an increase of 100% or more in 10mWT compared to baseline) between Day30 and Day169 (Week 24).
    - Proportion of subjects who could not discontinue the drug at Day183 (Week 26).
    - Proportion of subjects who resumed steroid treatment by Day337 (Week 48) among those who had discontinued the drug at Day183 (Week 26).

0.6 Target Sample Size and Study Period（Rapid）

- Target sample size: 8 subjects (4 subjects per arm)
  The planned number of subjects were determined as the maximum number possible to enroll from the perspective of feasibility.
  If a total of 8 subjects are enrolled and then 3 subjects are assigned to the pulse group and 5 subjects are assigned to the p.o. group, another subject will be enrolled for assessment. Apart from this, subjects shall be enrolled as many as possible during the enrollment period.
- Study period: August 2016 to August 2021
  （The secondary enrollment is possible until the end of June 2020.）
- However, the study period may be changed depending on the status of study progress.

0.7 Study Drug（Rapid）

- **Methylprednisolone** (Solu-Medrol 1000mg)
- **Prednisolone** (Prednisolone tablet 5mg)

Rapid progressor（Clinical Trial①）

0.8 Treatment Plan（After Secondary Enrollment）（Rapid）

Figure 3 : Treatment flow chart (Rapid progressor)

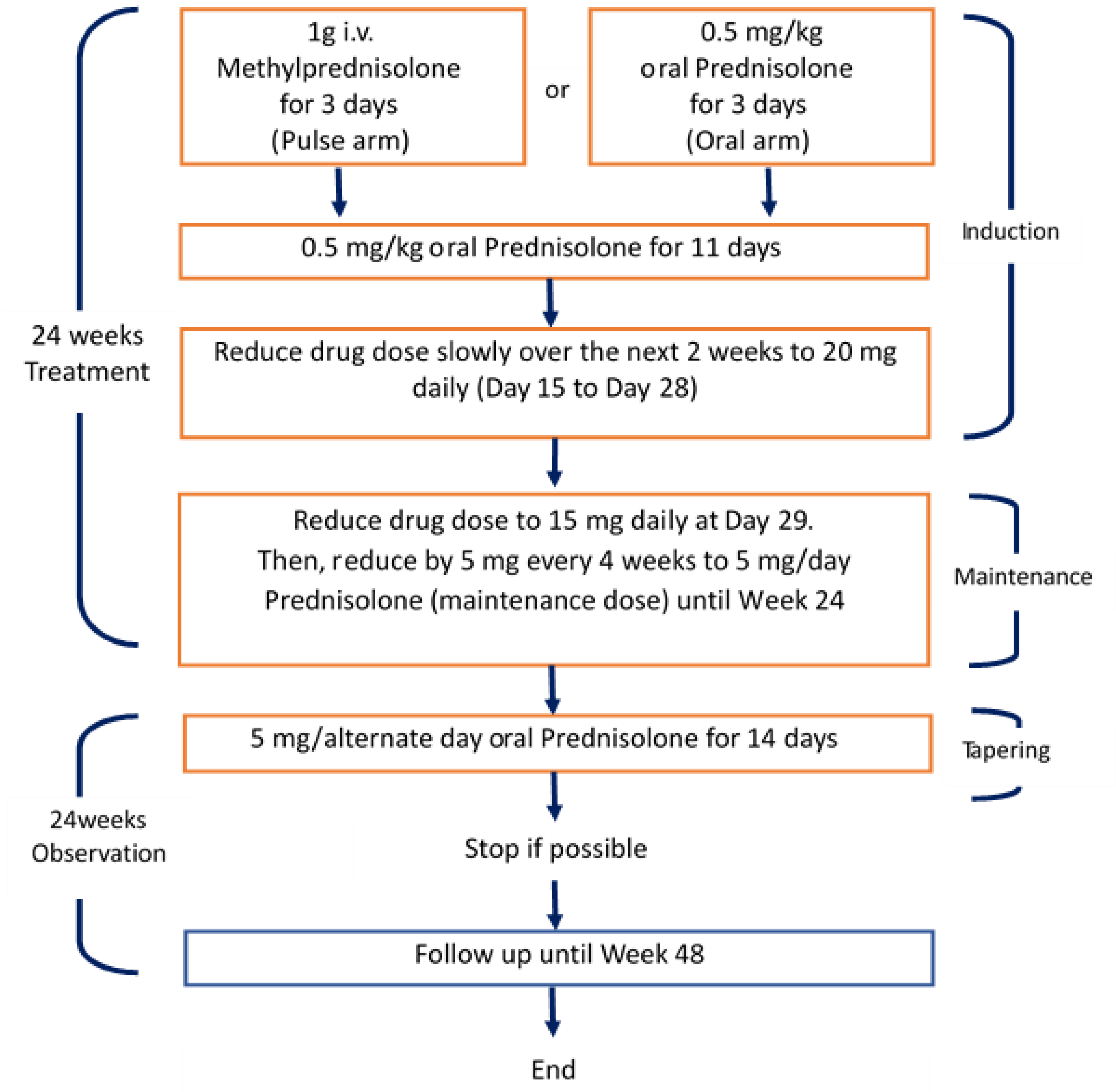

If a subject experienced worsening of symptoms of HAM due to a dose reduction or discontinuation during the study period and meets the criteria for dose increase or resumption, additional treatment shall be performed according to the procedure.

Rapid progressor（Clinical Trial①）

Prior to starting the study drug treatment, it shall be confirmed that the test values on the last assessment date meet the following criteria.

① Neutrophil count: ≥1,500/mm3
② Platelets: ≥100,000/mm3
③ Hemoglobin: ≥9.0 g/dL
④ AST: ≤3-times the upper limit of normal (ULN)
⑤ ALT: ≤3-times the ULN
⑥ Serum creatinine: ≤1.5-times the UL
⑦ HbA1c（NGSP）: ≤6.5%

＜Explanation for Figure 3＞

Testing and treatment can be done in the outpatient or inpatient setting.

Induction

Visits：Day1～Day3 (Pulse arm only), Day15±3 days, Week 4 (Day29±7 days)

- Patients will be treated with the assigned trial drug
  The pulse arm: administer a 3-day course of i.v. methylprednisolone
  The Oral arm: administer 0.5 mg/kg oral Prednisolone for 3 days [Day1～Day3]
- Administer 0.5 mg/kg oral Prednisolone for 11 days [Day4～Day14]
- Reduce drug dose slowly over the next 2 weeks to 20mg daily [Day15～Day28]

Maintenance

Visits：Week8 (Day57±7 days), Week12 (Day85±7 days), Week24 (Day169±7 days)

- Reduce drug dose to 15 mg daily at Day 29. Then, reduce by 5 mg every 4 weeks to 5 mg/day prednisolone (maintenance dose) until Week 24 [Day29～Day169]

Dose reduction/drug discontinuation

- Subjects shall take prednisolone 5 mg/day every other day for 14 days after Day 169 (Week 24) and then discontinue the drug.
- However, subjects who have increased the maintenance dose of oral predonine by Day 169 (Week 24) shall not taper the dose or discontinue the drug.

Follow up

Visits：Week28 (Day197±7days), Week32 (Day225±7days), Week36 (Day253±7days), Week48 (Day337±7days)

- Follow up patients until Week48.

0.8.1. Dose modification (dose increase/resumption)

Rapid progressor(Clinical Trial①)

If a subject experienced worsening of symptoms of HAM due to a reduction or discontinuation and meets the criteria for dose increase or resumption, additional treatment shall be performed according to the procedure within 7 days from the date when it is confirmed that the subject meets the criteria. Dose increase or resumption are permitted based on the values of the 10-meter timed walk and the OMDS obtained on an unscheduled study visit.

If a subject meets the dose increase/resumption criteria, the HAMLET-P Coordinating Center should be contacted before the dose increase/resumption. However, if no additional treatment is given, the clear reason should be documented.

After the dose increase, no transition to the next dose level shall be made until the end of the evaluation, regardless of the acceptable range.

Dose Increase and Resumption Criteria and Procedures

- After completion of evaluation on Day 29 to evaluation on Day 169 (Week 24)

  ① [Dose increase criteria]

  "≥30% worsening in the 10-meter timed walk" or "≥1 grade of worsening in the OMDS" compared to baseline

  [Dose increase procedures]

  Methylprednisolone 1 g/day is intravenously administered for 3 consecutive days. Subsequently, oral prednisolone is administered at 0.5 mg/kg/day (for 11 days) and gradually reduced to 20 mg/day over 2 weeks (the method of tapering should be determined at the discretion of physician). Subsequently, the dose is reduced by the range of 2.5 mg/day (5 mg every other day) /4 weeks to 5 mg/day/4 weeks to find a maintenance dose that does not meet the dose increase criteria, and the oral administration is continued at that maintenance dose.

  ② [Dose increase criteria]

  "≥10% worsening in the 10-meter timed walk compared to the value on Day 29 was observed on 2 visits". (Those 2 visits can be apart from each other.) If the walk test cannot be performed on Day 29 (Week 4) and the value is missing, the most recent result prior to Day 29 (Week 4) shall be used as the value on Day 29 (Week 4).

  [Dose increase procedures]

  Prednisolone is increased by 2.5 mg/day (5 mg every other day) /4 weeks to maintain at the dose that does not meet the increase criteria.

- After completion of evaluation on Day 169 (Week 24)

  ① [Dose increase/resumption criteria]

  "≥30% worsening in the 10-meter timed walk" or "≥1 grade of worsening in

the OMDS" compared to the value on Day 169 (Week 24) was observed. If the walk test cannot be performed on Day 169 (Week 24) and the value is missing, the most recent result prior to Day 169 (Week 24) shall be used as the value on 169 (Week 24).

[Dose increase/resumption procedures]

Methylprednisolone 1 g/day is intravenously administered for 3 consecutive days. Prednisolone is administered at the dose that was administered immediately before the worsening. If the dose increase criteria are subsequently met, the dose is increased by 2.5 mg/day (5 mg every other day) /4 weeks to find a maintenance dose that does not meet the dose increase criteria, and the oral administration is continued at that maintenance dose. When reducing the dose, the minimum maintenance dose shall be 5 mg.

② [Dose increase/resumption criteria]

"≥10% worsening in the 10-meter timed walk compared to the value on Day 169 (Week 24) was observed. If the walk test cannot be performed on Day 169 (Week 24) and the value is missing, the most recent result prior to Day 169 (Week 24) shall be used as the value on 169 (Week 24).

[Dose increase/resumption procedures]

Prednisolone is administered at the dose that was administered immediately before the worsening. If the dose increase criteria are subsequently met, the dose is increased by 2.5 mg/day (5 mg every other day) /4 weeks to find a maintenance dose that does not meet the dose increase criteria, and the oral administration is continued at that maintenance dose. When reducing the dose, the minimum maintenance dose shall be 5 mg.

0.8.2. Prednisolone Administration Rules

①Dose

- Do not split or crush the study drug.
- The dose should be set in 2.5 mg increments
- For the dose of 0.5 mg/kg/day, round off the fraction.

②Timing of oral administration

- Take the drug once a day.
- Oral administration after breakfast is recommended.

0.9 Criteria for Study Discontinuation

Rapid progressor（Clinical Trial①）

If a patient meets any of the following restrictions, said patient will be discontinued from the study:

(1) If a subject develops steroid-induced glaucoma.

(2) If a subject is observed to have clear disease progression (an increase of 100% or more in 10mWT compared to baseline) by Day169 (Week 24).

(3) If a patient develops an adverse event that would make it difficult for them to continue participation in the study as determined by the principal investigator or subinvestigator.

(4) If a patient requests discontinuation.

(5) If HBV-DNA is detected in the patient.

(6) If a patient is found to be ineligible for the study

(7) When the principal investigator or subinvestigator determines that the patient should be discontinued from the study

For subjects who discontinue the study due to clinical exacerbation of HAM, data will be collected on routine visit frequency for “details of post-treatment (only treatment for HAM [including drugs listed in Exclusion Criterion 1 and Exclusion Criterion 6 and treatment with HAL]), “10-meter timed walk”, and “Osame’s Motor Disability Score” until the period corresponding to Week 24.

Rapid progressor（Clinical Trial①）

0.10Schedule Table（Rapid）

X = mandatory, (x) = optional

Period		Screening *1	1st Enrollment *3	Progressor assessment *2				2nd Enrollment *4	Study drug treatment period								Observation period				Unplanned visits *25	Post-observation *6	At discontinuation *7
Week		-16 ～ - 12*3		-12 *3	-8	-4	Last assessment *4		0			2	4	8	12	24 *5	28	32	36	48			
Day		-112～-84 ±7		-84 ±7	-56 ±7	-28 ±7	±7		1 *4	2	3	15 ±3	29 ±7	57 ±7	85 ±7	169 ±7	197 ±7	225 ±7	253 ±7	337 ±7			
Informed consent		x																					
Eligibility confirmation		x																					
Study drug treatment									→	→	→	→	→	→	→	→							
Clinical Assessment	Physical examination	x		x*2	x*2	x*2	x*2					x	x	x	x	x	x	x	x	x		x	x
Clinical Assessment	Blood pressure, Pulse rate, Body temperature	x		x*2	x*2	x*2	x*2					x	x	x	x	x	x	x	x	x			x*8
Clinical Assessment	Height, Body weight	x														x*9				x*9			
Clinical Assessment	OMDS ☆	x		x*2	x*2	x*2	x*2					x	x	x	x	x	x	x	x	x	x		x
Clinical Assessment	Walking tests *A ☆	x		x*2	x*2	x*2	x*2					x	x	x	x	x	x	x	x	x	x		x*10
Clinical Assessment	Walking aids *B ☆	x		x*2	x*2	x*2	x*2					x	x	x	x	x	x	x	x	x	x		
Clinical Assessment	MAS ☆	x		x*2	x*2	x*2	x*2					x	x	x	x	x	x	x	x	x			
Clinical Assessment	IPEC1 ☆	x		x*2	x*2	x*2	x*2					x	x	x	x	x	x	x	x	x			
Clinical Assessment	VAS *C ☆			x*2	x*2	x*2	x*2					x	x	x	x	x	x	x	x	x			

Rapid progressor（Clinical Trial①）

Period	Screening *1	1st Enrollment *3	Progressor assessment *2				2nd Enrollment *4	Study drug treatment period								Observation period				Unplanned visits *25	Post-observation *6	At discontinuation *7
Week	-16 ～ - 12 *3		-12 *3	-8	-4	Last assessment *4		0			2	4	8	12	24 *5	28	32	36	48			
Day	-112～-84 ±7		-84 ±7	-56 ±7	-28 ±7	±7		1 *4	2	3	15 ±3	29 ±7	57 ±7	85 ±7	169 ±7	197 ±7	225 ±7	253 ±7	337 ±7			
QOL *D ☆	x		x *2	x *2	x *2	x *2					x	x	x	x	x	x	x	x	x			
Urinary dysfunction *E ☆	x		x *2	x *2	x *2	x *2					x	x	x	x	x	x	x	x	x			
Blood tests *11 *F / Urinalysis *G	x		x *2	x *2	x *2	x *2				x *12	x	x	x	x	x	x		x	x			x
Virus tests *18 *H	x *13											x *14	x *14	x *14	x *14	x *15		x *15	x *15			x *16
Pregnancy test *17	x					(x) *2																
Cerebrospinal fluid tests *18 *I	x *19					x *2					x				x	(x) *20	(x) *20	(x) *20	x			
Accompanying research: Blood tests *J	x		x *2	x *2	x *2	x *2					x	x	(x)	x	x	x		x	x			
Accompanying research: Biobank samples (Blood) *21	x		x *2	x *2	x *2	x *2					x	x	(x)	x	x	x		x	x			
Accompanying research: Cerebrospinal Fluid tests *K	x *19					x *2					x				x	(x) *20	(x) *20	(x) *20	x			
Accompanying research: Biobank samples (Cerebrospinal fluid) *21	x *19					x *2					x				x	(x) *20	(x) *20	(x) *20	x			

Rapid progressor（Clinical Trial①）

Period	Screening *1	1st Enrollment *3	Progressor assessment *2				2nd Enrollment *4	Study drug treatment period								Observation period				Unplanned visits *25	Post-observation *6	At discontinuation *7
Week	-16 ～ - 12*3		-12 *3	-8	-4	Last assessment *4 ±7		0			2	4	8	12	24 *5	28	32	36	48			
Day	-112～-84 ±7		-84 ±7	-56 ±7	-28 ±7			1 *4	2	3	15 ±3	29 ±7	57 ±7	85 ±7	169 ±7	197 ±7	225 ±7	253 ±7	337 ±7			
MRI	x*22		x*22									(x)			x				(x)			
Intraocular pressure*23	x*22		x*22									x	x*24	x*24	x*24			x*24	x*24			

☆: The person in charge of assessment of the clinical efficacy endpoints will perform the evaluation

*1: Subjects who meet the rapid progressor criteria at screening will not need to undergo the progressor assessment period and may enter the primary and secondary enrollment and start the study drug treatment within 7 days of screening. In this case, the values at the screening will be the baseline value.

*2: The progressor assessment period will be 12 weeks. Subjects who meet the rapid progressor criteria during the progressor assessment period may enter the secondary enrollment and start the study drug treatment after all required tests and assessments have been performed on the last assessment date. The values at the screening will be used as the baseline values and the progressor assessment period is not required only if the period between screening and the start of the study drug treatment is less than 4 weeks. If the time between screening and initiation of study treatment exceeds 4 weeks, the last assessment date must be performed.

*3: The progressor assessment period on Week -12 and screening can be performed on the same day. In this case, the items specified in the screening should be performed. In addition, the primary enrollment should occur within 7 days of screening.

*4: Secondary enrollment should be performed within 7 days of the last assessment date to start Day 1 (study drug treatment). The date may be the same as the date of the final assessment.

*5: For subjects who discontinue the study due to clinical exacerbation of HAM, data will be collected on "details of the post-treatment regimen (for treatment of HAM [including the drugs listed in Exclusion Criterion 1 and Exclusion Criterion 6 and treatment with HAL], "10-meter timed walk", and "Osame's Motor Disability Score" until the period corresponding to Week 24 in the study treatment period.

*6: To be performed 28 days (+28 days) after the final dose of the study drug. Follow-up is not required if 28 days have passed since the final dose of the study drug at Week 48.

*7: To be performed within 28 days after discontinuation of the study drug during the treatment period or before the start of post-treatment, whichever is earlier.

Rapid progressor（Clinical Trial①）

*8: Body temperature will be measured as needed.

*9: Height measurement is not required.

*10: 10-meter timed walk, 6-minute walk distance, and 2-minute walk distance will be performed. Timed up-and-go test is not required.

*11: Fasting glucose will be measured on Day 3 of rechallenge in all subjects who are re-treated with methylprednisolone.

*12: Fasting blood glucose only will be measured for subjects in the pulse group.

*13: HBs antigen, HBs antibody, HBc antibody, HCV antibody, HIV-1 antibody and HIV-2 antibody will be tested only at screening. If either HBs antibody or HBc antibody is positive, quantitative HBV-DNA measurement will be performed.

However, quantitative HBV-DNA is not required if the positive HBs antibody is clearly due to vaccination.

*14: Quantitative HBV-DNA measurement will be performed only in subjects who are positive for HBs or HBc antibodies (with the exception of positive HBs antibody clearly due to vaccination) at screening and whose quantitative HBV-DNA is below the detection sensitivity.

*15: Subjects who are positive for HBs or HBc antibodies (with the exception of positive HBs antibody clearly due to vaccination) at screening and whose quantitative HBV-DNA is below the detection sensitivity will be closely monitored for liver function tests, and if abnormal values are observed (if AST and ALT increase is ≥ 5 times the reference value at the site), quantitative HBV-DNA measurement will be performed.

*16: For subjects who are positive for HBs or HBc antibodies (with the exception of positive HBs antibody clearly due to vaccination) at screening and whose quantitative HBV-DNA is below the detection sensitivity, if the liver function test shows abnormal values (if AST and ALT increase is ≥ 5 times the reference value at the site), quantitative HBV-DNA measurement will be performed.

*17: It is not required for subjects who are permanently sterilized. (Permanent sterilization: postmenopausal [with no menses for at least 48 weeks with no other medical reason], post sterilization [hysterectomy, bilateral salpingectomy, bilateral oophorectomy], tubal occlusion without tubal ligation)

*18: If data is available from a previous test on anti-HTLV-1 antibody for diagnosis of HAM, that data may be used.

*19: Data obtained within 12 weeks prior to the date of consent may be used.

*20: When restarting prednisolone treatment due to exacerbation after completion of the study drug treatment, it is recommended to perform a cerebrospinal fluid test before restart.

*21: Residual samples will be used as samples for biobanking.

*22: MRI and intraocular pressure measurement will be performed at any visit from screening to the progressor assessment period and the data will be used as the baseline value. For MRI scans, data obtained within 12 weeks prior to the date of informed consent can be used.

However, if an image used as the baseline shows findings suggestive of inflammation in the spinal cord, a retest must be performed within 12 weeks prior to the secondary enrollment for that site only. In addition, if data obtained within 12 weeks prior to the date of informed consent at another hospital is available and indicates no findings of inflammation in the spinal cord, a retest is not required.

Rapid progressor（Clinical Trial①）

*23: Intraocular pressure will be measured at the visit corresponding to 4 weeks (± 7 days) after rechallenge with methylprednisolone in all subjects.

*24: To be performed only in subjects with glaucoma.

*25: Unscheduled visit is allowed to confirm the extent of disease progression. At the unscheduled visit, only the Osame's Motor Disability Score, the walk test (10-meter timed walk), and the use of walking aids will be performed.

*A：Waling tests (10 meter Timed Walk, 6 minute Walk Distance, 2 minute Walk Distance, Timed Up and Go Test)

*B：Ambulatory aid used at the clinic and home

*C：VAS（Overall, Walking, Lower extremity pain）

*D：QOL (Modified IPEC2, N-QOL, Sexual Health Inventory for Men）

*E：Urinary dysfunction (OABSS, ICIQ-SF, IPSS)

*F：Blood tests： Complete blood count with differential, Urea nitrogen and electrolytes (BUN, Cre, Na, K), Liver function (AST, ALT, ALP, T-Bil),
Glucose metabolism (HbA1c (NGSP), Fasting blood glucose), Bone metablism (Ca, P), Lipids (T-Cho, LDL-C, HDL-C, TG)

*G：Urinalysis: glucose, protein

*H：Virus tests: Hepatitis B virus (HBc antibody ((at screening only), HBs antibody (at screening only), HBs antigen (at screening only), HBV-DNA)
HCV antibody (at screening only), HIV-1 antibody･HIV-2 antibody (at screening only)
Anti-HTLV-1 antibody for HAM diagnosis (at screening only)

*I：Cerebrospinal fluid tests: Anti-HTLV-1 antibody for HAM diagnosis (at screening only)
Safety: Glucose
Efficacy: Cell counts/ Cell fractionation, Toral protein, neopterin

*J：Blood tests (Accompanying research): β2-microgloblin, B- and T-cell subsets/markers、sIL-2R、HTLV-1 proviral load, sVCAM-1、SPARC

*K：Cerebrospinal fluid tests (Accompanying research): HTLV-1 anribody, HTLV-1 proviral load, CXCL10, sVCAM-1

Slow progressor（Cllinical Trial②）

Slow progressor

0.4 Objectives（Slow）

- Primary objective

  To test the efficacy of oral prednisolone therapy in patients with slowly progressive HAM/TSP.

- Secondary objective

  To test the safety of of oral prednisolone therapy in patients with slowly progressive HAM/TSP.

0.5 Study Design and Evaluation（Slow）

- Study design: Double-blind, randomized, placebo-controlled trial

- Primary outcome measure
  - Efficacy

    ＜Comparison between the predinisolone group and the placebo group＞

    Change in 10mWT at Day169 (Week 24) from baseline

- Secondary outcome measures
  - Efficacy

    ＜Comparison between the predinisolone group and the placebo group＞

    - 10mWT
      Change at Day29 (Week 4)/ Day85 (Week 12) compared to baseline
    - 2-minute walk distance and 6-minute walk distance
      Change at Day29 (Week 4) / Day85 (Week 12)/ Day169 (Week 24) compared to baseline
    - CSF neopterin concentration
      Change at Day169 (Week 24) compared to baseline
    - Proportion of subjects who discontinued the study drug during the study drug treatment priod (Day 1 ~ Day169 (Week 24))

    ＜Comparison in the placebo group＞

    - Walking tests (10mWT, 2-minute and 6-minute walk distance)
      Change at Day169 (Week 24) compared to baseline and at Day337 (Week 48) compared to Day169 (Week 24)
    - CSF neopterin concentration
      Change at Day169 (Week 24) compared to baseline and at Day337 (Week 48)

compared to Day169 (Week 24)

- Safety

  Adverse events (frequency, severity)

0.6 Target Sample Size and Study Period（Slow）

- Target sample sizse: 40 subjects
- Study period: August 2016 to August 2021

  （The secondary enrollment is possible until the end of June 2020.）

  However, the study period may be changed depending on the status of study progress.

0.7 Study Drug（Slow）

Prednisolone or placebo that matches with prednisolone and cannot be differentiated from the active drug.

Slow progressor（Clinical Trial②）

0.8 Treatment Plan（After Secondary Enrollment）（Slow）

Figure 4 : Treatment flow chart (Slow progressor)

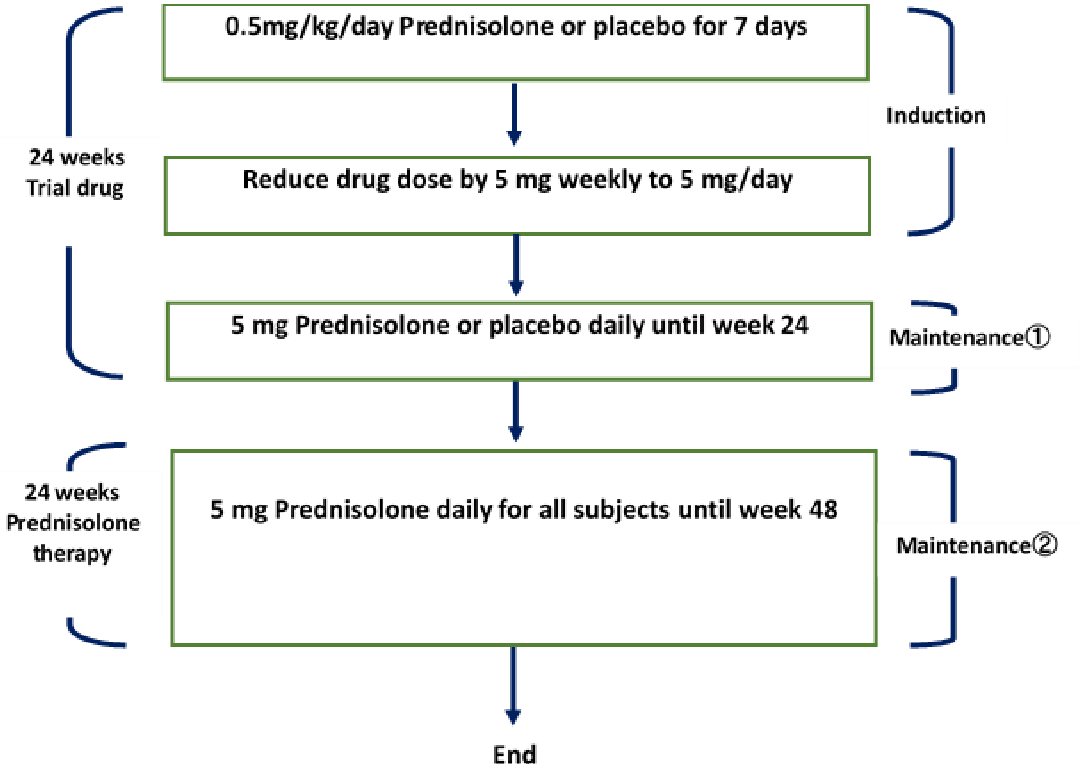

No additional treatment until Week 24. If a subject experienced worsening of symptoms of HAM and meets the criteria for dose increase or additional dose increase between the Week 24 visit and Week 48, additional treatment shall be performed according to the procedure.

Prior to starting the study drug treatment, it shall be confirmed that the test values on the last assessment date meet the following criteria.

① Neutrophil count: ≥1,500/mm3
② Platelets: ≥100,000/mm3
③ Hemoglobin: ≥9.0 g/dL
④ AST: ≤3-times the upper limit of normal (ULN)
⑤ ALT: ≤3-times the ULN
⑥ Serum creatinine: ≤1.5-times the UL
⑦ HbA1c（NGSP）: ≤6.5%

Slow progressor（Clinical Trial②）

＜Explanation for Figure 4＞

Patients are treated in the outpatient or inpatient setting.

Induction･Maintenance ① Treatment is prescribed according to the treatment plan (Figure 4).

Visits: Week 4 (Day29±7), Week 12 (Day85±7), Week 24 (Day169±7)

- Patients are initiated on 0.5mg/kg prednisone or placebo per day for 7 days [Day1～Day7]
- The drug dose is tapered in 5mg weekly decrements (every 7 days) to 5 mg prednisolone or placebo once daily. For the remaining treatment time until Week 24 is reached, patients are treated with 5 mg once daily [Day8～Day169]

Maintenance ②

Visits: Week 28 (Day197±7), Wek 32 (Day225±7), Week 36 (Day253±7), Week 48 (Day337±7)

- All subjects shall take prednisolone 5 mg/day daily. [Until Day 337]

The key code shall not be opened until the end of the study period (48 weeks) for all slow progressors.

0.8.1. Dose change (dose increase) after Week 24 visit

If a subject experienced worsening of symptoms of HAM and meets the criteria for dose increase or additional dose increase after the Day 169 (Week 24) visit, additional treatment shall be performed according to the procedure (maximum dose of 10 mg/day at the time of the increase) within 7 days from the date when it is confirmed that the subject meets the criteria. Dose increase or additional dose increase are permitted based on the values of the 10-meter timed walk and the OMDS obtained on an unscheduled study visit.

If a subject meets the dose increase/resumption criteria, the HAMLET-P Coordinating Center should be contacted before the dose increase/resumption.

After the dose increase, no transition to the next dose level shall be made until the end of the evaluation, regardless of the acceptable range.

➢ Determination based on OMDS

[Dose increase criteria]

"≥1 grade of worsening in the OMDS" compared to Day169 (Week 24)

[Dose increase procedures]

Prednisolone is increased by 2.5 mg/day (5 mg every other day) /4 weeks.

Slow progressor（Clinical Trial②）

➢ Determination based on the walk test

① [Dose increase criteria]

≥10% worsening in the 10-meter timed walk compared to the value on Day 169 (Week 24) was observed in the test at 2 study visits after Day 169 (Week 24). (Those 2 visits can be apart from each other.) If the walk test cannot be performed on Day 169 (Week 24) and the value is missing, the most recent result prior to Day 169 (Week 24) shall be used as the value on Day 169 (Week 24).

[Dose increase procedures]

The dose of prednisolone shall be increased to 7.5 mg/day (5 mg or 10 mg every other day) and the oral administration is continued at that dose.

② [Additional increase criteria]

If a test performed at the visit after an increase to 7.5 mg/day (5 mg/10 mg every other day) shows a 10% or more worsening in the 10-meter timed walk compared to the value observed at the time of the increase

[Additional increase procedures]

The dose of prednisolone shall be increased to 10.0 mg/day and the oral administration is continued at that dose.

0.8.2. Prednisolone Administration Rules

① Oral administration after breakfast is recommended. Dose

- Do not split or crush the study drug.
- The dose should be set in 2.5 mg increments
- For the dose of 0.5 mg/kg/day, round off the fraction.

② Timing of oral administration

- Take the drug once a day.
- Oral administration after breakfast is recommended.

0.9 Criteria for Study Discontinuation（Slow）

If a patient meets any of the following restrictions, said patient will be discontinued from the study:

(1) Up to Day 169 (Week 24):

Unequivocal HAM-induced disease progression (at least 100% worsening of 10-meter timed walk compared to baseline)

After Day 169 (Week 24) visit:

Slow progressor（Clinical Trial②）

Unequivocal progression of HAM-induced disease progression (at least 100% worsening of 10-meter timed walk compared to baseline) despite additional treatment

(2) If a subject develops steroid-induced glaucoma.

(3) If a patient develops an adverse event that would make it difficult for them to continue participation in the study as determined by the principal investigator or subinvestigator.

(4) If HBV-DNA is detected in the patient.

(5) If a patient requests discontinuation.

(6) If a patient is found to be ineligible for the study

(7) When the principal investigator or subinvestigator determines that the patient should be discontinued from the study

For subjects who discontinue the study due to clinical exacerbation of HAM, data will be collected on routine visit frequency for “details of post-treatment (only treatment for HAM [including drugs listed in Exclusion Criterion 1 and Exclusion Criterion 6 and treatment with HAL]), “10-meter timed walk”, and “Osame’s Motor Disability Score” until the period corresponding to Week 24.

Slow progressor（Clinical Trial②）

0.10Schedule Table（Slow）

X = mandatory, (x) = optional

	Period	Screening	1st Enrollment*1	Progressor Assessment				2nd Enrollment*2	Study drug treatment				Prednisolone treatment				Unplanned Visits*20	Post-observation*4 +28	At discontinuation*5
	Week	-16 ～ - 12*1		-12*1	-8	-4	Last assessment*2 ±7		0	4	12	24*3	28	32	36	48			
	Day	-112～-84 ±7		-84 ±7	-56 ±7	-28 ±7			1*2	29 ±7	85 ±7	169 ±7	197 ±7	225 ±7	253 ±7	337 ±7			
	Informed consent	x																	
	Eligibility confirmation	x																	
	Study drug treatment								Prednisolone or Placebo				Prednisolone						
Clinical Assessment	Physical examination	x		x	x	x	x	Randomization		x	x	x	x	x	x	x		x	x
Clinical Assessment	Blood pressure, Pulse rate, Body temperature	x		x	x	x	x	Randomization		x	x	x	x	x	x	x			x*6
Clinical Assessment	Height, Body weight	x						Randomization				x*7				x*7			
Clinical Assessment	OMDS	x		x	x	x	x	Randomization		x	x	x	x	x	x	x	x		x
Clinical Assessment	Walking tests *A	x		x	x	x	x	Randomization		x	x	x	x	x	x	x	x		x*8
Clinical Assessment	Walking aids *B	x		x	x	x	x	Randomization		x	x	x	x	x	x	x	x		
Clinical Assessment	MAS	x		x	x	x	x	Randomization		x	x	x	x	x	x	x			
Clinical Assessment	IPEC1	x		x	x	x	x	Randomization		x	x	x	x	x	x	x			
Clinical Assessment	VAS *C	x		x	x	x	x	Randomization		x	x	x	x	x	x	x			

Slow progressor（Clinical Trial②）

Period		Screening	1st Enrollment*1	Progressor Assessment				2nd Enrollment*2	Study drug treatment				Prednisolone treatment				Unplanned Visits*20	Post-observation*4 +28	At discontinuation*5
Week		-16 ～ - 12*1		-12*1	-8	-4	Last assessment*2		0	4	12	24*3	28	32	36	48			
Day		-112～-84 ±7		-84 ±7	-56 ±7	-28 ±7	±7		1*2	29 ±7	85 ±7	169 ±7	197 ±7	225 ±7	253 ±7	337 ±7			
	QOL*D	x		x	x	x	x			x	x	x	x	x	x	x			
	Urinary dysfunction*E	x		x	x	x	x			x	x	x	x	x	x	x			
Blood tests*F/ Urinalysis*G		x		x	x	x	x			x	x	x	x			x			x
Virus tests*14*H		x*9								x*11	x*10	x *10	x*11			x*11			x *12
Pregnancy test*13		x					(x)												
Cerebrospinal fluid tests*14 *I		x*15					x			(x)		x		(x) *16	(x) *16	x			
Accompaning research	Blood tests*J	x		x	x	x	x		x	x	x	x	x			x			
Accompaning research	Biobank samples (Blood)*17	x		x	x	x	x		x	x	x	x	x			x			
Accompaning research	Cerebrospinal Fluid tests *K	x*15					x			(x)		x		(x) *16	(x) *16	x			
Accompaning research	Biobank samples (Cerebrospinal fluid)*17	x*15					x			(x)		x		(x) *16	(x) *16	x			
MRI		x*18		x*18						(x)		x				(x)			
Intraocular pressure		x*18		x*18						x	x x	x			x	x			

Slow progressor（Clinical Trial②）

Period	Screening	1st Enrollment*1	Progressor Assessment				2nd Enrollment*2	Study drug treatment				Prednisolone treatment				Unplanned Visits*20	Post-observation*4	At discontinuation*5
Week	-16 ～ - 12*1		-12*1	-8	-4	Last assessment*2		0	4	12	24*3	28	32	36	48			
Day	-112～-84 ±7		-84 ±7	-56 ±7	-28 ±7	±7		1*2	29 ±7	85 ±7	169 ±7	197 ±7	225 ±7	253 ±7	337 ±7		+28	
										*19 *19	*19			*19	*19			

＊1: The progressor assessment period on Week -12 and screening can be performed on the same day. In this case, the items specified in the screening should be performed. In addition, the primary enrollment should occur within 7 days of screening.

＊2: Secondary enrollment should be performed within 7 days of the last assessment date to start Day 1 (study drug treatment). The date may be the same as the date of the final assessment.

＊3: For subjects who discontinue the study due to clinical exacerbation of HAM, data will be collected on “details of the post-treatment regimen (for treatment of HAM [including the drugs listed in Exclusion Criterion 1 and Exclusion Criterion 6 and treatment with HAL], “10-meter timed walk”, and “Osame’s Motor Disability Score” until the period corresponding to Week 24 in the study treatment period.

＊4: To be performed 28 days (+28 days) after the final dose of the study drug.

＊5: To be performed within 28 days after discontinuation of the study drug during the treatment period or before the start of post-treatment, whichever is earlier.

＊6: Body temperature will be measured as needed.

＊7: Height measurement is not required.

＊8: 10-meter timed walk, 6-minute walk distance, and 2-minute walk distance will be performed. Timed up-and-go test is not required.

＊9: HBs antigen, HBs antibody, HBc antibody, HCV antibody, HIV-1 antibody and HIV-2 antibody will be tested only at screening. If either HBs antibody or HBc antibody is positive, quantitative HBV-DNA measurement will be performed.

However, quantitative HBV-DNA is not required if the positive HBs antibody is clearly due to vaccination.

*10: Quantitative HBV-DNA measurement will be performed only in subjects who are positive for HBs or HBc antibodies (with the exception of positive HBs antibody clearly due to vaccination) at screening and whose quantitative HBV-DNA is below the detection sensitivity.

Slow progressor（Clinical Trial②）

*11: Subjects who are positive for HBs or HBc antibodies (with the exception of positive HBs antibody clearly due to vaccination) at screening and whose quantitative HBV-DNA is below the detection sensitivity will be closely monitored for liver function tests, and if abnormal values are observed (if AST and ALT increase is ≥ 5 times the reference value at the site), quantitative HBV-DNA measurement will be performed.

*12: For subjects who are positive for HBs or HBc antibodies (with the exception of positive HBs antibody clearly due to vaccination) at screening and whose quantitative HBV-DNA is below the detection sensitivity, if the liver function test shows abnormal values (if AST and ALT increase is ≥ 5 times the reference value at the site), quantitative HBV-DNA measurement will be performed.

*13: It is not required for subjects who are permanently sterilized. (Permanent sterilization: postmenopausal [with no menses for at least 48 weeks with no other medical reason], post sterilization [hysterectomy, bilateral salpingectomy, bilateral oophorectomy], tubal occlusion without tubal ligation)

*14: If data is available from a previous test on anti-HTLV-1 antibody for diagnosis of HAM, that data may be used.

*15: Data obtained within 12 weeks prior to the date of consent may be used.

*16: When increasing prednisolone treatment due to exacerbation, it is recommended to perform a cerebrospinal fluid test before.

*17: Residual samples will be used as samples for biobanking.

*18: MRI and intraocular pressure measurement will be performed at any visit from screening to the progressor assessment period and the data will be used as the baseline value. For MRI scans, data obtained within 12 weeks prior to the date of informed consent can be used.

However, if an image used as the baseline shows findings suggestive of inflammation in the spinal cord, a retest must be performed within 12 weeks prior to the secondary enrollment for that site only. In addition, if data obtained within 12 weeks prior to the date of informed consent at another hospital is available and indicates no findings of inflammation in the spinal cord, a retest is not required.

*19: To be performed only in subjects with glaucoma at week 8, 12, 24, 36, and 48

*20: Unscheduled visit is allowed to confirm the extent of disease progression. At the unscheduled visit, only the Osame’s Motor Disability Score, the walk test (10-meter timed walk), and the use of walking aids will be performed.

*A：Waling tests (10 meter Timed Walk, 6 minute Walk Distance, 2 minute Walk Distance, Timed Up and Go Test)

*B：Ambulatory aid used at the clinic and home

*C：VAS（Overall, Walking, Lower extremity pain）

*D：QOL (Modified IPEC2, N-QOL, Sexual Health Inventory for Men）

*E：Urinary dysfunction (OABSS, ICIQ-SF, IPSS)

*F：Blood tests： Complete blood count with differential, Urea nitrogen and electrolytes (BUN, Cre, Na, K), Liver function (AST, ALT, ALP, T-Bil),
Glucose metabolism (HbA1c (NGSP), Fasting blood glucose), Bone metablism (Ca, P), Lipids (T-Cho, LDL-C, HDL-C, TG)

Slow progressor（Clinical Trial②）

*G：Urinalysis: glucose, protein

*H：Virus tests: Hepatitis B virus (HBc antibody ((at screening only), HBs antibody (at screening only), HBs antigen (at screening only), HBV-DNA)

HCV antibody (at screening only), HIV-1 antibody・HIV-2 antibody (at screening only)

Anti-HTLV-1 antibody for HAM diagnosis (at screening only)

*I：Cerebrospinal fluid tests: Anti-HTLV-1 antibody for HAM diagnosis (at screening only)

Safety: Glucose

Efficacy: Cell counts/ Cell fractionation, Toral protein, neopterin

*J：Blood tests (Accompanying research): β2-microgloblin, B- and T-cell subsets/markers、sIL-2R、HTLV-1 proviral load, sVCAM-1、SPARC

*K：Cerebrospinal fluid tests (Accompanying research): HTLV-1 anribody, HTLV-1 proviral load, CXCL10, sVCAM-1

Non progressor（Accompanying Research）

Non progressor

0.4 Objectives（Non）

To explore prognostic and progressive disease categorical factors of clinical progression in patients with HAM.

0.5 Study Design and Evaluation（Non）

- Study design: Accompanying study
- Endpoints: Exploratory investigation of prognostic and progressive disease categorical factor

0.6 Target Sample Size and Study Period（Non）

- Target sample sizse: 5 subjects
  After reaching the target sample size of 5 subjects, subject enrollment will be continued until the target sample size of 40 in slow progressors has been reached or until the end of the study period.
- Study period: August 2016 to August 2021
  （The secondary enrollment is possible until the end of June 2020.）
  However, the study period may be changed depending on the status of study progress.

0.7 No study drug will be used（Non）

0.8 Treatment Plan（Non）

Patients are treated in the outpatient or inpatient setting.

Follow up

Visits：Week 12 (Day85±7days), Week 24 (Day169±7days), Week 48 (Day337±7days)

As an option, Monthly visits are allowed to confirm the extent of disease progression.

- Follow up patients until Week48.

<Change of progressor category>

If a subject experiences worsening of symptoms during the observation period up to Week 48 and meets the definition of the rapid or slow progressor, the subject will finish the non-progressor study and start the secondary enrollment as a rapid or slow progressor, with the study treatment + observation according to the protocol for respective progressor. Test items specified on the “final assessment date” for the

rapid or slow progressor will be performed before the secondary enrollment.

0.9 Criteria for Study Discontinuation（Non）

If a patient meets any of the following restrictions, said patient will be discontinued from the study:

(1) If a patient requests discontinuation.

(2) If a patient is found to be ineligible for the study

(3) When the principal investigator or subinvestigator determines that the patient should be discontinued from the study

Non progressor（Accompanying Research）

0.10Schedule Table（Non）

X = mandatory, (x) = optional

Period		Screening	1st Enrollment*1	Progressor Assessment				Observation				Observation	
Week		-16 ～ - 12*1		-12*1	-8	-4	Last assessment*2	(x)*3	12	(x)*3	24	(x)*3	48
Day		-112～-84 ±7		-84 ±7	-56 ±7	-28 ±7	±7		85 ±7		169 ±7		337 ±7
Informed consent		x											
Eligibility confirmation		x											
Observation								Observation					
Clinical Assessment	Physical examination	x		x	x	x	x		x		x		x
	Blood pressure, Pulse rate, Body temperature	x		x	x	x	x		x		x		x
	Height, Body weight	x									x*4		x*4
	OMDS	x		x	x	x	x	x	x	x	x	x	x
	Walking tests *A	x		x	x	x	x*2	x	x	x	x	x	x
	Walking aids *B	x		x	x	x	x	x	x	x	x	x	x
	MAS	x		x	x	x	x		x		x		x
	IPEC1	x		x	x	x	x		x		x		x
	VAS *C	x		x	x	x	x		x		x		x
	QOL*D	x		x	x	x	x		x		x		x
	Urinary dysfunction*E	x		x	x	x	x		x		x		x
Blood tests*F/ Urinalysis*G		x		x	x	x	x				x		x

Non progressor（Accompanying Research）

Period		Screening	1st Enrollment*1	Progressor Assessment				Observation				Observation	
Week		-16 ～ - 12*1		-12*1	-8	-4	Last assessment*2	(x)*3	12	(x)*3	24	(x)*3	48
Day		-112～-84 ±7		-84 ±7	-56 ±7	-28 ±7	±7		85 ±7		169 ±7		337 ±7
Virus tests *7*H		x*5											
Pregnancy test *6		x											
Cerebrospinal fluid tests *7 *I		x*8					(x)				(x)		(x)
Accompaning research 意）	Blood tests *J	x		x	x	x	x				x		x
	Biobank samples (Blood)*9	x		x	x	x	x				x		x
	Cerebrospinal Fluid tests *K	x*8					(x)				(x)		(x)
	Biobank samples (Cerebrospinal fluid) *9	x*8					(x)				(x)		(x)
MRI		x*10		x*10							x		x
Intraocular pressure		x*10		x*10									

*1: The progressor assessment period on Week -12 and screening can be performed on the same day. In this case, the items specified in the screening should be performed. In addition, the primary enrollment should occur within 7 days of screening.

*2: The day after the implementation date of the walk test in the final assessment is set as Day 1 for non-progressor.

*3: Monthly visits are allowed to confirm the extent of disease progression. If a subject has a visit, the subject will be evaluated for the Osame's Motor Disability Score, the walk test (10-meter timed walk, 6-minute walk distance, 2-minute walk distance, and timed up-and-go test), and the use of walking aids (at clinic and home)

*4: Height measurement is not required.

*5: HBs antigen, HBs antibody, HBc antibody, HCV antibody, HIV-1 antibody and HIV-2 antibody will be tested only at screening. If either HBs antibody or HBc antibody is positive, quantitative HBV-DNA measurement will be performed.

However, quantitative HBV-DNA is not required if the positive HBs antibody is clearly due to vaccination.

Non progressor（Accompanying Research）

*6: It is not required for subjects who are permanently sterilized. (Permanent sterilization: postmenopausal [with no menses for at least 48 weeks with no other medical reason], post sterilization [hysterectomy, bilateral salpingectomy, bilateral oophorectomy], tubal occlusion without tubal ligation)

*7: If data is available from a previous test on anti-HTLV-1 antibody for diagnosis of HAM, that data may be used.

*8: Data obtained within 12 weeks prior to the date of consent may be used.

*9: Residual samples will be used as samples for biobanking.

*10: MRI and intraocular pressure measurement will be performed at any visit from screening to the progressor assessment period and the data will be used as the baseline value of non progressor. For MRI scans, data obtained within 12 weeks prior to the date of informed consent can be used.

However, if an image used as the baseline shows findings suggestive of inflammation in the spinal cord, a retest must be performed within the progressor assessment period for that site only. In addition, if data obtained within 12 weeks prior to the date of informed consent at another hospital is available and indicates no findings of inflammation in the spinal cord, a retest is not required.

*A：Waling tests (10 meter Timed Walk, 6 minute Walk Distance, 2 minute Walk Distance, Timed Up and Go Test)

*B：Ambulatory aid used at the clinic and home

*C：VAS（Overall, Walking, Lower extremity pain）

*D：QOL (Modified IPEC2, N-QOL, Sexual Health Inventory for Men）

*E：Urinary dysfunction (OABSS, ICIQ-SF, IPSS)

*F：Blood tests：Complete blood count with differential, Urea nitrogen and electrolytes (BUN, Cre, Na, K), Liver function (AST, ALT, ALP, T-Bil),
Glucose metabolism (HbA1c (NGSP), Fasting blood glucose), Bone metablism (Ca, P), Lipids (T-Cho, LDL-C, HDL-C, TG)

*G：Urinalysis: glucose, protein

*H：Virus tests: Hepatitis B virus (HBc antibody ((at screening only), HBs antibody (at screening only), HBs antigen (at screening only), HBV-DNA)
HCV antibody (at screening only), HIV-1 antibody･HIV-2 antibody (at screening only)
Anti-HTLV-1 antibody for HAM diagnosis (at screening only)

*I：Cerebrospinal fluid tests: Anti-HTLV-1 antibody for HAM diagnosis (at screening only)
Safety: Glucose
Efficacy: Cell counts/ Cell fractionation, Toral protein, neopterin

Non progressor（Accompanying Research）

*J：Blood tests (Accompanying research): β2-microgloblin, B- and T-cell subsets/markers、sIL-2R、HTLV-1 proviral load, sVCAM-1、SPARC

*K：Cerebrospinal fluid tests (Accompanying research): HTLV-1 anribody, HTLV-1 proviral load, CXCL10, sVCAM-1

0.11 Contact Information

- HAMLET-P coordination center

[Before September 30, 2017]
Kanagawa Institute of Industrial Science and Technology (KISTEC)
Global health research coordinating center
E-mail: hamlet-p@newkast.or.jp

[After October 1, 2017]
Clinical research data center, St. Marianna University
E-mail: mariadc_ham@marianna-u.ac.jp

- **Enrollment Center and Data Center**

Kenichi Kawano
Division of Health Data Science, Translational Research Center for Medical Innovation.
Address: 1-5-4, Minatojimaminamimachi, Chuo-ku, Kobe, 650-0047, Japan.
Telephone: +81-78-303-9116
FAX: +81-78-303-9117
E-mail: hamlet-p-dc@tri-kobe.org

- **Chief Investigator**

Yoshihisa Yamano
Department of Rare Disease Research, Institute of Medical Science, St. Marianna University School of Medicine.
Address: 2-16-1, Sugao, Miyamae-ku, Kawasaki, 2168511, Japan
Telephone: +81-44-977-8111
Fax +81-44-977-9772
E-mail: yyamano@marianna-u.ac.jp

Table of contents

0. Overview 4
0.1 Study Plan 4
0.2 Eligibility Criteria 7
0.3 Definition of the progressor caterogy 9
Rapid progressor 12
0.4 Objectves (Rapid) 12
0.5 Study design and evaluation （Rapid） 12
0.6 Target Sample Size and Study Period （Rapid） 13
0.7 Study Drug（Rapid） 13
0.8 Treatment Plan（After Secondary Enrollment）（Rapid） 14
0.9 Criteria for Study Discontinuation 17
0.10 Schedule Table（Rapid） 20
0.4 Objectives（Slow） 25
0.5 Study Design and Evaluation（Slow） 25
0.6 Target Sample Size and Study Period（Slow） 26
0.7 Study Drug（Slow） 26
0.8 Treatment Plan（After Secondary Enrollment）（Slow） 27
0.9 Criteria for Study Discontinuation（Slow） 29
0.10 Schedule Table（Slow） 31
Non progressor 35
0.4 Objectives（Non） 35
0.5 Study Design and Evaluation（Non） 35
0.6 Target Sample Size and Study Period（Non） 35
0.7 No study drug will be used（Non） 35
0.8 Treatment Plan（Non） 35
0.9 Criteria for Study Discontinuation（Non） 36
0.10 Schedule Table（Non） 37
0.11 Contact Information 40
Table of contents 41
1. Background and Rationale 44
2. Study plan 46
3. Eligibility Criteria 48
4. Definition of the progressor caterogy 50
5. Informed Consent 52
6. Subject Enrollment 54

Rapid progressor 59
7. Objectves (Rapid) 59
8. Study design and evaluation（Rapid） 59
9. Target Sample Size and Study Period（Rapid） 60
10. Treatment Plan（After Secondary Enrollment）（Rapid） 61
11. Administration Discontinuation and Study Discontinuation（Rapid） 65
12. Drug Information（Rapid） 67
13. Concomitant Drugs and Therapies（Rapid） 71
14. Observation, Investigations, Test Items, and Implementation Period（Rapid） 75
15. Management of Patients（Rapid） 90
16. Evaluation Criteria/Response Evaluation（Rapid） 90
17. Data Reporting Method（Rapid） 91
18. Adverse Event Reporting（Rapid） 92
19. Statistical Analysis（Rapid） 96
Slow Progressor 100
7. Objectives（Slow） 100
8. Study Design and Evaluation（Slow） 100
9. Target Sample Size and Study Period（Slow） 101
10. Treatment Plan（After Secondary Enrollment）（Slow） 101
11. Administration Discontinuation and Study Discontinuation（Slow） 104
12. Drug Information（Slow） 106
13. Concomitant Drugs and Therapies（Slow） 108
14. Observation, Investigations, Test Items, and Implementation Period（Slow） 112
15. Management of Patients（Slow） 125
16. Evaluation Criteria/Response Evaluation（Slow） 125
17. Data Reporting Method（Slow） 126
18. Adverse Event Reporting（Slow） 127
19. Statistical Analysis（Slow） 131
Non progressor 136
7. Objectives（Non） 136
8. Study Design and Evaluation（Non） 136
9. Target Sample Size and Study Period（Non） 136
10. Treatment Plan（Non） 136
11. Study Discontinuation（Non） 137
12. Drug Information（Non） 137
13. Concomitant Drugs and Therapies（Non） 137

14. Observation, Investigations, Test Items, and Implementation Period（Non） 139
15. Management of Patients（Non） 151
16. Evaluation（Non） 151
17. Data Reporting Method（Non） 151
18. Adverse Event Reporting（Non） 152
19. Statistical Analysis（Non） 153
20. Accompanying Research 154
21. Ethical Considerations and Good Clinical Practice（GCP） 155
22. Monitoring and Audits... 159
23. Efficacy and Safety Evaluation Committee 160
24. Retention of Records 160
25. Research Costs for the Study 161
26. Attribution and Publication of Research Results 161
27. Research Structure (Japan) 161
28. Rationale 164
29. References 171
30. Appendix 1 173

1. Background and Rationale

HTLV-1-associated myelopathy (HAM) is a disease characterized by progressive spinal palsy due to chronic inflammation in the spinal cord, which is a very severe and refractory rare disease with high unmet need[1)]. The number of patients in Japan is estimated to be around 1500 to 3000 patients, and the number of patients who develop the disease annually is estimated to be around 50 patients. However, since Japan is the only country in developed countries which has many patients with HAM and there are few patients in Western developed countries, high quality evidence of treatment and biomarkers is poor. Therefore, currently no international standard of care for HAM has been established that leads to extremely low quality of practice and it is imperative to establish a treatment to improve the long-term prognosis of patients.

It has been revealed in previous studies that HAM consists of populations with different disease activities defined by disease onset patterns, clinical course and others[2-6)]. This means that the patient population can be divided into a group of more active patients with progressive course and a group of less active patients with little progression for a long-period of time (non progressors), and the group of more active patients includes a group of patients with relatively rapid progression (rapid progressors) and a group of patients with slower progression (slow progressors). Thus, the disease activity of HAM varies significantly among individuals and these characteristics should be considered for decision of treatment strategy, but it has not been achieved yet due to a lack of report from clinical study that is designed by disease activity. In addition, different disease activities result in different future prognosis of HAM[3)], it is necessary to understand disease activity as early as possible and determine the treatment strategy accordingly, which requires identification of biomarkers that correlate with progression and can predict prognosis. Recently, a retrospective study has reported that the cerebrospinal fluid concentration of CXCL10 and neopterin are strongly correlated with the degree of progression[7)]. While these markers are promising candidates, they need to be tested in prospective studies to be established as predictive factors of the degree of progression.

Steroid treatment is also listed as the first candidate for international standard of care for HAM. There have long been reports of the efficacy of steroid treatment in HAM[8-11)], and it is still the most widely used treatment in the world today[1)]. In a survey of current treatment for HAM conducted by the Ministry of Health, Labour and Welfare research team in Japan in the 2014 fiscal year[2)], the percentage of patients using interferon-alpha, that is a treatment approved for medical insurance in Japan, was low at approximately 3 to 4%, while the percentage of patients continuously using any oral steroid, that is not approved for medical insurance, was found to be high at approximately 45 to 50%. Thus, steroid treatment is

expected to have a progression-suppressing effect on HAM with a superior risk-benefit balance and is recommended in the HAM Treatment Manual (2015) issued by the Ministry of Health, Labour and Welfare research team[12)], but it has not been approved for medical insurance in Japan which raises issues from the perspective of patient safety and health insurance system. In this situation, the HAM patient advocacy group and the Japanese Society for Neuroimmunology (in a joint submission with the Japanese Society of Neurology and Japanese Society for Neuroinfectious Diseases) submitted a written request to the "Unapproved/Off-label Drug Review Meeting" in December 2013 seeking public knowledge-based application for use of methylprednisolone and prednisolone in treatment of HAM. However, submission of at least one randomized controlled study result is required for review at this meeting but the efficacy of steroid treatment for HAM have been reported only in case series studies[8-11)] rather than randomized controlled studies that results in a low level of evidence, and it is currently impossible to achieve a public knowledge-based application. Furthermore, because HAM is characterized by the fact that only Japan has a large number of patients in developed countries, it is difficult to conduct clinical studies in Western developed countries due to difficulty in enrollment of sufficient number of patients and it cannot be expected to get supporting reports from randomized comparative studies conducted in Western developed countries as is the case in other diseases.

Based on the background described above, a protocol of international collaborative clinical study of steroid treatment for HAM by disease activity including prospective verification of predictive factors for disease progression has been developed in cooperation with HAM researchers worldwide (Japan, US, UK and Brazil) to establish an international standard of care in view of disease activity. Furthermore in Japan, the protocol was translated with the intention of getting steroid treatment approved for medical insurance and the study was planned to be conducted as an investigator-initiated study.

2. Study plan

Figure 2: Study flow chart

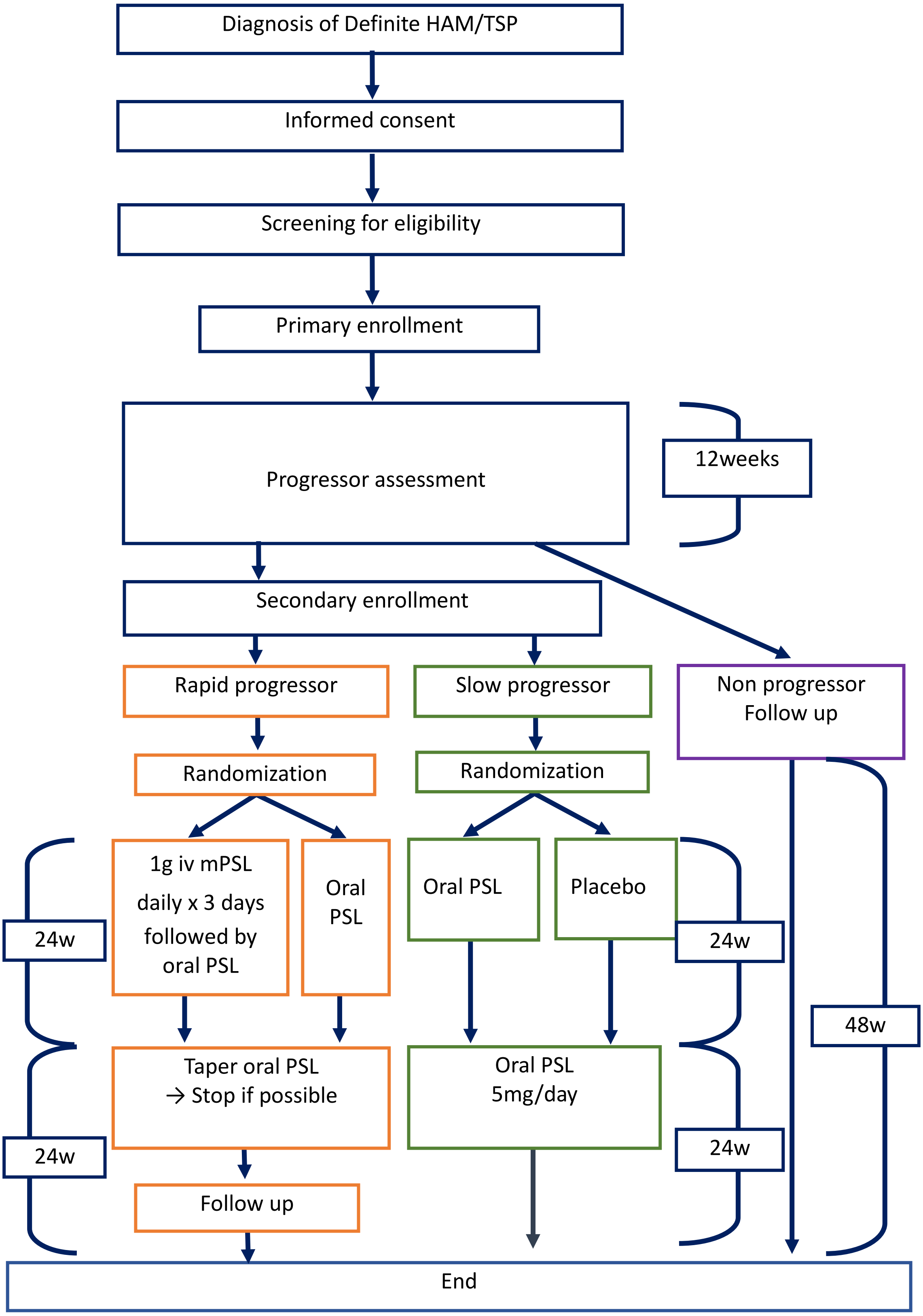

＜Explanation for Figure 2＞

Definite HAM/TSP

Patients with definite HAM/TSP who can walk 10 meter or more by themselves are potentially eligible for this study. The use of walking sticks but not wheelchairs or frames permitted.

Informed Consent

The principal investigator and subinvestigator provide the patient with information of the study and obtain written informed consent from the patients

Screening for Eligibility

Patients will be screened for eligibility by blood test, urinalysis, CSF test, and MRI.

MRI：MRI should be performed in the screening period. However, if it is difficult, data from previous tests may be used for eligibility screening.

If data obtained within 12 weeks prior to the date of informed consent at another hospital is available, a retest is not required.

Primary enrollment

Eligible subjects will be enrolled by web-based centralized enrollment.

Progressor assessment

During the period of baseline assessments (12 weeks), participants are recruited into one of three study sub-groups: rapid progressors, slow progressors or non-progressors depending on walking test and the evaluation of clinical history.

Patients with rapidly progressing disease are treated as soon as identified and do not have to complete the 12 weeks assessment period.

Slow and non progressors must complete the 12 weeks assessment period.

Secondary enrollment

The secondary enrollment and treatment assignment will be performed on the web for subjects determined as rapid or slow progressor. The secondary enrollment will not be performed for non progressor.

Treatmernt/Follow up

・ Rapid progressor will be treated with the study drug until Week 24, and followed up until Week 48.

・ Slow progressor will be treated with the study drug until Week 24, and with prednisolone until Week 48.
・ Non progressor will be followed up without treatment until Week 48.

For, details, refer to '10. Treatment Plan (After Secondary Enrollment).

3. Eligibility Criteria

3.1 Indication for Study

HTLV-1-associated myelopathy/tropical spastic paraparesis (HAM/TSP)
Diagnosis of HAM/TSP to Belem Criteria（2006）_Definite HAM/TSP

1. A non-remitting progressive spastic paraparesis with sufficiently impaired gait to be perceived by the patient. Sensory symptoms or signs may or may not be present. When present, they remain subtle and without a clear-cut sensory level. Urinary and anal sphincter signs or symptoms may or may not be present.
2. Presence of HTLV-I antibodies in serum and CSF confirmed by Western blot＊[1] or Line blot and/or a positive PCR for HTLV-1 in blood and/or CSF
3. Exclusion of other disorders* that can resemble HAM/TSP

   * : multiple sclerosis; carcinomatous meningitis; familial spastic paraparesis; transverse myelitis; primary lateral sclerosis; syringomyelia; Lyme disease; B 12 and folate deficiency; Behçet disease; neurosyphilis; neurotuberculosis; sarcoidosis; HIV vacuolar myelopathy; collagen vascular diseases; autoimmune myelopathies; Sjögren's syndrome; toxic myelopathies; amyotrophic lateral sclerosis; fungal myelopathy; spinal arteriovenous fistula; hepatic myelopathy; parasitic myelopathy (visceral larva migrans of *Toxocara canis* and *Ascaris suum*); spinal cord compression (spinal tumor, cervical spondylosis, brain parasagittal tumor etc.); endemic regional myelopathies with similar clinical manifestations (including schistosomiasis and neurocysticercosis

3.2 Inclusion criteria

(7) Patients diagnosed with HAM/TSP according to the diagnostic guidelines described in Section 3.1
(8) Patients who are 18 years of age or older at the time when informed consent is obtained
(9) Patients who are capable of walking at least 10 meters (regardless of whether a walking aid is used or not) at the time when informed consent is obtained
(10) Patients whose primary organ functions are stable

(According to the latest laboratory results from within a maximum of 28 days prior to the date of enrolment)

⑧ Neutrophil count: ≥1,500/mm^3

⑨ Platelets: ≥100,000/mm^3

⑩ Hemoglobin: ≥9.0 g/dL

⑪ AST: ≤3-times the upper limit of normal (ULN)

⑫ ALT: ≤3-times the ULN

⑬ Serum creatinine: ≤1.5-times the UL

⑭ HbA1c（NGSP）: ≤6.5%

(11) Patients who have given written consent to participate in the study of their own free will

(12) Patient must be willing and able to comply with all the aspects of trial design and follow-up

3.3 Exclusion criteria

(1) Patients who have received corticosteroids or other immune-modulating or anti-viral agent that alter the immune response to HTLV-1 within 12 weeks of entering the study if rapidly progressing

(2) Patients who have received corticosteroids or other immune-modulating or anti-viral agent that alter the immune response to HTLV-1 within 48 weeks if slowly progressing or not progressing

(3) Patients who have undergone highly invasive surgery under general anesthesia within 24 weeks prior to giving informed consent

(4) Patients who have received other study drugs within 16 weeks prior to giving informed consent

(5) Patients who have undergone live or attenuated/inactivated vaccinations within 4 weeks prior to the date of enrollment, or who plan on being vaccinated during the study period

(6) Patients who have taken ascorbic acid (≥1.5 g/day), prosultiamine, or pentosan polysulfate within 2 weeks prior to the date of enrollment

(7) Patients with a history of myocardial infarction

(8) Patients with a history of tuberculosis or with active tuberculosis

(9) Patients with serious complications (heart failure, lung disease, renal failure, hepatic failure, uncontrolled diabetes mellitus, etc.)

(10) Patients with uncontrolled high blood pressure

(11) Patients with uncontrolled electrolyte disorder

(12) Patients with thrombotic disease

(13) Patients who have a history of cancer, or those complicated with cancer

However, patients with radically resected solid tumor which has not recurred for at least 3 years before enrollment will be able to enroll in the study. Patients with radically resected basal cell carcinoma of the skin, squamous cell carcinoma (except malignant melanoma), non-invasive cervix carcinoma, and carcinoma in situ in the gastrointestinal tract or corpus of the uterus will be able to enroll in the study if they are determined to be completely cured even if within 3 years of enrollment

(14) Patients with peptic ulcer
(15) Patients complicated with adult T-cell leukemia/lymphoma (ATL)
(16) Patients with uncontrolled eye disease
(17) Patients with a history of steroid-induced glaucoma
(18) Patients who are pregnant, breastfeeding, who may be pregnant, or wish to bear children
(19) Patients with complications of spinal cord compressive lesions such as spondylitis, ossification of the posterior longitudinal ligament, and ossification of the yellow ligament, or articular diseases such as rheumatoid arthritis and osteoarthritis
(20) Patients with neurological disorder or MRI findings attributable to other disease
(21) Patiens with a history of spine compression fracture
However, patients with traumatic fracture or those with ≥70% of lumber spine bone mineral density (YAM) will be able to enroll in the study.
(22) Patients with psychiatric disorder, epilepsy, or dementia
However, patients with epilepsy who have experienced no seizures for 3 years before enrollment will be able to enroll in the study.
(23) Patients who test positive for HBs antigen or HBV-DNA (using real-time PCR
(24) Patients who test positive for HIV antibody
(25) Patiens with a history of strongyloidis stercoralis infection
(26) Patients with systemic bacterial or fungal infection
(27) Patients who need medications that strongly induce or inhibit CYP3A4.
(28) Patients with a history of allergic reaction to the study drug
(29) Patients considered unqualified to participate in the study by the principal investigator or subinvestigator

4. Definition of the progressor caterogy

Subjects will be classified into three clinical subgroups (rapid-progressor, slow-progressor, or non-progressor) based on clinical history and the change in the 10 meter timed walk (10mWT) during the 12 week run-in. Clinical history will be assessed using OMDS. Grade 1 and Grade 2 of the original OMDS will be treated as Grade 2 in the study. The average of

two measuments of 10mWT (rounded to two decimal place) will be used for judgement.

- Rapid progressor:

Subjects who fulfil the criteria based on clinical history will be categorized as rapid progressors without 12-week assessment. Where patients cannot be categorized as rapid progressors based on clinical history, they are followed up during the baseline assessment. The baseline assessment can be shortened if rapid clinical deterioration is confirmed.

[Assessment based on clinical history]

Patients display at least one of the following:

- Deterioration in the 10mTW ≥ 30% within the previous 12 weeks
- Deterioration in OMDS ≥ 1 Grade within the previous 12 weeks

[12-week Progressor assessment]

Patients display at least one of the following:

- Deterioration in the 10mTW of ≥ 30% during the 12 weeks of assessment
- Deterioration in OMDS of ≥ 1 Grade during the 12 weeks of assessment
- Non progressors who display, during the 48 weeks of follow up, deterioration in the 10mTW of ≥ 30% compared with the best record in the baseline assessment period.

- Slow progressor:

Patients display at least one of the following:

- Deterioration in the 10mTW of ≥ 10% but < 30% at the end of the 12 weeks of assessment
- Non progressors who display, during the 48 weeks of follow up, deterioration in the 10mTW of ≥ 10% but < 30% compared with the best record in the baseline assessment period.

- Non progressor:

These patients do not display, at the end of the 12 weeks of observations, any deterioration in the 10mTW that is more than or equal to 10%.

＜*1 **Osame Motor Disability Scale**＞

Grade	Motor disability
**0**	Normal gait and running
**1**	Normal gait but runs slowly } *1
**2**	Abnormal gait (staggering or spastic) } *1
**3**	Abnormal gait and unable to run
**4**	Needs support while using stairs but walks without assistance
**5**	Needs one hand support in walking
**6**	Needs two hands support in walking (can walk more than 10 meter)
**7**	Needs two hands support in walking (can walk less than 10 meter)
**8**	Needs two hands support in walking (can walk less than 5 meter)
**9**	Unable to walk but can walk on all fours
**10**	Unable to walk on all fours but can crawl with hands
**11**	Unable to crawl with hands but can turn sideways in bed
**12**	Unable to turn sideways but can move the toes
**13**	Completely bedridden (unable to move the toes)

[Notes for the OMDS assessment in this study]

*1: To be rated as "Grade2"

5. Informed Consent

(1) Consent

- Prior to enrollment, the principal investigator and subinvestigator will provide the patient with an information sheet approved by the Institutional Review Board of the study site and a full explanation. After the patient is given an explanation of the study and confirmed to fully understand the content of the study, their intention to participate in the study will be confirmed.
- The physician giving an explanation and the patient receiving the explanation will write their names and the date of consent on the attached consent form and sign their names. A copy of the informed consent form will be handed to the patient and the original will be stored in the patient's medical records.

(2) Explanations given to patient

- The study includes research
- Study objectives
- Name and title of the investigators, contact information
- Design and method of the study
- Expected clinical risks and benefits
- Other treatment options for subjects
- Observation period
- Refusal to give consent and withdrawal of consent
- Direct access to the medical history by monitors and auditors
- The fact that the utmost effort will be made to keep the name of patients and personal information confidential.
- Compensation
- Planned number of subjects
- Prompt provision of new information that may affect the patient's willingness to continue participation in the study to participants
- Discontinuation or Interruption Criteria
- Cost burden of subjects
- Participant obligations
- Research review board
- Source of funds and conflict of interest
- Publication of research results
- Attribution of research results
- Protection of human rights

(3) Provision of information to patients, revisions to the informed consent form, and obtaining re-consent

When any new information that may affect the patient's willingness to continue participation in the study is obtained, the principal investigator or subinvestigator will promptly give an explanation of the details of such information to the patient participating in the study and then confirm whether or not the patient is willing to continue participation in the study. The details of the explanation, the date the explanation is given, the person confirming the patient's intention, and the patient's intention will be recorded in the patient's medical records.

When determining that a revision to the informed consent form is required regarding the already-given explanation, the principal investigator will revise the informed consent form and obtain approval from the Institutional Review Board. Subsequently, the

principal investigator or subinvestigator will give an explanation again using the revised informed consent form and obtain written re-consent from the patient to continue participation in the study by following the same procedures as the obtainment of the initial consent.

6. Subject Enrollment

6.1 Subject enrollment method

Subject enrollment for this study will be done by web-based centralized enrollment.
Subjects will be enrolled by the following two steps:

- Web-based primary enrollment.
- Web-based secondary enrollment and automatic assignment in rapid and slow progressors.

6.2 Subject enrollment (primary enrollment)

(1) Subject enrollment procedure

1) he principal investigator, etc. shall obtain written consent from the subject with confirmed diagnosis of HAM (if the subject is <20 years of age, the subject and his/her parent or guardian) and register all subjects in the screening list.
2) The principal investigator, etc. shall confirm that the subjects meet "3. Subject Inclusion Criteria" and perform input of screening information and primary subject enrollment on the web.
3) Eligibility will be automatically confirmed on the web, and only eligible subjects complete the primary enrollment. The principal investigator, etc. shall confirm that the primary subject enrollment has been completed on the web screen.

6.3 Subject enrollment (secondary enrollment)

(1) Subject enrollment procedure

1) After the primary subject enrollment, the principal investigator, etc. shall assess the medical history, determine the progressor type (up to 12 weeks) and then perform the secondary subject enrollment of rapid or slow progressors on the web. Assignment will be automatically performed for rapid and slow progressors.
2) The principal investigator, etc. shall confirm the completion of secondary subject enrollment on the web page.

6.4 Emergency subject enrollment

If subject enrollment is not available for any reason such as temporary access failure to the web system by sites, the data center will accept subject enrollment by fax.
Enrollment by fax is limited to 10 a.m. to 3 p.m. on weekdays. (It is not available on Saturdays, Sundays, and holidays.)

6.5 Assignment for rapid and slow progressors

6.5.1. Randomization

(1) Rapid progressor

After the secondary enrollment, eligible subjects will be automatically assigned on the web. The blinded status will be maintained by blinding the person in charge of assessment of the following clinical efficacy endpoints: The results of assignment will be unlocked after the database lock.

＜Clinical efficacy assessment＞

Modified Ashworth scale, VAS, OMDS, Walking aid usage, walking tests (10mWT, 6-minute walk distance, 2-minute walk distance, Time up and go test), IPEC1, Urinary symptom scores（OABSS, ICIQ-SF, IPSS）, QOL（Modified IPEC2, N-QOL, Sexual Health Inventory for Men）

(2) Slow progressor

After the secondary enrollment, eligible subjects will be automatically assigned on the web. After automatic assignment, blinded drug numbers will be displayed on the web page. The results of assignment will be disclosed only to the “key controller”. Subjects will be randomized in a double-blinded manner and neither the subject nor the study staff (including the data center) can know which drug the subject received (active or placebo). Only the Efficacy and Safety Evaluation Committee will be allowed to ask the key controller to obtain this information in order to disclose it. After the database is locked for data on Week 24 or discontinuation by Week 24 other than neopterin concentration data, the results of assignment will be unblinded and the key controller will simultaneously confirm the emergency key codes remain unbroken other than those broken according to the specified procedures.

(3) Emergency key code breaking

The emergency key codes will be stored until the key controller breaks them. In the event that the principal investigator deems it necessary to know the treatment group of a subject in order to treat the subject or secure the safety in a medical emergency, the principal investigator shall discuss the necessity of breaking the emergency key code

with the coordinating investigator and the coordinating investigator shall report it to the chairperson of the Efficacy and Safety Evaluation Committee for decision of the chairperson. Only when a permission is obtained from the chairperson, the key controller may be contacted to break the code of the subject for which unblinding is deemed necessary on a case-by-case basis. The background of emergency key code breaking shall be reported to the Efficacy and Safety Evaluation Committee.

6.5.2. Assignment factors

(1) Rapid progressor

Subjects will be automatically assigned via the web using the permuted block method. The assignment factors are defined as follows:

① Number of canes (one or less/two or more*) *: A walker should be regarded as “two or more”.

Number of canes:

Subjects using a tripod cane in daily life should test whether they are able to walk in a 10-meter timed walk using a single-tip cane at the site (on a flat floor without obstructions) to determine the number of canes to be used as an assignment factor.

(2) Slow progressor

Subjects will be automatically assigned via the web using the minimization method. The assignment factors are defined by the following 3 factors:

① Sex (male/female)

② Number of canes (one or less/two or more*) *: A walker should be regarded as “two or more”.

Number of canes:

Subjects using a tripod cane in daily life should test whether they are able to walk in a 10-meter timed walk using a single-tip cane at the site (on a flat floor without obstructions) to determine the number of canes to be used as an assignment factor.

③ Trial site

6.6 Precautions when Enrolling Patients

(1) The principal investigator shall keep the screening list.

(2) Enrollment after the initiation of the treatment according to the protocol will not be accepted without exception.

(3) Enrollment of subjects cannot be withdrawn (removed from the database). For duplicate enrollments, the initial enrollment information (subject number) will be used.

(4) The Data Center must be notified if any erroneous or duplicate enrollment is found.

(5) If a non progressor experienced worsening of symptoms during the observation period up to 48 weeks and meets the definitions of rapid or slow progressor, the subject shall complete the study enrolled as a non-progressor and newly enrolled as a rapid or slow progressor by the secondary enrollment. In this case, the test items specified on the “Date of Last Assessment” for rapid or slow progressor shall be performed prior to the secondary enrollment.

(6) If a subject was planned to be enrolled as a rapid progressor but is found not to fall into a rapid progressor prior to the secondary enrollment, the subject shall withdraw from the study and may participate the study as a new subject upon providing the written consent again if the criteria described below are met. In this case, the procedures shall be started from the primary enrollment.

① If registered as a rapid progressor

Primary enrollment can be performed if the subject meets the criteria for rapid progressor. However, it must be confirmed that the subject meets the eligibility criteria (inclusion and exclusion criteria).

② If registered as a slow progressor or non-progressor

Primary enrollment can be performed when the subject becomes not to meet the exclusion criteria (2) of “in slow progressors or non progressors, patients who have received corticosteroids or other HAM-targeted therapeutic agents within 48 weeks prior to the date of informed consent”. However, it must be confirmed that the subject meets the eligibility criteria (inclusion and exclusion criteria).

<Reuse of screening test data if a subject participates the study again>

Data from the screening tests performed prior to re-enrollment may be used if it meets the following windows:

Evaluation	Screening	Allowable range of data reuse
Clinical assessmemt	x	Data obtained within 4 weeks prior to the date of consent may be used (Data of hight obtained within 8 weeks prior to the date of consent may be used)
Safety<br>Blood tests/ Urinalysis	x	Within 4 weeks prior to the date of consent (within 12 weeks for HBV)
Pregnancy test	x	Within 8 weeks prior to the date of consent
Blood tests for research	x	Within 4 weeks prior to the date of consent
Biobank samples	x	Within 4 weeks prior to the date of consent
MRI	x	Within 12 weeks prior to the date of consent (However, if an image used as the baseline shows findings suggestive of inflammation in the spinal cord, a retest must be performed within 12 weeks prior to the secondary enrollment for that site only.)
CSF tests	x	Within 12 weeks prior to the date of consent
Ocular pressure measurements	x	Within 8 weeks prior to the date of consent

Rapid progressor (Clinical trial①)

Rapid progressor

7. Objectves (Rapid)

- Primary objective

  To test the efficacy of i.v. methylprednisolone followed by oral prednisolone therapy in patients with rapidly progressive HAM/TSP compared to oral prednisolone alone.

- Secondary objectives

  To test the safety of i.v. methylprednisolone and oral prednisolone therapy in patients with rapidly progressive HAM/TSP.

  To test the efficacy of oral prednisolone therapy in patients with rapidly progressive HAM/TSP.

8. Study design and evaluation（Rapid）

- Study design ： Prospective, Randomized, Open, Blinded-Endopoint (PROBE)
- Primary outcome measures
  - Efficacy

    Presence or absence of “≥30% improvement in the 10-meter timed walk” or “≥1 improvement in the OMDS” at Day 15 compared to the baseline value
- Secondary outcome measures
  - Efficacy
    - 10mWT
      - Presence or absence of “≥30% improvement in the 10-meter timed walk” at Day 15 compared to the baseline value
      - Area under the curve (Baseline, Day15, Day29 (Week4))
      - Change at Day15 / Day29 (Week4) compared to baseline
    - 2-minute walk distance and 6-minute walk distance
      - Area under the curve (Baseline, Day15, Day29 (Week4))
      - Change at Day15 / Day29 (Week4) compared to baseline
    - OMDS

      Presence or absence of “≥1 improvement in the OMDS” at Day 15 compared to the baseline value
    - CSF neopterin concentration

      Change at Day15 compared to baseline
    - Proportion of subjects who received i.v. methylprednisolone between Day29（Week 4）and Day169（Week 24）
  - Safety

Adverse events（frequency, severity）

- Exprolatory outcomes
  - Efficacy
    - 10mWT
      Change at Day85 (Week12) / Day169 (Week24) compared to baseline
    - 2-minute walk distance and 6-minute walk distance
      Change at Day85 (Week12) / Day169 (Week24) compared to baseline
    - CSF neopterin concentration
      Change at Day169 (Week24) compared to baselin
    - Proportion of subjects who discontinue the study due to clinical exacerbation of HAM (an increase of 100% or more in 10mWT compared to baseline) between Day30 and Day169 (Week 24).
    - Proportion of subjects who could not discontinue the drug at Day183 (Week 26).
    - Proportion of subjects who resumed steroid treatment by Day337 (Week 48) among those who had discontinued the drug at Day183 (Week 26).

9. Target Sample Size and Study Period（Rapid）

- Target sample size: 8 subjects (4 subjects per arm)
  The planned number of subjects were determined as the maximum number possible to enroll from the perspective of feasibility.
  If a total of 8 subjects are enrolled and then 3 subjects are assigned to the pulse group and 5 subjects are assigned to the p.o. group, another subject will be enrolled for assessment. Apart from this, subjects shall be enrolled as many as possible during the enrollment period.
- Study period: August 2016 to August 2021
  （The secondary enrollment is possible until the end of June 2020.）
- However, the study period may be changed depending on the status of study progress.

Rapid progressor (Clinical trial①)

10. Treatment Plan（After Secondary Enrollment）（Rapid）

Figure 3: Treatment flow chart (Rapid progressor)

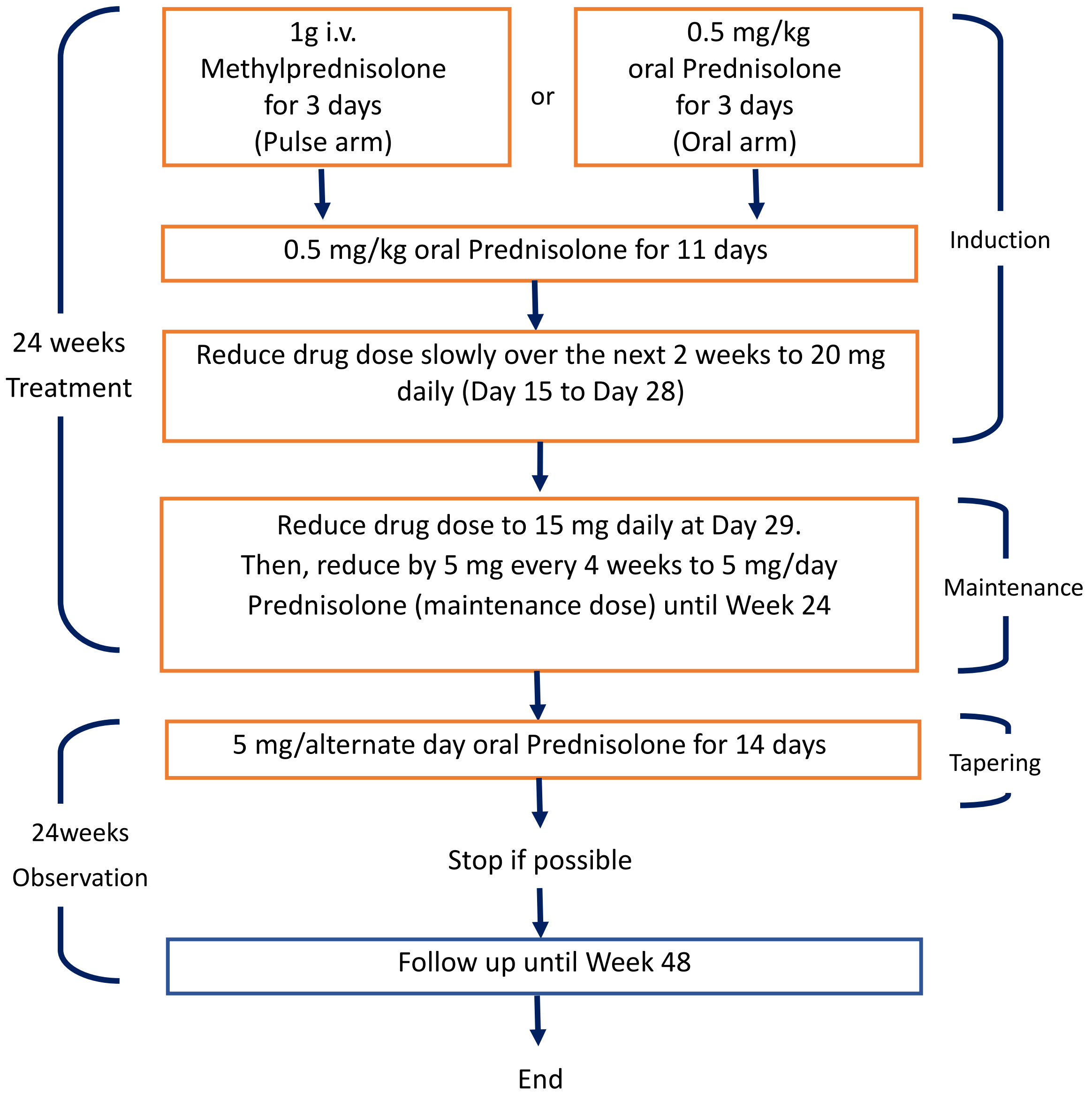

If a subject experienced worsening of symptoms of HAM due to a dose reduction or discontinuation during the study period and meets the criteria for dose increase or resumption, additional treatment shall be performed according to the procedure.

Rapid progressor (Clinical trial①)

Prior to starting the study drug treatment, it shall be confirmed that the test values on the last assessment date meet the following criteria.

⑧ Neutrophil count: ≥1,500/mm3
⑨ Platelets: ≥100,000/mm3
⑩ Hemoglobin: ≥9.0 g/dL
⑪ AST: ≤3-times the upper limit of normal (ULN)
⑫ ALT: ≤3-times the ULN
⑬ Serum creatinine: ≤1.5-times the UL
⑭ HbA1c（NGSP）: ≤6.5%

＜Explanation for Figure 3＞

Testing and treatment can be done in the outpatient or inpatient setting.

Induction

Visits：Day1～Day3 (Pulse arm only), Day15±3 days, Week 4 (Day29±7 days)

- Patients will be treated with the assigned trial drug
  The pulse arm: administer a 3-day course of i.v. methylprednisolone
  The Oral arm: administer 0.5 mg/kg oral Prednisolone for 3 days [Day1～Day3]
- Administer 0.5 mg/kg oral Prednisolone for 11 days [Day4～Day14]
- Reduce drug dose slowly over the next 2 weeks to 20mg daily [Day15～Day28]

Maintenance

Visits：Week8 (Day57±7 days), Week12 (Day85±7 days), Week24 (Day169±7 days)

- Reduce drug dose to 15 mg daily at Day 29. Then, reduce by 5 mg every 4 weeks to 5 mg/day prednisolone (maintenance dose) until Week 24 [Day29～Day169]

Dose reduction/drug discontinuation

- Subjects shall take prednisolone 5 mg/day every other day for 14 days after Day 169 (Week 24) and then discontinue the drug.
- However, subjects who have increased the maintenance dose of oral predonine by Day 169 (Week 24) shall not taper the dose or discontinue the drug.

Follow up

Visits：Week28 (Day197±7days), Week32 (Day225±7days), Week36 (Day253±7days), Week48 (Day337±7days)

- Follow up patients until Week48.

Rapid progressor (Clinical trial①)

10.1. Dose modification (dose increase/resumption)

If a subject experienced worsening of symptoms of HAM due to a reduction or discontinuation and meets the criteria for dose increase or resumption, additional treatment shall be performed according to the procedure within 7 days from the date when it is confirmed that the subject meets the criteria. Dose increase or resumption are permitted based on the values of the 10-meter timed walk and the OMDS obtained on an unscheduled study visit.

If a subject meets the dose increase/resumption criteria, the HAMLET-P Coordinating Center should be contacted before the dose increase/resumption. However, if no additional treatment is given, the clear reason should be documented.

After the dose increase, no transition to the next dose level shall be made until the end of the evaluation, regardless of the acceptable range.

Dose Increase and Resumption Criteria and Procedures

- After completion of evaluation on Day 29 to evaluation on Day 169 (Week 24)
  - ① [Dose increase criteria]

    "≥30% worsening in the 10-meter timed walk" or "≥1 grade of worsening in the OMDS" compared to baseline

    [Dose increase procedures]

    Methylprednisolone 1 g/day is intravenously administered for 3 consecutive days. Subsequently, oral prednisolone is administered at 0.5 mg/kg/day (for 11 days) and gradually reduced to 20 mg/day over 2 weeks (the method of tapering should be determined at the discretion of physician). Subsequently, the dose is reduced by the range of 2.5 mg/day (5 mg every other day) /4 weeks to 5 mg/day/4 weeks to find a maintenance dose that does not meet the dose increase criteria, and the oral administration is continued at that maintenance dose.
  - ② [Dose increase criteria]

    "≥10% worsening in the 10-meter timed walk compared to the value on Day 29 was observed on 2 visits". (Those 2 visits can be apart from each other.) If the walk test cannot be performed on Day 29 (Week 4) and the value is missing, the most recent result prior to Day 29 (Week 4) shall be used as the value on Day 29 (Week 4).

    [Dose increase procedures]

    Prednisolone is increased by 2.5 mg/day (5 mg every other day) /4 weeks to maintain at the dose that does not meet the increase criteria.
- After completion of evaluation on Day 169 (Week 24)

Rapid progressor (Clinical trial①)

① [Dose increase/resumption criteria]

"≥30% worsening in the 10-meter timed walk" or "≥1 grade of worsening in the OMDS" compared to the value on Day 169 (Week 24) was observed. If the walk test cannot be performed on Day 169 (Week 24) and the value is missing, the most recent result prior to Day 169 (Week 24) shall be used as the value on 169 (Week 24).

[Dose increase/resumption procedures]

Methylprednisolone 1 g/day is intravenously administered for 3 consecutive days. Prednisolone is administered at the dose that was administered immediately before the worsening. If the dose increase criteria are subsequently met, the dose is increased by 2.5 mg/day (5 mg every other day) /4 weeks to find a maintenance dose that does not meet the dose increase criteria, and the oral administration is continued at that maintenance dose. When reducing the dose, the minimum maintenance dose shall be 5 mg.

② [Dose increase/resumption criteria]

"≥10% worsening in the 10-meter timed walk compared to the value on Day 169 (Week 24) was observed. If the walk test cannot be performed on Day 169 (Week 24) and the value is missing, the most recent result prior to Day 169 (Week 24) shall be used as the value on 169 (Week 24).

[Dose increase/resumption procedures]

Prednisolone is administered at the dose that was administered immediately before the worsening. If the dose increase criteria are subsequently met, the dose is increased by 2.5 mg/day (5 mg every other day) /4 weeks to find a maintenance dose that does not meet the dose increase criteria, and the oral administration is continued at that maintenance dose. When reducing the dose, the minimum maintenance dose shall be 5 mg.

<u>Dose Modification Confirmation Procedures</u>

① If a subject meets the above dose increase/resumption criteria, the investigator or study collaborator shall contact the HAMLET-P Coordinating Center by email.

② The HAMLET-P Coordinating Center shall determine if the subject is eligible for additional treatment.

③ After confirming the eligibility of additional treatment, the HAMLET-P Coordination Center shall notify the investigator or study collaborator by email whether additional treatment is permitted or not.

Rapid progressor (Clinical trial①)

【HAMLET-P coordination center】
[Before September 30, 2017]
Kanagawa Institute of Industrial Science and Technology (KISTEC)
Global health research coordinating center
E-mail：hamlet-p@newkast.or.jp

[After October 1, 2017]
Clinical research data center, St. Marianna University
E-mail：mariadc_ham@marianna-u.ac.jp

10.2. Prednisolone Administration Rules

① Dose

- Do not split or crush the study drug.
- The dose should be set in 2.5 mg increments
- For the dose of 0.5 mg/kg/day, round off the fraction.

② Timing of oral administration

- Take the drug once a day.
- Oral administration after breakfast is recommended.

11. Administration Discontinuation and Study Discontinuation（Rapid）

11.1 Administration Discontinuation

The beginning and end time of administration of the study drug and whether administration is completed will be recorded in the CRF. Furthermore, if the administration is discontinued, the time of the administration discontinuation and the reasons will be recorded in the CRF.

11.2 Criteria for Study Discontinuation

If a patient meets any of the following restrictions, said patient will be discontinued from the study:

(1) If a subject develops steroid-induced glaucoma.

(2) If a subject is observed to have clear disease progression (an increase of 100% or more in 10mWT compared to baseline) by Day169 (Week 24).

(3) If a patient develops an adverse event that would make it difficult for them to continue participation in the study as determined by the principal investigator or subinvestigator.

(4) If a patient requests discontinuation.

(5) If HBV-DNA is detected in the patient.

(6) If a patient is found to be ineligible for the study

(7) When the principal investigator or subinvestigator determines that the patient should be discontinued from the study

11.3 Discontinuation Procedures

- If a patient is discontinued from the study, the study doctor will promptly notify the Coordinating center of the study discontinuation of said patient. If a patient wishes to discontinue participation in the study, the study doctor will confirm the reasons for discontinuation to the extent possible.
- The day when the patient is confirmed to meet the withdrawal criteria will be the date of discontinuation. The tests at study discontinuation will be performed on the day closest to the date of discontinuation on which tests can be performed.
- For subjects who discontinue the study due to clinical exacerbation of HAM, data will be collected on routine visit frequency for "details of post-treatment (only treatment for HAM [including drugs listed in Exclusion Criterion 1 and Exclusion Criterion 6 and treatment with HAL]), "10-meter timed walk", and "Osame's Motor Disability Score" until the period corresponding to Week 24.
- The observation of adverse events, including follow-up observation of any existing adverse events and observation of new occurrences of adverse events, will be performed up to at least 28 days after the final administration of the study drug. However, any adverse events that have not resolved at Week 48 and for which a causal relationship to the study drug cannot be ruled out will be followed-up on even after Week 48 until they are resolved or improved. However, this will not apply to any of the following cases where the study doctor determines that the follow-ups are not required:
  - If subsequent treatment is initiated and a causal relationship to the study drug is not evaluable.
  - If a patient is lost to follow-up because they have been transferred to another hospital, etc.
  - If a patient refuses to be followed-up on.
  - If a patient has died.
  - If a patient has not yet improved but their symptoms are stable, or when the principal investigator or subinvestigator determines that no further resolution can be expected.

Rapid progressor (Clinical trial①)

12. Drug Information（Rapid）

12.1 Study Drug

12.1.1 Methylprednisolone

(1) Component code

HAM-methylPSL

(2) Generic name and brand name 一般名および販売名

Generic name: Methylprednisolone sodium succinate

Brand name: Solu-Medrol 1000mg

(3) Pharmacologic Category

Synthetic corticosteroid

(4) Composition and properties

Chemical name:

11β,17,21-trihydroxy-6α-methyl-1,4-pregnadiene-3,20-dione 21-sodium succinate

Molecular formula:

C26H33NaO8

Molecular weight:

496.53

Structure:

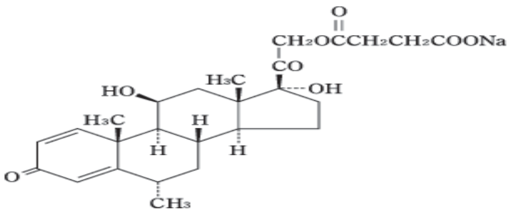

Composition:

・ Active ingredient (in 1 vial)
Methylprednisolone sodium succinate: 1326.0 mg (equivalent to 1000 mg of methylprednisolone)

・ Excipients (in 1 vial)
Anhydrous sodium monohydrogen phosphate, sodium dihydrogen phosphate monohydrate, pH modifier

Properties:

Methylprednisolone is a white mass or powder, and the solution for injection dissolved in the accompanying solution for reconstitution is clear and colorless to slightly yellow, has a pH between 7.0 and 8.0, and has an osmotic pressure ratio (to normal saline) of approximately 1.

Rapid progressor (Clinical trial①)

(5) Storage method and expiration period

Storage method: Store protected from light at room temperature

Expiration period: For 5 years from the date of manufacture

(6) Packaging form

Each box contains 5 vials

(7) Labeling

Information included in the labeling for the study drug is “for study use,” “study code,” “lot number,” “storage conditions,” and “expiration date.” For details, refer to the Standard Operating Procedures (SOPs) relating to study drug management.

(8) Precautions for use

- Reconstitution

  Reconstitute with the attached water for injection and mix with 500 mL of normal saline before use. Since methylprednisolone may generate white precipitates by changes in pH, etc., caution should be taken when mixing with infusion solution, etc.

- After reconstitution

  Use as soon as possible after reconstitution. Even if storage is required, store at 10°C or lower and use within 24 hours.

- Administration

  Because intravenous administration can cause vascular pain and phlebitis, pay sufficient attention to the preparation of the injection solution, injection site, injection method, etc. and the injection rate should be as slow as possible for the prevention.

(9) Study drug provision

The study drug will be provided to the study site following IRB approval and submission of the Clinical Trial Notification.

12.1.2 Prednisolone

(1) Component code

HAM-PSL

(2) Generic name and brand name

Generic name: Prednisolone

Brand name: Prednisolone tablet 5mg (NP)

(3) Pharmacologic category

Synthetic corticosteroid

(4) Composition and properties

Chemical name:

11β, 17, 21-Trihydroxypregna-1, 4-diene-3, 20-dione

Rapid progressor (Clinical trial①)

Molecular formula:

$C_{21}H_{28}O_5$

Molecular weight:

360.44

Structure:

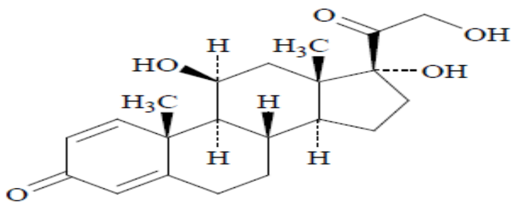

Composition:

- Active ingredient (in 1 tablet)
  Prednisolone 5 mg
- Excipients (in 1 tablet)

Lactose hydrate, corn starch, microcrystalline cellulose, talc, calcium carmellose, gelatin, calcium stearate

Form:

White scored uncoated tablet

Size:

Diameter (mm): 7.0, thickness (mm): 3.4, weight (mg): 150

(5) Storage method and expiration period

Storage method: Store at room temperature

Expiration period: For 3 years from the date of manufacture

(6) Packaging form

Press through package (PTP)

(7) Labeling

Information included in the labeling for the study drug is “for study use,” “study code,” “lot number,” “storage conditions,” and “expiration date.” For details, refer to the Standard Operating Procedures (SOPs) relating to study drug management.

(8) Study drug provision

The study drug will be provided to the study site following IRB approval and submission of the Clinical Trial Notification.

12.2 Procedures for Storage, Management, and Disposal

The investigational product accountability manager at the study site will store/manager the study drug appropriately according to the “Standard Operating Procedure for Study Drug

Management" and prepare the records on the stock, status of use, and disposal of the study drug. The investigational product accountability manager will confirm the number of unused and used study drugs (including empty vials and containers) as needed. For details on the provision, storage, management, and disposal of the study drug, refer to the "Standard Operating Procedure for Study Drug Management".

12.3 Expected Adverse Drug Reactions

12.3.1 Methylprednisolone

The following side effects have been reported with the use of methylprednisolone, so sufficient monitoring should be performed when using this drug, and appropriate measures should be taken if any side effects occur.

For details and latest information on adverse drug reactions, please refer to the latest package insert of Solu-Medrol for Intravenous Use 1000 mg.

Significant adverse reactions (frequency)

1) Shock (0.08%)
2) Cardiac arrest, circulatory shock, arrhythmia (unknown frequency)
3) Infection (2.54%)
4) Secondary adrenal insufficiency (unknown frequency)
5) Osteoporosis (unknown frequency), osteonecrosis (0.36%)
6) Gastrointestinal perforation (0.02%), hemorrhage (0.80%), peptic ulcer (0.02%)
7) Myopathy (unknown frequency)
8) Thrombosis (unknown frequency)
9) Increased intracranial pressure (unknown frequency), seizure (unknown frequency)
10) Psychiatric disturbance (0.06%), depression (0.02%)
11) Diabetes mellitus (3.95%)
12) Glaucoma (unknown frequency), Subcapsular posterior cataract (0.09%), central serous chorioretinopathy (unknown frequency), multifocal posterior pigment epitheliopathy (unknown frequency)
13) Bronchial Asthma (unknown frequency)
14) Cardiac rupture (unknown frequency)
15) Pancratitis (0.03%)
16) Congestive heart failure (0.02%)
17) Esophagitis (unknown frequency)
18) Kaposi's sarcoma (unknown frequency)
19) tendon rupture (unknown frequency)
20) Hepatic dysfunction (1.21%), jaundice (unknown frequency)

Rapid progressor (Clinical trial①)

12.3.2 Prednisolone

The following side effects have been reported, so sufficient monitoring should be performed when using this drug, and appropriate measures should be taken if any side effects occur. For details and latest information on adverse drug reactions, please refer to the latest package insert of Prednisolone tablet 5 mg NP.

Significant adverse reactions (frequency)

1) Infection (unknown frequency)
2) Secondary adrenal insufficiency, diabetes mellitus (unknown frequency)
3) Gastrointestinal perforation, hemorrhage, peptic ulcer (unknown frequency)
4) Pancratitis (unknown frequency)
5) Psychiatric disturbance, depression, seizure (unknown frequency)
6) Osteoporosis (unknown frequency), osteonecrosis, myopathy (unknown frequency
7) Glaucoma, Subcapsular posterior cataract, central serous chorioretinopathy, multifocal posterior pigment epitheliopathy (unknown frequency)
8) Thrombosis (unknown frequency)
9) Myocardial infarction, cerebral infarction, aneurysm (unknown frequency)
10) epidural lipoma (unknown frequency)
11) Tendon rupture (unknown frequency)

13. Concomitant Drugs and Therapies（Rapid）

13.1 Prohibited Concomitant Drugs

Until the completion of the observation period (Week 48), the concomitant use of the following drugs considered to have an effect on the evaluation of the study drug will be prohibited:

① Therapeutic drugs for the treatment of HAM/TSP other than the study drug
Concomitant use will be prohibited during the study period.

② Immuno-suppresive drugs and corticosteroids (systemic administration)
Concomitant use will be prohibited during the study period.

③ Medications for gait symptoms associated with HAM (restricted concomitant use)
Concomitant use of medications to relieve symptoms of HAM (muscle relaxants, Botox, etc.)

- Is prohibited throughout the study if not used within 2 weeks prior to the date of

informed consent.

- If they have been used within 2 weeks prior to the date of informed consent, it may be used concomitantly even after participation in the study at the stable dose without change from the time of participation throughout the study.

Details can be found in 13.3.1. Concomitant Medications.

④ Pentosan polysulfate, vitamins and supplements

- Pentosan polysulfate (prohibited concomitant use)
  Concomitant use is prohibited throughout the study.
- Prosultiamine (prohibited concomitant use)
  Concomitant use is prohibited throughout the study.
- Ascorbic acid (restricted concomitant use)
  - Use of 1.5 g/day or more is prohibited throughout the study.
  - Concomitant use is prohibited throughout the study if not used prior to the primary enrollment.
  - If it has been used since before the primary enrollment, it may be used concomitantly after the primary enrollment at the stable dose without change from the time of primary enrollment throughout the study.
- Vitamins and supplements used for HAM other than those listed above (restricted concomitant use)
  - Concomitant use is prohibited throughout the study if not used prior to the primary enrollment.
  - If it has been used since before the primary enrollment, it may be used concomitantly after the primary enrollment at the stable dose without change from the time of primary enrollment throughout the study.

⑤ Live or attenuated vaccinations

Concomitant use will be prohibited during the study period.

⑥ Hypnotics (restricted concomitant use)

The use of intermediate and long-acting drugs is prohibited throughout the study.

The use of the ultra-short-acting form is prohibited within 4 hours before the efficacy evaluation, and the short-acting form is prohibited within 10 hours before the efficacy evaluation.

⑦ Strong CYP3A4 inducers and inhibitors (prohibited concomitant use)

Concomitant use is prohibited throughout the study.

⑧ Other study drugs

Concomitant use will be prohibited during the study period.

⑨ Desmopressin Acetate Hydrate

Concomitant use will be prohibited during the study period.

13.2Drugs requiring caution for concomitant use

Review the latest package insert.

13.3Allowed Concomitant Drugs and Therapies

13.3.1 Allowed Concomitant Drugs

The concomitant use of drugs is allowed in the following cases.
Note that the name, administration and dosage, route of administration, administration period, and the reasons for the administration of the drugs concomitantly used from the start date of administration of the study drug to the completion of the observation period will be recorded in the CRF

① When drugs other than prohibited concomitant drugs for the treatment of symptoms accompanying complications or the primary disease have been used since before participation in the study, the continuous use of these drugs will be allowed after the start of the study

② Drugs other than prohibited concomitant drugs can be used for symptomatic treatment of adverse events that occur after the start of administration of the study drug.

③ The prophylactic use of the drugs for adverse events that have already been observed once will be allowed.

＜Recommended concomitant drugs＞
It is recommended to co-medicate participants with the following medications to prevent adverse reactions associated with corticosteroids:

1） Antiacids (H2-blocker, etc.)
2） Osteoporosis prevention (Bisphosphonate, Vitamin D2, etc.)

＜Other Allowed Concomitant Drugs＞
Recommended symptomatic treatment, prophylactic use, and therapy for complications are shown below (as references).

1) Hyperglycemia
As glucose metabolism abnormalities may occur and cause high blood sugar levels due to administration of this study drug, if such signs are observed, appropriate measures such as the use of blood sugar control drugs should be promptly taken.
2) Infectious disease

As administering this study drug may decrease immune function and increase susceptibility to infection, if any infection or signs of infection are observed, use antibiotics and take appropriate measures promptly.

3) Insomnia

As administration of the study drug may cause insomnia, if such signs are observed, appropriate measures such as the use of sleep medications should be taken promptly.

4) Muscle tightness (spasms)

If antispasticity drugs (tizanidine hydrochloride, eperisone hydrochloride, baclofen, etc.) are being orally taken for muscle tightness (spasms) caused by HAM at the time of primary enrollment, the continuous use of those drugs are allowed.

However, dose modifications and new start of oral administration during the study period are not permitted.

5) Decrease in voiding function

For decreased urinary function (neuropathic bladder, overactive bladder) caused by HAM disease, the medications used for the symptoms at the time of primary enrollment (e.g., propiverin hydrochloride, sorifenacin succinate, imidafenacin, prazocin hydrochloride, and distigmine bromide) may be used continuously even after the primary enrollment.

However, any change in oral dosage or start of new oral administration during the study period is not permitted.

6) Neuropathic pain

For neuropathic pain caused by HAM disease, the medications used for the symptoms at the time of primary enrollment (pregabalin, duloxetine hydrochloride, amitriptyline hydrochloride, clonazepam, etc.) may be used continuously even after primary enrollment.

However, any change in oral dosage or start of new oral administration during the study period is not permitted.

7) Skin disease and localized allergy symptoms such as pollinosis

For skin disease (eczema, etc.) and allergy symptoms such as pollinosis, the use of topical corticosteroids/corticosteroid nasal sprays/corticosteroid eye drops is allowed. If local steroid therapy is being performed at the time of primary enrollment and it will also be performed after the primary enrollment, the same drugs should be used before and after the primary enrollment.

For skin disease, the use of topical corticosteroids is allowed as symptomatic treatment of adverse events that occur after the start of administration of the study drug.

Rapid progressor (Clinical trial①)

13.3.2 Allowed Concomitant Therapies

If concomitant therapies are performed, the name or details of the therapies performed, period of the therapies, and the reasons for the therapies will be recorded in the CRF.

1） Physical therapy

If physical therapy is performed for functional impairment caused by HAM prior to the primary enrollment, its continuation is allowed during the study period. However, changing of the content or frequency of the therapy and starting of new therapy is not allowed during the study period.

2) Self-catheterization

If a subject has undergone self-catheterization prior to the primary enrollment instance for functional impairment due to HAM disease, it is acceptable to continue during the study. However, in principle, changing of the frequency and starting of new procedure is not allowed during the study period.

13.4Subsequent Treatment after Study Completion/Discontinuation

There are no specific regulations on subsequent treatment after the completion/discontinuation of the study.

14. Observation, Investigations, Test Items, and Implementation Period （Rapid）

14.1Observation Schedule

14.1.1 Screening

When the start of study drug administration is defined as Day 1, the screening tests will be performed between Weeks -16 and -12. Screening tests (Weeks -16 to -12) and tests in the progressor assessment period on Week -12 may be performed on the same day.

14.1.2 Progressor assessment period

When the start of study drug administration is defined as Day 1, the progressor assessment period will be 12 weeks from Week -12 to the last assessment date. In addition, if the rapid progressor criteria are met, the secondary enrollment and treatment can be started at any time during the progressor assessment period. In that case, if the period between the visits corresponding to Week -12 to the start of treatment is less than 4 weeks, it is not necessary to perform the tests after the visits meeting the rapid progressor criteria to before the secondary enrollment.

Rapid progressor (Clinical trial①)

14.1.3 Sudy drug treatment period

When the start of study drug administration is defined as Day 1, the study drug treatment period will be 24 weeks from Day 1 to the day before study visit on Week 24.

14.1.4 Observation period

When the start of study drug administration is defined as Day 1, this will be 24 weeks from Week 24 to Week 48. Other than when the study is discontinued due to the subject's request, follow-up will be performed after the study discontinuation date until Week 48 unless the subject is lost to follow-up.

14.1.5 Post-observation period

The post-observation period will be 28 days from the last administration of the study drug.

14.2 Patient demographic information

Date consent was obtained for participation in the study, subject identification number, sex, date of birth, height, body weight, ethnicity, Clinical history of HAM/TSP (date of diagnosis, date of onset, OMDS), history of previous treatments, presence/absence of complications (the name of the disease(s) if complications are present, presence/absence of treatment at the start of the study), presence/absence of past medical history (the name of the disease(s) if a patient has a past medical history), familial history of HAM/TSP and ATL, blood transfusion history.

14.3 Observation, Investigations, and Test Items

Investigations of patients will be performed according to the parameters and schedule shown in Section 14.4 Study Schedule.

14.3.1 Clinical Assessment

(1) Physical examination

In physical examination, information will be collected on any symptoms and findings that are considered clinically meaningful.

The information shall be handled as "past medical history/complications" prior to administration of the study drug and "adverse events" after administration of the study drug.

(2) Blood Pressure, Pulse Rate, Body Temperature

Blood pressure and pulse rate will be measured at rest in the sitting position. Axillary temperature will be used for body temperature.

Rapid progressor (Clinical trial①)

(3) Height, Body Weight

(4) Overall Evaluation (Using a VAS)

The person in charge of assessment of the clinical efficacy endpoints will perform the evaluation.

<Implementation methods>

Since HAM/TSP not only causes gait disturbance and urination disorder but also has impact on daily life due to its various symptoms, the patient will be instructed to comprehensively express the level of its impact using a VAS as the evaluation method. The patient is instructed to look at a black line with a length of 10 cm (the left end with "condition with no impact of HAM/TSP and no impact on daily life" and the right end with "worst impact of HAM/TSP ever") and point at the current level of their overall condition. Give the VAS questionnaire (Appendix 2) to the subject to complete.

(5) Evaluation of Ambulatory Status (Using a VAS)

The person in charge of assessment of the clinical efficacy endpoints will perform the evaluation.

<Implementation methods>

A black line with a 10cm length (the right end indicating "no effect on walking by HAM at all" and the left end indicating "worst impact on walking by HAM so far) will be presented to a subject and the subject will indicate the extent of current ambulatory status. Give the VAS questionnaire (Appendix 2) to the subject to complete.

(6) Evaluation of Lower Extremity Pain (Using a VAS)

The person in charge of assessment of the clinical efficacy endpoints will perform the evaluation.

＜Implementation methods＞

The patient is instructed to look at a black line with a length of 10 cm (the left end with "no pain" and the right end with "worst pain ever") and point at their current level of pain (Appendix2).

(7) Ambulatory aid used at the clinic (= outdoors during a clinic visit)

The person in charge of assessment of the clinical efficacy endpoints will perform the evaluation.

＜Implementation methods＞

Rapid progressor (Clinical trial①)

Type of walking aid used at the site visit and any changes from the previous visit will be collected (Appendix1).

(8) Ambulatory aid used at home (= indoors)

The person in charge of assessment of the clinical efficacy endpoints will perform the evaluation.

＜Implementation methods＞

Type of walking aid used at home and any changes from the previous visit will be collected (Appendix1).

(9) 10 meter Walk Test (Seconds)

The person in charge of assessment of the clinical efficacy endpoints will perform the evaluation in accordance with the “Standard Operating Procedure for Performance of Walk Test”.

<Implementation methods>

Place markings with tape, etc. at the start and finish points of a straight 10-meter long line on the floor. The time required to walk the length of the 10-meter course will be measured using a stopwatch.

Measurements must be taken twice and both measurements will be recorded. Data will be assessed using the mean values (rounded to two decimal places). If 1 of the 2 measurements is missing, the 1 measurement value will be used as is for evaluation.

(10) 6 minute Walk Distance (meters)

The person in charge of assessment of the clinical efficacy endpoints will perform the evaluation in accordance with the “Standard Operating Procedure for Performance of Walk Test”.

<Implementation methods>

Place markings with tape, etc. at the start and finish points of a straight 10 meter long line on the floor. Have the patient repeatedly walk from the start point to the finish point and back and measure the distance the patient is able to walk during a 6 minute period. If a 6 minute walk is difficult for a patient, the actual distance walked and the time taken will be recorded.

(11) 2 minute Walk Distance (meters)

Rapid progressor (Clinical trial①)

The person in charge of assessment of the clinical efficacy endpoints will perform the evaluation in accordance with the “Standard Operating Procedure for Performance of Walk Test”.

<Implementation methods>

When the 6 minute walk distance test is performed, the distance the subject walked in 2 minutes after the start of the walk will be simultaneously measured and record as data for the 2 minute walk distance.

If a 2 minute walk is difficult for a patient, the actual distance walked and the time taken will be recorded.

(12) Timed Up and Go Test (seconds)

The person in charge of assessment of the clinical efficacy endpoints will perform the evaluation in accordance with the “Standard Operating Procedure for Performance of Walk Test”.

<Implementation methods>

Place markings with tape, etc. at the start and finish points of a straight 3 meter long line on the floor. Have the patient walk from the start point to the finish point and back and measure the time required for the walk with a stopwatch. The patient will be instructed to perform these series of movements at both “a normal walking speed” and “a maximum walking speed” (a total of 2 sets of movements) according to directions from the person performing the measurements. After measurements are taken twice, the smaller measurement (faster time) will be used and recorded in seconds to one decimal place. (Round numbers to one decimal place)

(13) Osame Motor Disability Score

The person in charge of assessment of the clinical efficacy endpoints will perform the evaluation.

<Implementation methods>

The patient’s condition will be determined according to the table and scored (Appendix 1).

(14) IPEC 1

The person in charge of assessment of the clinical efficacy endpoints will perform the evaluation.

<Implementation methods＞

The patient’s condition will be determined according to the table and scored (Appendix 1).

(15) Modified IPEC 2

The person in charge of assessment of the clinical efficacy endpoints will perform the evaluation.

<Implementation methods＞

Patients wil fill in the Questionnaire sheet (Appendix 1).

(16) Modified Ashworth scale (MAS)

The person in charge of assessment of the clinical efficacy endpoints will perform the evaluation.

<Implementation methods＞

The patient’s condition will be determined according to the table and scored (Appendix 1).

(17) OABSS

The person in charge of assessment of the clinical efficacy endpoints will perform the evaluation.

<Implementation methods＞

The patient will undergo an interview investigation (Appendix 1).

(18) ICIQ-SF

The person in charge of assessment of the clinical efficacy endpoints will perform the evaluation.

<Implementation methods＞

The patient will undergo an interview investigation (Appendix 1).

(19) N-QOL

The person in charge of assessment of the clinical efficacy endpoints will perform the evaluation.

<Implementation methods＞

Patients wil fill in the Questionnaire sheet (Appendix 1).

(20) IPSS

The person in charge of assessment of the clinical efficacy endpoints will perform the

evaluation.

<Implementation methods>

The patient will undergo an interview investigation (Appendix 1).

(21) **Sexual Health Inventory for Men**

The person in charge of assessment of the clinical efficacy endpoints will perform the evaluation.

<Implementation methods>

Patients wil fill in the Questionnaire sheet (Appendix 1).

14.3.2 Blood tests

Items with the term ‘Accompanying Research Items’ will be performed as an accompanying research to understand the duration of efficacy of methylprednisolone and prednisolone treatment, the influences and effects on the subject’s immune system, and prognostic and progressive disease categorical factors of clinical progression. Consent for participation in the accompanying research will be separately obtained from the subject.

(1) Safety

＜Test parameters＞

① Complete blood count with differential
② Urea nitrogen and electrolytes (BUN, Cre, Na, K)
③ Liver function (AST, ALT, ALP, T-Bil)
④ Glucose metabolism (HbA1c (NGSP), Fasting blood glucose)
⑤ Bone metablism (Ca, P)
⑥ Lipids (T-Cho, LDL-C, HDL-C, TG)
⑦ Hepatitis B virus tests (HBc antibody, HBs antibody, HBs antigen, HBV-DNA)
⑧ HCV antibody
⑨ HIV-1 antibody, HIV-2 antibody

＜Institutions peforming measurements＞

The tests shall be performed at the Clinical Laboratory of each study site. Qualitative HBV-DNA shall be performed according to local laboratory procedures.

(2) Research

＜Test parameters＞

① β2-microglobrin (Accompaning research)

② sVCAM-1 (Accompaning research)
③ SPARC (Accompaning research)
④ sIL-2R (Accompaning research)
⑤ HTLV-1 proviral load (Accompaning research)
⑥ B- and T-cell subsets/markers (Accompaning research)
⑦ HTLV-1 antibody for HAM/TSP diagnosis

＜Implementation methods＞

- Test parameters ①②③④⑤⑥:
  The tests shall be perform by central measurement. The procedure for processing and transporting samples is described in the “Standard Operating Procedure for Sample Processing for Central Measurement”.
- Test parameter ⑦:
  The tests shall be performed according to local laboratory procedures.

＜Institutions peforming measurements＞

④: SRL, Inc.
①,②,③,⑤,⑥: The Department of Rare Diseases Research, Institute of Medical Sciences, St. Marianna University School of Medicine.
⑦: The tests shall be performed according to local laboratory procedures.

14.3.3 Urinalysis

＜Test parameters＞
① Urine glucose
② Urine protein
③ Pregnancy test

＜Institutions peforming measurements＞

The tests shall be performed at the Clinical Laboratory of each study site.

14.3.4 Cerebrospinal Fluid tests

(1) General tests **1**

＜Test parameters＞
① Cell counts/ Cell fractionation
② Total protein
③ Glucose

＜Implementation methods＞

Fifteen drops of cerebrospinal fluid will be collected in Spitz tubes.

＜Institutions peforming measurements＞

The tests shall be performed at the Clinical Laboratory of each study site.

(2) Genaral test 2

＜Test parameters＞

Neopterin concentration

＜Implementation methods＞

Three mL of cerebrospinal fluid will be collected in Spitz tubes

The procedure for processing and transporting samples is described in the "Standard Operating Procedure for Sample Processing for Central Measurement".

＜Institutions peforming measurements＞

SRL, Inc.

(3) Research

＜Test parameters＞

① HTLV-1 antibody (for HAM diagnosis (at screening only) and Accompaning research)
② HTLV-1 proviral load (Accompaning research)
③ CXCL10 (Accompaning research)
④ sVCAM-1 (Accompaning research)

＜Implementation methods＞

The tests shall be perform by central measurement. The procedure for processing and transporting samples is described in the "Standard Operating Procedure for Sample Processing for Central Measurement".

＜Institutions peforming measurements＞

①：SRL, Inc

②,③,④：The Department of Rare Diseases Research, Institute of Medical Sciences, St. Marianna University School of Medicine

However, samples for the test item ② HTLV-1 proviral load will be stored in the form of DNA and, in principle, measured at the global research institute (Imperial College London, UK).

Test item ③ CXCL10 will be measured using CXCL10 measurement kits (Cosmic Corporation Co., Ltd., Becton Dickinson &Company).

Rapid progressor (Clinical trial①)

14.3.5 MRI

<Test site>

The brain (T1, T2) and the cervical to upper thoracic spinal cord (T1, T2) shall be measured.

<Institution performing measurements>

The tests shall be performed at each site. However, the tests to be performed before the secondary enrollment is not required at each study site if data performed at another hospital can be obtained.

<Institution performing evaluations>

As a general rule, MRI image assessments will be performed on all subjects enrolled at the primary enrollment at an international collaborative research organization (NIH, U.S.).

14.3.6 Intraocular pressure

＜Implementation methods＞

Follow each site’s measurement method. Throughout the study period, the method of measurement should be unified for the same subject.

Rapid progressor (Clinical trial①)

14.4Schedule Table

X = mandatory, (x) = optional

Period		Screening *1	1st Enrollment *3	Progressor assessment*2				2nd Enrollment*4	Study drug treatment period								Observation period				Unplanned visits*25	Post-observation*6	At discontinuation*7
Week		-16 ～ - 12*3		-12 *3	-8	-4	Last assessment *4 ±7		0			2	4	8	12	24 *5	28	32	36	48			
Day		-112～-84 ±7		-84 ±7	-56 ±7	-28 ±7			1 *4	2	3	15 ±3	29 ±7	57 ±7	85 ±7	169 ±7	197 ±7	225 ±7	253 ±7	337 ±7			
Informed consent		x																					
Eligibility confirmation		x																					
Study drug treatment									→	→	→	→	→	→	→	→							
Clinical Assessment	Physical examination	x		x*2	x*2	x*2	x*2					x	x	x	x	x	x	x	x	x		x	x
	Blood pressure, Pulse rate, Body temperature	x		x*2	x*2	x*2	x*2					x	x	x	x	x	x	x	x	x			x*8
	Height, Body weight	x														x*9				x*9			
	OMDS ☆	x		x*2	x*2	x*2	x*2					x	x	x	x	x	x	x	x	x	x		x
	Walking tests *A ☆	x		x*2	x*2	x*2	x*2					x	x	x	x	x	x	x	x	x	x		x*10
	Walking aids *B ☆	x		x*2	x*2	x*2	x*2					x	x	x	x	x	x	x	x	x	x		
	MAS ☆	x		x*2	x*2	x*2	x*2					x	x	x	x	x	x	x	x	x			
	IPEC1 ☆	x		x*2	x*2	x*2	x*2					x	x	x	x	x	x	x	x	x			
	VAS *C ☆			x*2	x*2	x*2	x*2					x	x	x	x	x	x	x	x	x			
	QOL *D ☆	x		x*2	x*2	x*2	x*2					x	x	x	x	x	x	x	x	x			

Rapid progressor (Clinical trial①)

Period		Screening *1	1st Enrollment *3	Progressor assessment *2				2nd Enrollment *4	Study drug treatment period								Observation period				Unplanned visits *25	Post-observation *6	At discontinuation *7
Week		-16 ～ - 12*3		-12 *3	-8	-4	Last assessment *4		0			2	4	8	12	24 *5	28	32	36	48			
Day		-112～-84 ±7		-84 ±7	-56 ±7	-28 ±7	±7		1 *4	2	3	15 ±3	29 ±7	57 ±7	85 ±7	169 ±7	197 ±7	225 ±7	253 ±7	337 ±7			
	Urinary dysfunction *E ☆	x		x*2	x*2	x*2	x*2					x	x	x	x	x	x	x	x	x			
Blood tests*11*F/ Urinalysis*G		x		x*2	x*2	x*2	x*2				x*12	x	x	x	x	x	x		x	x			x
Virus tests *18*H		x*13											x*14	x*14	x*14	x*14	x *15		x *15	x *15			x *16
Pregnancy test*17		x					(x) *2																
Cerebrospinal fluid tests*18 *I		x*19					x*2					x				x	(x) *20	(x) *20	(x) *20	x			
Accompanying research	Blood tests *J	x		x*2	x*2	x*2	x*2					x	x	(x)	x	x	x		x	x			
Accompanying research	Biobank samples (Blood) *21	x		x*2	x*2	x*2	x*2					x	x	(x)	x	x	x		x	x			
Accompanying research	Cerebrospinal Fluid tests *K	x*19					x*2					x				x	(x) *20	(x) *20	(x) *20	x			
Accompanying research	Biobank samples (Cerebrospinal fluid) *21	x*19					x*2					x				x	(x) *20	(x) *20	(x) *20	x			
MRI		x*22		x*22									(x)			x				(x)			
Intraocular pressure*23		x*22		x*22									x	x*24	x*24	x*24			x*24	x*24			

Rapid progressor (Clinical trial①)

☆: The person in charge of assessment of the clinical efficacy endpoints will perform the evaluation

*1: Subjects who meet the rapid progressor criteria at screening will not need to undergo the progressor assessment period and may enter the primary and secondary enrollment and start the study drug treatment within 7 days of screening. In this case, the values at the screening will be the baseline value.

*2: The progressor assessment period will be 12 weeks. Subjects who meet the rapid progressor criteria during the progressor assessment period may enter the secondary enrollment and start the study drug treatment after all required tests and assessments have been performed on the last assessment date. The values at the screening will be used as the baseline values and the progressor assessment period is not required only if the period between screening and the start of the study drug treatment is less than 4 weeks. If the time between screening and initiation of study treatment exceeds 4 weeks, the last assessment date must be performed.

*3: The progressor assessment period on Week -12 and screening can be performed on the same day. In this case, the items specified in the screening should be performed. In addition, the primary enrollment should occur within 7 days of screening.

*4: Secondary enrollment should be performed within 7 days of the last assessment date to start Day 1 (study drug treatment). The date may be the same as the date of the final assessment.

*5: For subjects who discontinue the study due to clinical exacerbation of HAM, data will be collected on “details of the post-treatment regimen (for treatment of HAM [including the drugs listed in Exclusion Criterion 1 and Exclusion Criterion 6 and treatment with HAL], “10-meter timed walk”, and “Osame’s Motor Disability Score” until the period corresponding to Week 24 in the study treatment period.

*6: To be performed 28 days (+28 days) after the final dose of the study drug. Follow-up is not required if 28 days have passed since the final dose of the study drug at Week 48.

*7: To be performed within 28 days after discontinuation of the study drug during the treatment period or before the start of post-treatment, whichever is earlier.

*8: Body temperature will be measured as needed.

*9: Height measurement is not required.

*10: 10-meter timed walk, 6-minute walk distance, and 2-minute walk distance will be performed. Timed up-and-go test is not required.

*11: Fasting glucose will be measured on Day 3 of rechallenge in all subjects who are re-treated with methylprednisolone.

*12: Fasting blood glucose only will be measured for subjects in the pulse group.

*13: HBs antigen, HBs antibody, HBc antibody, HCV antibody, HIV-1 antibody and HIV-2 antibody will be tested only at screening. If either HBs antibody or HBc antibody is positive, quantitative HBV-DNA measurement will be performed.

However, quantitative HBV-DNA is not required if the positive HBs antibody is clearly due to vaccination.

*14: Quantitative HBV-DNA measurement will be performed only in subjects who are positive for HBs or HBc antibodies (with the exception of positive HBs antibody clearly due to vaccination) at screening and whose quantitative HBV-DNA is below the detection sensitivity.

Rapid progressor (Clinical trial①)

*15: Subjects who are positive for HBs or HBc antibodies (with the exception of positive HBs antibody clearly due to vaccination) at screening and whose quantitative HBV-DNA is below the detection sensitivity will be closely monitored for liver function tests, and if abnormal values are observed (if AST and ALT increase is ≥ 5 times the reference value at the site), quantitative HBV-DNA measurement will be performed.

*16: For subjects who are positive for HBs or HBc antibodies (with the exception of positive HBs antibody clearly due to vaccination) at screening and whose quantitative HBV-DNA is below the detection sensitivity, if the liver function test shows abnormal values (if AST and ALT increase is ≥ 5 times the reference value at the site), quantitative HBV-DNA measurement will be performed.

*17: It is not required for subjects who are permanently sterilized. (Permanent sterilization: postmenopausal [with no menses for at least 48 weeks with no other medical reason], post sterilization [hysterectomy, bilateral salpingectomy, bilateral oophorectomy], tubal occlusion without tubal ligation)

*18: If data is available from a previous test on anti-HTLV-1 antibody for diagnosis of HAM, that data may be used.

*19: Data obtained within 12 weeks prior to the date of consent may be used.

*20: When restarting prednisolone treatment due to exacerbation after completion of the study drug treatment, it is recommended to perform a cerebrospinal fluid test before restart.

*21: Residual samples will be used as samples for biobanking.

*22: MRI and intraocular pressure measurement will be performed at any visit from screening to the progressor assessment period and the data will be used as the baseline value. For MRI scans, data obtained within 12 weeks prior to the date of informed consent can be used.

However, if an image used as the baseline shows findings suggestive of inflammation in the spinal cord, a retest must be performed within 12 weeks prior to the secondary enrollment for that site only. In addition, if data obtained within 12 weeks prior to the date of informed consent at another hospital is available and indicates no findings of inflammation in the spinal cord, a retest is not required.

*23: Intraocular pressure will be measured at the visit corresponding to 4 weeks (± 7 days) after rechallenge with methylprednisolone in all subjects.

*24: To be performed only in subjects with glaucoma.

*25: Unscheduled visit is allowed to confirm the extent of disease progression. At the unscheduled visit, only the Osame's Motor Disability Score, the walk test (10-meter timed walk), and the use of walking aids will be performed.

*A：Waling tests (10 meter Timed Walk, 6 minute Walk Distance, 2 minute Walk Distance, Timed Up and Go Test)

*B：Ambulatory aid used at the clinic and home

*C：VAS（Overall, Walking, Lower extremity pain）

*D：QOL (Modified IPEC2, N-QOL, Sexual Health Inventory for Men）

*E：Urinary dysfunction (OABSS, ICIQ-SF, IPSS)

Rapid progressor (Clinical trial①)

*F：Blood tests： Complete blood count with differential, Urea nitrogen and electrolytes (BUN, Cre, Na, K), Liver function (AST, ALT, ALP, T-Bil),
Glucose metabolism (HbA1c (NGSP), Fasting blood glucose), Bone metablism (Ca, P), Lipids (T-Cho, LDL-C, HDL-C, TG)

*G：Urinalysis: glucose, protein

*H：Virus tests: Hepatitis B virus (HBc antibody ((at screening only), HBs antibody (at screening only), HBs antigen (at screening only), HBV-DNA)
HCV antibody (at screening only), HIV-1 antibody･HIV-2 antibody (at screening only)
Anti-HTLV-1 antibody for HAM diagnosis (at screening only)

*I：Cerebrospinal fluid tests: Anti-HTLV-1 antibody for HAM diagnosis (at screening only)
Safety: Glucose
Efficacy: Cell counts/ Cell fractionation, Toral protein, neopterin

*J：Blood tests (Accompanying research): β2-microgloblin, B- and T-cell subsets/markers、sIL-2R、HTLV-1 proviral load, sVCAM-1、SPARC

*K：Cerebrospinal fluid tests (Accompanying research): HTLV-1 anribody, HTLV-1 proviral load, CXCL10, sVCAM-1

Rapid progressor (Clinical trial①)

15. Management of Patients（Rapid）

15.1 Notification to Other Departments/Hospitals

The principal investigator or subinvestigator will confirm whether the patient will visit other departments or hospitals before the start of administration of the study drug. If the patient has already visited another department or hospital, the principal investigator or subinvestigator will notify the patient's primary care physician of their participation in the study. If the patient uses any drugs other than those prescribed by the principal investigator or subinvestigator, the name of the drug(s) and the status of usage will be investigated.
If the patient newly visits another department or hospital during the study, the principal investigator or subinvestigator will take the same action as above.

15.2 Notification to Patients

The principal investigator, subinvestigator, or study collaborator will give the following instructions to patients:
＜Visits＞

For the pulse arm, study visits should be performed on an inpatient or outpatient basis from the day of methylprednisolone administration until Day 3. In principle, the progressor assessment period, the treatment period of study drug other than methylprednisolone, and the observation period will be performed on an outpatient basis. During visits, subjects will undergo the prescribed tests in accordance with the explanations and instructions of the Principal Investigator, etc.

For any abnormality, the principal investigator, etc. must be contacted and the subject must visit the hospital at any time.

16. Evaluation Criteria/Response Evaluation（Rapid）

16.1 Safety Evaluation

<u>Adverse events (frequency and severity)</u>

The worst grade of each adverse event, as well as the number and proportion of patients who develop the event will be obtained by setting the number of patients, regardless of their eligibility/ineligibility, who receive any part of the treatment specified in the protocol (all subjects who receive treatment) as the denominator using the CTCAE v4.0 Japanese

translation JCOG/JSCO version.

16.2 Efficacy Evaluation

Refer to the primary and secondary outcome measures.

17. Data Reporting Method（Rapid）

17.1 Target for Preparation of Case Report Forms

Case report forms shall be created for all enrolled subjects.

17.2 Preparation and Storage of Case Report Forms

In this study, case report forms shall be prepared and submitted in the EDC system called eCB.
After the completion of specified tests/observations, the principal investigator shall promptly enter data and sign on the eCB. If the subinvestigator or study collaborator completes data entry, the principal investigator shall review and check the details and then sign the form. For the method of input and correction, follow the guide which will be separately prepared.
If the study collaborator enters data into the case report form, only the extent that the data can be transcribed from the source documents is acceptable. The subinvestigators and study collaborators who can fill in the case report forms must be registered in the "List of subinvestigators and study collaborators".
After data lock, the principal investigator shall obtain and store the CD-R containing the input data (including the correction history) from the data center.

17.3 Identification of Source Documents

The source documents in this study are as follows:

- Medical records
- Diagnostic imaging films
- List of names for screening
- Informed consent forms
- Study drug accountability log
- Output form for general laboratory tests results
- QOL questionnaire, VAS questionnaire, and questionnaire for assessment of dysuria
- Clinical Efficacy Endpoint Assessment Form

Rapid progressor (Clinical trial①)

The following items will be directly entered onto a CRF, which can be handled as a source document. However, items that are accompanied by medical judgments must be directly entered onto a CRF by the principal investigator or subinvestigator.

1) The following items related to adverse events:
   - Corresponding CTCAE term
   - Seriousness
   - Causal relationship to the study drug and comments on causal relationship
2) Reasons for the performing of concomitant treatment
3) Reasons for determining why an adverse event did not require follow-up

17.4Procedures for Handling Missing, Unused, and Abnormal Data

For missing, unused, and abnormal data not expected before the start of the study, the principal investigator and the statistician will discuss and decide how to handle the data. Data handling details will be described in the analysis reports.

18. Adverse Event Reporting（Rapid）

18.1 Definition of Adverse Event

An adverse event is any unfavorable or unintended sign (including abnormal variations in laboratory test findings), symptom, or disease that occurs after the start of treatment, regardless of relationship with the study drug. It also includes an exacerbation of a pre-existing condition during the study.

In this study, when a pre-existing condition worsens by one level or more from the grade originally observed before the start of the initial administration of the study drug according to CTCAE v4.0 criteria, it is considered an adverse event. For laboratory test parameters not specified in the protocol, refer to Section 18.2. A progression of the disease (HAM/TSP) is not handled as an adverse event. However, when a condition cannot be clearly determined, it should be handled as an adverse event.

18.2 Abnormal Values for Laboratory Tests Whose Measurements are not specified in the Protocol

Any abnormal laboratory test values that meet the following conditions are handles as an adverse event:

Rapid progressor (Clinical trial①)

1) If it meets the definition of serious adverse event

2) If it is related to any changes in the study drug (changes in dose, interruption or discontinuation of the study drug) or when it is related to any changes in concomitant treatment (addition, change, or discontinuation of concomitant treatment)

3) If it is accompanied by clinical symptoms

4) If it is determined be clinically problematic

18.3Serious Adverse Events

An adverse event that meets any of the following conditions is determined to be serious:

(1) Death

- Death during the study, regardless of whether there is causal relationship to the study.
- Deaths that occur after the study for which a causal relationship to the study cannot be ruled out. Deaths suspected to be related to treatment for the study are included. Deaths clearly due to primary disease are not included.

(2) Life-threatening

- Grade 4 non-hematotoxicity
- Grade4 hematotoxicity (excluding Leukocytosis)

(3) Adverse events that require hospitalization or prolonging of existing hospitalization. However, hospitalization for the following purposes is not handled as a serious adverse event:

- Hospitalization scheduled before the start of the study
- Hospitalization previously planned, such as for the purpose of reducing the burden on patients, due to visiting the hospital from far locations

(4) Results in persistent or significant disability/incapacity

(5) Congenital anomaly/birth defect in later generations

(6) Other medically important conditions

This refers to conditions that are not included in (2) to (5) above, but are considered to be medically important.

18.4Causal Relationship to the Study Drug

The principal investigator or subinvestigator will determine the causal relationship to the study drug by classifying it into the following five types:

- Definite: Definitely due to study treatment
- Probable: Probably due to study treatment
- Possible: Possibly due to study treatment

- Unlike: Unlikely due to study treatment
- Not related: Not related to study treatment

18.5 Determination of the Severity of an Adverse Event

The severity (Grade) of an adverse event will be determined using the CTCAE v4.0 Japanese translation JCOG version and the Grade for the event which is closest to the definition of the event term will be allocated

18.6 Classification of Intervention against an Adverse Event

Interventions against an adverse event will be selected from and classified into the following six types (selection of multiple types of intervention except for type 0 is possible):

0: No intervention performed
1: Decrease in dose or interruption of the study drug
2: Discontinuation of the study
3: Treatment with any drugs
4: Treatment using methods other than drugs
5: Hospitalization or prolonging of existing hospitalization
6: Other

18.7 Reporting Procedures if a Serious Adverse Event Occurs

18.7.1 Reporting Obligations of the Principal Investigator and Subinvestigator and Reporting Procedures

If a serious adverse event occurs, the study doctor will promptly perform appropriate intervention and also immediately report it to the principal investigator. Upon request of the principal investigator, the study doctor will submit follow-up reports on the course, etc. of adverse events.

1) Initial report

When learning of the occurrence of a serious adverse event, the principal investigator (or instructed subinvestigator or study collaborator) will report the occurrence of the serious adverse event and its details in oral or written form to the head of the study site and the HAMLET-P Coordinating Center within 24 hours (by the next business day at the latest). In addition, the specified fields on the report concerning the serious adverse event will be promptly filled out to the extent possible and submitted to the head of the study site and the HAMLET-P Coordinating Center.

2) Follow-up reports

If any new information is obtained, the principal investigator will send follow-up/additional information to the HAMLET-P Coordinating Center as required.

【HAMLET-P coordination center】

[Before September 30, 2017]

Kanagawa Institute of Industrial Science and Technology (KISTEC)
Global health research coordinating center
E-mail：hamlet-p@newkast.or.jp

[After October 1, 2017]

Clinical research data center, St. Marianna University
E-mail：mariadc_ham@marianna-u.ac.jp

18.7.2 Reporting Obligations of the Principal Investigator and Reporting Procedures

The principal investigator will determine the seriousness, presence/absence of causal relationship, and predictability of the adverse event reported by the study doctor at the site. The principal investigator will then judge the necessity of reporting the adverse event to regulatory authorities and promptly report it to other subinvestigators and the company providing the study drug.

The principal investigator can also refer the matter to the Data and Safety Monitoring Committee when they determine that it is necessary to do so.

18.7.3 Obligations of the HAMLET-P Coordinating Center

HAMLET-P Coordinating Center will support the principal investigator, the company providing the study drug, and the monitor so that each piece of safety information can be transmitted smoothly without delay. The Center will also support the preparation of various reports, submit the reports to regulatory authorities on behalf of the principal investigator, as well as accumulate and manage the safety information.

18.7.4 Adverse Events Required to be Reported to Regulatory Authorities

In accordance with relevant laws and regulations, if an adverse event that is subject to reporting to the regulatory authorities occurs during the study period, the report should be made to the regulatory authorities according to the prescribed procedures.

18.8Responsibilities of the Principal Investigator and the Data and Safety Monitoring

Committee

18.8.1 Determination of the Necessity of Enrollment Suspension

The principal investigator will determine the urgency, importance, and level of impact of the reported event, as well as the presence/absence of an impact on the continuity of the study, and take actions such as the temporary suspension of enrollment and immediate notification to the subinvestigator or study collaborator of the matters to be disseminated.

18.8.2 Reporting to the Data and Safety Monitoring Committee

The principal investigator will promptly report a serious adverse event that occurs during the study in written form to the Data and Safety Monitoring Committee only when they determine that it is necessary to refer the event to the Committee. The principal investigator can also seek the opinion of the Data and Safety Monitoring Committee on the presence/absence of a relationship of the event to the study drug and the appropriateness of the decision on whether the event is expected or unexpected.

18.8.3 Examination by the Data and Safety Monitoring Committee

The Data and Safety Monitoring Committee will examine the reported details of the adverse event and will provide written recommendations to the principal investigator for future actions, including the propriety of the continuation of enrollment and whether a protocol revision is required.

19. Statistical Analysis（Rapid）

19.1 Sample size calculation and power justification

Pulse arm 4 subjects, Oral prednisolone arm 4 subjects

If a total of 8 subjects are enrolled and then 3 subjects are assigned to the pulse group and 5 subjects are assigned to the p.o. group, another subject will be enrolled for assessment. Apart from this, subjects shall be enrolled as many as possible during the enrollment period.

Rationale:

Of all patients with HAM, rapid progressors are as few as approximately 5%. Furthermore, the annual number of patients who developed HAM is approximately 50 in total and only several patients are expected to meet the criteria for rapid progressor in a year. Thus, it is not possible to enroll more than 10 patients. Therefore, the number of rapid progressors in this study was set as the maximum number of subjects that can be enrolled during the enrollment period (4 in the pulse group and 4 in the p.o. group).

Rapid progressor (Clinical trial①)

The primary endpoint of this study was set to be “the proportion of subjects whose 10-meter timed walk improved by ≥30%compared to baseline value on Day 15 or the OMDS improved by ≥1” based on clinical significance. Subjects with such a meaningful improvement are expected to be at least 50% in the pulse group and approximately 0 to 10% in the p.o. group. Study results will be assessed based on the number of subjects with the primary endpoint improvement. More specifically, if the number of subjects showing improvement was higher in the pulse group than in the p.o. group, the pulse therapy can be considered as more effective than the p.o. therapy. Given each group consists of 4 subjects, the probability to obtain a result indicating the higher effectiveness of the pulse rate was calculated. The calculation results are shown in Table 1 below. This table shows the following:

- When the expected improvement ratio in the pulse group is 75% (3 out of 4), even if the expected rate in the p.o. group is 25% (1 out of 4), the probability of having a higher number of subjects with improvement in the pulse group is not less than 0.8.
- When the expected ratio in the pulse group is 50% (2 out of 4) and the expected rate in the p.o. group is not more than 10%, the probability of having a higher number of subjects with improvement in the pulse group is not less than 0.8.

Based on the above results and the expected improvement ratio in each group, it was considered possible to assess the results with 4 subjects in each group in this study.

Table 1. Probability of having a higher number of subjects with clinically meaningful improvement in the pulse group than the p.o. group with 4 subjects in each group The vertical axis (%) shows the expected ratio of subjects with improvement in the pulse group and the horizontal axis (%) shows the expected ratio of subjects with improvement in the p.o. group.

	5%	10%	15%	20%	25%
50%	0.886	0.831	0.774	0.715	0.656
55%	0.917	0.870	0.819	0.766	0.710
60%	0.941	0.902	0.859	0.812	0.761
65%	0.960	0.929	0.893	0.852	0.807
70%	0.974	0.951	0.922	0.888	0.849
75%	0.984	0.967	0.945	0.918	0.886

19.2Methods of analysis

19.2.1 Definitions of data sets analyzed

Rapid progressor (Clinical trial①)

- Efficacy Analysis Set
  - Full Analysis set (FAS)
    A population of all randomized subjects, excluding the following subjects:
    - Subjects who have never received study treatment
    - Subjects who do not have both a pre-treatment 10-meter timed walk and an OMDS
    - Any subject who is subsequently found to have violated eligibility criteria
  - Per Protocol set (PPS)
    A population of subjects in the FAS who have no major protocol deviations
- Safety Analysis Set
  A population of all subjects who received at least one dose of study treatment. Secondary analyses will also be performed on a population excluding subjects who are found not to meet any of the inclusion criteria or meet any of the exclusion criteria after enrollment.

19.2.2 Baseline characteristics

The number and percentage (%) will be calculated for discrete values. Continuous values will be summarized by calculating mean, standard deviation, minimum, first quartile, median, third quartile, and maximum values.

19.2.3 Analysis of the primary efficacy endpoint

The FAS will be the primary analysis set, and secondary analyses will be performed in the PPS as well.
If any of the 10-meter timed walk or the OMDS on Day 15 is missing, the subject will be considered to have no improvement. The proportion of improvement for the primary endpoint and its 95% confidence interval will be calculated by group. The estimates of the treatment effect will be based on the difference in the proportion of improvement between treatment groups (pulse group - p.o. group) and its 95% confidence interval. When the difference in the proportion of improvement between groups is positive, pulse therapy is considered to be more effective than p.o. therapy.

19.2.4 Analysis of Secondary Endpoints

The main analysis set will be the FAS, and secondary analyses will also be conducted in the PPS. Details of the analysis methods will be described in a separate statistical analysis plan. When analyzing the change in the 10-meter timed walk, the measured value will be logarithmically transformed for analysis.

Slow progressor (Clinical trial②)

Slow Progressor

7. Objectives（Slow）

- Primary objective

  To test the efficacy of oral prednisolone therapy in patients with slowly progressive HAM/TSP.

- Secondary objective

  To test the safety of of oral prednisolone therapy in patients with slowly progressive HAM/TSP.

8. Study Design and Evaluation（Slow）

- Study design: Double-blind, randomized, placebo-controlled trial
- Primary outcome measure
  - Efficacy

    ＜Comparison between the predinisolone group and the placebo group＞

    Change in 10mWT at Day169 (Week 24) from baseline
- Secondary outcome measures
  - Efficacy

    ＜Comparison between the predinisolone group and the placebo group＞

    - 10mWT
      Change at Day29 (Week 4)/ Day85 (Week 12) compared to baseline
    - 2-minute walk distance and 6-minute walk distance
      Change at Day29 (Week 4) / Day85 (Week 12)/ Day169 (Week 24) compared to baseline
    - CSF neopterin concentration
      Change at Day169 (Week 24) compared to baseline
    - Proportion of subjects who discontinued the study drug during the study drug treatment priod (Day 1 ~ Day169 (Week 24))

    ＜Comparison in the placebo group＞

    - Walking tests (10mWT, 2-minute and 6-minute walk distance)
      Change at Day169 (Week 24) compared to baseline and at Day337 (Week 48) compared to Day169 (Week 24)
    - CSF neopterin concentration
      Change at Day169 (Week 24) compared to baseline and at Day337 (Week 48)

Slow progressor (Clinical trial②)

compared to Day169 (Week 24)

- Safety

  Adverse events (frequency, severity)

9. Target Sample Size and Study Period（Slow）

- Target sample sizse: 40 subjects
- Study period: August 2016 to August 2021

  （The secondary enrollment is possible until the end of June 2020.）

  However, the study period may be changed depending on the status of study progress.

10. Treatment Plan（After Secondary Enrollment）（Slow）

Figure 4 : Treatment flow chart (Slow progressor)

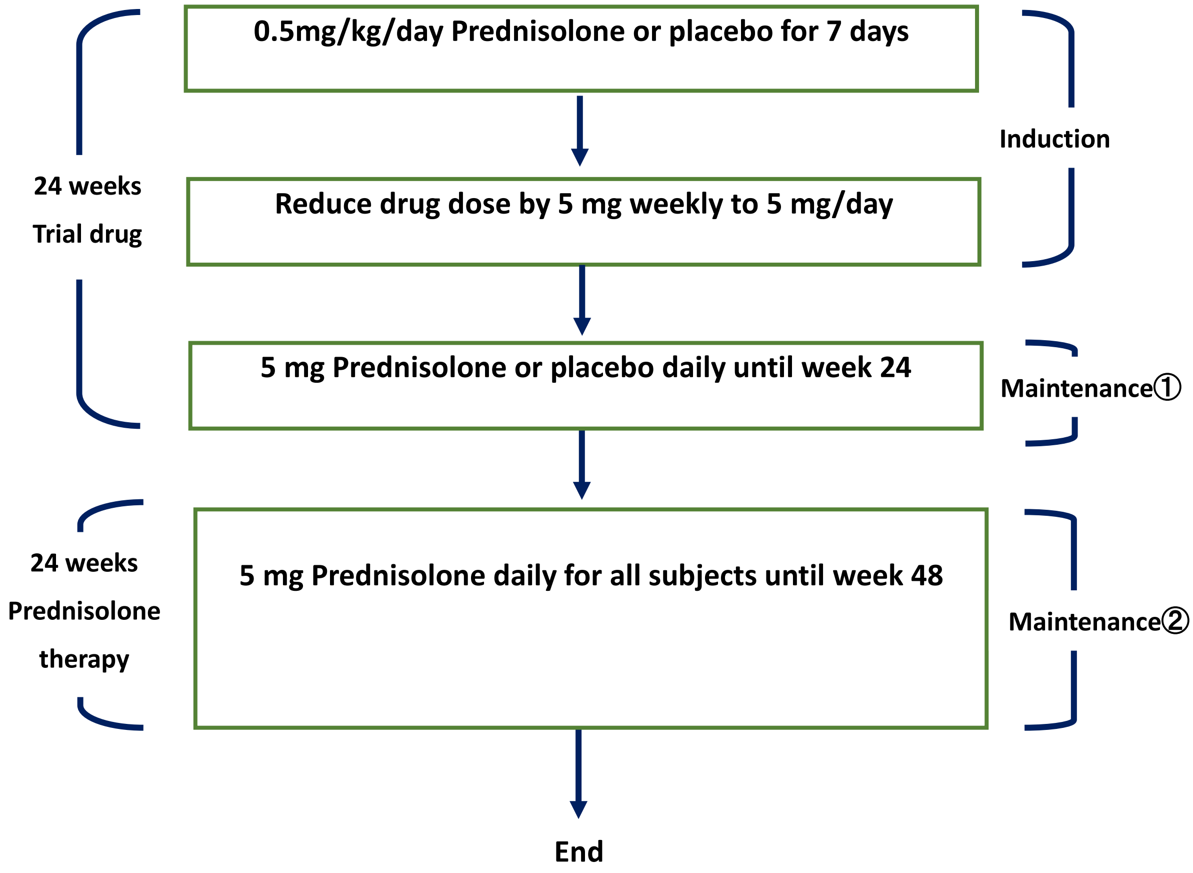

No additional treatment until Week 24. If a subject experienced worsening of symptoms of HAM and meets the criteria for dose increase or additional dose increase between the Week 24 visit and Week 48, additional treatment shall be performed according to

the procedure.

Prior to starting the study drug treatment, it shall be confirmed that the test values on the last assessment date meet the following criteria.

⑧ Neutrophil count: ≥1,500/mm3
⑨ Platelets: ≥100,000/mm3
⑩ Hemoglobin: ≥9.0 g/dL
⑪ AST: ≤3-times the upper limit of normal (ULN)
⑫ ALT: ≤3-times the ULN
⑬ Serum creatinine: ≤1.5-times the UL
⑭ HbA1c（NGSP）: ≤6.5%

＜Explanation for Figure 4＞

Patients are treated in the outpatient or inpatient setting.

Induction･Maintenance ① Treatment is prescribed according to the treatment plan (Figure 4).

Visits: Week 4 (Day29±7), Week 12 (Day85±7), Week 24 (Day169±7)

- Patients are initiated on 0.5mg/kg prednisone or placebo per day for 7 days [Day1～Day7]
- The drug dose is tapered in 5mg weekly decrements (every 7 days) to 5 mg prednisolone or placebo once daily. For the remaining treatment time until Week 24 is reached, patients are treated with 5 mg once daily [Day8～Day169]

Maintenance ②

Visits: Week 28 (Day197±7), Wek 32 (Day225±7), Week 36 (Day253±7), Week 48 (Day337±7)

- All subjects shall take prednisolone 5 mg/day daily. [Until Day 337]

The key code shall not be opened until the end of the study period (48 weeks) for all slow progressors.

10.1. Dose change (dose increase) after Week 24 visit

If a subject experienced worsening of symptoms of HAM and meets the criteria for dose increase or additional dose increase after the Day 169 (Week 24) visit, additional treatment shall be performed according to the procedure (maximum dose of 10 mg/day at the time of the increase) within 7 days from the date when it is confirmed that the subject meets the criteria. Dose increase or additional dose increase are permitted based on the values of the 10-meter timed walk and the OMDS obtained on an

Slow progressor (Clinical trial②)

unscheduled study visit.

If a subject meets the dose increase/resumption criteria, the HAMLET-P Coordinating Center should be contacted before the dose increase/resumption.
After the dose increase, no transition to the next dose level shall be made until the end of the evaluation, regardless of the acceptable range.

- Determination based on OMDS

  [Dose increase criteria]

  "≥1 grade of worsening in the OMDS" compared to Day169 (Week 24)

  [Dose increase procedures]

  Prednisolone is increased by 2.5 mg/day (5 mg every other day) /4 weeks.

- Determination based on the walk test

① [Dose increase criteria]

≥10% worsening in the 10-meter timed walk compared to the value on Day 169 (Week 24) was observed in the test at 2 study visits after Day 169 (Week 24). (Those 2 visits can be apart from each other.) If the walk test cannot be performed on Day 169 (Week 24) and the value is missing, the most recent result prior to Day 169 (Week 24) shall be used as the value on Day 169 (Week 24).

[Dose increase procedures]

The dose of prednisolone shall be increased to 7.5 mg/day (5 mg or 10 mg every other day) and the oral administration is continued at that dose.

② [Additional increase criteria]

If a test performed at the visit after an increase to 7.5 mg/day (5 mg/10 mg every other day) shows a 10% or more worsening in the 10-meter timed walk compared to the value observed at the time of the increase

[Additional increase procedures]

The dose of prednisolone shall be increased to 10.0 mg/day and the oral administration is continued at that dose.

<u>Dose Modification Confirmation Procedures</u>

① If a subject meets the above dose increase criteria, the investigator or study collaborator shall contact the HAMLET-P Coordinating Center by email.

② The HAMLET-P Coordinating Center shall determine if the subject is eligible for additional treatment.

③ After confirming the eligibility of additional treatment, the HAMLET-P Coordination Center shall notify the investigator or study collaborator by email whether

additional treatment is permitted or not.

【HAMLET-P coordination center】
[Before September 30, 2017]
Kanagawa Institute of Industrial Science and Technology (KISTEC)
Global health research coordinating center
E-mail：hamlet-p@newkast.or.jp

[After October 1, 2017]
Clinical research data center, St. Marianna University
E-mail：mariadc_ham@marianna-u.ac.jp

10.2. Prednisolone Administration Rules

③ Oral administration after breakfast is recommended. Dose
- Do not split or crush the study drug.
- The dose should be set in 2.5 mg increments
- For the dose of 0.5 mg/kg/day, round off the fraction.

④ Timing of oral administration
- Take the drug once a day.
- Oral administration after breakfast is recommended.

11. Administration Discontinuation and Study Discontinuation（Slow）

11.1Administration Discontinuation

The beginning and end time of administration of the study drug and whether administration is completed will be recorded in the CRF. Furthermore, if the administration is discontinued, the time of the administration discontinuation and the reasons will be recorded in the CRF.

11.2Criteria for Study Discontinuation

If a patient meets any of the following restrictions, said patient will be discontinued from the study:

(1) Up to Day 169 (Week 24):
Unequivocal HAM-induced disease progression (at least 100% worsening of 10-meter timed walk compared to baseline)
After Day 169 (Week 24) visit:
Unequivocal progression of HAM-induced disease progression (at least 100%

worsening of 10-meter timed walk compared to baseline) despite additional treatment

(2) If a subject develops steroid-induced glaucoma.
(3) If a patient develops an adverse event that would make it difficult for them to continue participation in the study as determined by the principal investigator or subinvestigator.
(4) If HBV-DNA is detected in the patient.
(5) If a patient requests discontinuation.
(6) If a patient is found to be ineligible for the study
(7) When the principal investigator or subinvestigator determines that the patient should be discontinued from the study

11.3 Discontinuation Procedures

- If a patient is discontinued from the study, the study doctor will promptly notify the Coordinating center of the study discontinuation of said patient. If a patient wishes to discontinue participation in the study, the study doctor will confirm the reasons for discontinuation to the extent possible.
- The day when the patient is confirmed to meet the withdrawal criteria will be the date of discontinuation. The tests at study discontinuation will be performed on the day closest to the date of discontinuation on which tests can be performed.
- For subjects who discontinue the study due to clinical exacerbation of HAM, data will be collected on routine visit frequency for "details of post-treatment (only treatment for HAM [including drugs listed in Exclusion Criterion 1 and Exclusion Criterion 6 and treatment with HAL]), "10-meter timed walk", and "Osame's Motor Disability Score" until the period corresponding to Week 24.
- The observation of adverse events, including follow-up observation of any existing adverse events and observation of new occurrences of adverse events, will be performed up to at least 28 days after the final administration of the study drug. However, any adverse events that have not resolved at Week 48 and for which a causal relationship to the study drug cannot be ruled out will be followed-up on even after Week 48 until they are resolved or improved. However, this will not apply to any of the following cases where the study doctor determines that the follow-ups are not required:
    - If subsequent treatment is initiated and a causal relationship to the study drug is not evaluable.
    - If a patient is lost to follow-up because they have been transferred to another hospital, etc.
    - If a patient refuses to be followed-up on.
    - If a patient has died.

Slow progressor (Clinical trial②)

- If a patient has not yet improved but their symptoms are stable, or when the principal investigator or subinvestigator determines that no further resolution can be expected.

12. Drug Information（Slow）

12.1 Study Drug (Predinosolone or placebo)

The slow progressor will receive the study drug (Day 1 to Week 24) of prednisolone listed below or placebo that matches with prednisolone and cannot be differentiated from the active drug.
The slow progressor will receive the study drug (after the end of assessment on Week 24 to Week 48) of prednisolone.

1） Component code
HAM-PSL

2） Generic name and brand name
Generic name: Prednisolone
Brand name: Prednisolone tablet 5mg (NP)

3） Pharmacologic category
Synthetic corticosteroid

4) Composition and properties
Chemical name:
11β, 17, 21-Trihydroxypregna-1, 4-diene-3, 20-dione
Molecular formula:
$C_{21}H_{28}O_5$
Molecular weight:
360.44
Structure:

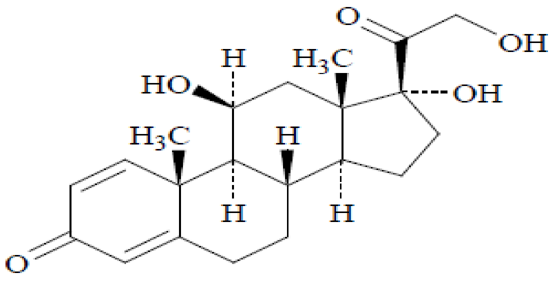

Composition:

・ Active ingredient (in 1 tablet)

Prednisolone 5 mg

・ Excipients (in 1 tablet)

Lactose hydrate, corn starch, microcrystalline cellulose, talc, calcium carmellose, gelatin, calcium stearate

Form:

White scored uncoated tablet

Size:

Diameter (mm): 7.0, thickness (mm): 3.4, weight (mg): 150

5） Storage method and expiration period

Storage method: Store at room temperature

Expiration period: For 3 years from the date of manufacture

6） Packaging form

Press through package (PTP)

7） Labeling

Information included in the labeling for the study drug is “for study use,” “study code,” “lot number,” “storage conditions,” and “expiration date.” For details, refer to the Standard Operating Procedures (SOPs) relating to study drug management.

8） Study drug provision

The study drug will be provided to the study site following IRB approval and submission of the Clinical Trial Notification

12.2Procedures for Storage, Management, and Disposal

The investigational product accountability manager at the study site will store/manager the study drug appropriately according to the “Standard Operating Procedure for Study Drug Management” and prepare the records on the stock, status of use, and disposal of the study drug. The investigational product accountability manager will confirm the number of unused and used study drugs (including empty vials and containers) as needed. For details on the provision, storage, management, and disposal of the study drug, refer to the “Standard Operating Procedure for Study Drug Management”.

12.3Expected Adverse Drug Reactions

The following side effects have been reported, so sufficient monitoring should be performed when using this drug, and appropriate measures should be taken if any side effects occur. For details and latest information on adverse drug reactions, please refer to the latest package insert of Prednisolone tablet 5 mg NP.

Slow progressor (Clinical trial②)

Significant adverse reactions (frequency)

1) Infection (unknown frequency)
2) Secondary adrenal insufficiency, diabetes mellitus (unknown frequency)
3) Gastrointestinal perforation, hemorrhage, peptic ulcer (unknown frequency)
4) Pancratitis (unknown frequency)
5) Psychiatric disturbance, depression, seizure (unknown frequency)
6) Osteoporosis (unknown frequency), osteonecrosis, myopathy (unknown frequency
7) Glaucoma, Subcapsular posterior cataract, central serous chorioretinopathy, multifocal posterior pigment epitheliopathy (unknown frequency)
8) Thrombosis (unknown frequency)
9) Myocardial infarction, cerebral infarction, aneurysm (unknown frequency)
10) Epidural lipoma (unknown frequency)
11) Tendon rupture (unknown frequency)

13. Concomitant Drugs and Therapies（Slow）

13.1 Prohibited Concomitant Drugs

Until the completion of the observation period (Week 48), the concomitant use of the following drugs considered to have an effect on the evaluation of the study drug will be prohibited:

① Therapeutic drugs for the treatment of HAM/TSP other than the study drug
Concomitant use will be prohibited during the study period.

② Immuno-suppresive drugs and corticosteroids (systemic administration)
Concomitant use will be prohibited during the study period.

③ Medications for gait symptoms associated with HAM (restricted concomitant use)
Concomitant use of medications to relieve symptoms of HAM (muscle relaxants, Botox, etc.)

- Is prohibited throughout the study if not used within 2 weeks prior to the date of informed consent.
- If they have been used within 2 weeks prior to the date of informed consent, it may be used concomitantly even after participation in the study at the stable dose without change from the time of participation throughout the study.

④ Pentosan polysulfate, vitamins and supplements

- Pentosan polysulfate (prohibited concomitant use)
  Concomitant use is prohibited throughout the study.

Slow progressor (Clinical trial②)

- ➢ Prosultiamine (prohibited concomitant use)
  Concomitant use is prohibited throughout the study.
- ➢ Ascorbic acid (restricted concomitant use)
  - ・ Use of 1.5 g/day or more is prohibited throughout the study.
  - ・ Concomitant use is prohibited throughout the study if not used prior to the primary enrollment.
  - ・ If it has been used since before the primary enrollment, it may be used concomitantly after the primary enrollment at the stable dose without change from the time of primary enrollment throughout the study.
- ➢ Vitamins and supplements used for HAM other than those listed above (restricted concomitant use)
  - ・ Concomitant use is prohibited throughout the study if not used prior to the primary enrollment.
  - ・ If it has been used since before the primary enrollment, it may be used concomitantly after the primary enrollment at the stable dose without change from the time of primary enrollment throughout the study.

⑤ Live or attenuated vaccinations
Concomitant use will be prohibited during the study period.

⑥ Hypnotics (restricted concomitant use)
The use of intermediate and long-acting drugs is prohibited throughout the study.
The use of the ultra-short-acting form is prohibited within 4 hours before the efficacy evaluation, and the short-acting form is prohibited within 10 hours before the efficacy evaluation.

⑦ Strong CYP3A4 inducers and inhibitors (prohibited concomitant use)
Concomitant use is prohibited throughout the study.

⑧ Other study drugs
Concomitant use will be prohibited during the study period.

⑨ Desmopressin Acetate Hydrate
Concomitant use will be prohibited during the study period.

13.2Drugs requiring caution for concomitant use

Review the latest package insert.

13.3Allowed Concomitant Drugs and Therapies

13.3.1 Allowed Concomitant Drugs

Slow progressor (Clinical trial②)

The concomitant use of drugs is allowed in the following cases.

Note that the name, administration and dosage, route of administration, administration period, and the reasons for the administration of the drugs concomitantly used from the start date of administration of the study drug to the completion of the observation period will be recorded in the CRF

① When drugs other than prohibited concomitant drugs for the treatment of symptoms accompanying complications or the primary disease have been used since before participation in the study, the continuous use of these drugs will be allowed after the start of the study

② Drugs other than prohibited concomitant drugs can be used for symptomatic treatment of adverse events that occur after the start of administration of the study drug.

③ The prophylactic use of the drugs for adverse events that have already been observed once will be allowed.

＜Recommended concomitant drugs＞

It is recommended to co-medicate participants with the following medications to prevent adverse reactions associated with corticosteroids:

1） Antiacids (H2-blocker, etc.)

2） Osteoporosis prevention (Bisphosphonate, Vitamin D2, etc.)

＜Other Allowed Concomitant Drugs＞

Recommended symptomatic treatment, prophylactic use, and therapy for complications are shown below (as references).

1) Hyperglycemia

As glucose metabolism abnormalities may occur and cause high blood sugar levels due to administration of this study drug, if such signs are observed, appropriate measures such as the use of blood sugar control drugs should be promptly taken.

2) Infectious disease

As administering this study drug may decrease immune function and increase susceptibility to infection, if any infection or signs of infection are observed, use antibiotics and take appropriate measures promptly.

3) Insomnia

As administration of the study drug may cause insomnia, if such signs are observed, appropriate measures such as the use of sleep medications should be taken promptly.

4) Muscle tightness (spasms)

If antispasticity drugs (tizanidine hydrochloride, eperisone hydrochloride, baclofen, etc.) are being orally taken for muscle tightness (spasms) caused by HAM at the time of primary enrollment, the continuous use of those drugs are allowed.

However, dose modifications and new start of oral administration during the study period are not permitted.

5) Decrease in voiding function

For decreased urinary function (neuropathic bladder, overactive bladder) caused by HAM disease, the medications used for the symptoms at the time of primary enrollment (e.g., propiverin hydrochloride, sorifenacin succinate, imidafenacin, prazocin hydrochloride, and distigmine bromide) may be used continuously even after the primary enrollment.

However, any change in oral dosage or start of new oral administration during the study period is not permitted.

6) Neuropathic pain

For neuropathic pain caused by HAM disease, the medications used for the symptoms at the time of primary enrollment (pregabalin, duloxetine hydrochloride, amitriptyline hydrochloride, clonazepam, etc.) may be used continuously even after primary enrollment.

However, any change in oral dosage or start of new oral administration during the study period is not permitted.

7) Skin disease and localized allergy symptoms such as pollinosis

For skin disease (eczema, etc.) and allergy symptoms such as pollinosis, the use of topical corticosteroids/corticosteroid nasal sprays/corticosteroid eye drops is allowed. If local steroid therapy is being performed at the time of primary enrollment and it will also be performed after the primary enrollment, the same drugs should be used before and after the primary enrollment.

For skin disease, the use of topical corticosteroids is allowed as symptomatic treatment of adverse events that occur after the start of administration of the study drug.

13.3.2 Allowed Concomitant Therapies

If concomitant therapies are performed, the name or details of the therapies performed, period of the therapies, and the reasons for the therapies will be recorded in the CRF.

1） Physical therapy

If physical therapy is performed for functional impairment caused by HAM prior to the

primary enrollment, its continuation is allowed during the study period. However, changing of the content or frequency of the therapy and starting of new therapy is not allowed during the study period.

2) Self-catheterization
If a subject has undergone self-catheterization prior to the primary enrollment instance for functional impairment due to HAM disease, it is acceptable to continue during the study. However, in principle, changing of the frequency and starting of new procedure is not allowed during the study period.

13.4 Subsequent Treatment after Study Completion/Discontinuation

There are no specific regulations on subsequent treatment after the completion/discontinuation of the study.

14. Observation, Investigations, Test Items, and Implementation Period （Slow）

14.1 Observation Schedule

14.1.1 Screening

When the start of study drug administration is defined as Day 1, the screening tests will be performed between Weeks -16 and -12. Screening tests (Weeks -16 to -12) and tests in the progressor assessment period on Week -12 may be performed on the same day.

14.1.2 Progressor assessment period

When the start of study drug administration is defined as Day 1, the progressor assessment period will be 12 weeks from Week -12 to the last assessment date.

14.1.3 Sudy drug treatment period

When the start of study drug administration is defined as Day 1, the study drug treatment period will be 24 weeks from Day 1 to Week 24

14.1.4 Sudy drug (Prednisolone) treatment period

When the start of study drug administration is defined as Day 1, this will be 24 weeks from Week 24 to Week 48. Other than when the study is discontinued due to the subject’s request, follow-up will be performed after the study discontinuation date until Week 48 unless the subject is lost to follow-up.

14.1.5 Post-observation period

The post-observation period will be 28 days from the last administration of the study drug.

Slow progressor (Clinical trial②)

14.2Patient demographic information

Date consent was obtained for participation in the study, subject identification number, sex, date of birth, height, body weight, ethnicity, Clinical history of HAM/TSP (date of diagnosis, date of onset, OMDS), history of previous treatments, presence/absence of complications (the name of the disease(s) if complications are present, presence/absence of treatment at the start of the study), presence/absence of past medical history (the name of the disease(s) if a patient has a past medical history), familial history of HAM/TSP and ATL, blood transfusion history.

14.3Observation, Investigations, and Test Items

Investigations of patients will be performed according to the parameters and schedule shown in Section 14.4 Study Schedule.

14.3.1 Clinical Assessment

(1) Physical examination

In physical examination, information will be collected on any symptoms and findings that are considered clinically meaningful.

The information shall be handled as “past medical history/complications” prior to administration of the study drug and “adverse events” after administration of the study drug.

(2) Blood Pressure, Pulse Rate, Body Temperature

Blood pressure and pulse rate will be measured at rest in the sitting position. Axillary temperature will be used for body temperature.

(3) Height, Body Weight

(4) Overall Evaluation (Using a VAS)

<Implementation methods>

Since HAM/TSP not only causes gait disturbance and urination disorder but also has impact on daily life due to its various symptoms, the patient will be instructed to comprehensively express the level of its impact using a VAS as the evaluation method. The patient is instructed to look at a black line with a length of 10 cm (the left end with “condition with no impact of HAM/TSP and no impact on daily life” and the right end

with “worst impact of HAM/TSP ever”) and point at the current level of their overall condition. Give the VAS questionnaire (Appendix 2) to the subject to complete.

(5) Evaluation of Ambulatory Status (Using a VAS)

<Implementation methods>

A black line with a 10cm length (the right end indicating “no effect on walking by HAM at all” and the left end indicating “worst impact on walking by HAM so far) will be presented to a subject and the subject will indicate the extent of current ambulatory status. Give the VAS questionnaire (Appendix 2) to the subject to complete.

(6) Evaluation of Lower Extremity Pain (Using a VAS)

＜Implementation methods＞

The patient is instructed to look at a black line with a length of 10 cm (the left end with “no pain” and the right end with “worst pain ever”) and point at their current level of pain (Appendix2).

(7) Ambulatory aid used at the clinic (= outdoors during a clinic visit)

＜Implementation methods＞

Type of walking aid used at the site visit and any changes from the previous visit will be collected (Appendix1).

(8) Ambulatory aid used at home (= indoors)

＜Implementation methods＞

Type of walking aid used at home and any changes from the previous visit will be collected (Appendix1).

(9) 10 meter Walk Test (Seconds)

The person in charge of assessment of the clinical efficacy endpoints will perform the evaluation in accordance with the “Standard Operating Procedure for Performance of Walk Test”.

<Implementation methods>

Place markings with tape, etc. at the start and finish points of a straight 10-meter long line on the floor. The time required to walk the length of the 10-meter course will be measured using a stopwatch.

Measurements must be taken twice and both measurements will be recorded. Data will be assessed using the mean values (rounded to two decimal places). If 1 of the 2

measurements is missing, the 1 measurement value will be used as is for evaluation.

(10) 6 minute Walk Distance (meters)

The person in charge of assessment of the clinical efficacy endpoints will perform the evaluation in accordance with the "Standard Operating Procedure for Performance of Walk Test".

<Implementation methods>

Place markings with tape, etc. at the start and finish points of a straight 10 meter long line on the floor. Have the patient repeatedly walk from the start point to the finish point and back and measure the distance the patient is able to walk during a 6 minute period. If a 6 minute walk is difficult for a patient, the actual distance walked and the time taken will be recorded

(11) 2 minute Walk Distance (meters)

The person in charge of assessment of the clinical efficacy endpoints will perform the evaluation in accordance with the "Standard Operating Procedure for Performance of Walk Test".

<Implementation methods>

When the 6 minute walk distance test is performed, the distance the subject walked in 2 minutes after the start of the walk will be simultaneously measured and record as data for the 2 minute walk distance.

If a 2 minute walk is difficult for a patient, the actual distance walked and the time taken will be recorded.

(12) Timed Up and Go Test (seconds)

The person in charge of assessment of the clinical efficacy endpoints will perform the evaluation in accordance with the "Standard Operating Procedure for Performance of Walk Test".

<Implementation methods>

Place markings with tape, etc. at the start and finish points of a straight 3 meter long line on the floor. Have the patient walk from the start point to the finish point and back and measure the time required for the walk with a stopwatch. The patient will be instructed to perform these series of movements at both "a normal walking speed" and "a maximum walking speed" (a total of 2 sets of movements) according to directions from the person performing the measurements. After measurements are taken twice,

the smaller measurement (faster time) will be used and recorded in seconds to one decimal place. (Round numbers to one decimal place)

(13) Osame Motor Disability Score

<Implementation methods>

The patient's condition will be determined according to the table and scored (Appendix 1).

(14) IPEC 1

The patient's condition will be determined according to the table and scored (Appendix 1).

(15) Modified IPEC 2

<Implementation methods＞

Patients wil fill in the Questionnaire sheet (Appendix 1).

(16) Modified Ashworth scale（MAS）

<Implementation methods＞

The patient's condition will be determined according to the table and scored (Appendix 1).

(17) OABSS

<Implementation methods＞

The patient will undergo an interview investigation (Appendix 1).

(18) ICIQ-SF

<Implementation methods＞

The patient will undergo an interview investigation (Appendix 1).

(19) N-QOL

<Implementation methods＞

Patients wil fill in the Questionnaire sheet (Appendix 1).

(20) IPSS

<Implementation methods＞

The patient will undergo an interview investigation (Appendix 1).

Slow progressor (Clinical trial②)

(21) **Sexual Health Inventory for Men**

<Implementation methods＞

Patients wil fill in the Questionnaire sheet (Appendix 1).

14.3.2 Blood tests

Items with the term 'Accompanying Research Items' will be performed as an accompanying research to understand the duration of efficacy of prednisolone treatment, the influences and effects on the subject's immune system, and prognostic and progressive disease categorical factors of clinical progression. Consent for participation in the accompanying research will be separately obtained from the subject

(1) Safety

＜Test parameters＞

① Complete blood count with differential
② Urea nitrogen and electrolytes (BUN, Cre, Na, K)
③ Liver function (AST, ALT, ALP, T-Bil)
④ Glucose metabolism (HbA1c (NGSP), Fasting blood glucose)
⑤ Bone metablism (Ca, P)
⑥ Lipids (T-Cho, LDL-C, HDL-C, TG)
⑦ Hepatitis B virus tests (HBc antibody, HBs antibody, HBs antigen, HBV-DNA)
⑧ HCV antibody
⑨ HIV-1 antibody, HIV-2 antibody

＜Institutions peforming measurements＞

The tests shall be performed at the Clinical Laboratory of each study site. Qualitative HBV-DNA shall be performed according to local laboratory procedures.

(2) Research

＜Test parameters＞

① β2-microglobrin (Accompaning research)
② sVCAM-1 (Accompaning research)
③ SPARC (Accompaning research)
④ sIL-2R (Accompaning research)
⑤ HTLV-1 proviral load (Accompaning research)
⑥ B- and T-cell subsets/markers (Accompaning research)
⑦ HTLV-1 antibody for HAM/TSP diagnosis

Slow progressor (Clinical trial②)

＜Implementation methods＞

- Test parameters ①②③④⑤⑥:
  The tests shall be perform by central measurement. The procedure for processing and transporting samples is described in the “Standard Operating Procedure for Sample Processing for Central Measurement”.
- Test parameter ⑦:
  The tests shall be performed according to local laboratory procedures.

＜Institutions peforming measurements＞

④: SRL, Inc.

①,②,③,⑤,⑥: The Department of Rare Diseases Research, Institute of Medical Sciences, St. Marianna University School of Medicine.

⑦: The tests shall be performed according to local laboratory procedures.

14.3.3 Urinalysis

＜Test parameters＞

① Urine glucose
② Urine protein
③ Pregnancy test

＜Institutions peforming measurements＞

The tests shall be performed at the Clinical Laboratory of each study site.

14.3.4 Cerebrospinal Fluid tests

(1) General test 1

＜Test parameters＞

① Cell counts/ Cell fractionation
② Total protein
③ Glucose

＜Implementation methods＞

Fifteen drops of cerebrospinal fluid will be collected in Spitz tubes.

＜Institutions peforming measurements＞

The tests shall be performed at the Clinical Laboratory of each study site.

(2) Genaral test 2

Slow progressor (Clinical trial②)

＜Test parameters＞

Neopterin concentration

＜Implementation methods＞

Three mL of cerebrospinal fluid will be collected in Spitz tubes
The procedure for processing and transporting samples is described in the “Standard Operating Procedure for Sample Processing for Central Measurement”.

＜Institutions peforming measurements＞

SRL, Inc.

(3) Research

＜Test parameters＞

① HTLV-1 antibody (for HAM diagnosis (at screening only) and Accompaning research)
② HTLV-1 proviral load (Accompaning research)
③ CXCL10 (Accompaning research)
④ sVCAM-1 (Accompaning research)

＜Implementation methods＞

The tests shall be perform by central measurement. The procedure for processing and transporting samples is described in the “Standard Operating Procedure for Sample Processing for Central Measurement”.

＜Institutions peforming measurements＞

①:SRL, Inc
②,③,④:The Department of Rare Diseases Research, Institute of Medical Sciences, St. Marianna University School of Medicine

However, samples for the test item ② HTLV-1 proviral load will be stored in the form of DNA and, in principle, measured at the global research institute (Imperial College London, UK).

Test item ③ CXCL10 will be measured using CXCL10 measurement kits (Cosmic Corporation Co., Ltd., Becton Dickinson &Company).

14.3.5 MRI

<Test site>

The brain (T1, T2) and the cervical to upper thoracic spinal cord (T1, T2) shall be measured.

Slow progressor (Clinical trial②)

<Institution performing measurements>

The tests shall be performed at each site. However, the tests to be performed before the secondary enrollment is not required at each study site if data performed at another hospital can be obtained.

<Institution performing evaluations>

As a general rule, MRI image assessments will be performed on all subjects enrolled at the primary enrollment at an international collaborative research organization (NIH, U.S.).

14.3.6 Intraocular pressure

＜Implementation methods＞

Follow each site’s measurement method. Throughout the study period, the method of measurement should be unified for the same subject.

Slow progressor (Clinical trial②)

14.4Schedule Table

X = mandatory, (x) = optional

Period		Screening	1st Enrollment*1	Progressor Assessment				2nd Enrollment*2	Study drug treatment				Prednisolone treatment				Unplanned Visits*20	Post-observation*4 +28	At discontinuation*5
Week		-16 ～ - 12*1		-12*1	-8	-4	Last assessment*2		0	4	12	24*3	28	32	36	48			
Day		-112～-84 ±7		-84 ±7	-56 ±7	-28 ±7	±7		1*2	29 ±7	85 ±7	169 ±7	197 ±7	225 ±7	253 ±7	337 ±7			
Informed consent		x																	
Eligibility confirmation		x																	
Study drug treatment									Prednisolone or Placebo				Prednisolone						
Clinical Assessment	Physical examination	x		x	x	x	x	Randomization		x	x	x	x	x	x	x		x	x
	Blood pressure, Pulse rate, Body temperature	x		x	x	x	x			x	x	x	x	x	x	x			x*6
	Height, Body weight	x										x*7				x*7			
	OMDS	x		x	x	x	x			x	x	x	x	x	x	x	x		x
	Walking tests *A	x		x	x	x	x			x	x	x	x	x	x	x	x		x*8
	Walking aids *B	x		x	x	x	x			x	x	x	x	x	x	x	x		
	MAS	x		x	x	x	x			x	x	x	x	x	x	x			
	IPEC1	x		x	x	x	x			x	x	x	x	x	x	x			
	VAS *C	x		x	x	x	x			x	x	x	x	x	x	x			
	QOL*D	x		x	x	x	x			x	x	x	x	x	x	x			

Slow progressor (Clinical trial②)

Period		Screening	1st Enrollment *1	Progressor Assessment				2nd Enrollment *2	Study drug treatment				Prednisolone treatment				Unplanned Visits *20	Post-observation *4 +28	At discontinuation *5
Week		-16 ～ - 12 *1		-12 *1	-8	-4	Last assessment *2		0	4	12	24 *3	28	32	36	48			
Day		-112～-84 ±7		-84 ±7	-56 ±7	-28 ±7	±7		1 *2	29 ±7	85 ±7	169 ±7	197 ±7	225 ±7	253 ±7	337 ±7			
	Urinary dysfunction *E	x		x	x	x	x			x	x	x	x	x	x	x			
Blood tests *F/ Urinalysis *G		x		x	x	x	x			x	x	x	x			x			x
Virus tests *14 *H		x *9								x *11	x *10	x *10	x *11			x *11			x *12
Pregnancy test *13		x					(x)												
Cerebrospinal fluid tests *14 *I		x *15					x			(x)		x		(x) *16	(x) *16	x			
Accompaning research	Blood tests *J	x		x	x	x	x		x	x	x	x	x			x			
Accompaning research	Biobank samples (Blood) *17	x		x	x	x	x		x	x	x	x	x			x			
Accompaning research	Cerebrospinal Fluid tests *K	x *15					x			(x)		x		(x) *16	(x) *16	x			
Accompaning research	Biobank samples (Cerebrospinal fluid) *17	x *15					x			(x)		x		(x) *16	(x) *16	x			
MRI		x *18		x *18						(x)		x				(x)			
Intraocular pressure		x *18		x *18						x	x *19 x *19	x *19			x *19	x *19			

Slow progressor (Clinical trial②)

＊1: The progressor assessment period on Week -12 and screening can be performed on the same day. In this case, the items specified in the screening should be performed. In addition, the primary enrollment should occur within 7 days of screening.

＊2: Secondary enrollment should be performed within 7 days of the last assessment date to start Day 1 (study drug treatment). The date may be the same as the date of the final assessment.

＊3: For subjects who discontinue the study due to clinical exacerbation of HAM, data will be collected on "details of the post-treatment regimen (for treatment of HAM [including the drugs listed in Exclusion Criterion 1 and Exclusion Criterion 6 and treatment with HAL], "10-meter timed walk", and "Osame's Motor Disability Score" until the period corresponding to Week 24 in the study treatment period.

＊4: To be performed 28 days (+28 days) after the final dose of the study drug.

＊5: To be performed within 28 days after discontinuation of the study drug during the treatment period or before the start of post-treatment, whichever is earlier.

＊6: Body temperature will be measured as needed.

＊7: Height measurement is not required.

＊8: 10-meter timed walk, 6-minute walk distance, and 2-minute walk distance will be performed. Timed up-and-go test is not required.

＊9: HBs antigen, HBs antibody, HBc antibody, HCV antibody, HIV-1 antibody and HIV-2 antibody will be tested only at screening. If either HBs antibody or HBc antibody is positive, quantitative HBV-DNA measurement will be performed.

However, quantitative HBV-DNA is not required if the positive HBs antibody is clearly due to vaccination.

*10: Quantitative HBV-DNA measurement will be performed only in subjects who are positive for HBs or HBc antibodies (with the exception of positive HBs antibody clearly due to vaccination) at screening and whose quantitative HBV-DNA is below the detection sensitivity.

*11: Subjects who are positive for HBs or HBc antibodies (with the exception of positive HBs antibody clearly due to vaccination) at screening and whose quantitative HBV-DNA is below the detection sensitivity will be closely monitored for liver function tests, and if abnormal values are observed (if AST and ALT increase is ≥ 5 times the reference value at the site), quantitative HBV-DNA measurement will be performed.

*12: For subjects who are positive for HBs or HBc antibodies (with the exception of positive HBs antibody clearly due to vaccination) at screening and whose quantitative HBV-DNA is below the detection sensitivity, if the liver function test shows abnormal values (if AST and ALT increase is ≥ 5 times the reference value at the site), quantitative HBV-DNA measurement will be performed.

*13: It is not required for subjects who are permanently sterilized. (Permanent sterilization: postmenopausal [with no menses for at least 48 weeks with no other medical reason], post sterilization [hysterectomy, bilateral salpingectomy, bilateral oophorectomy], tubal occlusion without tubal ligation)

*14: If data is available from a previous test on anti-HTLV-1 antibody for diagnosis of HAM, that data may be used.

*15: Data obtained within 12 weeks prior to the date of consent may be used.

Slow progressor (Clinical trial②)

*16: When increasing prednisolone treatment due to exacerbation, it is recommended to perform a cerebrospinal fluid test before.

*17: Residual samples will be used as samples for biobanking.

*18: MRI and intraocular pressure measurement will be performed at any visit from screening to the progressor assessment period and the data will be used as the baseline value. For MRI scans, data obtained within 12 weeks prior to the date of informed consent can be used.

However, if an image used as the baseline shows findings suggestive of inflammation in the spinal cord, a retest must be performed within 12 weeks prior to the secondary enrollment for that site only. In addition, if data obtained within 12 weeks prior to the date of informed consent at another hospital is available and indicates no findings of inflammation in the spinal cord, a retest is not required.

*19: To be performed only in subjects with glaucoma at week 8, 12, 24, 36, and 48

*20: Unscheduled visit is allowed to confirm the extent of disease progression. At the unscheduled visit, only the Osame's Motor Disability Score, the walk test (10-meter timed walk), and the use of walking aids will be performed.

*A：Waling tests (10 meter Timed Walk, 6 minute Walk Distance, 2 minute Walk Distance, Timed Up and Go Test)

*B：Ambulatory aid used at the clinic and home

*C：VAS（Overall, Walking, Lower extremity pain）

*D：QOL (Modified IPEC2, N-QOL, Sexual Health Inventory for Men）

*E：Urinary dysfunction (OABSS, ICIQ-SF, IPSS)

*F：Blood tests： Complete blood count with differential, Urea nitrogen and electrolytes (BUN, Cre, Na, K), Liver function (AST, ALT, ALP, T-Bil),
Glucose metabolism (HbA1c (NGSP), Fasting blood glucose), Bone metablism (Ca, P), Lipids (T-Cho, LDL-C, HDL-C, TG)

*G：Urinalysis: glucose, protein

*H：Virus tests: Hepatitis B virus (HBc antibody ((at screening only), HBs antibody (at screening only), HBs antigen (at screening only), HBV-DNA)
HCV antibody (at screening only), HIV-1 antibody･HIV-2 antibody (at screening only)
Anti-HTLV-1 antibody for HAM diagnosis (at screening only)

*I：Cerebrospinal fluid tests: Anti-HTLV-1 antibody for HAM diagnosis (at screening only)
Safety: Glucose
Efficacy: Cell counts/ Cell fractionation, Toral protein, neopterin

*J：Blood tests (Accompanying research): β2-microgloblin, B- and T-cell subsets/markers、sIL-2R、HTLV-1 proviral load, sVCAM-1、SPARC

Slow progressor (Clinical trial②)

*K：Cerebrospinal fluid tests (Accompanying research): HTLV-1 anribody, HTLV-1 proviral load, CXCL10, sVCAM-1

Slow progressor (Clinical trial②)

15. Management of Patients（Slow）

15.1 Notification to Other Departments/Hospitals

The principal investigator or subinvestigator will confirm whether the patient will visit other departments or hospitals before the start of administration of the study drug. If the patient has already visited another department or hospital, the principal investigator or subinvestigator will notify the patient's primary care physician of their participation in the study. If the patient uses any drugs other than those prescribed by the principal investigator or subinvestigator, the name of the drug(s) and the status of usage will be investigated.
If the patient newly visits another department or hospital during the study, the principal investigator or subinvestigator will take the same action as above.

15.2 Notification to Patients

The principal investigator, subinvestigator, or study collaborator will give the following instructions to patients:

＜Visits＞

In principle, the progressor assessment period, the treatment period of study drug, and the observation period will be performed on an outpatient basis. During visits, subjects will undergo the prescribed tests in accordance with the explanations and instructions of the Principal Investigator, etc.

For any abnormality, the principal investigator, etc. must be contacted and the subject must visit the hospital at any time.

16. Evaluation Criteria/Response Evaluation（Slow）

16.1 Safety Evaluation

Adverse events (frequency and severity)

The worst grade of each adverse event, as well as the number and proportion of patients who develop the event will be obtained by setting the number of patients, regardless of their eligibility/ineligibility, who receive any part of the treatment specified in the protocol (all subjects who receive treatment) as the denominator using the CTCAE v4.0 Japanese translation JCOG/JSCO version.

Slow progressor (Clinical trial②)

16.2Efficacy Evaluation

Refer to the primary and secondary outcome measures.

17. Data Reporting Method（Slow）

17.1Target for Preparation of Case Report Forms

Case report forms shall be created for all enrolled subjects.

17.2Preparation and Storage of Case Report Forms

In this study, case report forms shall be prepared and submitted in the EDC system called eCB.
After the completion of specified tests/observations, the principal investigator shall promptly enter data and sign on the eCB. If the subinvestigator or study collaborator completes data entry, the principal investigator shall review and check the details and then sign the form. For the method of input and correction, follow the guide which will be separately prepared.
If the study collaborator enters data into the case report form, only the extent that the data can be transcribed from the source documents is acceptable. The subinvestigators and study collaborators who can fill in the case report forms must be registered in the “List of subinvestigators and study collaborators”.
After data lock, the principal investigator shall obtain and store the CD-R containing the input data (including the correction history) from the data center.

17.3Identification of Source Documents

The source documents in this study are as follows:

- Medical records
- Diagnostic imaging films
- List of names for screening
- Informed consent forms
- Study drug accountability log
- Output form for general laboratory tests result
- QOL questionnaire, VAS questionnaire, and questionnaire for assessment of dysuria
- Clinical Efficacy Endpoint Assessment Form

The following items will be directly entered onto a CRF, which can be handled as a source

document. However, items that are accompanied by medical judgments must be directly entered onto a CRF by the principal investigator or subinvestigator.

1) The following items related to adverse events:
   - Corresponding CTCAE term
   - Seriousness
   - Causal relationship to the study drug and comments on causal relationship
2) Reasons for the performing of concomitant treatment
3) Reasons for determining why an adverse event did not require follow-up

17.4 Procedures for Handling Missing, Unused, and Abnormal Data

For missing, unused, and abnormal data not expected before the start of the study, the principal investigator and the statistician will discuss and decide how to handle the data. Data handling details will be described in the analysis reports.

18. Adverse Event Reporting（Slow）

18.1 Definition of Adverse Events

An adverse event is any unfavorable or unintended sign (including abnormal variations in laboratory test findings), symptom, or disease that occurs after the start of treatment, regardless of relationship with the study drug. It also includes an exacerbation of a pre-existing condition during the study.

In this study, when a pre-existing condition worsens by one level or more from the grade originally observed before the start of the initial administration of the study drug according to CTCAE v4.0 criteria, it is considered an adverse event. For laboratory test parameters not specified in the protocol, refer to Section 18.2. A progression of the disease (HAM/TSP) is not handled as an adverse event. However, when a condition cannot be clearly determined, it should be handled as an adverse event.

18.2 Abnormal Values for Laboratory Tests Whose Measurements are not specified in the Protocol

Any abnormal laboratory test values that meet the following conditions are handles as an adverse event:

1) If it meets the definition of serious adverse event

2) If it is related to any changes in the study drug (changes in dose, interruption or discontinuation of the study drug) or when it is related to any changes in concomitant treatment (addition, change, or discontinuation of concomitant treatment)

3) If it is accompanied by clinical symptoms

4) If it is determined be clinically problematic

18.3Serious Adverse Events

An adverse event that meets any of the following conditions is determined to be serious:

(1) Death

- Death during the study, regardless of whether there is causal relationship to the study.
- Deaths that occur after the study for which a causal relationship to the study cannot be ruled out. Deaths suspected to be related to treatment for the study are included. Deaths clearly due to primary disease are not included.

(2) Life-threatening

- Grade 4 non-hematotoxicity
- Grade4 hematotoxicity (excluding Leukocytosis)

(3) Adverse events that require hospitalization or prolonging of existing hospitalization. However, hospitalization for the following purposes is not handled as a serious adverse event:

- Hospitalization scheduled before the start of the study
- Hospitalization previously planned, such as for the purpose of reducing the burden on patients, due to visiting the hospital from far locations

(4) Results in persistent or significant disability/incapacity

(5) Congenital anomaly/birth defect in later generations

(6) Other medically important conditions

This refers to conditions that are not included in (2) to (5) above, but are considered to be medically important.

18.4Causal Relationship to the Study Dru

The principal investigator or subinvestigator will determine the causal relationship to the study drug by classifying it into the following five types:

- Definite: Definitely due to study treatment
- Probable: Probably due to study treatment
- Possible: Possibly due to study treatment
- Unlike: Unlikely due to study treatment

- Not related: Not related to study treatment

18.5 Determination of the Severity of an Adverse Event

The severity (Grade) of an adverse event will be determined using the CTCAE v4.0 Japanese translation JCOG version and the Grade for the event which is closest to the definition of the event term will be allocated.

18.6 Classification of Intervention against an Adverse Event

Interventions against an adverse event will be selected from and classified into the following six types (selection of multiple types of intervention except for type 0 is possible):

0: No intervention performed
1: Decrease in dose or interruption of the study drug
2: Discontinuation of the study
3: Treatment with any drugs
4: Treatment using methods other than drugs
5: Hospitalization or prolonging of existing hospitalization
6: Other

18.7 Reporting Procedures if a Serious Adverse Event Occurs

18.7.1 Reporting Obligations of the Principal Investigator and Subinvestigator and Reporting Procedures

If a serious adverse event occurs, the study doctor will promptly perform appropriate intervention and also immediately report it to the principal investigator. Upon request of the principal investigator, the study doctor will submit follow-up reports on the course, etc. of adverse events.

1) Initial report

When learning of the occurrence of a serious adverse event, the principal investigator (or instructed subinvestigator or study collaborator) will report the occurrence of the serious adverse event and its details in oral or written form to the head of the study site and the HAMLET-P Coordinating Center within 24 hours (by the next business day at the latest). In addition, the specified fields on the report concerning the serious adverse event will be promptly filled out to the extent possible and submitted to the head of the study site and the HAMLET-P Coordinating Center.

2) Follow-up reports

Slow progressor (Clinical trial②)

If any new information is obtained, the principal investigator will send follow-up/additional information to the HAMLET-P Coordinating Center as required.

【HAMLET-P coordination center】

[Before September 30, 2017]

Kanagawa Institute of Industrial Science and Technology (KISTEC)
Global health research coordinating center
E-mail：hamlet-p@newkast.or.jp

[After October 1, 2017]

Clinical research data center, St. Marianna University
E-mail：mariadc_ham@marianna-u.ac.jp

18.7.2 Reporting Obligations of the Principal Investigator and Reporting Procedures

The principal investigator will determine the seriousness, presence/absence of causal relationship, and predictability of the adverse event reported by the study doctor at the site. The principal investigator will then judge the necessity of reporting the adverse event to regulatory authorities and promptly report it to other subinvestigators and the company providing the study drug.

The principal investigator can also refer the matter to the Data and Safety Monitoring Committee when they determine that it is necessary to do so.

18.7.3 Obligations of the HAMLET-P Coordinating Center

HAMLET-P Coordinating Center will support the principal investigator, the company providing the study drug, and the monitor so that each piece of safety information can be transmitted smoothly without delay. The Center will also support the preparation of various reports, submit the reports to regulatory authorities on behalf of the principal investigator, as well as accumulate and manage the safety information.

18.7.4 Adverse Events Required to be Reported to Regulatory Authorities

In accordance with relevant laws and regulations, if an adverse event that is subject to reporting to the regulatory authorities occurs during the study period, the report should be made to the regulatory authorities according to the prescribed procedures.

18.8 Responsibilities of the Principal Investigator and the Data and Safety Monitoring Committee

Slow progressor (Clinical trial②)

18.8.1 Determination of the Necessity of Enrollment Suspension

The principal investigator will determine the urgency, importance, and level of impact of the reported event, as well as the presence/absence of an impact on the continuity of the study, and take actions such as the temporary suspension of enrollment and immediate notification to the subinvestigator or study collaborator of the matters to be disseminated.

18.8.2 Reporting to the Data and Safety Monitoring Committee

The principal investigator will promptly report a serious adverse event that occurs during the study in written form to the Data and Safety Monitoring Committee only when they determine that it is necessary to refer the event to the Committee. The principal investigator can also seek the opinion of the Data and Safety Monitoring Committee on the presence/absence of a relationship of the event to the study drug and the appropriateness of the decision on whether the event is expected or unexpected

18.8.3 Examination by the Data and Safety Monitoring Committee

The Data and Safety Monitoring Committee will examine the reported details of the adverse event and will provide written recommendations to the principal investigator for future actions, including the propriety of the continuation of enrollment and whether a protocol revision is required.

19. Statistical Analysis（Slow）

19.1Sample size calculation and power justification

<u>Twenty subjects in the Prednisolone group and 20 subjects in the placebo group</u>

Data analysis of Daily clinical practice in patients with HAM was performed to estimate the change and standard deviation in the logarithmically transformed 10-meter timed walk (log 10mWT) with prednisone treatment. The data assessed are Data Set A consisting of 14 Japanese patients with HAM who received prednisolone treatment and Data Set B consisting of 71 patients with HAM who were observed for 6 months. Data Set B is composed of 41 patients in Japan, 27 patients in the UK, and 3 patients in Brazil, and includes the period during which the patients in Japan are receiving steroid treatment. Steroid treatment was not administered to the patients in the UK and Brazil.
First, the effect of prednisolone treatment was estimated. In Data Set A, log 10mWT decreased by 0.165 in 6 months compared to pre-treatment (improved by 15%). On the

Slow progressor (Clinical trial②)

other hand, in the patient group (n=30) in which steroid treatment was not performed in Data Set B, the disease worsened by 0.0575 over 6 months (worsened by 6%). Next, the standard deviation for log 10mWT was estimated. A log 10mWT regression model at 6 months adjusted for the values at the beginning of observation was created using Japanese data from Data Set B to estimate the standard deviation for the error section and the estimated value was 0.21. Therefore, the estimated difference between groups due to this study intervention is around 0.223, and the estimated standard deviation of log 10mWT when performing analysis of covariance is around 0.21.

Under the above estimate, when the significance level is set at 0.05 (two-sided), and the group comparison is performed with an analysis of covariance adjusted for the baseline values, the statistical power of 0.8 or more can be obtained with the number of patients of greater than or equal to 30. Tables 2, 3, and 4 show the number of patients required for the statistical power of 0.8, 0.85, and 0.9, respectively. It is expected that approximately 40 subjects will be enrolled at sites participating in this study during the enrollment period. Given the differences between the groups and standard deviations as estimated above, the statistical power of 0.9 can be obtained with 40 patients. In addition, even if the standard deviation is estimated to be about 15% larger, 0.24, the statistical power of 0.8 can be maintained, indicating a somewhat conservative setting. Therefore, the target number of patients for this study is 40.

Table 2. Required sample size (statistical power of 80%)

Treatment effect	SD										
	0.17	0.18	0.19	0.20	0.21	0.22	0.23	0.24	0.25	0.26	0.27
−0.248（22%減少）	16	18	20	22	24	26	28	32	34	36	40
−0.236（21%減少）	18	20	22	24	26	30	32	34	38	40	44
−0.223（20%減少）	20	22	24	28	30	32	36	38	42	44	48
−0.211（19%減少）	22	24	28	30	34	36	40	42	46	50	54
−0.198（18%減少）	26	28	30	34	38	40	44	48	52	56	60
−0.186（17%減少）	28	32	34	38	42	46	50	54	58	64	68
−0.174（16%減少）	32	36	40	44	48	52	56	62	66	72	78
−0.163（15%減少）	36	40	44	50	54	60	64	70	76	82	88
−0.151（14%減少）	42	46	52	58	62	68	74	82	88	96	102
−0.139（13%減少）	48	54	60	66	74	80	88	96	104	112	120

Slow progressor (Clinical trial②)

Table 3. Required sample size (statistical power of 85%)

Treatment effect	SD										
	0.17	0.18	0.19	0.20	0.21	0.22	0.23	0.24	0.25	0.26	0.27
−0.248 (22%減少)	18	20	24	26	28	30	32	36	38	42	44
−0.236 (21%減少)	20	24	26	28	30	34	36	40	42	46	50
−0.223 (20%減少)	22	26	28	30	34	36	40	44	48	50	54
−0.211 (19%減少)	26	28	32	34	38	42	44	48	52	56	60
−0.198 (18%減少)	28	32	34	38	42	46	50	54	58	64	68
−0.186 (17%減少)	32	36	40	44	48	52	56	62	66	72	78
−0.174 (16%減少)	36	40	44	50	54	60	64	70	76	82	88
−0.163 (15%減少)	42	46	52	56	62	68	74	80	86	94	102
−0.151 (14%減少)	48	54	58	66	72	78	86	92	100	108	118
−0.139 (13%減少)	56	62	68	76	84	92	100	108	118	128	136

Table 4. Required sample size (statistical power of 90%)

Treatment effect	SD										
	0.17	0.18	0.19	0.20	0.21	0.22	0.23	0.24	0.25	0.26	0.27
−0.248 (22%減少)	22	24	26	30	32	34	38	42	44	48	52
−0.236 (21%減少)	24	26	30	32	36	38	42	46	50	54	58
−0.223 (20%減少)	26	30	32	36	40	42	46	50	54	60	64
−0.211 (19%減少)	30	32	36	40	44	48	52	56	62	66	70
−0.198 (18%減少)	32	36	40	44	50	54	58	64	68	74	80
−0.186 (17%減少)	38	42	46	50	56	60	66	72	78	84	90
−0.174 (16%減少)	42	46	52	58	62	68	76	82	88	96	102
−0.163 (15%減少)	48	54	60	66	72	78	86	94	102	110	118
−0.151 (14%減少)	56	62	68	76	84	92	100	108	118	126	136
−0.139 (13%減少)	64	72	80	88	98	106	116	126	138	148	160

19.2 Methods of analysis

19.2.1 Definitions of data sets analyzed

➢ <u>Efficacy Analysis Set</u>

- Full Analysis set (FAS)

  A population of all randomized subjects, excluding the following subjects:

  - Subjects who have never received study treatment
  - Subjects who do not have the pre-treatment primary outcome (10-meter timed walk)
  - Subjects who do not have any primary outcomes during the study drug treatment period (24 weeks)
  - Any subject who is subsequently found to have violated eligibility criteria

- Per Protocol set (PPS)

  A population of subjects in the FAS who have no major protocol deviations

➢ <u>Safety Analysis Set</u>

Slow progressor (Clinical trial②)

A population of all subjects who received at least one dose of study treatment. Secondary analyses will also be performed on a population excluding subjects who are found not to meet any of the inclusion criteria or meet any of the exclusion criteria after enrollment.

19.2.2 Handling of missing data

Discontinuation of study treatment occurs when a subject permanently discontinued his/her assigned investigational treatment. Withdrawal of study occurs when a subject died, is lost to follow-up, or withdraw consent. As long as consent to the study is obtained, data will be collected at the planned timepoints even after discontinuation of study treatment.
Analysis of the primary endpoint will be performed for the efficacy evaluation of investigational treatment through Week 24. Therefore, data after discontinuation of study treatment will be handled as missing.
Last Observation Carried Forward (LOCF) and Observed Case (OC) will be defined as data sets available for the way of handling of missing data. The OC is a data set in which data obtained at each timepoint will be handled as data at the timepoint. The LOCF is a data set in which data obtained will be handled as data obtained at the specified timepoint, and missing value data not obtained at the specified timepoint will be imputed with data most recently obtained. However, data at baseline will not be used for missing value imputation.

19.2.3 Baseline characteristics

The number and percentage (%) will be calculated for discrete values. Continuous values will be summarized by calculating mean, standard deviation, minimum, first quartile, median, third quartile, and maximum values.

19.2.4 Analysis of the primary efficacy endpoint

The FAS will be the primary analysis set, and secondary analyses will be performed in the PPS as well.
The primary efficacy endpoint is the 10-meter timed walk (10mWT) at Week 24. The 10mWT will be logarithmically transformed (log 10mWT) for use in the analysis, so that the values of 10mWT are distributed symmetrically as much as possible.
The null hypothesis of the primary comparison is that there is no difference in the primary endpoint between the prednisolone group and the placebo group. The primary analysis will be analyzed using a mixed model for repeated measure (MMRM) with the log 10mWT of baseline value, treatment group, timepoint (Day29, Week 12 and Week 24), and interaction between treatment group and timepoint as explanatory variables. This analysis will be

performed using an OC data set that is based on the FAS. Differences between treatment groups will be tested (in 2-sided) for those at Week 24. The significance level is 0.05.
As sensitivity analyses, analysis using an analysis of covariance model with log 10mWT of baseline values as covariates will be performed. This analysis will be performed using a LOCF data set that is based on the FAS. In addition, analysis will be performed adding factors used at randomization to the covariates. Subgroup analyses will be performed by gender, presence or absence of concomitant drugs, and disease duration. In addition, subgroup analyses will be performed based on whether or not subjects were enrolled in the HAL study prior to enrollment in the study.

19.2.5 Analysis of Secondary Endpoints

Analysis of secondary endpoints will be analyzed using the FAS. Details of the analysis methods will be described in a separate statistical analysis plan.

Non progressor（Accompanying Research）

Non progressor

7. Objectives（Non）

To explore prognostic and progressive disease categorical factors of clinical progression in patients with HAM.

8. Study Design and Evaluation（Non）

- Study design: Accompanying study
- Endpoints: Exploratory investigation of prognostic and progressive disease categorical factor

9. Target Sample Size and Study Period（Non）

- Target sample sizse: 5 subjects
  After reaching the target sample size of 5 subjects, subject enrollment will be continued until the target sample size of 40 in slow progressors has been reached or until the end of the study period.
- Study period: August 2016 to August 2021
  （The secondary enrollment is possible until the end of June 2020.）
  However, the study period may be changed depending on the status of study progress.

10. Treatment Plan（Non）

Patients are treated in the outpatient or inpatient setting.

Follow up

Visits：Week 12 (Day85±7days), Week 24 (Day169±7days), Week 48 (Day337±7days)

As an option, Monthly visits are allowed to confirm the extent of disease progression.

・ Follow up patients until Week48.

<Change of progressor category>

If a subject experiences worsening of symptoms during the observation period up to Week 48 and meets the definition of the rapid or slow progressor, the subject will finish the non-progressor study and start the secondary enrollment as a rapid or slow progressor, with the study treatment + observation according to the protocol for respective progressor. Test items specified on the “final assessment date” for the rapid or slow progressor will be performed before the secondary enrollment.

Non progressor（Accompanying Research）

11. Study Discontinuation（Non）

11.1 Criteria for Study Discontinuation

If a patient meets any of the following restrictions, said patient will be discontinued from the study:

(4) If a patient requests discontinuation.

(5) If a patient is found to be ineligible for the study

(6) When the principal investigator or subinvestigator determines that the patient should be discontinued from the study

11.2 Discontinuation Procedures

- If a patient is discontinued from the study, the study doctor will promptly notify the Coordinating center of the study discontinuation of said patient. If a patient wishes to discontinue participation in the study, the study doctor will confirm the reasons for discontinuation to the extent possible.
- The day when the patient is confirmed to meet the withdrawal criteria will be the date of discontinuation.

12. Drug Information（Non）

No study drug will be used.

13. Concomitant Drugs and Therapies（Non）

13.1 Prohibited Concomitant Drugs

Until the completion of the observation period (Week 48), the concomitant use of the following drugs considered to have an effect on the evaluation of the study drug will be prohibited:

① Methylprednisolone and prednisolone (systemic administration)
Concomitant use will be prohibited during the study period.

② Therapeutic drugs for the treatment of HAM/TSP other than the study drug
Concomitant use will be prohibited during the study period.

③ Immuno-suppresive drugs and corticosteroids other than ① (systemic administration)
Concomitant use will be prohibited during the study period.

④ Medications for gait symptoms associated with HAM (restricted concomitant use)
Concomitant use of medications to relieve symptoms of HAM (muscle relaxants, Botox,

etc.)

- Is prohibited throughout the study if not used within 2 weeks prior to the date of informed consent.
- If they have been used within 2 weeks prior to the date of informed consent, it may be used concomitantly even after participation in the study at the stable dose without change from the time of participation throughout the study.

Details can be found in 13.3.1. Concomitant Medications.

⑤ Pentosan polysulfate, vitamins and supplements

➢ Pentosan polysulfate (prohibited concomitant use)
Concomitant use is prohibited throughout the study.

➢ Prosultiamine (prohibited concomitant use)
Concomitant use is prohibited throughout the study.

➢ Ascorbic acid (restricted concomitant use)
- Use of 1.5 g/day or more is prohibited throughout the study.
- Concomitant use is prohibited throughout the study if not used prior to the primary enrollment.
- If it has been used since before the primary enrollment, it may be used concomitantly after the primary enrollment at the stable dose without change from the time of primary enrollment throughout the study.

➢ Vitamins and supplements used for HAM other than those listed above (restricted concomitant use)
- Concomitant use is prohibited throughout the study if not used prior to the primary enrollment.
- If it has been used since before the primary enrollment, it may be used concomitantly after the primary enrollment at the stable dose without change from the time of primary enrollment throughout the study.

⑥ Live or attenuated vaccinations
Concomitant use will be prohibited during the study period.

⑦ Hypnotics (restricted concomitant use)
The use of intermediate and long-acting drugs is prohibited throughout the study.
The use of the ultra-short-acting form is prohibited within 4 hours before the efficacy evaluation, and the short-acting form is prohibited within 10 hours before the efficacy evaluation.

⑧ Strong CYP3A4 inducers and inhibitors (prohibited concomitant use)
Concomitant use is prohibited throughout the study.

⑨ Other study drugs

Concomitant use will be prohibited during the study period.

13.2 Allowed Concomitant Drugs and Therapies

13.2.1 Allowed Concomitant Drugs

When drugs other than prohibited concomitant drugs for the treatment of symptoms accompanying complications or the primary disease have been used since before participation in the study, the continuous use of these drugs will be allowed after the start of the study.

13.2.2 Allowed Concomitant Therapies

1） Physical therapy

If physical therapy is performed for functional impairment caused by HAM prior to the primary enrollment, its continuation is allowed during the study period. However, changing of the content or frequency of the therapy and starting of new therapy is not allowed during the study period.

2) Self-catheterization

If a subject has undergone self-catheterization prior to the primary enrollment instance for functional impairment due to HAM disease, it is acceptable to continue during the study. However, in principle, changing of the frequency and starting of new procedure is not allowed during the study period.

13.3 Subsequent Treatment after Study Completion/Discontinuation

There are no specific regulations on subsequent treatment after the completion/discontinuation of the study.

14. Observation, Investigations, Test Items, and Implementation Period （Non）

14.1 Observation Schedule

14.1.1 Screening

The screening tests will be performed between Weeks -16 and -12. Screening tests (Weeks -16 to -12) and tests in the progressor assessment period on Week -12 may be performed on the same day.

14.1.2 Progressor assessment period

The progressor assessment period will be 12 weeks from Week -12 to the last assessment date.

Non progressor（Accompanying Research）

14.1.3 Observation period

This will be 48 weeks from Day 1 to Week 48. Other than when the study is discontinued due to the subject's request, follow-up will be performed until Week 48 unless the subject is lost to follow-up. Day 1 is defined as the day after the day when the walking test for the last assessment is performed.

14.2Patient demographic information

Date consent was obtained for participation in the study, subject identification number, sex, date of birth, height, body weight, ethnicity, Clinical history of HAM/TSP (date of diagnosis, date of onset, OMDS), history of previous treatments, presence/absence of complications (the name of the disease(s) if complications are present, presence/absence of treatment at the start of the study), presence/absence of past medical history (the name of the disease(s) if a patient has a past medical history), familial history of HAM/TSP and ATL, blood transfusion history.

14.3Observation, Investigations, and Test Items

Investigations of patients will be performed according to the parameters and schedule shown in Section 14.4 Study Schedule.

14.3.1 Clinical assessment

(1) Physical examination

In physical examination, information will be collected on any symptoms and findings that are considered clinically meaningful.

The information shall be handled as "past medical history/complications" prior to administration of the study drug and "adverse events" after administration of the study drug.

(2) Blood Pressure, Pulse Rate, Body Temperature

Blood pressure and pulse rate will be measured at rest in the sitting position. Axillary temperature will be used for body temperature.

(3) Height, Body Weight

(4) Overall Evaluation (Using a VAS)

<Implementation methods>

Non progressor（Accompanying Research）

Since HAM/TSP not only causes gait disturbance and urination disorder but also has impact on daily life due to its various symptoms, the patient will be instructed to comprehensively express the level of its impact using a VAS as the evaluation method. The patient is instructed to look at a black line with a length of 10 cm (the left end with “condition with no impact of HAM/TSP and no impact on daily life” and the right end with “worst impact of HAM/TSP ever”) and point at the current level of their overall condition. Give the VAS questionnaire (Appendix 2) to the subject to complete.

(5) Evaluation of Ambulatory Status (Using a VAS)

<Implementation methods>

A black line with a 10cm length (the right end indicating “no effect on walking by HAM at all” and the left end indicating “worst impact on walking by HAM so far) will be presented to a subject and the subject will indicate the extent of current ambulatory status. Give the VAS questionnaire (Appendix 2) to the subject to complete.

(6) Evaluation of Lower Extremity Pain (Using a VAS)

＜Implementation methods＞

The patient is instructed to look at a black line with a length of 10 cm (the left end with “no pain” and the right end with “worst pain ever”) and point at their current level of pain (Appendix2).

(7) Ambulatory aid used at the clinic (= outdoors during a clinic visit)

＜Implementation methods＞

Type of walking aid used at the site visit and any changes from the previous visit will be collected (Appendix1).

(8) Ambulatory aid used at home (= indoors)

＜Implementation methods＞

Type of walking aid used at home and any changes from the previous visit will be collected (Appendix1).

(9) 10 meter Walk Test (Seconds)

The person in charge of assessment of the clinical efficacy endpoints will perform the evaluation in accordance with the “Standard Operating Procedure for Performance of Walk Test”.

<Implementation methods>

Place markings with tape, etc. at the start and finish points of a straight 10-meter long line on the floor. The time required to walk the length of the 10-meter course will be measured using a stopwatch.
Measurements must be taken twice and both measurements will be recorded. Data will be assessed using the mean values (rounded to two decimal places). If 1 of the 2 measurements is missing, the 1 measurement value will be used as is for evaluation.

(10) 6 minute Walk Distance (meters)

The person in charge of assessment of the clinical efficacy endpoints will perform the evaluation in accordance with the “Standard Operating Procedure for Performance of Walk Test”.

<Implementation methods>

Place markings with tape, etc. at the start and finish points of a straight 10 meter long line on the floor. Have the patient repeatedly walk from the start point to the finish point and back and measure the distance the patient is able to walk during a 6 minute period. If a 6 minute walk is difficult for a patient, the actual distance walked and the time taken will be recorded

(11) 2 minute Walk Distance (meters)

The person in charge of assessment of the clinical efficacy endpoints will perform the evaluation in accordance with the “Standard Operating Procedure for Performance of Walk Test”.

<Implementation methods>

When the 6 minute walk distance test is performed, the distance the subject walked in 2 minutes after the start of the walk will be simultaneously measured and record as data for the 2 minute walk distance.
If a 2 minute walk is difficult for a patient, the actual distance walked and the time taken will be recorded.

(12) Timed Up and Go Test (seconds)

The person in charge of assessment of the clinical efficacy endpoints will perform the evaluation in accordance with the “Standard Operating Procedure for Performance of Walk Test”.

<Implementation methods>

Non progressor（Accompanying Research）

Place markings with tape, etc. at the start and finish points of a straight 3 meter long line on the floor. Have the patient walk from the start point to the finish point and back and measure the time required for the walk with a stopwatch. The patient will be instructed to perform these series of movements at both “a normal walking speed” and “a maximum walking speed” (a total of 2 sets of movements) according to directions from the person performing the measurements. After measurements are taken twice, the smaller measurement (faster time) will be used and recorded in seconds to one decimal place. (Round numbers to one decimal place)

(13) Osame Motor Disability Score

<Implementation methods>

The patient’s condition will be determined according to the table and scored (Appendix 1).

(14) IPEC 1

The patient’s condition will be determined according to the table and scored (Appendix 1).

(15) Modified IPEC 2

<Implementation methods＞

Patients wil fill in the Questionnaire sheet (Appendix 1).

(16) Modified Ashworth scale（MAS）

<Implementation methods＞

The patient’s condition will be determined according to the table and scored (Appendix 1).

(17) OABSS

<Implementation methods＞

The patient will undergo an interview investigation (Appendix 1).

(18) ICIQ-SF

<Implementation methods＞

The patient will undergo an interview investigation (Appendix 1).

(19) N-QOL

Non progressor（Accompanying Research）

<Implementation methods＞

Patients wil fill in the Questionnaire sheet (Appendix 1).

(20) IPSS

<Implementation methods＞

The patient will undergo an interview investigation (Appendix 1).

(21) **Sexual Health Inventory for Men**

<Implementation methods＞

Patients wil fill in the Questionnaire sheet (Appendix 1).

14.3.2 Blood tests

Items with the term 'Accompanying Research Items' will be performed as an accompanying research to understand the duration of efficacy of methylprednisolone and prednisolone treatment, the influences and effects on the subject's immune system, and prognostic and progressive disease categorical factors of clinical progression. Consent for participation in the accompanying research will be separately obtained from the subject

(1) Gereral test

＜Test parameters＞

① Complete blood count with differential
② Urea nitrogen and electrolytes (BUN, Cre, Na, K)
③ Liver function (AST, ALT, ALP, T-Bil)
④ Glucose metabolism (HbA1c (NGSP), Fasting blood glucose)
⑤ Bone metablism (Ca, P)
⑥ Lipids (T-Cho, LDL-C, HDL-C, TG)
⑦ Hepatitis B virus tests (HBc antibody, HBs antibody, HBs antigen, HBV-DNA)
⑧ HCV antibody
⑨ HIV-1 antibody, HIV-2 antibody

＜Institutions peforming measurements＞

The tests shall be performed at the Clinical Laboratory of each study site. Qualitative HBV-DNA shall be performed according to local laboratory procedures.

(2) Research

＜Test parameters＞

⑧ β2-microglobrin (Accompaning research)

Non progressor（Accompanying Research）

⑨ sVCAM-1 (Accompaning research)
⑩ SPARC (Accompaning research)
⑪ sIL-2R (Accompaning research)
⑫ HTLV-1 proviral load (Accompaning research)
⑬ B- and T-cell subsets/markers (Accompaning research)
⑭ HTLV-1 antibody for HAM/TSP diagnosis

＜Implementation methods＞

- Test parameters ①②③④⑤⑥:
  The tests shall be perform by central measurement. The procedure for processing and transporting samples is described in the "Standard Operating Procedure for Sample Processing for Central Measurement".
- Test parameter ⑦:
  The tests shall be performed according to local laboratory procedures.

＜Institutions peforming measurements＞

④: SRL, Inc.
①,②,③,⑤,⑥: The Department of Rare Diseases Research, Institute of Medical Sciences, St. Marianna University School of Medicine.
⑦: The tests shall be performed according to local laboratory procedures.

14.3.3 Urinalysis

＜Test parameters＞
① Urine glucose
② Urine protein
③ Pregnancy test

＜Institutions peforming measurements＞
The tests shall be performed at the Clinical Laboratory of each study site.

14.3.4 Cerebrospinal Fluid tests

(1) General test 1

＜Test parameters＞
① Cell counts/ Cell fractionation
② Total protein
③ Glucose

＜Implementation methods＞

Non progressor（Accompanying Research）

Fifteen drops of cerebrospinal fluid will be collected in Spitz tubes.

＜Institutions peforming measurements＞

The tests shall be performed at the Clinical Laboratory of each study site.

(2) Genaral test 2

＜Test parameters＞

Neopterin concentration

＜Implementation methods＞

Three mL of cerebrospinal fluid will be collected in Spitz tubes

The procedure for processing and transporting samples is described in the “Standard Operating Procedure for Sample Processing for Central Measurement”.

＜Institutions peforming measurements＞

SRL, Inc.

(3) Research

＜Test parameters＞

① HTLV-1 antibody (for HAM diagnosis (at screening only) and Accompaning research)

② HTLV-1 proviral load (Accompaning research)

③ CXCL10 (Accompaning research)

④ sVCAM-1 (Accompaning research)

＜Implementation methods＞

The tests shall be perform by central measurement. The procedure for processing and transporting samples is described in the “Standard Operating Procedure for Sample Processing for Central Measurement”.

＜Institutions peforming measurements＞

①：SRL, Inc

②,③,④：The Department of Rare Diseases Research, Institute of Medical Sciences, St. Marianna University School of Medicine

However, samples for the test item ② HTLV-1 proviral load will be stored in the form of DNA and, in principle, measured at the global research institute (Imperial College London, UK).

Non progressor（Accompanying Research）

Test item ③ CXCL10 will be measured using CXCL10 measurement kits (Cosmic Corporation Co., Ltd., Becton Dickinson &Company).

14.3.5 MRI

<Test site>

The brain (T1, T2) and the cervical to upper thoracic spinal cord (T1, T2) shall be measured.

<Institution performing measurements>

The tests shall be performed at each site. However, the tests to be performed before the secondary enrollment is not required at each study site if data performed at another hospital can be obtained.

<Institution performing evaluations>

As a general rule, MRI image assessments will be performed on all subjects enrolled at the primary enrollment at an international collaborative research organization (NIH, U.S.).

14.3.6 Intraocular pressure

＜Implementation methods＞

Follow each site’s measurement method. Throughout the study period, the method of measurement should be unified for the same subject.

Non progressor（Accompanying Research）

14.4Schedule Table

X = mandatory, (x) = optional

Period		Screening	1st Enrollment*1	Progressor Assessment				Observation				Observation	
Week		-16 ～ - 12*1		-12*1	-8	-4	Last assessment*2 ±7	(x)*3	12	(x)*3	24	(x)*3	48
Day		-112〜-84 ±7		-84 ±7	-56 ±7	-28 ±7			85 ±7		169 ±7		337 ±7
Informed consent		x											
Eligibility confirmation		x											
Observation								Observation					
Clinical Assessment	Physical examination	x		x	x	x	x		x		x		x
	Blood pressure, Pulse rate, Body temperature	x		x	x	x	x		x		x		x
	Height, Body weight	x									x*4		x*4
	OMDS	x		x	x	x	x	x	x	x	x	x	x
	Walking tests *A	x		x	x	x	x*2	x	x	x	x	x	x
	Walking aids *B	x		x	x	x	x	x	x	x	x	x	x
	MAS	x		x	x	x	x		x		x		x
	IPEC1	x		x	x	x	x		x		x		x
	VAS *C	x		x	x	x	x		x		x		x
	QOL*D	x		x	x	x	x		x		x		x
	Urinary dysfunction*E	x		x	x	x	x		x		x		x
Blood tests*F/ Urinalysis*G		x		x	x	x	x				x		x

Non progressor（Accompanying Research）

Period		Screening	1st Enrollment*1	Progressor Assessment				Observation				Observation	
Week		-16 ～ - 12*1		-12*1	-8	-4	Last assessment*2 ±7	(x)*3	12	(x)*3	24	(x)*3	48
Day		-112～-84 ±7		-84 ±7	-56 ±7	-28 ±7			85 ±7		169 ±7		337 ±7
Virus tests *7*H		x*5											
Pregnancy test *6		x											
Cerebrospinal fluid tests *7 *I		x*8					(x)				(x)		(x)
Accompaning research	Blood tests *J	x		x	x	x	x				x		x
	Biobank samples (Blood)*9	x		x	x	x	x				x		x
	Cerebrospinal Fluid tests *K	x*8					(x)				(x)		(x)
	Biobank samples (Cerebrospinal fluid) *9	x*8					(x)				(x)		(x)
MRI		x*10		x*10							x		x
Intraocular pressure		x*10		x*10									

*1: The progressor assessment period on Week -12 and screening can be performed on the same day. In this case, the items specified in the screening should be performed. In addition, the primary enrollment should occur within 7 days of screening.

*2: The day after the implementation date of the walk test in the final assessment is set as Day 1 for non-progressor.

*3: Monthly visits are allowed to confirm the extent of disease progression. If a subject has a visit, the subject will be evaluated for the Osame's Motor Disability Score, the walk test (10-meter timed walk, 6-minute walk distance, 2-minute walk distance, and timed up-and-go test), and the use of walking aids (at clinic and home)

*4: Height measurement is not required.

*5: HBs antigen, HBs antibody, HBc antibody, HCV antibody, HIV-1 antibody and HIV-2 antibody will be tested only at screening. If either HBs antibody or HBc antibody is positive, quantitative HBV-DNA measurement will be performed.

However, quantitative HBV-DNA is not required if the positive HBs antibody is clearly due to vaccination.

Non progressor（Accompanying Research）

*6: It is not required for subjects who are permanently sterilized. (Permanent sterilization: postmenopausal [with no menses for at least 48 weeks with no other medical reason], post sterilization [hysterectomy, bilateral salpingectomy, bilateral oophorectomy], tubal occlusion without tubal ligation)

*7: If data is available from a previous test on anti-HTLV-1 antibody for diagnosis of HAM, that data may be used.

*8: Data obtained within 12 weeks prior to the date of consent may be used.

*9: Residual samples will be used as samples for biobanking.

*10: MRI and intraocular pressure measurement will be performed at any visit from screening to the progressor assessment period and the data will be used as the baseline value of non progressor. For MRI scans, data obtained within 12 weeks prior to the date of informed consent can be used.

However, if an image used as the baseline shows findings suggestive of inflammation in the spinal cord, a retest must be performed within the progressor assessment period for that site only. In addition, if data obtained within 12 weeks prior to the date of informed consent at another hospital is available and indicates no findings of inflammation in the spinal cord, a retest is not required.

*A：Waling tests (10 meter Timed Walk, 6 minute Walk Distance, 2 minute Walk Distance, Timed Up and Go Test)

*B：Ambulatory aid used at the clinic and home

*C：VAS（Overall, Walking, Lower extremity pain）

*D：QOL (Modified IPEC2, N-QOL, Sexual Health Inventory for Men）

*E：Urinary dysfunction (OABSS, ICIQ-SF, IPSS)

*F：Blood tests： Complete blood count with differential, Urea nitrogen and electrolytes (BUN, Cre, Na, K), Liver function (AST, ALT, ALP, T-Bil),
Glucose metabolism (HbA1c (NGSP), Fasting blood glucose), Bone metablism (Ca, P), Lipids (T-Cho, LDL-C, HDL-C, TG)

*G：Urinalysis: glucose, protein

*H：Virus tests: Hepatitis B virus (HBc antibody ((at screening only), HBs antibody (at screening only), HBs antigen (at screening only), HBV-DNA)
HCV antibody (at screening only), HIV-1 antibody･HIV-2 antibody (at screening only)
Anti-HTLV-1 antibody for HAM diagnosis (at screening only)

*I：Cerebrospinal fluid tests: Anti-HTLV-1 antibody for HAM diagnosis (at screening only)
Safety: Glucose
Efficacy: Cell counts/ Cell fractionation, Toral protein, neopterin

Non progressor（Accompanying Research）

*J：Blood tests (Accompanying research): β2-microgloblin, B- and T-cell subsets/markers、sIL-2R、HTLV-1 proviral load, sVCAM-1、SPARC

*K：Cerebrospinal fluid tests (Accompanying research): HTLV-1 anribody, HTLV-1 proviral load, CXCL10, sVCAM-1

Non progressor（Accompanying Research）

15. Management of Patients（Non）

15.1 Notification to Other Departments/Hospitals

The principal investigator or subinvestigator will confirm whether the patient will visit other departments or hospitals before the start of administration of the study drug. If the patient has already visited another department or hospital, the principal investigator or subinvestigator will notify the patient's primary care physician of their participation in the study. If the patient uses any drugs other than those prescribed by the principal investigator or subinvestigator, the name of the drug(s) and the status of usage will be investigated.
If the patient newly visits another department or hospital during the study, the principal investigator or subinvestigator will take the same action as above.

15.2 Notification to Patients

The principal investigator, subinvestigator, or study collaborator will give the following instructions to patients:

＜Visits＞

In principle, the progressor assessment period, the treatment period of study drug, and the observation period will be performed on an outpatient basis. During visits, subjects will undergo the prescribed tests in accordance with the explanations and instructions of the Principal Investigator, etc.

For any abnormality, the principal investigator, etc. must be contacted and the subject must visit the hospital at any time.

16. Evaluation（Non）

16.1 Exploratory investigation

The biomarkers and clinical indicators will be used to explore prognostic and progressive disease categorical factors.

17. Data Reporting Method（Non）

17.1 Target for Preparation of Case Report Forms

Case report forms shall be created for all enrolled subjects.

17.2Preparation and Storage of Case Report Forms

In this study, case report forms shall be prepared and submitted in the EDC system called eCB.
After the completion of specified tests/observations, the principal investigator shall promptly enter data and sign on the eCB. If the subinvestigator or study collaborator completes data entry, the principal investigator shall review and check the details and then sign the form. For the method of input and correction, follow the guide which will be separately prepared.
If the study collaborator enters data into the case report form, only the extent that the data can be transcribed from the source documents is acceptable. The subinvestigators and study collaborators who can fill in the case report forms must be registered in the “List of subinvestigators and study collaborators”.
After data lock, the principal investigator shall obtain and store the CD-R containing the input data (including the correction history) from the data center.

17.3Identification of Source Documents

The source documents in this study are as follows:

- Medical records
- Diagnostic imaging films
- List of names for screening
- Informed consent forms
- Study drug accountability log
- Output form for general laboratory tests result
- QOL questionnaire, VAS questionnaire, and questionnaire for assessment of dysuria
- Clinical Efficacy Endpoint Assessment Form

17.4Procedures for Handling Missing, Unused, and Abnormal Data

For missing, unused, and abnormal data not expected before the start of the study, the principal investigator and the statistician will discuss and decide how to handle the data. Data handling details will be described in the analysis reports.

18. Adverse Event Reporting（Non）

Not applicable because this is an accompanying research without intervention.

Non progressor（Accompanying Research）

19. Statistical Analysis（Non）

19.1 Sample size calculation and power justification

Five subjects

No intervention for non-progressor will be performed in this study. Observation is set as the objective to allow rapid enrollment in the slow progressor or the rapid progressor. Based on the clinical experience, the target samples size was set to be the expected maximum number of subjects to be enrolled during the study. After reaching to the target sample size of 5 subjects, subject enrollment will be continued until the target sample size of 40 in slow progressors has been reached or until the end of enrollment period (2 years after the start of the study).

19.2Methods of analysis

19.2.1 Definitions of data sets analyzed

A population of all patients who are enrolled as non-progressors.

19.2.2 Analysis of Endpoints

Descriptive statistics will be calculated for the 10mWT and log 10mWT by timepoint to assess the natural history of HAM patients who are non-progressor, and descriptive statistics for the 10mWT and log 10mWT by timepoint for changes from the start of observation (=final assessment) will be calculated as well. In addition, the number and percentage of subjects whose disease progression category was changed to slow or rapid progressor during the observation period will be calculated, and background information will be separately summarized for the patient population with and without a change in the disease progression category.

20. Accompanying Research

20.1 Accompanying Research using collected samples

20.1.1 Accompanying Research Objective

The main objective of the study is to understand the duration of effect of methylprednisolone and prednisolone treatment, influences and effects on the antiviral immunity and immune system of subjects, and prognostic and progressive disease categorical factors of clinical exacerbation using samples (blood and spinal fluid collected) of subjects who have participated the study and consented to provide samples for the accompanying research using collected samples.

20.1.2 Consent from Patients

This accompanying research will be conducted as an exploratory examination in all patients who participated in the study and gave their consent to provide their samples to the accompanying research.

20.1.3 Test Parameters/Sample Processing Methods

Refer to Section 14. Observation, Investigations, Test Items, and Implementation Period.

20.1.4 Storage of Biological Specimens

Specimens will be stored at the Department of Rare Diseases Research, Institute of Medical Sciences, St. Marianna University School of Medicine.

20.1.5 Future Use of Samples

Details regarding the use of provided samples in future research will be separately specified before the conduct of research.

20.2 Secondary use of data

The data collected in this study may only be used for other research if the subject provided a consent and the use is approved by the Institutional Review Board during or after the study. (e.g. Comparison with results from other studies in patients with HAM, and integrated analysis with study data of the same drugs and similar therapies as this study drug).
In addition, if the data collected in this study alone provides insufficient information, the investigator will perform additional investigations based on the subject’s source documents, and will not place new burdens on the subject.

21. Ethical Considerations and Good Clinical Practice（GCP）

21.1 GCP and Compliance with the Study Protocol

This study will be conducted in accordance with the study protocol, the Pharmaceutical Affairs Law, the “Ministerial Ordinance on Good Clinical Practice for Drugs” (Ordinance of the Ministry of Health and Welfare No. 28, 1997; hereinafter referred to as “GCP”), the Ministerial Ordinance on the Partial Revision of the ordinance, and other related notifications in compliance with the declaration of Helsinki.

21.2 Review by the Institutional Review Board

- Prior to conducting the study, the IRB at the study site shall obtain the protocol, informed consent form, study drug summary document, and other materials required by the IRB, review the conduct and continuation of the study from the viewpoint of ethical, scientific, and medical validity, and notify the head of the medical institution of its opinion in writing. This study shall be conducted after review and approval by the Institutional Review Board.
- If the protocol or Informed Consent Form is changed during the study, the details of the changes shall be reviewed and the results shall be notified to the head of the medical institution in writing.
- If the study period exceeds 1 year, the appropriateness of continuing the study will be reviewed at least once a year and the results will be notified to the head of the medical institution in writing.

21.3 Subject Responsibilities

- The principal investigator, etc. shall explain the appropriate usage of the study drug to the subject, and confirm whether the subject is using the study drug properly as necessary.
- If a subject is being treated by another physician, the principal investigator, etc. shall inform the physician, with the consent of the subject that the subject will participate in this study.
- The head of the study site and the principal investigator, etc. shall take necessary steps in advance to ensure that appropriate medical care will be provided to subjects for adverse events that occur.
- The principal investigator, etc. will notify the subject if an adverse event occurs and treatment is deemed necessary.

21.4 Subject Privacy

The principal investigator, etc. shall pay due consideration to the protection of the privacy of subjects. Subject identification shall be done using a subject identification code, and the subject's private information shall not be entered in the case report form. In addition, in the event that the results of the study are published for academic purposes, etc., consideration shall be given to the protection of subjects' privacy.

21.5 Conflict of Interest (COI) in the Study

Any "conflict of interest in this study" of the research representative in the study, as well as the principal investigator and the study doctor at each study site, will in principle be reviewed/approved by the Conflict of Interest Review Committee or the Ethics Committee specified by their affiliated institutions. The conflicts of interest of the person in charge of the Coordinating Center, the person in charge of the Data Center, and the statistician will be similarly reviewed and approved in accordance with the provisions of their affiliated organizations.

21.6 Preparation and Changes of Protocol

21.6.1. Preparation of Protocol

The coordinating investigator shall obtain information on the quality, efficacy, and safety of the study drug and coordinate opinions of the principal investigator among each study site to prepare the study protocol.

21.6.2. Changes to Protocol

If the coordinating investigator becomes aware of any information related to the quality, efficacy, and safety of the study drug or other important information required to appropriately carry out the study, he/she should revise the protocol as necessary.

The principal investigator, etc. must not deviate from or make any changes to the protocol without prior written agreement with the coordinating investigator and written approval from the Institutional Review Board based on prior review. However, this shall not apply in the event of an unavoidable medical situation, such as to avoid an imminent hazard to a subject, or in the event of changes to administrative matters of the study only.

21.6.3. Deviations From the Protocol

If the principal investigator deviates from or changes to the protocol for unavoidable medical

reasons in order to avoid immediate risks to subjects, the details and reasons for the deviation or change shall be submitted to the coordinating investigator and the head of the medical institution, and the IRB via the head of the medical institution as soon as possible, and approval shall be obtained from the IRB.
The principal investigator, etc. shall record all actions that deviate from the protocol.
(Report described in this section is not necessary for the accompanying research in non-progressor. However, all deviations should be documented.)

21.6.4. Amendments and Revision to the Protocol

Prior to implementation of protocol amendments, the changes to be implemented must be submitted to Data and Safety Monitoring Committee and the Institutional Review Board of the study site for approval.

The addition of supplemental explanations not applicable to the protocol amendments will be distinguished and handled as Memorandums to the protocol. The definitions of the terms and their handling are as follows:

1) Amendment
   A partial change to the protocol that poses a possible increase in risk to patients participating in the study or is related to the primary endpoint of the study. It requires approval from the Data and Safety Monitoring Committee and the Institutional Review Board of the study site (or the Contract Research Review Committee).
2) Revision
   A change to the protocol that poses no possible increase in risk to patients participating in the study or is not related to the primary endpoint of the study. It requires approval from the Institutional Review Board of the study site (or the Contract Research Review Committee). An application to the Data and Safety Monitoring Committee is not required.
3) Memorandum
   A supplemental explanation to the protocol that is not a change in the content of the protocol and distributed from the principal investigator/the 0761HAM Coordinating Center to parties related to the study for the purpose of changing of wording or calling attention in particular. Any type of form is acceptable. It will be submitted to the Institutional Review Board of the study site (or the Contract Research Review Committee) according to the regulations of the study site. An application to the Data and Safety Monitoring Committee is not required.

21.7 Study Discontinuation Criteria and Procedures

If there is no choice but to discontinue or interrupt the study based on the criteria shown below, the principal investigator will contact the head of the study site and the Pharmaceuticals and Medical Devices Agency. The head of the study site will promptly inform the Institutional Review Board of the relevant study site of this fact in written form and provide a detailed explanation.
The principal investigator will promptly give patients an explanation of the fact and take necessary actions.
＜Discontinuation Criteria＞
(1) If important information on the safety or efficacy of study drug administration is obtained in or outside Japan and the continuation of the study is determined to not be possible
(2) If a discontinuation or interruption of the study is recommended by the Data and Safety Monitoring Committee and the continuation of the study is determined to not be possible
(3) If there is no choice but to prematurely discontinue or interrupt the entire study due to other reasons

21.8 Compensation

When any unexpected serious health damage (which is not described in the KW-0761 Investigator’s Brochure and which includes death; the same shall apply hereinafter) caused by the study occurs in a patient, compensation will be provided to the patient according to the “Standard Operating Procedures for Compensation and Indemnity”. Compensation will be provided in the form of medical services.
The compensation principles shall not prevent the patient from claiming compensation for damage. If health damage occurs due to the study drug or any clinical interventions or procedures specified in the protocol and which would not have occurred if the patient had not participated in the study, the compensation provided to the patient will also take into consideration this probability. The principal investigator will have crinical trial insurance for compensation.

21.9 Payment of Money to Subjects (Compensation to Subjects)

Compensation to subjects to reduce burdens in this study shall follow the rules of the study site.

21.10 Research Registration

This study will be registered in the UMIN Clinical Trials Registry (UMIN-CTR) to make information on the plan, implementation, and results of the clinical study publicly available.
UMIN URL : http://www.umin.ac.jp/ctr/index-j.htm

22. Monitoring and Audits

Monitoring and audits will be conducted to confirm that the conduct of the study as well as the preparation, recording, and reporting of the data comply with the protocol, GCP, and other related notifications.

When monitoring, audits, or investigations by regulatory authorities are conducted, the study doctor and the study site must provide direct access to all study-related records, such as source documents.

22.1. Monitoring

In order to ensure the proper conduct of the study, the principal investigator will entrust monitoring tasks to a third party outside the study site to ensure that the conduct of the study as well as recording and reporting of the data are implemented in compliance with the GCP, protocol, and procedures. The entrusted party (the monitor) will perform monitoring according to the separately specified Standard Operating Procedures.

22.1.1. Responsibilities of the Monitor

Based on the results of monitoring, if it is confirmed that this study is not being conducted in compliance with ministerial ordinances or the protocol at the study site, the monitor must promptly inform the principal investigator of this fact.

When conducting monitoring, the monitor must submit a monitoring report describing the items below to the principal investigator and the head of the study site for each monitoring instance.

(1) Date and time of monitoring
(2) Name of the monitor
(3) Name of the principal investigator, etc. from whom an explanation was received during monitoring
(4) Summary of the monitoring results
(5) Matters conveyed to the principal investigator
(6) Measures to be taken based on the monitoring results and the monitor’s opinions on those measures

22.2. Auditing

In order to guarantee that the conduct of the study as well as the recording and analyzing of data is conducted according to the protocol, the principal investigator will entrust site-visit

audits to a third party outside the study site. The entrusted external auditor will visit the study site and perform reviews of IRB-approved documents and informed consent forms for patients as well as cross-referencing of the data on case report forms with medical records (source data verification), etc. according to the audit plan and the standard operating procedures prepared by the person responsible for the task based on the plan.

23. Efficacy and Safety Evaluation Committee

If the opinion is requested regarding serious adverse events and other safety issues from the coordinating investigator, the Efficacy and Safety Evaluation Committee shall review the relevant event in accordance with the “Standard Operating Procedure for Review by the Efficacy and Safety Evaluation Committee” separately established, and advise/recommend the results to the coordinating investigator in writing.

An “Efficacy and Safety Evaluation Committee” independent of coordinating investigator, principal investigator, and subinvestigators shall be established to secure the safety of subjects and conduct appropriate and scientific studies.

In accordance with the “Standard Operating Procedures for the Efficacy and Safety Evaluation Committee” separately established, the Efficacy and Safety Evaluation Committee will consult upon the occurrence of new Drug Safety Information and upon changes to the protocol, and provide advice and recommendations in writing to the coordinating investigator.

24. Retention of Records

24.1. Retention at the Study Site

The head of the study site and the founder of the Institutional Review Board will retain the study-related documents and records specified in the GCP, such as records concerning patient consent, records used as a basis for preparing CRFs (medical records, test slips, etc.), records of IRB reviews, the study drug storage/accountability log, and the report on the completion of the study, until the later of the dates below. However, the retention period can be extended at the discretion of the principal investigator.

1）The date of marketing approval for the pharmaceutical product related to the test drug
2）The date on which three years have elapsed from the date of discontinuation of development of the test drug or that of the discontinuation or completion of the study

24.2. Storage of source documents for outsourced testing

The source documents on the cerebrospinal fluid concentration of neopterin will be stored as specified below. The records, etc. attesting to accuracy control, etc. in tests will be stored for 10 years from the date of receipt of the samples.

(Storage location) SRL, Inc.

(Responsible person) Yoji Hirabayashi

2-1-1 Nishishinjuku, Shinjuku-ku, Tokyo 163-0409

TEL：+81-3-6279-0900　FAX：+81-3-6279-0971

24.3. Storage of Biological Specimens

The samples used for measuring the cerebrospinal fluid concentration of neopterin will be stored until the preparation of the final report at SRL Medisearch Inc. Subsequent handling of the samples will be separately discussed.

25. Research Costs for the Study

The major source of funds for conducting this clinical study will be received from the Practical Research Project for Rare/Intractable Diseases of the Japan Agency for Medical Research and Development

26. Attribution and Publication of Research Results

The results of this study will belong to Yoshihisa Yamano, Study Coordinator, St. Marianna University Graduate School of Medicine.

In addition, the results of the study will be made public at academic meetings and in academic journals in and outside Japan. In the event of publication, prior notification shall be given to the above person having the rights of ownership, and the principal investigators of each study site and the study drug provider.

27. Research Structure (Japan)

This clinical study is a multicenter, investigator-initiated study.

(1) Chief Investigator

Yoshihisa Yamano

Department of Rare Disease Research, Institute of Medical Science, St. Marianna

University School of Medicine.
Address: 2-16-1, Sugao, Miyamae-ku, Kawasaki, 2168511, Japan
Telephone: +81-44-977-8111
Fax +81-44-977-9772
E-mail：yyamano@marianna-u.ac.jp

(2) Study Site/ Primary Investigator

St. Marianna University School of Medicine, Kawasaki, Japan/ Yoshihisa Yamano
Kyoto Prefectural University of Medicine Graduate School of Medical Science, Kyoto, Japan/ Masanori Nakagawa
Fukuoka University, Fukuoka, Japan/ Yoshio Tsuboi
Kagoshima University Graduate School of Medical and Dental Sciences, Kagoshima, Japan/ Eiji Matsuura
University of the Ryukyus, Okinawa, Japan/ Hirokuni Sakima

(3) Statisticians

＜In charge of statistical analysis＞
Kenichiro Tanabe
Department of Frontier Medicine, Institute of Medical Science, St. Marianna University School of Medicine
Address: 2-16-1, Sugao, Miyamae-ku, Kawasaki, 2168511, Japan
Telephone: +81-44-977-8111
Fax +81-44-977-8166
E-mail： kenichiro.tanabe@marianna-u.ac.jp

＜Statistical Analysis Advisor＞
Eisuke Inoue
Showa University Research Administration Center
Address: 1-5-8, Hatanodai, Shinagawa-ku, Tokyo, 142-8555, Japan
Telephone: +81-3-3784-8000
E-mail：eisuke.inoue@med.showa-u.ac.jp

(4) HAMLET-P Coordinating Center, Enrollment Center, and Data Center

(1) Coordinating Center

【HAMLET-P coordination center】

[Before September 30, 2017]
Kanagawa Institute of Industrial Science and Technology (KISTEC)
Global health research coordinating center
E-mail：hamlet-p@newkast.or.jp

[After October 1, 2017]
Clinical research data center, St. Marianna University
E-mail：mariadc_ham@marianna-u.ac.jp

(2) Enrollment Center and Data Center

Kenichi Kawano
Division of Health Data Science, Translational Research Center for Medical Innovation.
Address: 1-5-4, Minatojimaminamimachi, Chuo-ku, Kobe, 650-0047, Japan.
Telephone: +81-78-303-9116
FAX: +81-78-303-9117
E-mail：hamlet-p-dc@tri-kobe.org

(5) Efficacy and Safety Evaluation Committee

Mitsuharu Osame
President, Public Interest Incorporated Foundation Jiaikai
Masaaki, Niino
Director, Devision of Clinical Research, Hokkaido medical center
Masahiro Nagai
Professor, Clinical Research Center, Ehime University Hospital

(6) Contracted Organization for Monitoring

Atsuhiko Kawamoto
Division of Monitaring, Translational Research Center for Medical Innovation.
Telephone: +81-78-303-9104
FAX: +81-78-303-9094
E-mail: kawamoto@fbri.org

(7) Contracted Organization for Audit Duties

Refer to Appendix.

(8) Contracted Organization for the Preparation of Clinical Study Reports

Refer to Appendix.

(9) Study Drug Provider

Nipro Pharma Corporation
Address: 2-2-7, Dosho-machi, Chuo-ku, Osaka, 541-0045, Japan
Telephone: +81-6-6231-9850

(10) Supplier of Storage and Shipping of Study Drug

CHUOUNYU CO., LTD.
Address: 10-2, Nihonbashiodenma-cho, Chuo-ku, Tokyo,103-0011, Japan.
TEL: +81-3-3661-7743
<Storage warehouse>
CHUOUNYU CO., LTD., Kazo sales office
Address: 37-3, Kitatsuji, Kazo, 347-0023, Japan.
TEL: +81-480-66-2330

(11) CXCL10 Measurement Kit Provider

Cosmic Corporation Co., Ltd.
Address: Tomisaka Building, 7-3 Koishikawa 2-chome, Bunkyo-ku, Tokyo, 112-0002, Japan.
TEL: +81-3-5802-5880

(12) Key Controller Contract Organization

DOT WORLD Co., Ltd.
Address: Comodio Shiodome 4F, 2-14-1, Higashi-Shimbashi, Minato-ku, Tokyo, 105-0021, Japan.
TEL: +81-3-3433-6155 FAX: +81-3-3433-6156

28. Rationale

28.1. Study Design

The primary objective of this study is to provide evidence of the efficacy and safety of corticosteroid therapy depending on the degree of disease progression based on previous open-label clinical studies and clinical experience with corticosteroids in patients with HAM,

as well as events observed in the nonclinical studies for HAM. As the clinical course of HAM is thought to be broadly classified into three categories of rapid progression, slow progression, and non-progression, this study is designed to assign the patients into three groups of rapid progressor, slow progressor non-progressor to allow the study treatment and observation depending on the characteristics of the clinical course. In addition, by establishing an observation period as a progressor assessment period after primary enrollment and the non-progressor group without therapeutic intervention, the study allows to follow up on the Natural History of symptomatic deterioration and investigate indicators such as biomarkers that correlate with disease progression.
Due to the fact that it is ethically difficult to establish a placebo group in rapid progressors due to the high need for treatment, the study in rapid progressors was designed as a prospective, randomized, open, blinded-endopoint study, while the study in slow progressors was designed as a randomized, double-blind, placebo-controlled comparative study.

28.2. Efficacy Evaluation Criteria

28.2.1. 10 meter walk test

The reasons why the 10 meter walk test (10mWT) was selected as the clinical evaluation indicator were that it is the sole clinical evaluation indicator for HAM/TSP that is evaluable as continuous numeric data and has superior sensitivity/quantification as an indicator to capture clinical changes, as well as the fact that it has previously been used as an evaluation indicator in clinical studies in HAM/TSP patients. The analysis of the data on the 10mWT in 76 HAM/TSP patients collected from 4 countries outside Japan showed that the log-transformed values of the 10mWT are normally distributed and proved to be able to withstand biostatistical analysis of the efficacy evaluation.

28.2.2. 6 minute Walk Distance, 2 minute Walk Distance, and Time Up and Go

In addition to the 10 mWT, the 6-minute walk distance, 2-minute walk distance, and timed up-and-go tests were used for multidimensional and quantitative assessment of gait disturbances, which are the primary symptoms of HAM. The 6-minute walk distance and 2-minute walk distance tests also have characteristics that can evaluate the endurance of walking, and particularly in mild patients, their sensitivity is excellent as an indicator of clinical change. The timed up-and-go test is also characterized by being able to assess the balance of walking.

28.2.3. Other Clinical Evaluations

The clinical evaluation indicators that have been used in the clinical studies in HAM/TSP

patients, such as the Osame Motor Disability Score, IPEC1, Modified IPEC2, Modified Ashworth scale, presence/absence of lower extremity clonus, walking, lower extremity pain and overall evaluation using a VAS by patient, were selected to perform the multi-angled evaluations/observations by evaluating and recording the data obtained from those indicators. Other than the N-QOL, which is used internationally as an evaluation indicator for urination disorders to understand changes in the symptoms caused by neurogenic bladder and whose usability has been recognized in HAM/TSP patients based on our experience, I-PSS (evaluation of urinary retention), OABSS (Overactive Bladder Symptom Score), and ICIQ-SF (International Consultation on Incontinence Questionnaire-Short Form) were used

28.2.4. Efficacy evaluation period

In order to determine the treatment duration in this study, data analysis was performed on 14 Japanese HAM patients who were treated with prednisolone in daily medical practice. This data is the same as that in Data set A used for design of sample size. The time course of 10-meter timed walk (logarithmically transformed) in these subjects is shown in Figure 6. The red line in the diagram represents the mean and standard error, while the other lines represent individual patient measurements. Missing values were imputed with the latest measurement prior to the timepoint (LOCF). In addition, the change (%) at Months 1, 3, 6, 9 and 12 relative to the values before prednisolone treatment (values at Month 0) is presented in Table 5. These tables and figures show that the effects of prednisolone appear 1 to 3 months after the start of administration. In addition, patients who responded to treatment by Month 6 can still be seen to have maintained the treatment effect. The group means also support these results. This is a placebo-controlled study and, given the definition of slow progressor, the control group is expected to include patients with worsening by 10 to 30% in 3 months, and it is not clinically preferable to continue placebo treatment for at least 6months.

Based on the above, it was considered most desirable to assess the efficacy of prednisolone at Month 6.

Table 5. Change (%) in the 10 meter timed walk in HAM patients treated with prednisolone

	Month 1	Month 3	Month 6	Month 9	Month 12
No imputation	8.1	10.8	15.7	16.7	16.0
LOCF	9.0	10.8	13.8	11.4	15.1

Figure 6.　Time course of logarithmically transformed 10-meter timed walk in HAM patients treated with prednisolone. Missing data were imputed by the LOCF.

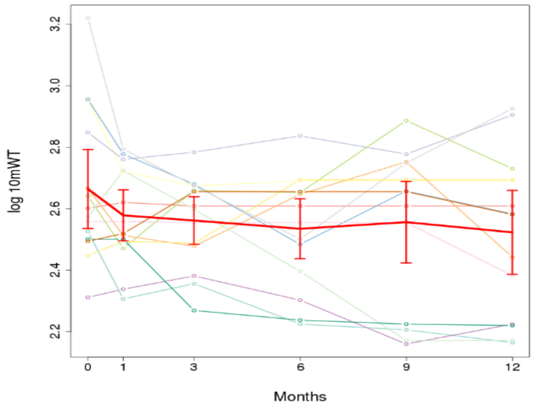

28.2.5. Primary Endpoint in Rapid Progressor

The criteria for the rapid progressor is a worsening of ≥30% in the 10-meter timed walk or a ≥Grade 1 worsening of the OMDS within 12 weeks. When methylprednisolone therapy is administered to this population, the clinical expectation is whether the condition can be restored to the state before it deteriorated, and if this criteria is met, the treatment is determined to be successful. Therefore, the primary endpoint of this study was defined.

28.3. Eligibility Criteria

(1) Mandatory diagnostic parameters for eligible HAM/TSP patients were clarified to meet the study objectives.

(2) Considering the ability to consent and ethics, the lower limit of age was set to be 18 years in accordance with the eligibility criteria for the overseas study in the sister trial. For minors, the consent of a legal representative was also required. Because patients with HAM are relatively elderly, there is no age limit provided that eligibility criteria are met.

(3) This was selected as the condition of eligible patients to evaluate the efficacy of the study drug.

(4) It was set as the condition of patients with adequate organ function (bone marrow function, liver function, kidney function and glucose tolerance).

(5) This was selected because this is a clinical study conducted in compliance with GCP.

(6) This was selected as the condition of eligible patients to evaluate the safety and efficacy of the study drug.

28.4. Exclusion Criteria

(1) (2) These were selected to ensure the safety of patients and exclude any unqualified patients for the safety evaluation of the study drug.
(3) This was selected to ensure the safety of patients and exclude any unqualified patients for the safety evaluation of the study drug.
(4) This was selected to eliminate the effects of other study drugs.
(5) This was selected to ensure the safety of patients and exclude any unqualified patients for the safety evaluation of the study drug.
(6) This was selected to exclude any unqualified patients for the efficacy evaluation of the study drug.
(7) ～(18) These were selected to ensure the safety of patients and exclude any unqualified patients for the safety evaluation of the study drug.
(19) (20) This was selected to exclude any unqualified patients for the efficacy evaluation of the study drug.
(21) It was set to exclude patients inappropriate for the safety assessment of the study drug due to concerns about the induction and exacerbation of osteoporosis caused by the study drug.
(22) This was selected to exclude any unqualified patients for the safety and efficacy evaluation of the study drug.
(23) ～(28) This was selected to exclude any unqualified patients for the safety evaluation of the study drug.
(29) This was selected to exclude patients considered unqualified to participate in the study by the principal investigator or subinvestigator due to other reasons.

28.5. Treatment Plan

28.5.1 Dose, Method and Period of Administration

(1) **Rapid progressor**

It was set based on the literature on methylprednisolone treatment/prednisolone treatment for HAM and the “HAM treatment manual” prepared by the Ministry of Health, Labour and Welfare research group. This is the dosage, administration method, and treatment period that have been used frequently so far, and it was considered valid from the perspective of safety.

(2) **Slow progressor**

It was set based on the literature on methylprednisolone treatment/prednisolone treatment

for HAM and the "HAM treatment manual" prepared by the Ministry of Health, Labour and Welfare research group. This is the dosage, administration method, and treatment period that have been used frequently so far, and it was considered valid from the perspective of safety.

28.5.2 Dose increase criteria

(1) **Methylprednisolone additional treatment criteria**

There have been many reports of efficacy of steroid therapy for HAM, and in terms of subject safety, dose increase was allowed if clinically evident deterioration was observed. The clinical deterioration of grade 1 or more in the Osame's Motor Disability Score (OMDS) can be regarded as a clinically obvious deterioration, and the data analysis of in patients with HAM showed that the 10mWT values varied approximately 60% by a difference by 1 grade in the OMDS and that the 10mWT in patients with clinically stable HAM varied within ±10%. Therefore, this study defined a worsening of 30% or more to identify an apparent clinical deterioration in a short period.

(2) **Criteria for additional oral prednisolone treatment**

There have been many reports of efficacy of steroid therapy for HAM, and in terms of subject safety, dose increase was allowed if clinically evident deterioration was observed. Since the clinically stable HAM patients demonstrated a variation within the range of ±10% in the 10mWT, this study defined 2 worsenings of 10% or more to identify an apparent clinical deterioration while taking prednisolone.
However, in rapid progressors who reduce the dose of/discontinue prednisolone after Week 24, clinical rescue is highly necessary to perform if the 10 mWT worsens by 10% or more by Week 28, so resumption of oral prednisolone at the dose at Week 24 was permitted.

28.6. Study Discontinuation Criteria

[Rapid progressor]
①、②、③、⑤、⑥ These were selected in consideration of the safety of patients.
④ This was selected in consideration of the ethicality for patients.
⑦ This was selected to place importance on medical judgments made by the principal investigator or subinvestigator.

[Slow progressor]
①、②、③、④、⑥ These were selected in consideration of the safety of patients.
⑤ This was selected in consideration of the ethicality for patients.

⑦ This was selected to place importance on medical judgments made by the principal investigator or subinvestigator.

[Non progressor]
① This was selected in consideration of the ethicality for patients.
② This was selected in consideration of the safety of patients.
③ This was selected to place importance on medical judgments made by the principal investigator or subinvestigator.

28.7. Concomitant Drugs

28.7.1. Prohibited Concomitant Drugs

[Rapid progressor] [Slow progressor]
①～③、⑤～⑨ These were selected because these drugs are considered to affect the efficacy and safety evaluation.
④This was selected because these drugs are considered to affect the efficacy evaluation.
[Non progressor]
①～④、⑥～⑨ These were selected because these drugs are considered to affect the efficacy and safety evaluation.
⑤This was selected because these drugs are considered to affect the efficacy evaluation.

28.7.2. Drugs requiring caution for concomitant use

Thses were selected based on the drug information in consideration of the safety of patients

28.7.3. Allowed Concomitant Drugs and therapies

＜Recommended Concomitant Drugs＞
1)、2)Thses were selected in consideration of the safety of patients.
＜Other Allowed Concomitant Drugs＞
1)～7)Thses were selected in consideration of the safety of patients.
＜Allowed Concomitant Therapies＞
1)This was selected in consideration of the safety of patients.

28.8. Accompanying research using samples collected

The B- and T-cell subsets/markers will be analyzed to understand the influences and

effects of the study drugs on the immune system in patients with HAM. In order to analyze the influences of the study drug on HTLV-1 infection, the quantitative HTLV-1 provirus in peripheral blood and spinal fluid will be measured. In addition, serum sIL-2R concentration, beta-2 microglobulin concentration, cerebrospinal fluid concentration of CXCL10 and neopterin, and anti-HTLV-1 antibody titer will be measured in order to analyze improvements in systemic and central nervous system inflammation in HAM.

28.9. Assessment of Safety

It was set to assess the influences of the study drug on the body.

29. References

1. Bangham C, Taylor G, Yamano Y, Araujo A. HTLV-I-Associated Myelopathy/Tropical Spastic Paraparesis (HAM/TSP). Nature Reviews Disease Primers, 1: 1-16, 2015.

2. 高田礼子、八木下尚子、鈴木弘子. HAM 患者登録システム(HAM ねっと)を用いた HAM の疫学的解析. 厚生労働科学研究委託費難治性疾患等克服研究事業(難治性疾患等実用化研究事業(難治性疾患実用化研究事業))「HAM の革新的な医薬品等の開発促進に関する研究」平成 26 年度委託業務成果報告書, 2014. (Japanese)

3. Martin, F., Fedina, A., Youshya, S., and Taylor, G.P. (2010). A 15-year prospective longitudinal study of disease progression in patients with HTLV-1 associated myelopathy in the UK. J. Neurol. Neurosurg. Psychiatry 81, 1336-1340.

4. Olindo, S., Cabre, P., Lézin, A., Merle, H., Saint-Vil, M., Signate, A., Bonnan, M., Chalon, A., Magnani, L., Cesaire, R., and Smadja, D. (2006). Natural history of human T-lymphotropic virus 1-associated myelopathy: A 14-year follow-up study. Arch. Neurol. 63, 1560-1566.

5. Nakagawa, M., Izumo, S., Ijichi, S., Kubota, H., Arimura, K., Kawabata, M., and Osame, M. (1995). HTLV-I-associated myelopathy: analysis of 213 patients based on clinical features and laboratory findings. J. Neurovirol. 1, 50-61.

6. Gotuzzo, E., Cabrera, J., Deza, L., Verdonck, K., Vandamme, A.M., Cairampoma, R., Vizcarra, D., Cabada, M., Narvarte, G., and De las Casas, C. (2004). Clinical characteristics of patients in Peru with human T cell lymphotropic virus type 1-

associated tropical spastic paraparesis. Clin. Infect. Dis. 39, 939-944.

7. Sato T, Coler-Reilly A, Utsunomiya A, Araya N, Yagishita N, Ando H, Yamauchi J, Inoue E, Ueno T, Hasegawa Y, Nishioka K, Nakajima T, Jacobson S, Izumo S, Yamano Y. CSF CXCL10, CXCL9, and Neopterin as Candidate Prognostic Biomarkers for HTLV-1-Associated Myelopathy/Tropical Spastic Paraparesis. PLoS Negl Trop Dis., 7(10): e2479, 2013.

8. Osame M, Matsumoto M, Usuku K, Izumo S, Ijichi N, Amitani H, Tara M, Igata A. Chronic progressive myelopathy associated with elevated antibodies to human T-lymphotropic virus type I and adult T-cell leukemialike cells. Ann Neurol. 21(2):117-22, 1987.

9. Osame, M., Igata, A., Matsumoto, M., Kohka, M., Usuku, K., and Izumo, S. (1990). HTLV-I-associated myelopathy (HAM) treatment trials, retrospective survey and clinical and laboratory findings. Hematol. Reviews 3, 271-284.

10. Nakagawa, M., Nakahara, K., Maruyama, Y., Kawabata, M., Higuchi, I., Kubota, H., Izumo, S., Arimura, K., and Osame M. (1996). Therapeutic trials in 200 patients with HTLV-I-associated myelopathy/tropical spastic paraparesis. J. Neurovirol. 2, 345-355.

11. Croda, M.G., de Oliveira, A.C., Vergara, M.P., Bonasser, F., Smid, J., Duarte, A.J., and Casseb, J. (2008). Corticosteroid therapy in TSP/HAM patients: The results from a 10 years open cohort. J. Neurol. Sci. 269, 133-137.

12. HAM 診療マニュアル（第 2 版），厚生労働科学研究費補助金難治性疾患等政策研究事業「HAM 及び HTLV-1 関連希少難治性疾患の実態調査に基づく診療指針作成と診療基盤の構築を目指した政策研究班」2015. (Japanese)

13. 佐藤知雄, HAM 患者を対象としたステロイド治療の長期的効果に関する後ろ向き研究，厚生労働科学研究費補助金難治性疾患等実用化研究事業（難治性疾患実用化研究事業）「HAM の革新的な治療法となる抗 CCR4 抗体療法の実用化に向けた開発」平成 25～26 年度総合研究報告書, 2014. (Japanese)

30. Appendix 1

<Ambulatory aid used at the clinic (= outdoors during a clinic visit)>

Type of walking aid used at the site visit and any changes from the previous visit will be collected.

Type of aid......................None / 1 / 2 / walker / unable to walk

Type of cane ....Right: Single-tip cane / Tripod cane / Quad cane, Left: Single-tip cane / Tripod cane / Quad cane

Changed walk aids since last visit? .... Yes / No

<Ambulatory aid used at home (= indoors)>

Type of walking aid used at home and any changes from the previous visit will be collected.

Type of aid......................None / 1 / 2 / walker / unable to walk

Type of cane ....Right: Single-tip cane / Tripod cane / Quad cane, Left: Single-tip cane / Tripod cane / Quad cane

Changed walk aids since last visit? .... Yes / No

[Notes for assessment of walking aid]

- For “type of aid”, “1” indicates 1 cane and “2” indicates 2 canes.
- “Type of cane” shall be collected only when “1” or “2” was chosen in “type of aid”
- If the type of aid used by a subject is not a cane or walker, the study doctor shall determine the “type of aid” that is consistent with the OMDS assessment on a flat floor.

＜Osame Motor Disability Scale＞

Grade	Motor disability	
**0**	Normal gait and running	
**1**	Normal gait but runs slowly	＊1
**2**	Abnormal gait (staggering or spastic)	＊1
**3**	Abnormal gait and unable to run	
**4**	Needs support while using stairs but walks without assistance	
**5**	Needs one hand support in walking	
**6**	Needs two hands support in walking (can walk more than 10 meter)	
**7**	Needs two hands support in walking (can walk less than 10 meter)	
**8**	Needs two hands support in walking (can walk less than 5 meter)	
**9**	Unable to walk but can walk on all fours	
**10**	Unable to walk on all fours but can crawl with hands	
**11**	Unable to crawl with hands but can turn sideways in bed	
**12**	Unable to turn sideways but can move the toes	
**13**	Completely bedridden (unable to move the toes)	

[Notes for the OMDS assessment in this study]

*1: To be rated as "Grade2"

＜IPEC1＞

MOTOR SCORE :

< Gait >

0	Normal
1	Abnormal but can walk independently
2	Abnormal and dependent on eventual unilateral support
3	Abnormal and dependent on permanent unilateral support
4	Abnormal and dependent on eventual bilateral support
5	Abnormal and dependent on permanent bilateral support
6	Abnormal, dependent on permanent bilateral support, and occasional use of a wheelchair (WC)
7	Permanent use of a WC, stands up, and remains upright without support
8	Permanent use of a WC, uses arms to stand up, and remains upright without support
9	Permanent use of a WC, needs assistance from others to stand up and remain upright with support
10	Permanent use of a WC, unable to stand up, exhibits voluntary movements of the lower limbs when seated
11	Permanent use of WC, unable to stand up, and does not have any voluntary movements of the lower limbs

< Running >

0	Able to run
1	Unable to run

< Climbing stairs >

0	Climbs stairs
1	Climbs only when holding the handrail
2	Unable to climb

< Jumping >

0	.Jumps on one or two feet ＊1
1	.Jumps on two feet, but not with only one
2	Jumps on two feet only with hand support
3	Unable to jump

SPASTICITY SCORE :

< Clonus >

0	Absent
1	Only induced by the examiner
2	Spontaneous

< Flexor / Extensor Spasms >

0	Absent
1	Present

SENSORY SCORE :

< Paresthesia > ＊2

0	Absent
1	Present, intermittently
2	Present, permanently

< Lumbar and / or lower limb pain >

0	Absent
1	Present, intermittently
2	Present, during most of the day

SPHINCTER SCORE :

< Bladder control >

0	Total
1	Urgency, intermittently
2	Occasional incontinence or retention
3	Use of permanent catheter or regular use of relieve catheter

< Bowel Continence >

0	Normal
1	Constipation
2	Incontinence or total retention, needs manual extraction or enemas

TOTAL SCORE (0-29) : ______

＊1：Only 'Jumps on one foot' is scored '0'.

＊2：e.g. numbness.

＜Modified IPEC 2＞

Please describe your physical well being over the last 14 days by using the below scoring system.

① Moving about

How difficult were the following activities for you?

	None	Minimal	Small	Moderate	A lot	Extreme	Unable
a. Raising from a chair	0	1	2	3	4	5	6
b. Staying upright for at least 5 minutes	0	1	2	3	4	5	6
c. Moving around inside your house	0	1	2	3	4	5	6
d. Moving around outside your house	0	1	2	3	4	5	6
e. Going up and down stairs	0	1	2	3	4	5	6

f. How frequently did you need support (persons, walking sticks, wheelchair) to do most of the activities above	Never	Rarely	Eventually	Frequently	Often	Always
	0	0.25	0.50	0.75	1	1.25

② Bladder and Bowel Motions:

How difficult has it been?

	None	Minimal	Small	Moderate	A lot	Extreme	Unable
1. Make it to the toilet in time?	0	1	2	3	4	5	6
2. Passing stool?	0	1	2	3	4	5	6

＜Modified Ashworth scale（MAS）＞

Place the patient in a supine position to examine muscle tone and score the tone accordingly:

< Scoring >

**0**	No increase in muscle tone
**1**	Slight increase in muscle tone, manifested by a catch and release or by minimal resistance at the end of the range of motion when the affected part(s) is moved in flexion or extension
**1+**	Slight increase in muscle tone, manifested by a catch, followed by minimal resistance throughout the remainder (less than half) of the ROM
**2**	More marked increase in muscle tone through most of the ROM, but affected part(s) easily moved
**3**	Considerable increase in muscle tone, passive movement difficult
**4**	Affected part(s) rigid in flexion or extension

＜OABSS＞

Please circle the score that best applied to your urinary condition during <u>last 7 days</u>.

**Question**	**Response**	**Score**
Q1. How many times do you typically urinate from waking in the morning until sleeping at night?	<= 7	0
	8-14	1
	>=15	2
Q2. How many times do you typically wake up to urinate from sleeping at night until waking in the morning?	0	0
	1	1
	2	2
	>=3	3
Q3. How often do you have a sudden desire to urinate, which is difficult to defer?	None	0
	＜once /wk	1
	>= once /wk	2
	About once/d	3
	2-4 times/d	4
	>= 5 times/d	5
Q4. How often do you leak urine because you cannot defer the sudden desire to urinate?	None	0
	＜once /wk	1
	>= once /wk	2
	About once/d	3
	2-4 times/d	4
	>= 5 times/d	5

＜ICIQ-SF＞

How often and how much you maybe have leaked urine, and how much this may have bothered you over the last four weeks?

1. How often do you leak urine? (Tick one box)

never □ 0

about once a week or less often □ 1

two or three times a week □ 2

about once a day □ 3

several times a day □ 4

all the time □ 5

2. How much urine do you usually leak (whether you wear protection or not)?

(Tick one box)

none □ 0

a small amount □ 2

a moderate amount □ 4

a large amount □ 6

3. Overall, how much does leaking urine interfere with your everyday life?

Please ring a number between 0 (not at all) and 10 (a great deal)

0 1 2 3 4 5 6 7 8 9 10

not at all a great deal

4. When does urine leak? (Please tick all that apply to you)

never-urine does not leak □

leaks before you can get to the toilet □

leaks when you cough or sneeze □

leaks when you are asleep □

leaks when you are physically active/exercising □

leaks when you have finished urinating and are dressed □

leaks for no obvious reason □

leaks all the time □

＜N-QOL＞

The following statements are about the impact of 'having to get up at night to urinate'.

For each item, please mark an (✔) in the box next to the response that best describes how you have felt. Please mark only one box for each statement.

Over the past 2 weeks, having to get up at night to urinate…

1 Has made it difficult for me to concentrate the next day

Every day	□	4
Most days	□	3
Some days	□	2
Rarely	□	1
Never	□	0

2 Has made me feel generally low in energy the next day

Every day	□	4
Most days	□	3
Some days	□	2
Rarely	□	1
Never	□	0

3 Has required me to nap during the day

Every day	□	4
Most days	□	3
Some days	□	2
Rarely	□	1
Never	□	0

4 Has made me less productive the next day

Every day	□	4
Most days	□	3
Some days	□	2
Rarely	□	1
Never	□	0

5 Has caused me to participate less in activities I enjoy

Extremely	□	4
Quite a bit	□	3
Moderately	□	2
A little bit	□	1
Not at all	□	0

6 Has caused me to be careful about when or how much I dring

All the time	□	4
Most of the time	□	3
Some of the time	□	2
Rarely	□	1
Never	□	0

7 Has made it difficult for me to get enough sleep at night

Every night	□	4
Most nights	□	3

Some nights	□	2
Rarely	□	1
Never	□	0

Over the past 2 weeks, I have been…….

8 Concerned that I am disturbing others in the house because of having to get up at night to urinate

Extremely	□	4
Quite a bit	□	3
Moderately	□	2
A little bit	□	1
Not at all	□	0
No family or others in the house	□	0

9 Preoccupied about having to get up at night to urinate

All the time	□	4
Most of the time	□	3
Some of the time	□	2
Rarely	□	1
Never	□	0

10 Worried that this condition will get worse in the future

Extremely	□	4
Quite a bit	□	3
Moderately	□	2
A little bit	□	1
Not at all	□	0

11 Worried that there is no effective treatment for this condition (having to get up at night to urinate)

Extremely	□	4
Quite a bit	□	3
Moderately	□	2
A little bit	□	1
Not at all	□	0

12 Overall, how bothersome has having to get up at night to urinate been during the past 2 weeks?

Extremely	□	4
Quite a bit	□	3
Moderately	□	2
A little bit	□	1
Not at all	□	0

13 Overall I would rate my quality of life to be…
Please ring a number between 0 (very poor) and 10 (very good)

0	1	2	3	4	5	6	7	8	9	10
Very poor										Very good

This is the end of the questionnaire.

<Scoring methods>

The N-QOL results are shown in the overall score and the scores of the two subscales (sleep/energy and bother/concern).

(1) Overall score

An overall score will be calculated using the total points of the 12 items from Q1 to Q12 in the questionnaire above.

・Since the response scale for each question ranges from 4 (lowest QOL) to 0 (highest QOL), the points are reversed so that the scale ranges from 0 (lowest QOL) to 4 (highest QOL). Subsequently, a score will be calculated using the formula below in a manner where the highest QOL scores 100 points out of 100.

・When there is at most one missing value, an overall score can be still calculated using the formula below.

・If the response to Q8 "Concerned that I am disturbing others in the house because of having to get up at night to urinate" is "There is no family or others in the house," this will be handled as a missing value for the question.

◆ If all items (12 questions) have been answered:

$$\text{N-QOL overall score} = \frac{\text{Total points of the items answered}}{4 \times 12\ (48)} \times 100$$

◆ When there is 1 missing value and only 11 questions have been answered:

$$\text{N-QOL overall score} = \frac{\text{Total points of the items answered}}{4 \times 11\ (44)} \times 100$$

(2) Scores of the two subscales (sleep/energy and bother/concern)

・Similar to the overall score, the score for each of the two subscales (sleep/energy or bother/concern) will be calculated using the formula below in a manner where the highest QOL scores 100 points out of 100. (Refer to the table "Structure of the N-QOL Questionnaire" below)

・For the two subscales, the scores can be calculated when all items have been answered (if there is even one missing response, a score cannot be calculated).

$$\text{Points of the N-QOL subscale} = \frac{\text{Total points of the subscale items answered}}{4 \times 6\ (24)} \times 100$$

Table: Structure of the N-QOL Questionnaire

Structure of the subscales (Q1 to Q12)			
Sleep/energy		Bother/concern	
Question No.	Item	Question No.	Item
Q1	Concentration	Q6	Worries about consuming water
Q2	Energy	Q8	Concerns about disturbing family
Q3	Naps during the day	Q9	Worries about getting up at night to urinate
Q4	Productivity	Q10	Concerns that the condition will get worse
Q5	Participation in leisure activities	Q11	Concerns that there is no effective treatment
Q7	Sleep at night	Q12	Bothersome about getting up at night to urinate
Overall health status<br>Q13: Overall disturbance of daily life			

<IPSS>

Questionnaire

Please circle the score that best applies to your urinary condition over the last one month in response to each question.

		Not at all	Less than 1 time in 5	Less than half the time	About half the time	More than half the time	Almost always
Q 1	Over the past month, how often have you had a sensation of not emptying your bladder completely after you finished urinating?	0	1	2	3	4	5
Q 2	Over the past month, how often have you had to urinate again less than two hours after you finished urinating?	0	1	2	3	4	5
Q 3	Over the past month, how often have you found you stopped and started agina several times when you urinated?	0	1	2	3	4	5
Q 4	Over the past month, how often have you found it difficult to postpone urination?	0	1	2	3	4	5
Q 5	Over the past month, how often have you had a weak urinary stream?	0	1	2	3	4	5
Q 6	Over the past month, how often have you had a push or strain to begin urination?	0	1	2	3	4	5

		Not at all	1 time	2 times	3 times	4 times	5 or more times
Q 7	Over the past month, how many times did you most typically get up to urinate from the time you went to bed at night until the time you got up in the morning?	0	1	2	3	4	5

Total IPSS Score =

Symptom severity score: Mild (0-7), moderate (8-19), severe (20-35)

<Sexual Health Inventory for Men>

Only for MEN

Some patients experience erectile dysfunction, also known as impotence. This questionnaire is designed to help you and your doctor to identify if you may be experiencing erectile dysfunction. Please circle the one number of the response that best describes your own situation at the present time.

1. How do you rate your confidence that you could get and keep an erection?

Very low<br>1	Low<br>2	Moderate<br>3	High<br>4	Very high<br>5

2. When you had erections with sexual stimulation, how often were your erections hard enough for penetration (entering your partner)?

No Sexual activity<br>0	Almost never or never<br>1	A few times (much less than half of the time)<br>2	Sometimes (About half the time)<br>3	Most times (Much more than half the time)<br>4	Almost always or always<br>5

3. During sexual intercourse, how often were you able to maintain your erection after you had penetrated (entered) your partner?

Did not attempt intercourse<br>0	Almost never or never<br>1	A few times (much less than half of the time)<br>2	Sometimes (About half the time)<br>3	Most times (Much more than half the time)<br>4	Almost always or always<br>5

4. During sexual intercourse, how difficult was it to maintain your erection to completion of intercourse?

Did not attempt intercourse<br>0	Extremely difficult<br>1	Very difficult<br>2	Difficult<br>3	Slightly difficult<br>4	Not difficult<br>5

5. When you attempted sexual intercourse, how often was it satisfactory for you?

Did not attempt intercourse<br>0	Almost never or never<br>1	A few times (much less than half of the time)<br>2	Sometimes (About half the time)<br>3	Most times (Much more than half the time)<br>4	Almost always or always<br>5
