# Supplementary material for: Efficacy of Corticosteroid Therapy for HTLV-1-Associated Myelopathy: A Randomized Controlled Trial (HAMLET-P)
Source: Viruses. 2022 Jan 12;14(1):136. doi: 10.3390/v14010136 (PMC8780460; doi:10.3390/v14010136)
Supplement: Supplementary file 1 [file viruses-14-00136-s001.zip › Appendix B_StatisticalAnalysisPlan_Rapid_final.pdf]

Confidential

Phase IIb clinical trial of steroid therapy in patients with HAM/TSP Rapid Progressor HAMLET-P（TRINEU1603）

Statistical Analysis Plan
Version 1.0

Date 2020/11/18

Chief Statistician: Kenichiro Tanabe

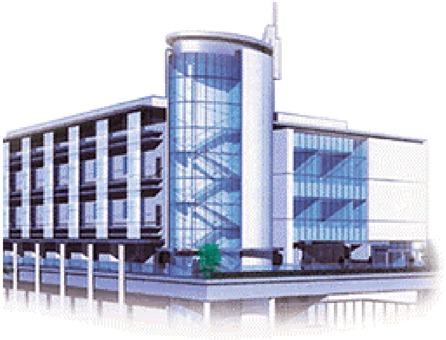

Translational Research Center for Medical Innovation
founded in 2003 by MEXT & Kobe City
for acceleration of translational research in Japan

Copyright © 2022 Translational Research Center for Medical Innovation, Foundation for Biomedical Research and Innovation at Kobe All Rights Reserved.

Table of contents

1 Basic items related to analysis ... 1
1.1 Study Objectives and Research Question ... 1
1.2 Study flow chart ... 3
1.3 Summary of Research ... 5
1.4 Storage location of data ... 11
1.5 Analysis software ... 11
2 Objectives of Analysis ... 12
2.1 Changes to the analysis method from the protocol ... 12
3 Analysis Set ... 13
3.1 Efficacy Analysis Population ... 13
3.1.1 FAS ... 13
3.1.2 PPS ... 13
3.2 Safety Analysis Population ... 13
3.2.1 SS1 ... 13
3.2.2 SS2 ... 13
4 Endpoint Definitions ... 14
4.1 Efficacy Endpoints ... 14
4.1.1 Primary Endpoint ... 14
4.1.2 Secondary Endpoints ... 14
4.1.3 Other Efficacy Endpoints ... 15
4.2 Safety Evaluation ... 17
4.2.1 Secondary Endpoints ... 17
4.2.2 Other Safety Endpoints ... 17
5 Data Handling ... 19
5.1 Handling of timepoint allowable range (allowance) ... 19
5.2 Data handling at discontinuation ... 19
5.3 Handling of missing value ... 19
5.3.1 10-meter timed walk ... 19
5.3.2 OMDS ... 19
5.3.3 2 minute walk distance ... 20
5.3.4 6 minute walk distance ... 20
5.3.5 IPEC1 ... 20
5.3.6 Modified IPEC 2 ... 20

Copyright © 2022 Translational Research Center for Medical Innovation, Foundation for Biomedical Research and Innovation at Kobe All Rights Reserved.

5.3.7 N-QOL 20
5.3.8 OABSS 20
5.3.9 ICIQ-SF 20
5.3.10 IPSS 21
5.3.11 Month of diagnosis 21
5.4 Handling of limit of detection in laboratory values 21
5.5 Handling of outliers or abnormal values 21
5.6 Definition of the baseline 21
5.6.1 10-meter timed walk 21
5.6.2 Cerebrospinal fluid concentration of neopterin 21
5.6.3 CXCL10 concentration 21
5.6.4 Other items 21
6 Analysis methods 22
6.1 Subject composition 23
6.2 Subjects for analysis 24
6.3 Patient demographics 24
6.4 Treatment status 24
6.5 Efficacy endpoints 25
6.5.1 Primary Endpoint 25
6.5.2 Secondary Endpoints 25
6.5.3 Other Efficacy Endpoints 26
6.6 Safety evaluation 30
6.6.1 Secondary Endpoints 30
6.6.2 Other Safety Endpoints 31
6.7 Lists 32
6.8 Sample size calculation and power justification 34
7 Appendix 36
8 Reference Material 36
9 References 36
10 Revision History 37

Copyright © 2022 Translational Research Center for Medical Innovation, Foundation for Biomedical Research and Innovation at Kobe All Rights Reserved.

Abbreviations

Abbreviation	Unabbreviated term
ADS	analysis dataset
ALP	alkaline phosphatase
ALT	alanine aminotranferase
AST	aspartate aminotransferase
BA	basophil
BUN	blood urea nitrogen
Ca	calcium
Cr	creatinine
CTCAE v4.0	common terminology criteria for adverse events v4.0
CXCL10	C-X-C motif chemokine ligand 10
DM	data manager
EDC	Electronic Data Capture
EO	eosinophil
FAS	full analysis set
HAM	HTLV-1-associated myelopathy
Hb	hemoglobin
HbA1c	hemoglobin A1c
HDL-C	high-density nipoprotein cholesterol
Hct	hematocrit
HTLV-1	human T-lymphotropic virus type 1
ICIQ-SF	international consultation on incontinence questionnaire-short form
ICSA	individual clinically significant abnormalities
IP	inorganic phosphorus
IPEC1	-
IPSS	international prostate symptom score
K	kalium
LDL-C	low-density lipoprotein cholesterol
LLT	lowest level terms
Lym	lymphocyte
MAS	modified Ashworth scale
MCH	mean corpuscular hemoglobin
MCHC	mean corpuscular hemoglobin concentration
MCV	mean corpuscular volume
MedDRA/J	medical dictionary for regulatory activities/J
MO	monocyte
Modified IPEC 2	-
MRI	magnetic resonanse imaging

Copyright © 2022 Translational Research Center for Medical Innovation, Foundation for Biomedical Research and Innovation at Kobe All Rights Reserved.

Abbreviation	Unabbreviated term
Na	natrium
NE	neutrophil
N-QOL	nocturia quality of life questionnaire
OABSS	overactive bladder symptom score
OMDS	Osame's motor disability score
PLT	platelet
PPS	per protocol set
PT	preferred terms
QOL	quality of life
RBC	red blood cell
SS1	safety set 1
SS2	safety set 2
SOC	system organ class
T-Cho	total cholesterol
TG	triglyceride
VAS	visual analog scale
WBC	white blood cell
T.BIL	total bilirubin

Copyright © 2022 Translational Research Center for Medical Innovation, Foundation for Biomedical Research and Innovation at Kobe All Rights Reserved.

1 Basic items related to analysis

1.1 Study Objectives and Research Question

Primary objective

To test the efficacy of i.v. methylprednisolone followed by oral prednisolone therapy in patients with rapidly progressive HAM/TSP compared to oral prednisolone alone.

Secondary objectives

To test the safety of i.v. methylprednisolone and oral prednisolone therapy in patients with rapidly progressive HAM/TSP.

To test the efficacy of oral prednisolone therapy in patients with rapidly progressive HAM/TSP.

Research Question:

① In a randomized controlled study in the rapid progressors among HAM patients, does methylprednisolone treatment have superior efficacy effect compared to oral prednisone treatment?

② Is it safe to treat the rapid progressors among HAM patients with methylprednisolone and prednisolone?

③ Is prednisolone treatment for the rapid progressors among HAM patients effective?

Estimand:

1. Target population: Full analysis set (FAS) (see Section 3.1.1)

1.1.1.1 Variable (or endpoint): Presence or absence of "≥30% improvement in 10-meter timed walk" or "≥1 improvement in the Osame's Motor Disability Score (OMDS)" at Day 15 compared to baseline (see Section 4.1.1)

2. Considerations for interim events: See section 5.3.1 for details
   i) Data may also be collected after discontinuation of study treatment for the purpose of collecting prognostic data. However, data after discontinuation of study treatment will be handled as missing as the primary endpoint analysis will be performed for efficacy evaluation of study treatment at Day 15. Therefore, the effect of post-treatment, etc. will not be considered.
   ii) If at least either of 10-meter timed walk or the OMDS at Day 15 is missing, the subject should be handled as "no improvement."
3. Summary of variables at the population level

Null hypothesis: The percentage of improvement in the methylprednisolone group is equal to that in the prednisolone group at Day 15.

The treatment effect will be estimated and determined based on difference in percentage of

Copyright © 2022 Translational Research Center for Medical Innovation, Foundation for Biomedical Research and Innovation at Kobe All Rights Reserved.

improvement between groups and its 95% confidence interval. However, no significant level is defined in the tests.

Copyright © 2022 Translational Research Center for Medical Innovation, Foundation for Biomedical Research and Innovation at Kobe All Rights Reserved.

1.2 Study flow chart

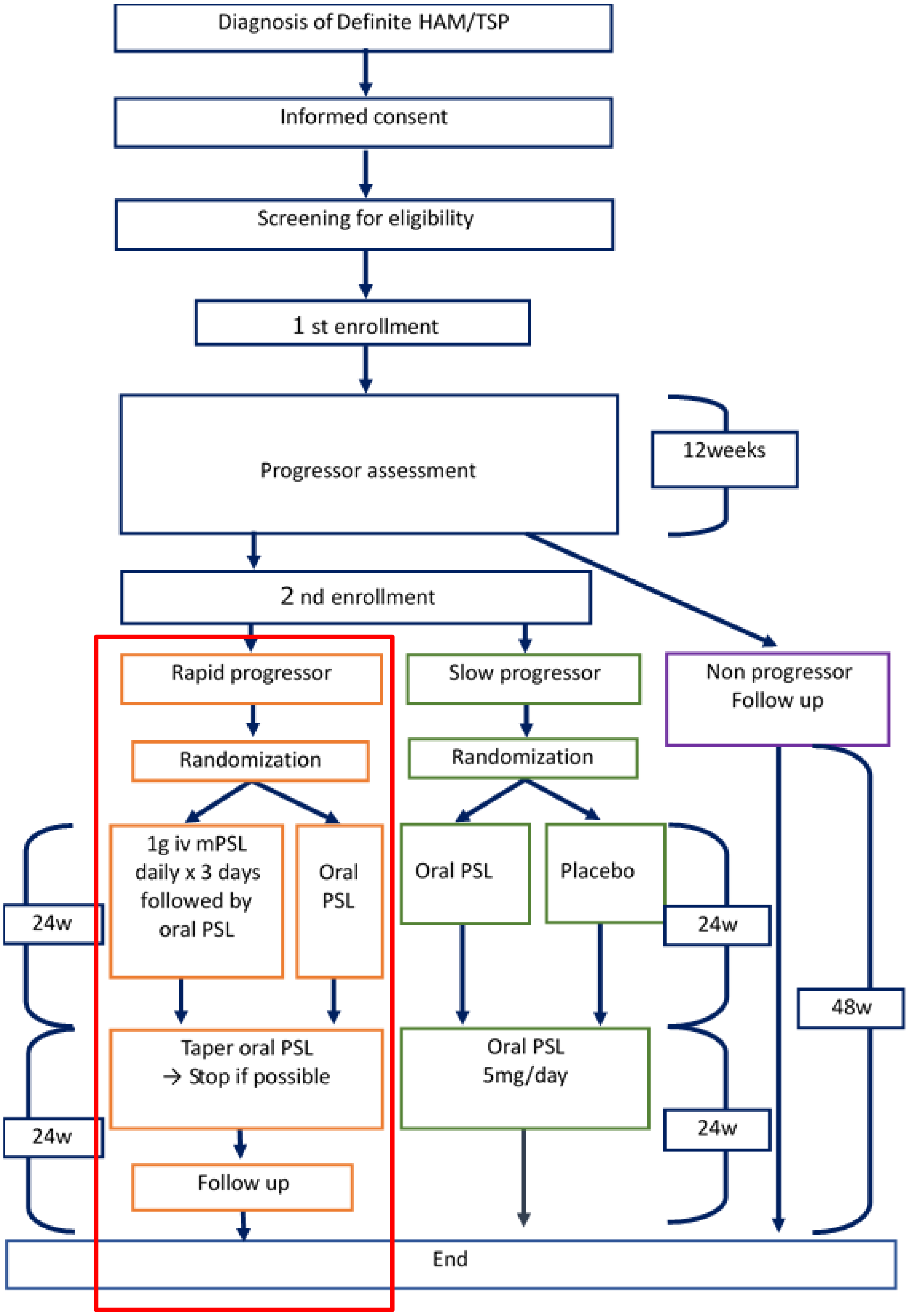

Copyright © 2022 Translational Research Center for Medical Innovation, Foundation for Biomedical Research and Innovation at Kobe All Rights Reserved.

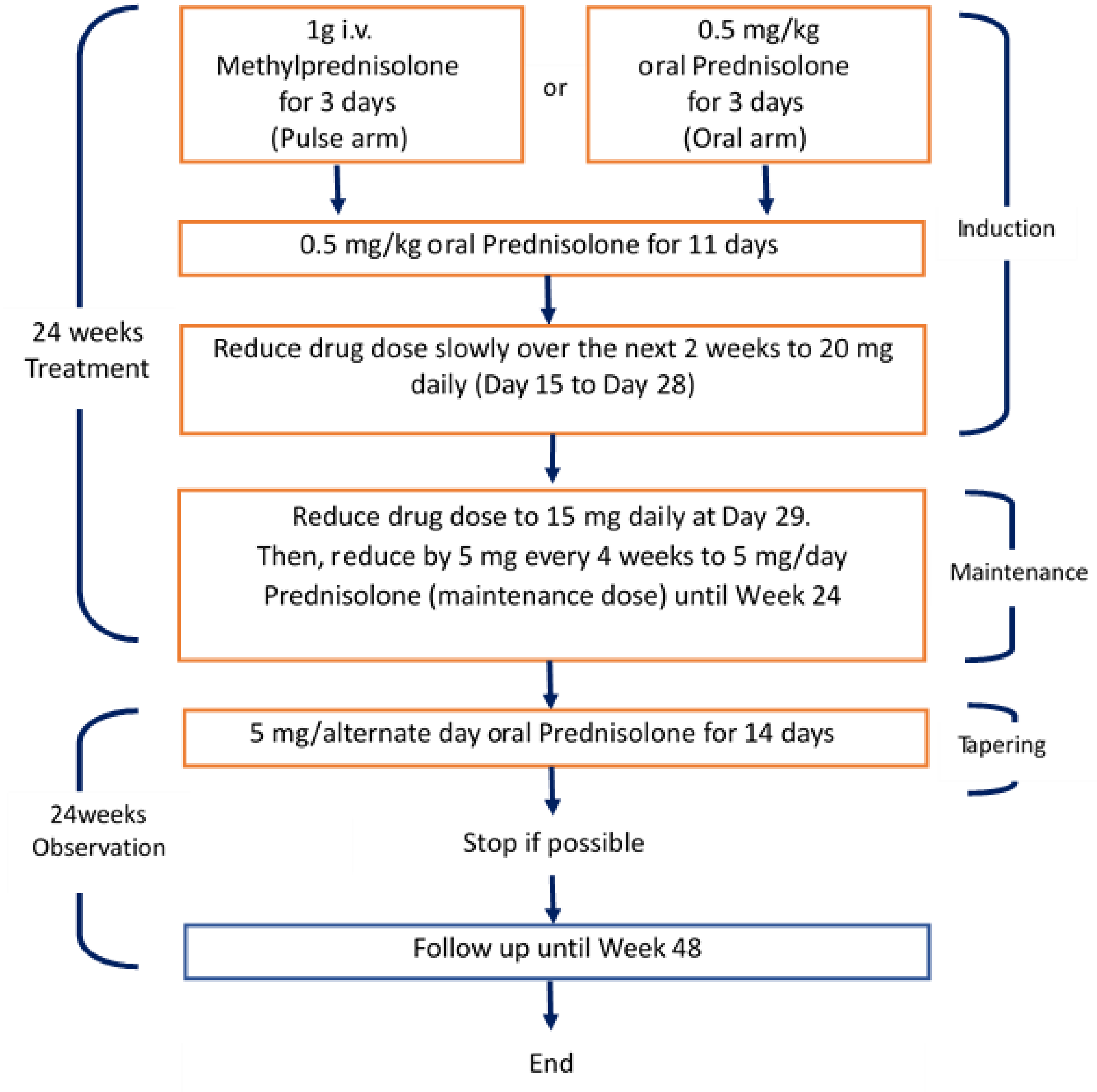

Copyright © 2022 Translational Research Center for Medical Innovation, Foundation for Biomedical Research and Innovation at Kobe All Rights Reserved.

1.3 Summary of Research

Target sample size	8 subjects
Subject enrollment period	August 2016 to March 2019 (2 years and 8 months)
Follow-up end date	(Secondary enrollment) One year (48 weeks) after the last subject enrollment
Observation/intervention	Interventional Research
Phase of the study	Phase IIb
Control Type	Active drug-controlled study<br>Study group: Treated with intravenous methylprednisolone<br>Control group: Treated with oral placebo
Randomization	Stratified randomization with permuted block design (block size of 2)<br>Stratification factor:<br>• Number of canes: One or less/Two or more<br>(A walker should be regarded as “two or more”.)
Blinding Level	prospective, randomized, open, blinded-endpoint（PROBE）

Copyright © 2022 Translational Research Center for Medical Innovation, Foundation for Biomedical Research and Innovation at Kobe All Rights Reserved.

Schedule Table：

Period		Screening*1	1st Enrollment*3	Progressor assessment*2				2nd Enrollment*4	Study drug treatment period								Observation period				Unplanned visits*25	Post-observation*6	At discontinuation*7
Week		-16 ～ - 12*3		-12*3	-8	-4	Last assessment*4 ±7		0			2	4	8	12	24*5	28	32	36	48			
Day		-112～-84 ±7		-84 ±7	-56 ±7	-28 ±7			1*4	2	3	15 ±3	29 ±7	57 ±7	85 ±7	169 ±7	197 ±7	225 ±7	253 ±7	337 ±7			
Informed consent		x																					
Eligibility confirmation		x																					
Study drug treatment									→	→	→	→	→	→	→	→							
Clinical Assessment	Physical examination	x		x*2	x*2	x*2	x*2					x	x	x	x	x	x	x	x	x		x	x
	Blood pressure, Pulse rate, Body temperature	x		x*2	x*2	x*2	x*2					x	x	x	x	x	x	x	x	x			x*8
	Height, Body weight	x														x*9				x*9			
	OMDS ☆	x		x*2	x*2	x*2	x*2					x	x	x	x	x	x	x	x	x	x		x
	Walking tests *A ☆	x		x*2	x*2	x*2	x*2					x	x	x	x	x	x	x	x	x	x		x*10
	Walking aids *B ☆	x		x*2	x*2	x*2	x*2					x	x	x	x	x	x	x	x	x	x		
	MAS ☆	x		x*2	x*2	x*2	x*2					x	x	x	x	x	x	x	x	x			

Period		Screening*1	1st Enrollment*3	Progressor assessment*2				2nd Enrollment*4	Study drug treatment period								Observation period				Unplanned visits*25	Post-observation*6	At discontinuation*7
Week		-16 ～ - 12*3		-12*3	-8	-4	Last assessment*4 ±7		0			2	4	8	12	24*5	28	32	36	48			
Day		-112～-84 ±7		-84 ±7	-56 ±7	-28 ±7			1*4	2	3	15 ±3	29 ±7	57 ±7	85 ±7	169 ±7	197 ±7	225 ±7	253 ±7	337 ±7			
	IPEC1 ☆	x		x*2	x*2	x*2	x*2					x	x	x	x	x	x	x	x	x			
	VAS *C ☆			x*2	x*2	x*2	x*2					x	x	x	x	x	x	x	x	x			
	QOL *D ☆	x		x*2	x*2	x*2	x*2					x	x	x	x	x	x	x	x	x			
	Urinary dysfunction *E ☆	x		x*2	x*2	x*2	x*2					x	x	x	x	x	x	x	x	x			
Blood tests *11 *F/ Urinalysis*G		x		x*2	x*2	x*2	x*2				x*12	x	x	x	x	x	x		x	x			x
Virus tests *18*H		x*13											x*14	x*14	x*14	x*14	x*15		x*15	x*15			x*16
Pregnancy test*17		x					(x)*2																
Cerebrospinal fluid tests*18 *I		x*19					x*2					x				x	(x)*20	(x)*20	(x)*20	x			
Accompaning research	Blood tests *J	x		x*2	x*2	x*2	x*2					x	x	(x)	x	x	x		x	x			
Accompaning research	Biobank samples	x		x*	x*	x*	x*2					x	x	(x	x	x	x		x	x			

Period		Screening*1	1st Enrollment*3	Progressor assessment*2				2nd Enrollment*4	Study drug treatment period								Observation period				Unplanned visits*25	Post-observation*6	At discontinuation*7
Week		-16 ～ - 12*3		-12*3	-8	-4	Last assessment*4 ±7		0			2	4	8	12	24*5	28	32	36	48			
Day		-112～-84 ±7		-84±7	-56±7	-28±7			1*4	2	3	15±3	29±7	57±7	85±7	169±7	197±7	225±7	253±7	337±7			
	(Blood)*21			2	2	2								)									
	Cerebrospinal Fluid tests *K	x*19					x*2					x				x	(x)*20	(x)*20	(x)*20	x			
	Biobank samples (Cerebrospinal fluid) *21	x*19					x*2					x				x	(x)*20	(x)*20	(x)*20	x			
MRI		x*22		x*22									(x)			x				(x)			
Intraocular pressure*23		x*22		x*22									x	x*24	x*24	x*24			x*24	x*24			

☆: The person in charge of assessment of the clinical efficacy endpoints will perform the evaluation

*1: Subjects who meet the rapid progressor criteria at screening will not need to undergo the progressor assessment period and may enter the primary and secondary enrollment and start the study drug treatment within 7 days of screening. In this case, the values at the screening will be the baseline value.

*2: The progressor assessment period will be 12 weeks. Subjects who meet the rapid progressor criteria during the progressor assessment period may enter the secondary enrollment and start the study drug treatment after all required tests and assessments have been performed on the last assessment date. The values at the screening will be used as the baseline values and the progressor assessment period is not required only if the period between screening and the start of the study drug treatment is less than 4 weeks. If the time between screening and initiation of study treatment exceeds 4 weeks, the last assessment date must be performed.

*3: The progressor assessment period on Week -12 and screening can be performed on the same day. In this case, the items specified in the screening should be performed. In addition, the primary enrollment should occur within 7 days of screening.

*4: Secondary enrollment should be performed within 7 days of the last assessment date to start Day 1 (study drug treatment). The date may be the same as the date of the final assessment.

*5: For subjects who discontinue the study due to clinical exacerbation of HAM, data will be collected on “details of the post-treatment regimen (for treatment of HAM [including the drugs listed in Exclusion Criterion 1 and Exclusion Criterion 6 and treatment with HAL], “10-meter timed walk”, and “Osame’s Motor Disability Score” until the period corresponding to Week 24 in the study treatment period.

*6: To be performed 28 days (+28 days) after the final dose of the study drug. Follow-up is not required if 28 days have passed since the final dose of the study drug at Week 48.

*7: To be performed within 28 days after discontinuation of the study drug during the treatment period or before the start of post-treatment, whichever is earlier.

*8: Body temperature will be measured as needed.

*9: Height measurement is not required.

*10: 10-meter timed walk, 6-minute walk distance, and 2-minute walk distance will be performed. Timed up-and-go test is not required.

*11: Fasting glucose will be measured on Day 3 of rechallenge in all subjects who are re-treated with methylprednisolone.

*12: Fasting blood glucose only will be measured for subjects in the pulse group.

*13: HBs antigen, HBs antibody, HBc antibody, HCV antibody, HIV-1 antibody and HIV-2 antibody will be tested only at screening. If either HBs antibody or HBc antibody is positive, quantitative HBV-DNA measurement will be performed.

However, quantitative HBV-DNA is not required if the positive HBs antibody is clearly due to vaccination.

*14: Quantitative HBV-DNA measurement will be performed only in subjects who are positive for HBs or HBc antibodies (with the exception of positive HBs antibody clearly due to vaccination) at screening and whose quantitative HBV-DNA is below the detection sensitivity.

*15: Subjects who are positive for HBs or HBc antibodies (with the exception of positive HBs antibody clearly due to vaccination) at screening and whose quantitative HBV-DNA is below the detection sensitivity will be closely monitored for liver function tests, and if abnormal values are observed (if AST and ALT increase is ≥ 5 times the reference value at the site), quantitative HBV-DNA measurement will be performed.

*16: For subjects who are positive for HBs or HBc antibodies (with the exception of positive HBs antibody clearly due to vaccination) at screening and whose quantitative HBV-DNA is below the detection sensitivity, if the liver function test shows abnormal values (if AST and ALT increase is ≥ 5 times the reference value at the site), quantitative HBV-DNA measurement will be performed.

*17: It is not required for subjects who are permanently sterilized. (Permanent sterilization: postmenopausal [with no menses for at least 48 weeks with no other medical reason], post sterilization [hysterectomy, bilateral salpingectomy, bilateral oophorectomy], tubal occlusion without tubal ligation)

*18: If data is available from a previous test on anti-HTLV-1 antibody for diagnosis of HAM, that data may be used.

*19: Data obtained within 12 weeks prior to the date of consent may be used.

*20: When restarting prednisolone treatment due to exacerbation after completion of the study drug treatment, it is recommended to perform a cerebrospinal fluid test before restart.

*21: Residual samples will be used as samples for biobanking.

*22: MRI and intraocular pressure measurement will be performed at any visit from screening to the progressor assessment period and the data will be used as the baseline value. For MRI scans, data obtained within 12 weeks prior to the date of informed consent can be used.

However, if an image used as the baseline shows findings suggestive of inflammation in the spinal cord, a retest must be performed within 12 weeks prior to the secondary enrollment for that site only. In addition, if data obtained within 12 weeks prior to the date of informed consent at another hospital is available and indicates no findings of inflammation in the spinal cord, a retest is not required.

*23: Intraocular pressure will be measured at the visit corresponding to 4 weeks (± 7 days) after rechallenge with methylprednisolone in all subjects.

*24: To be performed only in subjects with glaucoma.

*25: Unscheduled visit is allowed to confirm the extent of disease progression. At the unscheduled visit, only the Osame’s Motor Disability Score, the walk test (10-meter timed walk), and the use of walking aids will be performed.

*A：Waling tests (10 meter Timed Walk, 6 minute Walk Distance, 2 minute Walk Distance, Timed Up and Go Test)

*B：Ambulatory aid used at the clinic and home

*C：VAS（Overall, Walking, Lower extremity pain）

*D：QOL (Modified IPEC2, N-QOL, Sexual Health Inventory for Men）

*E：Urinary dysfunction (OABSS, ICIQ-SF, IPSS)

*F：Blood tests： Complete blood count with differential, Urea nitrogen and electrolytes (BUN, Cre, Na, K), Liver function (AST, ALT, ALP, T-Bil),
Glucose metabolism (HbA1c (NGSP), Fasting blood glucose), Bone metablism (Ca, P), Lipids (T-Cho, LDL-C, HDL-C, TG)

*G：Urinalysis: glucose, protein

*H：Virus tests: Hepatitis B virus (HBc antibody ((at screening only), HBs antibody (at screening only), HBs antigen (at screening only), HBV-DNA)
HCV antibody (at screening only), HIV-1 antibody・HIV-2 antibody (at screening only)
Anti-HTLV-1 antibody for HAM diagnosis (at screening only)

*I：Cerebrospinal fluid tests: Anti-HTLV-1 antibody for HAM diagnosis (at screening only)
Safety: Glucose
Efficacy: Cell counts/ Cell fractionation, Toral protein, neopterin

*J：Blood tests (Accompanying research): β2-microgloblin, B- and T-cell subsets/markers、sIL-2R、HTLV-1 proviral load, sVCAM-1、SPARC

*K：Cerebrospinal fluid tests (Accompanying research): HTLV-1 anribody, HTLV-1 proviral load, CXCL10, sVCAM-1

1.4 Storage location of data

Data provided by the data manager (DM) and analysis data set (ADS) used for the analysis are stored below.

DM Dataset: (After secondary enrollment)	Z:¥¥01_Project¥¥TRIProject 2¥¥258_TRINEU1603(Yamano) ¥¥09_Biostatistics¥¥03_Final analysis¥¥2_ANALYSIS¥¥EXECUTE1¥¥01_DATA ¥¥DM_RAW¥¥20161209_Dummy¥¥SASDS
DM Dataset: (Before secondary enrollment)	Z:¥¥01_Project¥¥TRIProject2¥¥260_TRINEU1605(Yamano) ¥¥09_Biostatistics¥¥03_Final analysis¥¥2_ANALYSIS¥¥EXECUTE1¥¥01_DATA ¥¥DM_RAW¥¥20161209_Dummy¥¥SASDS
ADS :	Z:¥¥01_Project¥¥TRIProject 2¥¥258_TRINEU1603(Yamano) ¥¥09_Biostatistics¥¥03_Final analysis¥¥2_ANALYSIS¥¥EXECUTE1¥¥01_DATA ¥¥ADS¥¥Main

1.5 Analysis software

Analysis will be performed using SAS Version 9.4 (SAS Institute Inc., Cary, NC, USA).

2 Objectives of Analysis

This Statistical Analysis Plan (hereinafter referred to as the "SAP") provides details of the final analyses as defined in the Protocol [1)] (Version 1.12).

2.1 Changes to the analysis method from the protocol

Changes to the analysis method from the Protocol[1)] (Version 1.12) are shown below.

Details before change	Details after change	Reason for change
8. Study design and evaluation (Rapid)	Added C-X-C motif chemokine ligand 10 (CXCL10) concentration not subject to Electronic Data Capture (EDC) to the secondary and exploratory efficacy endpoints.	Because, in consultation with the coordinating investigator, it was considered that CXCL10 concentration should be evaluated in the same way as cerebrospinal fluid concentration of neopterin.

3 Analysis Set

3.1 Efficacy Analysis Population

The FAS and the per protocol set (PPS) will be defined as the population for the analysis of efficacy. The FAS will be used for analyses of the primary efficacy results, and the PPS will be used for analyses of the secondary efficacy results.

3.1.1 FAS

A population of all subjects secondary enrolled as a rapid progressor and randomized, excluding the subjects listed below. It will be used for analyses of the primary efficacy results.

- Subjects who have never received study treatment
- Subjects who do not have a pre-treatment value of 10-meter timed walk or OMDS
- Subject who is subsequently found to have violated the eligibility criteria

3.1.2 PPS

Subjects in the FAS who have no major protocol deviations. It will be used for analyses of the secondary efficacy results. Serious deviations will be determined by the time the database is locked.

3.2 Safety Analysis Population

The Safety Set 1 (SS1) and Safety Set 2 (SS2) will be defined as the safety analysis sets. The SS1 will be used for analyses of the primary safety results, and the SS2 will be used for the secondary safety results.

3.2.1 SS1

A population of all subjects secondary enrolled as a rapid progressor who have received at least one dose of study treatment. It will be used for analyses of the primary safety results

3.2.2 SS2

A population of subjects included in the SS1 excluding those who is found not to meet any of the inclusion criteria or to meet any of the exclusion criteria after secondary enrollment as a rapid progressor. It will be used for analyses of the secondary safety results.

4 Endpoint Definitions

4.1 Efficacy Endpoints

For "≥30% improvement in 10-meter timed walk", the original measurement values without natural-logarithmical transformation should be used.

4.1.1 Primary Endpoint

Presence or absence of "≥30% improvement in the 10-meter timed walk" or "≥1 improvement in the OMDS" at Day 15 compared to the baseline value

4.1.2 Secondary Endpoints

4.1.2.1 10-meter timed walk

- Presence or absence of "≥30% improvement in the 10-meter timed walk" at Day 15 compared to the baseline value
- Area under the curve (Baseline, Day15, Day29 [Week 4])
  For the calculation of area under the curve, for the Y-axes, the baseline measurement will be taken as the reference value, and the baseline value will be taken as 1 and the values at Day 15 and Day 29 will be plotted as the ratio to baseline value. In addition, the X-axes shall be defined as 0 for the baseline, 0.5 for Day 15, and 1 for Day 29. For 10-meter timed walk, the original measurement values will be used instead of the natural-logarithmically transformed values.
- Change at Day15/Day29 (Week 4) compared to baseline

4.1.2.2 2-minute walk distance

- Area under the curve (Baseline, Day15, Day29 [Week 4])
  The calculation method for area under the curve is the same as that for the 10-meter walk time.
- Change at Day15/Day29 (Week 4) compared to baseline

4.1.2.3 6-minute walk distance

- Area under the curve (Baseline, Day15, Day29 [Week 4])
  The calculation method for area under the curve is the same as that for the 10-meter walk time.
- Change at Day15/Day29 (Week 4) compared to baseline

4.1.2.4 OMDS

- Presence or absence of "≥1 improvement in the OMDS" at Day 15 compared to the baseline value

4.1.2.5 Cerebrospinal fluid concentration of neopterin

- Change at Day15 compared to baseline

4.1.2.6 Proportion of subjects who received i.v. methylprednisolone between Day29（Week 4）and

Day169（Week 24）

4.1.2.7 Cerebrospinal fluid concentration of CXCL10

- Change at Day15 compared to baseline

4.1.3 Other Efficacy Endpoints

4.1.3.1 10-meter timed walk

- Change at Day85 (Week 12)/ Day169 (Week 24) from baseline

4.1.3.2 2-minute walk distance

- Change at Day85 (Week 12)/ Day169 (Week 24) from baseline

4.1.3.3 6-minute walk distance

- Change at Day85 (Week 12)/ Day169 (Week 24) from baseline

4.1.3.4 Cerebrospinal fluid concentration of neopterin

- Change at Day169 (Week 24) from baseline

4.1.3.5 Subjects who discontinue the study

- Proportion of subjects who discontinue the study due to clinical exacerbation of HAM (an increase of 100% or more in 10-meter timed walk compared to baseline) between Day30 and Day169 (Week 24)

4.1.3.6 Subjects who could not discontinue the drug

- Proportion of subjects who could not discontinue the drug at Day183 (Week 26)

4.1.3.7 Subjects who resumed steroid treatment

- Proportion of subjects who resumed steroid treatment by Day337 (Week 48) among those who had discontinued the drug at Day183 (Week 26).

4.1.3.8 Cerebrospinal fluid concentration of CXCL10

- Change at Day169 (Week 24) from baseline

4.1.3.9 Use of walking aids

- Ambulatory aid used at the clinic
- Ambulatory aid used at home

4.1.3.10 IPEC1

- Total score

4.1.3.11 QOL：quality of life

4.1.3.11.1 Modified IPEC 2

- Total score

4.1.3.11.2 N-QOL：nocturia quality of life questionnaire

Since the response scale for each question ranges from 4 (lowest QOL) to 0 (highest QOL), the points are reversed so that the scale ranges from 0 (lowest QOL) to 4 (highest QOL). Subsequently, a score will be calculated using the formula below in a manner where the highest QOL scores 100 points out of 100. If the response to Q8 “Concerned that I am disturbing others in the house because of having to get up at night to urinate” is “There is no family or others in the house,” this will be handled as a missing value for the question.

- Overall score
  - ✓ If all items (12 questions) have been answered:
    Total points of the items × 100/48
  - ✓ When there is 1 missing value and only 11 questions have been answered:
    Total points of the items × 100/44
- Subscales
  - ✓ Sleep/Energy
    Total points of the subscale items × 100/24
  - ✓ Bother/Concern
    Total points of the subscale items × 100/24
- Overall health status

Q13: Overall disturbance of daily life

4.1.3.11.3 Sexual Health Inventory for Men

- Individual score of question items

4.1.3.12 MAS：modified Ashworth scale

- Scale

4.1.3.13 VAS：visual analog scale

- Global assessment
- Walking assessment
- Lower extremity pain assessment

4.1.3.14 Urinary dysfunction

4.1.3.14.1 OABSS：overactive bladder symptom score）

- Total score

4.1.3.14.2 ICIQ-SF：international consultation on incontinence questionnaire-short form

- Individual score of question items

4.1.3.14.3 IPSS：international prostate symptom score

- Total score

4.1.3.15 Time up and go test

- Time (second)

4.1.3.16 OMDS

- Grade

4.2 Safety Evaluation

4.2.1 Secondary Endpoints

4.2.1.1 Adverse events

- Occurrence status (frequency, severity)

However, events that occurred more than 28 days after the final dose of the study drug in subjects who discontinued the study, or those occurred more than 28 days after the last observation day at Week 48 (the last day if the 48-week visit measurement occurred for more than one day) in subjects who completed the study will not be included in the analysis.

4.2.2 Other Safety Endpoints

4.2.2.1 Clinical laboratory examination

- Complete blood count
  - ✓ WBC：white blood cell
  - ✓ WBC differential
  - ✓ RBC：red blood cell
  - ✓ Hb：hemoglobin
  - ✓ Hct：hematocrit
  - ✓ MCV：mean corpuscular volume
  - ✓ MCH：mean corpuscular hemoglobin
  - ✓ MCHC：mean corpuscular hemoglobin concentration
  - ✓ PLT：platelet
- Blood chemistry
  - ✓ BUN：blood urea nitrogen
  - ✓ Cr：creatinine
  - ✓ Na：natrium
  - ✓ K：kalium
  - ✓ AST：aspartate aminotransferase
  - ✓ ALT：alanine aminotranferase
  - ✓ ALP：alkaline phosphatase
  - ✓ T.BIL：total bilirubin
  - ✓ HbA1c：hemoglobin A1c
  - ✓ Fasting blood glucose

- ✓ Ca：calcium
- ✓ IP：inorganic phosphorus
- ✓ T-Cho：total cholesterol
- ✓ LDL-C：low-density lipoprotein cholesterol
- ✓ HDL-C：high-density lipoprotein cholesterol
- ✓ TG：triglyceride
- Urinalysis (Urine glucose, Urine protein)

4.2.2.2 Vital signs

- Body weight
- Blood pressure (systolic, diastolic)
- Pulse rate
- Body temperature

5 Data Handling

5.1 Handling of timepoint allowable range (allowance)

For data at scheduled visits, the FAS will, in principle, accept the any range of timepoints, but if data is outside the range or if there are multiple values within the range, the handling will be determined after discussion with the coordinating investigator and the principal investigator. Data handling in the PPS, SS1, and SS2 will also be determined after discussion with the coordinating investigator and the principal investigator.

5.2 Data handling at discontinuation

Data will be included in lists. If data is included in the timepoint allowable range, then it may be used for analysis.

5.3 Handling of missing value

All efficacy data after study treatment discontinuation will be treated as missing. Missing value will remain missing for items not listed below.

5.3.1 10-meter timed walk

- In principle, the mean of two measurements will be used as the value at the timepoint; however, if only one measurement has been taken (missing for one measurement), the measurement will be used as is for evaluation.
- If the 10-meter timed walk on Day 15 is missing in the analysis for presence or absence of "≥30% improvement in 10-meter timed walk" or "≥1 improvement in the OMDS" at Day 15 compared with baseline, the subject will be treated as "no improvement".
- If only Day 15 measurement values are missing in the calculation of area under the curve (baseline value, Day 15 and Day 29 [Week 4]), area under the curve will be calculated by linearly connecting 1, the Y axis value at baseline, and the Y axis value at Day 29 (the ratio of measurement values at Day 29 to the baseline value). If only the Day 29 measurement is missing, the area under the curve will be calculated using the Y axis value at Day 15 (ratio of measurement at Day 15 to baseline value) as the Y axis value at Day 29. If data have not been measured through Day 29, the area under the curve will be calculated by a linear imputation to the Day 29 value by connecting 1, the Y axis value at baseline, and the Y axis value at the earliest measurement timepoint after Day 29 as the ratio of the measurement at that timepoint to the baseline value.

5.3.2 OMDS

- If the OMDS on Day 15 is missing in the analysis for presence or absence of "≥30% improvement in 10-meter timed walk" or "≥1 improvement in the OMDS" at Day 15 compared with baseline, the subject will be treated as "no improvement".

5.3.3 2 minute walk distance

- This will be imputed similarly with respect to the calculation of area under the curve (baseline value, Day 15 and Day 29 [Week 4]) using the method described in "5.3.1 10-meter timed walk".

5.3.4 6 minute walk distance

- This will be imputed similarly with respect to the calculation of area under the curve (baseline value, Day 15 and Day 29 [Week 4]) using the method described in "5.3.1 10-meter timed walk".
- If there is a suspension within 2 minutes, the 6-minute walk distance will be missing on the EDC data. In this case, the 2-minute walk distance value will be imputed as the 6-minute walk distance.

5.3.5 IPEC1

- If at least 1 of all the questions (11 questions) of “motor score”, “spasm score”, “perceptive score”, and “sphincter muscle score” is missing, the overall score will be missing.

5.3.6 Modified IPEC 2

- If at least 1 of all the questions (8 questions) of “body movement” and “bladder and bowel movements” is missing, the overall score will be missing.

5.3.7 N-QOL

- If the answer in Q8 is “No family members or cohabiters”, Q8 will be missing.
- The overall score will be calculated only if all 12 questions have been answered from Q1 through Q12 (not considering Q13) or if only 11 questions have been answered with one missing question. Thus, if 2 or more questions from Q1 to Q12 are missing, the overall score will be missing.
- The subscale “sleep/energy” will only be calculated if all 6 questions in Q1, Q2, Q3, Q4, Q5, and Q7 have been answered. Thus, if at least 1 of the 6 questions in Q1, Q2, Q3, Q4, Q5, and Q7 is missing, the subscale “sleep/energy” will be missing.
- The subscale of “worry/concern” will be only calculated if all 6 questions in Q6, Q8, Q9, Q10, Q11, and Q12 have been answered. In other words, if at least 1 of the 6 questions in Q6, Q8, Q9, Q10, Q11, and Q12 is missing, the subscale of “worry/concern” will be missing.

5.3.8 OABSS

- The overall score will only be calculated if all Q1 through Q4 are answered. Thus, if at least one of Q1 through Q4 is missing, the overall score will be missing.

5.3.9 ICIQ-SF

- For Q4 (When do you have urine leakage?), if the first item of (None - No urine leakage) is checked (data shows a score of 1), all items 2 through 8 of Q4 will be imputed with 0 (None) for the analysis.

5.3.10 IPSS

- The IPSS (sum of the scores in Q1 to Q7) will only be calculated if all Q1 to Q7 responses have been made. Thus, if at least 1 question is missing from Q1 to Q7, IPSS will be missing.

5.3.11 Month of diagnosis

- When calculating disease duration, if the month of diagnosis is missing, ‘January’ will be imputed. However, the data will be considered as missing in lists.

5.4 Handling of limit of detection in laboratory values

For summary statistics of laboratory values, the site’s limit will be used as the measurement value for laboratory values that are considered to be the limit of detection. However, the data will be considered as missing in lists, and will be shown so that it can be identified as below the quantification limit.

5.5 Handling of outliers or abnormal values

As a general rule, outliers or abnormal values will not be processed. However, if there is any problem, the handling of such problem shall be determined through consultation with the coordinating investigator and the principal investigator.

5.6 Definition of the baseline

5.6.1 10-meter timed walk

- This will be the final assessment (assessment at the time of the last progressor assessment).

5.6.2 Cerebrospinal fluid concentration of neopterin

- In principle, the final assessment is made before the start of treatment, but if a subject rolls over from a non-progressor and it is considered that there is a problem with the period from the final assessment, the handling should be determined after discussion with the coordinating investigator and the principal investigator, including data obtained within 12 weeks prior to obtaining consent.

5.6.3 CXCL10 concentration

- Same as “5.6.2 Cerebrospinal fluid concentration of neopterin”.

5.6.4 Other items

- In principle, the final assessment is made before the start of treatment, but if a subject rolls over from the non-progressor and it is considered that there is a problem with the period from the final assessment, the handling should be determined after discussion with the coordinating investigator and the principal investigator.

6 Analysis methods

General handling of statistical analyses will be described. If specific handling is defined, it should be prioritized.

- In principle, in calculation of summary statistics (sample size, mean, standard deviation, minimum, first quartile, median, third quartile, and maximum values), the number of decimal places for values of mean, standard deviation, first quartile, median, and third quartile shall be 1 digit more than the maximum number of digits of data. Similarly, the number of decimal places for values of differences of mean values between groups, 95% confidence interval other than percentage, least square mean (LSMean), standard error (SE) for LSMean, and 95% confidence interval for LSMean shall also be 1 digit more than the maximum number of digits of data. The number of decimal places for minimum and maximum values shall be the same as the maximum number of digits of data.
- Summary statistics for each timepoint for 10-meter timed walk, cerebrospinal fluid concentration of neopterin, and CXCL10 concentration will be derived for both the original measurement and the natural-logarithmically transformed value. Mean, standard deviation, first quartile, median and third quartile of 10-meter timed walk shall be displayed to three decimal places. Similarly, differences of mean values between groups and 95% confidence interval other than percentage shall also be displayed to three decimal places. Minimum and maximum shall be displayed to two decimal places.
- For variables that may be circulating decimals or irrational numbers, mean, standard deviation, first quartile, median, third quartile, differences of mean values between groups, and 95% confidence interval other than percentage shall be displayed to three decimal places and minimum and maximum values shall be displayed to two decimal places.
- Sample size, number of adverse events occurred and number of subjects with adverse events will be displayed as integers, and percentage (%) and its 95% confidence interval shall be displayed to one decimal place. The 95% confidence interval for percentage (%) will be calculated as an exact confidence interval using the Clopper-Pearson method.
- Difference of percentage (%) between groups and its 95% confidence interval shall be one decimal place. The 95% confidence interval for difference in percentage (%) will be calculated as an exact confidence interval using the Santerner and Snell method.
- For calculations of confidence interval of difference between groups based on the 2-sample t-test method, if a test for equality of variance provides $p<0.05$, the 95% confidence interval by the Satterthwaite method will be used assuming that the variance is not equal, and if a test for equality of variance provides $p\geq0.05$, the 95% confidence interval by the pooled estimate will be used assuming that the variance is equal.
- The exact method shall be used for the two-sample Wilcoxon test.
- All calculations of the confidence interval shall be 2-sided 95% confidence interval.
- No significant level is defined in the tests, however, if an exploratory test method is used, a two-sided p-value of 5% is used as a guide.

- Adverse events, complications, and medical history shall be assigned the codes of system organ class (SOC), preferred term (PT), and lower level term (LLT) using the Medical Dictionary for Regulatory Activities/J dictionary (MedDRA/J). The version of MedDRA/J to be used in the analysis shall be the most recent version at the time of database lock.
- The severity of adverse events shall be determined using the Common Terminology Criteria for Adverse Events v4.0 (CTCAE v4.0) and the JCOG version translated into Japanese.
- Drug codes in the Iyakuhinmei Data File will be assigned to concomitant medications and post-treatment drugs.
- Among the adverse events, those with the relationship with investigational drug of "possible", "probable" and "definite", exluding "not related" and "unlikely", shall be considered as adverse drug reactions.
- Start date for period until onset of an adverse event shall be the start date of treatment.
  Time to adverse event = start date of adverse event - start date of treatment + 1
- In tabulation of adverse events, the results will be displayed in the order of SOC code and PT code. If the same event occurs multiple times in the same subject, the number of events shall be counted multiple times, and the number of subjects shall be counted as one subject. In tabulation by severity, if the same event occurs with different severities, the number of events shall be all counted for each severity, while the number of subjects shall be counted as one subject with the most severe event. Similarly, in tabulation by seriousness, if the same event occurred as both serious and non-serious events, the number of events shall be all counted for both serious and non-serious, while the number of subjects shall be counted as one subject with the serious event.

6.1 Subject composition

- A diagram showing the number of subjects who were secondarily enrolled as rapid progressors will be created for secondarily enrolled subjects, randomized subjects, untreated subjects, treated subjects, subjects who discontinued the study prior to Day 15, subjects who continues the study on Day 15, subjects who discontinued the study prior to Day 29 (Week 4), subjects who continues the study on Day 29 (Week 4), subjects who discontinued the study prior to Day 57 (Week 8), subjects who continues the study on Day 57 (Week 8), subjects who discontinued the study prior to Day 85 (Week 12), subjects who continues the study on Day 85 (Week 12), subjects who discontinued the study prior to Day 169 (Week 24), subjects who continues the study on Day 169 (Week 24), subjects who discontinued the study prior to Day 197 (Week 28), subjects who continues the study on Day 197 (Week 28), subjects who discontinued the study prior to Day 225 (Week 32), subjects who continues the study on Day 225 (Week 32), subjects who discontinued the study prior to Day 253 (Week 36), subjects who continues the study on Day 253 (Week 36), subjects who discontinued the study prior to Day 337 (Week 48), and subjects who continues the study on Day 337 (Week 48). Subjects who continues the study on Day X are defines as those who have at least one record of any specified test on Day X.

6.2 Subjects for analysis

- A diagram showing the sample size of the SS1, SS2, FAS and PPS for subjects who were secondarily enrolled as rapid progressors will be created by treatment group. In the diagram, the number of subjects who were excluded from the FAS will be determined by reason for exclusion.

6.3 Patient demographics

- For patient demographics in the FAS, PPS, SS1 and SS2, summary statistics will be determined for quantitative variables, and sample size and percentage (%) will be determined for qualitative variables by treatment group and in overall.

Disease duration (months) = 12 × (year at the time of consent obtained – year at the time of diagnosis) + (month at the time of consent obtained – month at the time of diagnosis) + 1

- For the SS1, the number and percentage of subjects with medical history and complications will be calculated by SOC and PT.

6.4 Treatment status

- For the SS1 and FAS, the following variables will be analyzed for the 4 periods from Day 1 to Day 14, Day 15 to Day 28, Day 29 to Week 24, and Week 25 onwards, as well as for the entire treatment period. Summary statistics will be summarized for quantitative variables and the number and percentage (%) of subjects will be summarized for qualitative variables by treatment group and in overall. For body weight at start of treatment, in principle, body weight at the initial test (screening test) shall be used; for subjects who roll over from non-progressors to rapid progressors, the most recent body weight shall be used. In addition, the drug discontinuation period shall not be included in the denominator in the calculation of the drug compliance.
  - ✓ Number of pulse therapies received (counted as 1 time if received only at start of treatment)
  - ✓ Total dose of methylprednisolone (g), total dose/body weight at start of treatment (g/kg)
  - ✓ Prednisolone compliance (based on number of days) (%), total dose (mg), total dose/body weight at start of treatment (mg/kg)
  - ✓ Total dose (mg) of methylprednisolone and prednisolone, total dose/body weight at start of treatment (mg/kg)
    If methylprednisolone and prednisolone are added together, the dose of methylprednisolone will be converted to that of prednisolone as follows:
    Methylprednisolone 1 g = Prednisolone 1250 mg
  - ✓ Presence/absence of dose increase
  - ✓ Presence/absence of discontinuation of drug
  - ✓ Presence/absence of resumption

6.5 Efficacy endpoints

The following analyses will be performed in the FAS and PPS.
Summaries will be analyzed by treatment group and in overall.

6.5.1 Primary Endpoint

- Number and percentage of subjects with "≥30% improvement in 10-meter timed walk" or "≥1 improvement in the OMDS" at Day 15 compared to the baseline values and their 95% confidence interval will be calculated. Furthermore, the difference in the percentage (%) between groups (pulse group – p.o. group) will be calculated, and when the difference in the proportion is positive, pulse therapy will be deemed to be effective compared to oral treatment. In addition, 95% confidence interval of difference in percentage (%) will be calculated, and difference between groups will be tested using Fisher's exact test.

6.5.2 Secondary Endpoints

6.5.2.1 10-meter timed walk

- Number of subjects with "≥30% improvement" at Day 15 compared to baseline and the percentage (%) and corresponding 95% confidence interval will be calculated. In addition, difference in the percentage (%) between treatment groups (pulse group – p.o. group) and its 95% confidence interval will be calculated, and difference between treatment groups will be tested using Fisher's exact test.
- Summary statistics will be calculated for area under the curve of 10-meter walk time based on baseline values, Day 15 and Day 29 (Week 4). In addition, treatment differences (pulse group – p.o. group) and their 95% confidence intervals will be calculated and tested using a 2-sample t-test.
- Summary statistics will be calculated for baseline/Day 15/Day 29 (Week 4) measurements and change from baseline. In addition, treatment differences (pulse group – p.o. group) will be calculated for the original measurements, and differences between the groups will be tested using a 2-sample Wilcoxon test, and differences and its 95% confidence intervals will be calculated for the natural-logarithmically transformed value, and differences between the groups will be tested using a 2-sample t-test.

6.5.2.2 2 minute walk distance

- Summary statistics will be calculated for area under the curve of 2-minute walk distance based on baseline values, Day 15 and Day 29 (Week 4). In addition, treatment differences (pulse group – p.o. group) and their 95% confidence intervals will be calculated and tested using a 2-sample t-test.
- Summary statistics will be calculated for baseline/Day 15/Day 29 (Week 4) measurements and change from baseline. In addition, treatment differences (pulse group – p.o. group) and their 95% confidence intervals will be calculated and tested using a 2-sample t-test.

6.5.2.3 6 minute walk distance

The same analyses as in “6.5.2.2 2 minute walk distance” will be performed.

6.5.2.4 OMDS

- Number of subjects with “≥1 improvement” at Day 15 compared to baseline and the percentage (%) and corresponding 95% confidence interval will be calculated. In addition, difference in the percentage (%) between treatment groups (pulse group – p.o. group) and its 95% confidence interval will be calculated, and difference between treatment groups will be tested using Fisher's exact test.

6.5.2.5 Cerebrospinal fluid concentration of neopterin

- Summary statistics will be calculated for baseline/Day 15 measurements and change from baseline. In addition, treatment differences (pulse group – p.o. group) will be calculated for the original measurements, and differences between the groups will be tested using a 2-sample Wilcoxon test, and differences and its 95% confidence intervals will be calculated for the natural-logarithmically transformed value, and differences between the groups will be tested using a 2-sample t-test.

6.5.2.6 Rate of subjects receiving methylprednisolone pulse therapy

- For subjects who continue the study up to Day 29 (Week 4), number and percentages (%) of subjects who received methylprednisolone pulse therapy from Day 29 (Week 4) to Day 169 (Week 24) and their 95% confidence intervals will be calculated. In addition, difference in the percentage (%) between treatment groups (pulse group – p.o. group) and its 95% confidence interval will be calculated, and difference between treatment groups will be tested using Fisher's exact test.

6.5.2.7 CXCL10 concentration

- The same analysis as for “6.5.2.4 Cerebrospinal fluid concentration of neopterin” will be performed.

6.5.3 Other Efficacy Endpoints

6.5.3.1 10-meter Timed Walk

- Summary statistics will be calculated for Day 57 (Week 8)/Day 85 (Week 12)/Day 169 (Week 24)/Day 197 (Week 28)/Day 225 (Week 32)/Day 253 (Week 36)/Day 337 (Week 48) measurements and changes from baseline. In addition, treatment differences (pulse group – p.o. group) will be calculated for the original measurements, and differences between the groups will be tested using a 2-sample Wilcoxon test, and differences and its 95% confidence intervals will be calculated for the natural-logarithmically transformed value, and differences between the groups will be tested using a 2-sample t-test.
- A graph showing time course of measured values and changes from baseline will be created by treatment group at each timepoint from baseline to Day 337 (Week 48). A graph will be created

using median (first quartile - third quartile) for the original measurement values and mean ± SE for the natural-logarithmically transformed values.

- A graph showing time course (Spaghetti plot) of measured values and changes from baseline will be created by treatment group for each subject at each timepoint from baseline to Day 337 (Week 48). It will be created for both the original measurement and the natural-logarithmically transformed value.

6.5.3.2 2 minute walk distance

- Summary statistics will be calculated for Day 57 (Week 8)/Day 85 (Week 12)/Day 169 (Week 24)/Day 197 (Week 28)/Day 225 (Week 32)/Day 253 (Week 36)/Day 337 (Week 48) measurements and changes from baseline. In addition, treatment differences (pulse group – p.o. group) and their 95% confidence intervals will be calculated and tested using a 2-sample t-test.
- A graph showing time course of measured values and changes (mean ± SE) will be created at each timepoint from baseline to Day 337 (Week 48).
- A graph showing time course (Spaghetti plot) of measured values and changes from baseline will be created by treatment group for each subject at each timepoint from baseline to Day 337 (Week 48).

6.5.3.3 6 minute walk distance

- The same analyses as in "6.5.3.2 2-minute walk distance" will be performed.

6.5.3.4 Cerebrospinal fluid concentration of neopterin

- Summary statistics will be calculated for Day 169 (Week 24)/Day 337 (Week 48) measurements and changes from baseline. In addition, treatment differences (pulse group – p.o. group) will be calculated for the original measurements, and differences between the groups will be tested using a 2-sample Wilcoxon test, and differences and its 95% confidence intervals will be calculated for the natural-logarithmically transformed value, and differences between the groups will be tested using a 2-sample t-test.
- A graph showing time course of measured values and change from baseline will be created by treatment group at each timepoint from baseline to Day 337 (Week 48). A graph will be created using median (first quartile - third quartile) for the original measurement values and mean ± SE for the natural-logarithmically transformed values.
- A graph showing time course (Spaghetti plot) of measured values and changes from baseline will be created by treatment group for each subject at each timepoint from baseline to Day 337 (Week 48). It will be created for both the original measurement and the natural-logarithmically transformed value.

6.5.3.5 Study Discontinuation Rate

- For subjects who continue the study through Day 30, the number of subjects who discontinued the study with a >100% worsening from baseline in the 10-meter walk time (based on the original measurement) from Day 30 to Day 169 (Week 24), along with the percentage and its

95% confidence interval, will be calculated. In addition, difference in the percentage (%) between treatment groups (pulse group – p.o. group) and its 95% confidence interval will be calculated, and difference between treatment groups will be tested using Fisher's exact test.

6.5.3.6 Proportion of subjects who could not discontinue the drug

- For subjects who continue the study through Day 183 (Week 26), the number and percentage (%) of subjects who are unable to discontinue the study drug until Day 183 and its 95% confidence interval will be calculated. In addition, difference in the percentage (%) between treatment groups (pulse group – p.o. group) and its 95% confidence interval will be calculated, and difference between treatment groups will be tested using Fisher's exact test.

6.5.3.7 Proportion of subjects who restart steroid treatment

- For subjects who are able to discontinue treatment at Day 183 (Week 26), the number and percentages of subjects who resumed treatment with steroids before Day 337 (Week 48) and their 95% confidence intervals will be calculated. In addition, difference in the percentage (%) between treatment groups (pulse group – p.o. group) and its 95% confidence interval will be calculated, and difference between treatment groups will be tested using Fisher's exact test.

6.5.3.8 CXCL10 concentration

- The same analysis as for "6.5.3.4 Cerebrospinal fluid concentration of neopterin" will be performed.

6.5.3.9 Use of walking aids

- Shift tables will be created for use of walking aids in the clinic on Day15 / Day29 (Week4) / Day57 (Week8)/ Day85 (Week12)/ Day169 (Week24)/ Day197 (Week28)/ Day225 (Week32)/ Day253 (Week36)/ Day337 (Week48) compared with baseline.
- Shift tables will be created for use of walking aids at home on Day15 / Day29 (Week4) / Day57 (Week8)/ Day85 (Week12)/ Day169 (Week24)/ Day197 (Week28)/ Day225 (Week32)/ Day253 (Week36)/ Day337 (Week48) compared with baseline.

6.5.3.10 IPEC1

- Summary statistics will be calculated for overall score at baseline/ Day15 / Day29 (Week4) / Day57 (Week8)/ Day85 (Week12)/ Day169 (Week24)/ Day197 (Week28)/ Day225 (Week32)/ Day253 (Week36)/ Day337 (Week48) and changes from baseline.
- Graphs showing time course of total scores and changes from baseline (mean ± SD) by treatment group will be created at each timepoint from baseline to Day 337 (Week 48).

6.5.3.11 QOL

6.5.3.11.1 Modified IPEC 2

- Summary statistics will be calculated for overall score at baseline/ Day15 / Day29 (Week4) / Day57 (Week8)/ Day85 (Week12)/ Day169 (Week24)/ Day197 (Week28)/ Day225 (Week32)/

Day253 (Week36)/ Day337 (Week48) and changes from baseline.

- Graphs showing time course of total scores and changes from baseline (mean ± SD) by treatment group will be created at each timepoint from baseline to Day 337 (Week 48).

6.5.3.11.2 N-QOL

- Summary statistics will be calculated for overall score at baseline/ Day15 / Day29 (Week4) / Day57 (Week8)/ Day85 (Week12)/ Day169 (Week24)/ Day197 (Week28)/ Day225 (Week32)/ Day253 (Week36)/ Day337 (Week48) and changes from baseline.
- Graphs showing time course of total scores and changes from baseline (mean ± SD) by treatment group will be created at each timepoint from baseline to Day 337 (Week 48).
- The 2 subscales (sleep/energy and worry/concern) and overall disturbance of daily life will also be analyzed in the same manner as the overall score.

6.5.3.11.3 Sexual Health Inventory for Men

- Shift tables will be created for individual scores in the questionnaires on Day15 / Day29 (Week4) / Day57 (Week8)/ Day85 (Week12)/ Day169 (Week24)/ Day197 (Week28)/ Day225 (Week32)/ Day253 (Week36)/ Day337 (Week48) compared with baseline.

6.5.3.12 MAS

- Shift tables will be created for MAS on Day15 / Day29 (Week4) / Day57 (Week8)/ Day85 (Week12)/ Day169 (Week24)/ Day197 (Week28)/ Day225 (Week32)/ Day253 (Week36)/ Day337 (Week48) compared with baseline.

6.5.3.13 VAS

- Summary statistics will be calculated for the VAS scores in “Global Assessment”, “Walking Assessment” and “Lower Limb Pain Assessment” at baseline/ Day15 / Day29 (Week4) / Day57 (Week8)/ Day85 (Week12)/ Day169 (Week24)/ Day197 (Week28)/ Day225 (Week32)/ Day253 (Week36)/ Day337 (Week48) and changes from baseline.
- Graphs showing time course of the VAS scores in “Global Assessment”, “Walking Assessment” and “Lower Limb Pain Assessment” and changes from baseline (mean ± SE) by treatment group will be created at each timepoint from baseline to Day 337 (Week 48).
-

6.5.3.14 Assessment of Urinary dysfunction

6.5.3.14.1 OABSS

- The overall score will be classified into categories of “≤5 (mild)”, “6 to 11 (moderate)”, or “≥12 (severe)” and shift tables will be created on Day15 / Day29 (Week4) / Day57 (Week8)/ Day85 (Week12)/ Day169 (Week24)/ Day197 (Week28)/ Day225 (Week32)/ Day253 (Week36)/ Day337 (Week48) compared with baseline.

6.5.3.14.2 ICIQ-SF

- Shift tables will be created for individual scores in the questionnaires on Day15 / Day29 (Week4) / Day57 (Week8)/ Day85 (Week12)/ Day169 (Week24)/ Day197 (Week28)/ Day225 (Week32)/ Day253 (Week36)/ Day337 (Week48) compared with baseline.

6.5.3.14.3 IPSS

- The IPSS will be classified into categories of "≤ 7 (mild)", "8 to 19 (moderate)", or "≥20 (severe)" and shift tables will be created on Day15 / Day29 (Week4) / Day57 (Week8)/ Day85 (Week12)/ Day169 (Week24)/ Day197 (Week28)/ Day225 (Week32)/ Day253 (Week36)/ Day337 (Week48) compared with baseline.

6.5.3.15 Timed up-and-go test

- Summary statistics will be calculated for the measured values at baseline/ Day15 / Day29 (Week4) / Day57 (Week8)/ Day85 (Week12)/ Day169 (Week24)/ Day197 (Week28)/ Day225 (Week32)/ Day253 (Week36)/ Day337 (Week48) and change from baseline.
- A graph showing time course of measured values and changes from baseline (mean ± SD) will be created at each timepoint from baseline to Day 337 (Week 48).

6.5.3.16 OMDS

- Shift tables will be created for the ODMS on Day15 / Day29 (Week4) / Day57 (Week8)/ Day85 (Week12)/ Day169 (Week24)/ Day197 (Week28)/ Day225 (Week32)/ Day253 (Week36)/ Day337 (Week48) compared with baseline.
- Number of subjects with "≥1 improvement" at Day 29 (Week 4)/Day 85 (Week 12)/Day 169 (Week 24)/Day 337 (Week 48) compared to baseline and the percentage (%) and corresponding 95% confidence interval will be calculated. In addition, difference in the percentage (%) between treatment groups (pulse group – p.o. group) and its 95% confidence interval will be calculated, and difference between treatment groups will be tested using Fisher's exact test.
- A graph showing time course (Spaghetti plot) will be created by treatment group for each subject at each timepoint from baseline to Day 337 (Week 48).

6.6 Safety evaluation

The following analyses will be performed in the SS1 and SS2:
Summaries will be analyzed by treatment group and in overall.

6.6.1 Secondary Endpoints

6.6.1.1 Adverse events

- For all adverse events occurred, adverse drug reactions, adverse events of Grade 2 or higher, serious adverse events, adverse events leading to discontinuation, and fetal adverse events, number of events, number of subjects and incidence rate (%) with 95% confidence interval will be calculated and summarized by treatment group. In addition, Fisher's exact test will be used to

test differences between treatment groups. Summaries will also be performed by timing of occurrence from Day1 to Day3, Day4 to Day14, Day15 to Day28, Day29 to Week24, and Week 25 onwards.

- Number of events, number of subjects and incidence rate (%) of all adverse events occurred and adverse drug reactions by SOC and PT will be calculated by treatment group. Summaries will also be performed by timing of occurrence from Day1 to Day3, Day4 to Day14, Day15 to Day28, Day29 to Week24, and Week 25 onwards.
- Number of events, number of subjects and incidence rate (%) of all adverse events occurred and adverse drug reactions by SOC and PT will be calculated for each Grade by treatment group. Similarly, analysis will be performed for each seriousness category.
-

6.6.2 Other Safety Endpoints

6.6.2.1 Clinical laboratory examination

- 定量変数については、ベースライン/ Day15 / Day29（4 週）/ Day57（8 週）/ Day85（12 週）/ Day169（24 週）/ Day197（28 週）/ Day253（36 週）/ Day337（48 週）における要約統計量を検査項目別に算出する。同様に、ベースラインからの変化量についても算出する。 For quantitative variables, summary statistics at baseline/ Day15 / Day29 (Week4) / Day57 (Week8)/ Day85 (Week12)/ Day169 (Week24)/ Day197 (Week28)/ Day225 (Week32)/ Day253 (Week36)/ Day337 (Week48) will be calculated by laboratory parameter. Similarly, change from baseline will also be calculated.
- For qualitative variables, shift tables will be created on Day15 / Day29 (Week4) / Day57 (Week8)/ Day85 (Week12)/ Day169 (Week24)/ Day197 (Week28)/ Day225 (Week32)/ Day253 (Week36)/ Day337 (Week48) compared with baseline.
- Number of subjects and percentage (%) with decrease, increase, or no increase and decrease for individual clinically significant abnormalities (ICSA) on Day15 / Day29 (Week4) / Day57 (Week8)/ Day85 (Week12)/ Day169 (Week24)/ Day197 (Week28)/ Day225 (Week32)/ Day253 (Week36)/ Day337 (Week48) will be calculated by laboratory parameter.
- ICSA shall be determined according to the criteria by the Japanese Society of Chemotherapy [1]. Table 1 shows the confirmation criteria for clinical laboratory test items in this study. Items not specified in Table 1 are not determined because there are no criteria.

Table 1 ICSA Confirmation Criteria

Items		Unit	Criteria by Japanese Society of Chemotherapy
Complete blood count	WBC	x10^3/μL	<3,000/mm^3<br>. An increase shall not be considered as an AE unless there is any special reason.<br>It can be reported as neutrophil count decreased (<1,500/mm^3) or lymphocyte decreased (<800/mm^3).
	EO	%	≥500/mm3 or ≥10%<br>In addition, this should be considered if there is any associated allergic disease.
	RBC	x10 ^{6}/μL	Males: <3.5 million/mm^3<br>; females: <3.2 million/mm^3
	Hb	g/dL	Less than 10 g/dL
	Hct	%	Males: <35%,<br>females: <30%
	PLT	x10^3 /μL	Decrease: <75,000/mm^3<br>Increase: ≥600,000/mm^3 with any symptom, or<br>In case of 1 million/mm^3 or more
Blood chemistry	BUN	mg/dL	≥1.5 times the upper limit of normal in the site
	Cr	mg/dL	
	T.BIL	mg/dL	
	Na	mEq/L	Decrease: ≤125 mEq/L Increase: ≥155 mEq/L
	K	mEq/L	Decrease: ≤3.2 mEq/L Increase: ≥5.5 mEq/L
	AST	U/L	>2.5 times the upper limit of normal in the site<br>Even if it does not exceed 2.5 times, the following cases should also be considered as adverse events.<br>・ It does not exceed 2.5 times the site reference value, but the contribution of the study drug is considered large based on the range of variation.<br>・ It does not exceed 2.5 times the site reference value, but there is an increasing trend during treatment, and the subject recovers when the effect of the study drug is no longer observed.
	ALT	U/L	
	ALP	U/L	
	Fasting blood glucose	mg/dL	Decrease: <55 mg/dL Increase: >160 mg/dL<br>(A decrease is considered as an abnormal change when it changes to <55 mg/dL with or without food.)
Urine	Urine glucose	N/A	Variation of 2 levels or more (if ± is included in qualitative value, ± is also included in 1 level)
	Urine protein		

6.6.2.2 Vital signs

- Summary statistics at baseline/ Day15 / Day29 (Week4) / Day57 (Week8)/ Day85 (Week12)/ Day169 (Week24)/ Day197 (Week28)/ Day225 (Week32)/ Day253 (Week36)/ Day337 (Week48) will be calculated by test item. Similarly, changes from baseline will also be calculated.

6.7 Lists

The following lists will be created for the subjects secondary enrolled as rapid progressors.

- A list of subjects who discontinued the study will be created including the reason for discontinuation.
- A list of subjects who are excluded from either of the SS1, SS2, FAS or PPS will be created

including the reason for exclusion

- A list of demographic information will be created.
- A list of changes in the OMDS from the onset to the date of informed consent will be created.
- A list of prior treatment for HAM will be created.
- A list of history of blood transfusion will be created.
- For the SS1, a list will be created including reported adverse event terms, SOCs, PTs, seriousness, severity, causal relationship and outcome. Similarly, lists of serious adverse events and fatal adverse events will also be created.
- A list of complications and medical history will be created.
- A list of concomitant medications and treatments will be created.
- A list of post-treatment and post-treatment regimens will be created.
- A list of treatment status table including administration status will be created for theSS1.
- A list of antibody analysis will be created.
- A list of laboratory values will be created.
- For the SS1, a list of individual clinically significant abnormal laboratory values based on ICSA determination will be created.
- A list of cerebrospinal fluid test will be created.
- A list of walking aids will be created.
- A list of 10-meter timed walk will be created.
- A list of 2-minute walk distance will be created.
- A list of 6-minute walk distance will be created.
- A list of timed up-and-go test will be created.
- A list of the OMDS will be created.
- A list of the VAS will be created.
- A list of IPEC1 will be created.
- A list of Modified IPEC 2 will be created.
- A list of MAS will be created.
- A list of OABSS will be created.
- A list of ICIQ-SF will be created.
- A list of N-QOL will be created.
- A list of IPSS will be created.
- A list of sexual function assessment will be created.
- A list of vital signs will be created.
- A list of intraocular pressure measurement will be created.
- A list of CXCL10 will be created.

6.8 Sample size calculation and power justification

Pulse arm 4 subjects, Oral prednisolone arm 4 subjects

If a total of 8 subjects are enrolled and then 3 subjects are assigned to the pulse group and 5 subjects are assigned to the p.o. group, another subject will be enrolled for assessment. Apart from this, subjects shall be enrolled as many as possible during the enrollment period.

Rationale:

Of all patients with HAM, rapid progressors are as few as approximately 5%. Furthermore, the annual number of patients who developed HAM is approximately 50 in total and only several patients are expected to meet the criteria for rapid progressor in a year. Thus, it is not possible to enroll more than 10 patients. Therefore, the number of rapid progressors in this study was set as the maximum number of subjects that can be enrolled during the enrollment period (4 in the pulse group and 4 in the p.o. group).

The primary endpoint of this study was set to be “the proportion of subjects whose 10-meter timed walk improved by ≥30%compared to baseline value on Day 15 or the OMDS improved by ≥1” based on clinical significance. Subjects with such a meaningful improvement are expected to be at least 50% in the pulse group and approximately 0 to 10% in the p.o. group. Study results will be assessed based on the number of subjects with the primary endpoint improvement. More specifically, if the number of subjects showing improvement was higher in the pulse group than in the p.o. group, the pulse therapy can be considered as more effective than the p.o. therapy. Given each group consists of 4 subjects, the probability to obtain a result indicating the higher effectiveness of the pulse rate was calculated. The calculation results are shown in Table 1 below.

This table shows the following:

- When the expected improvement ratio in the pulse group is 75% (3 out of 4), even if the expected rate in the p.o. group is 25% (1 out of 4), the probability of having a higher number of subjects with improvement in the pulse group is not less than 0.8.
- When the expected ratio in the pulse group is 50% (2 out of 4) and the expected rate in the p.o. group is not more than 10%, the probability of having a higher number of subjects with improvement in the pulse group is not less than 0.8.

Based on the above results and the expected improvement ratio in each group, it was considered possible to assess the results with 4 subjects in each group in this study.

Table 2. Probability of having a higher number of subjects with clinically meaningful improvement in the pulse group than the p.o. group with 4 subjects in each group The vertical axis (%) shows the expected ratio of subjects with improvement in the pulse group and the horizontal axis (%) shows the expected ratio of subjects with improvement in the p.o. group.

	5%	10%	15%	20%	25%
50%	0.886	0.831	0.774	0.715	0.656
55%	0.917	0.870	0.819	0.766	0.710
60%	0.941	0.902	0.859	0.812	0.761
65%	0.960	0.929	0.893	0.852	0.807
70%	0.974	0.951	0.922	0.888	0.849
75%	0.984	0.967	0.945	0.918	0.886

7 Appendix

None

8 Reference Material

1) Protocol Version 1.12 (October 08, 2020)

9 References

1. "Antimicrobial Drug Safety Evaluation Criteria" Final Report (final version) by the Japanese Society of Chemotherapy, Committee for Criteria of Safety Evaluation of Antimicrobial Drugs. Journal of the Japanese Society of Chemotherapy Vol. 58, 4/2010 (July): pp. 484 to 493

10 Revision History

Version	Date prepared	Main content	Author
1.0	November 18, 2020	Analysis plan for the final analysis	Kenichiro Tanabe

Any transfer, copying, publication, or other unauthorized reproduction of all or part of this material is prohibited without the prior consent of the Translational Research Center for Medical Innovation (TRI).

Translational Research Center for Medical Innovation.
Address: 1-5-4, Minatojimaminamimachi, Chuo-ku, Kobe, 650-0047, Japan.
TEL: +81-78-303-9107 FAX: +81-303-9094
URL: http://www.tri-kobe.org

Copyright © 2022 Translational Research Center for Medical Innovation, Foundation for Biomedical Research and Innovation at Kobe All Rights Reserved.
SOP-STAT-002-RD-01 Statistical analysis plan Template 2019.8.23
