# Supplementary material for: Efficacy of Corticosteroid Therapy for HTLV-1-Associated Myelopathy: A Randomized Controlled Trial (HAMLET-P)
Source: Viruses. 2022 Jan 12;14(1):136. doi: 10.3390/v14010136 (PMC8780460; doi:10.3390/v14010136)
Supplement: Supplementary file 1 [file viruses-14-00136-s001.zip › Appendix C_StatisticalAnalysisPlan_Slow_final.pdf]

Confidential

Phase IIb clinical trial of steroid therapy in patients with HAM/TSP Slow Progressor HAMLET-P（TRINEU1604）

Statistical Analysis Plan
Version 1.0

Date 2020/11/18

Chief Statistician: Kenichiro Tanabe

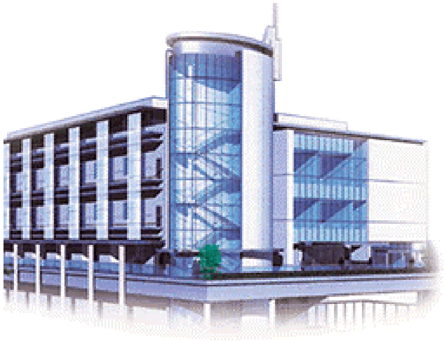

Translational Research Center for Medical Innovation
founded in 2003 by MEXT & Kobe City
for acceleration of translational research in Japan

Copyright © 2022 Translational Research Center for Medical Innovation, Foundation for Biomedical Research and Innovation at Kobe All Rights Reserved.

Table of contents

1 Basic items related to analysis 1
1.1 Study Objectives and Research Question 1
1.2 Study flow chart 2
1.3 Summary of Research 4
1.4 Storage location of data 9
1.5 Analysis software 9
2 Objectives of Analysis 10
2.1 Changes to the analysis method from the protocol 10
3 Analysis Set 11
3.1 Efficacy Analysis Population 11
3.1.1 FAS 11
3.1.2 PPS 11
3.2 Safety Analysis Population 11
3.2.1 SS1 11
3.2.2 SS2 11
4 Endpoint Definitions 12
4.1 Efficacy Endpoint 12
4.1.1 Primary Endpoint 12
4.1.2 Secondary Endpoints 12
4.1.3 Other Efficacy Endpoints 12
4.2 Safety Evaluation 14
4.2.1 Secondary Endpoints 14
4.2.2 Other Safety Endpoints 15
5 Data Handling 17
5.1 Handling of timepoint allowable range (allowance) 17
5.2 Data handling at discontinuation 17
5.3 Handling of missing value 17
5.3.1 10-meter timed walk 17
5.3.2 6-minute walk distance 17
5.3.3 IPEC1 17
5.3.4 Modified IPEC 2 17
5.3.5 N-QOL 18
5.3.6 OABSS 18

Copyright © 2022 Translational Research Center for Medical Innovation, Foundation for Biomedical Research and Innovation at Kobe All Rights Reserved.

5.3.7 ICIQ-SF 18
5.3.8 IPSS 18
5.3.9 Month of diagnosis 18
5.4 Handling of limit of detection in laboratory values 18
5.5 Handling of outliers or abnormal values 18
5.6 Definition of the baseline 19
5.6.1 10-meter timed walk 19
5.6.2 Cerebrospinal fluid concentration of neopterin 19
5.6.3 CXCL10 concentration 19
5.6.4 Other items 19
6 Analysis methods 20
6.1 Subject composition 21
6.2 Subjects for analysis 22
6.3 Patient demographics 22
6.4 Treatment status 22
6.5 Efficacy endpoints 23
6.5.1 Primary Endpoint 23
6.5.2 Secondary Endpoints 24
6.5.3 Other Efficacy Endpoints 26
6.6 Safety evaluation 29
6.6.1 Secondary Endpoints 29
6.6.2 Other Safety Endpoints 29
6.7 Lists 32
6.8 Sample size calculation and power justification 34
7 Appendix 36
8 Reference Material 36
9 References 36
10 Revision History 37

Copyright © 2022 Translational Research Center for Medical Innovation, Foundation for Biomedical Research and Innovation at Kobe All Rights Reserved.

Abbreviations

Abbreviation	Unabbreviated term
ADS	analysis dataset
ALP	alkaline phosphatase
ALT	alanine aminotranferase
ANCOVA	analysis of covariance
AST	aspartate aminotransferase
BA	basophil
BUN	blood urea nitrogen
Ca	calcium
Cr	creatinine
CTCAE v4.0	common terminology criteria for adverse events v4.0
CXCL10	C-X-C motif chemokine ligand 10
DM	data manager
EDC	Electronic Data Capture
EO	eosinophil
FAS	full analysis set
HAM	HTLV-1-associated myelopathy
Hb	hemoglobin
HbA1c	hemoglobin A1c
HDL-C	high-density nipoprotein cholesterol
Hct	hematocrit
HTLV-1	human T-lymphotropic virus type 1
ICIQ-SF	international consultation on incontinence questionnaire-short form
ICSA	individual clinically significant abnormalities
IP	inorganic phosphorus
IPEC1	-
IPSS	international prostate symptom score
K	kalium
LDL-C	low-density lipoprotein cholesterol
LLT	lowest level terms
LOCF	last observation carried forward
log 10 mWT	logarithm of 10-m walking test
LSMean	least square mean
Lym	lymphocyte
MAS	modified Ashworth scale
MCH	mean corpuscular hemoglobin
MCHC	mean corpuscular hemoglobin concentration
MCV	mean corpuscular volume

Copyright © 2022 Translational Research Center for Medical Innovation, Foundation for Biomedical Research and Innovation at Kobe All Rights Reserved.

Abbreviation	Unabbreviated term
MedDRA/J	medical dictionary for regulatory activities/J
MMRM	mixed model repeated measures
MO	monocyte
Modified IPEC 2	-
MRI	magnetic resonanse imaging
Na	natrium
NE	neutrophil
N-QOL	nocturia quality of life questionnaire
OABSS	overactive bladder symptom score
OMDS	Osame's motor disability score
PLT	platelet
PPS	per protocol set
PT	preferred terms
QOL	quality of life
RBC	red blood cell
SE	standard error
SS1	safety set 1
SS2	safety set 2
SOC	system organ class
T-Cho	total cholesterol
TG	triglyceride
VAS	visual analog scale
WBC	white blood cell
T.BIL	total bilirubin

Copyright © 2022 Translational Research Center for Medical Innovation, Foundation for Biomedical Research and Innovation at Kobe All Rights Reserved.

1 Basic items related to analysis

1.1 Study Objectives and Research Question

<u>Primary objective</u>

To test the efficacy of oral prednisolone therapy in patients with slowly progressive HAM/TSP.

<u>Secondary objective</u>

To test the safety of of oral prednisolone therapy in patients with slowly progressive HAM/TSP.

Research Question:

① In a randomized controlled study in the slow progressors among HAM patients, does oral prednisolone treatment have superior efficacy effect compared to placebo?

② Is it safe to treat the slow progressors among HAM patients with prednisolone?

Estimand:

1. Target population: Full analysis set (FAS) (see Section 3.1.1)
2. Variable (or endpoint): Change from baseline in the 10-meter timed walk at Day 169 (Week 24) (see Section 4.1.1)
3. Considerations for interim events: See section 5.3.1 for details
   i) Data may also be collected after discontinuation of study treatment for the purpose of collecting prognostic data. However, data after discontinuation of study treatment will be handled as missing as the primary endpoint analysis will be performed for efficacy evaluation of study treatment at Day 169 (Week 24). Therefore, the effect of post-treatment, etc. will not be considered.
   ii) Last observation carried forward (LOCF) will be defined as the data set corresponding to the method of processing missing values for sensitivity analysis of the results of the main analysis. The LOCF is a data set in which data obtained at the specified timepoint, and missing value data not obtained at the specified timepoint will be imputed with data most recently obtained.
4. Summary of variables at the population level

   Null hypothesis: There are no differences in the changes of the 10-meter timed walk on Day 169 (Week 24) from baseline to Day 169 (Week 24) between the prednisolone and placebo groups

   The null hypothesis will be rejected using a two-sided type I error of 5%.

Copyright © 2022 Translational Research Center for Medical Innovation, Foundation for Biomedical Research and Innovation at Kobe All Rights Reserved.

1.2 Study flow chart

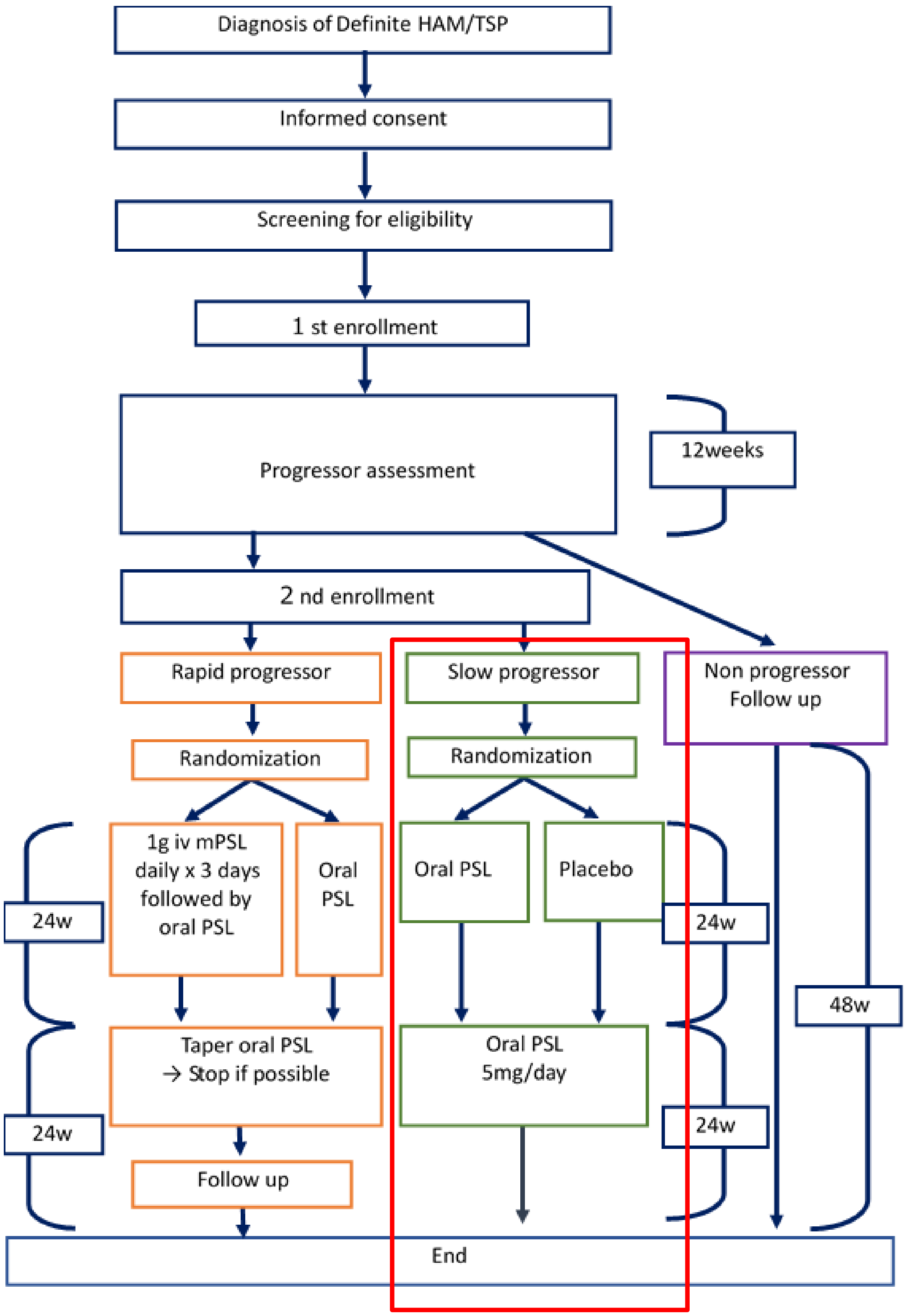

Copyright © 2022 Translational Research Center for Medical Innovation, Foundation for Biomedical Research and Innovation at Kobe All Rights Reserved.

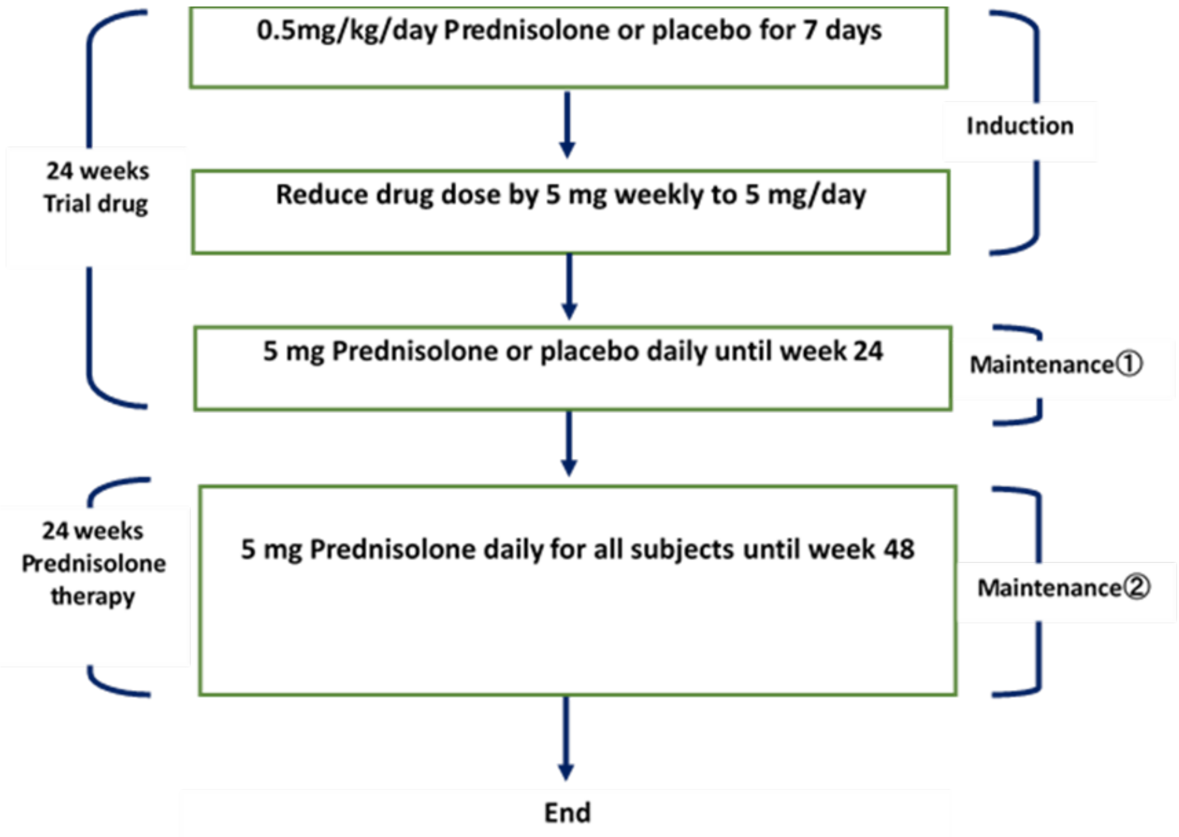

Copyright © 2022 Translational Research Center for Medical Innovation, Foundation for Biomedical Research and Innovation at Kobe All Rights Reserved.

1.3 Summary of Research

Target sample size	40 subjects
Subject enrollment period	August 2016 to March 2019 (2 years and 8 months)
Follow-up end date	(Secondary enrollment) One year (48 weeks) after the last subject enrollment
Observation/intervention	Interventional Research
Phase of the study	Phase IIb
Control Type	Placebo-controlled study<br>Study group: Treated with oral prednisolone<br>Control group: Treated with oral placebo
Randomization	Randomization study with minimization method<br>Randomization adjustment factors:<br>• Sex: Male, Female<br>• Number of canes: One or less/Two or more<br>(A walker should be regarded as “two or more”.)<br>• Study site
Blinding Level	Double-blind

Copyright © 2022 Translational Research Center for Medical Innovation, Foundation for Biomedical Research and Innovation at Kobe All Rights Reserved.

Schedule Table：

Period		Screening	1st Enrollment*1	Progressor Assessment				2nd Enrollment*2	Study drug treatment				Prednisolone treatment				Unplanned Visits*20	Post-observation*4 +28	At discontinuation*5
Week		-16 ～ - 12*1		-12*1	-8	-4	Last assessment *2 ±7		0	4	12	24*3	28	32	36	48			
Day		-112～-84 ±7		-84 ±7	-56 ±7	-28 ±7			1*2	29 ±7	85 ±7	169 ±7	197 ±7	225 ±7	253 ±7	337 ±7			
Informed consent		x						Randomization											
Eligibility confirmation		x																	
Study drug treatment									Prednisolone or Placebo				Prednisolone						
Clinical Assessment	Physical examination	x		x	x	x	x			x	x	x	x	x	x	x		x	x
	Blood pressure, Pulse rate, Body temperature	x		x	x	x	x			x	x	x	x	x	x	x			x*6
	Height, Body weight	x										x*7				x*7			
	OMDS	x		x	x	x	x			x	x	x	x	x	x	x	x		x
	Walking tests *A	x		x	x	x	x			x	x	x	x	x	x	x	x		x*8
	Walking aids *B	x		x	x	x	x			x	x	x	x	x	x	x	x		
	MAS	x		x	x	x	x			x	x	x	x	x	x	x			
	IPEC1	x		x	x	x	x			x	x	x	x	x	x	x			
	VAS *C	x		x	x	x	x			x	x	x	x	x	x	x			
	QOL*D	x		x	x	x	x			x	x	x	x	x	x	x			
	Urinary dysfunction*E	x		x	x	x	x			x	x	x	x	x	x	x			
Blood tests*F/ Urinalysis*G		x		x	x	x	x			x	x	x	x			x			x

Copyright © 2022 Translational Research Center for Medical Innovation, Foundation for Biomedical Research and Innovation at Kobe All Rights Reserved.

Period		Screening	1st Enrollment*1	Progressor Assessment				2nd Enrollment*2	Study drug treatment				Prednisolone treatment				Unplanned Visits*20	Post-observation*4	At discontinuation*5
Week		-16 ～ - 12*1		-12*1	-8	-4	Last assessment *2		0	4	12	24*3	28	32	36	48			
Day		-112～-84 ±7		-84 ±7	-56 ±7	-28 ±7	±7		1*2	29 ±7	85 ±7	169 ±7	197 ±7	225 ±7	253 ±7	337 ±7		+28	
Virus tests*14*H		x*9								x*11	x*10	x *10	x*11			x*11			x *12
Pregnancy test*13		x					(x)												
Cerebrospinal fluid tests*14 *I		x*15					x			(x)		x		(x) *16	(x) *16	x			
Accompanying research	Blood tests*J	x		x	x	x	x		x	x	x	x	x			x			
Accompanying research	Biobank samples (Blood)*17	x		x	x	x	x		x	x	x	x	x			x			
Accompanying research	Cerebrospinal Fluid tests *K	x*15					x			(x)		x		(x) *16	(x) *16	x			
Accompanying research	Biobank samples (Cerebrospinal fluid) *17	x*15					x			(x)		x		(x) *16	(x) *16	x			
MRI		x*18		x*18						(x)		x				(x)			
Intraocular pressure		x*18		x*18						x	x *19 x *19	x *19			x *19	x *19			

＊1: The progressor assessment period on Week -12 and screening can be performed on the same day. In this case, the items specified in the screening should be performed. In addition, the primary enrollment should occur within 7 days of screening.

＊2: Secondary enrollment should be performed within 7 days of the last assessment date to start Day 1 (study drug treatment). The date may be the same as the date of the final assessment.

Copyright © 2022 Translational Research Center for Medical Innovation, Foundation for Biomedical Research and Innovation at Kobe All Rights Reserved.

＊3: For subjects who discontinue the study due to clinical exacerbation of HAM, data will be collected on “details of the post-treatment regimen (for treatment of HAM [including the drugs listed in Exclusion Criterion 1 and Exclusion Criterion 6 and treatment with HAL], “10-meter timed walk”, and “Osame’s Motor Disability Score” until the period corresponding to Week 24 in the study treatment period.

＊4: To be performed 28 days (+28 days) after the final dose of the study drug.

＊5: To be performed within 28 days after discontinuation of the study drug during the treatment period or before the start of post-treatment, whichever is earlier.

＊6: Body temperature will be measured as needed.

＊7: Height measurement is not required.

＊8: 10-meter timed walk, 6-minute walk distance, and 2-minute walk distance will be performed. Timed up-and-go test is not required.

＊9: HBs antigen, HBs antibody, HBc antibody, HCV antibody, HIV-1 antibody and HIV-2 antibody will be tested only at screening. If either HBs antibody or HBc antibody is positive, quantitative HBV-DNA measurement will be performed.

However, quantitative HBV-DNA is not required if the positive HBs antibody is clearly due to vaccination.

*10: Quantitative HBV-DNA measurement will be performed only in subjects who are positive for HBs or HBc antibodies (with the exception of positive HBs antibody clearly due to vaccination) at screening and whose quantitative HBV-DNA is below the detection sensitivity.

*11: Subjects who are positive for HBs or HBc antibodies (with the exception of positive HBs antibody clearly due to vaccination) at screening and whose quantitative HBV-DNA is below the detection sensitivity will be closely monitored for liver function tests, and if abnormal values are observed (if AST and ALT increase is ≥ 5 times the reference value at the site), quantitative HBV-DNA measurement will be performed.

*12: For subjects who are positive for HBs or HBc antibodies (with the exception of positive HBs antibody clearly due to vaccination) at screening and whose quantitative HBV-DNA is below the detection sensitivity, if the liver function test shows abnormal values (if AST and ALT increase is ≥ 5 times the reference value at the site), quantitative HBV-DNA measurement will be performed.

*13: It is not required for subjects who are permanently sterilized. (Permanent sterilization: postmenopausal [with no menses for at least 48 weeks with no other medical reason], post sterilization [hysterectomy, bilateral salpingectomy, bilateral oophorectomy], tubal occlusion without tubal ligation)

*14: If data is available from a previous test on anti-HTLV-1 antibody for diagnosis of HAM, that data may be used.

*15: Data obtained within 12 weeks prior to the date of consent may be used.

*16: When increasing prednisolone treatment due to exacerbation, it is recommended to perform a cerebrospinal fluid test before.

*17: Residual samples will be used as samples for biobanking.

Copyright © 2022 Translational Research Center for Medical Innovation, Foundation for Biomedical Research and Innovation at Kobe All Rights Reserved.

*18: MRI and intraocular pressure measurement will be performed at any visit from screening to the progressor assessment period and the data will be used as the baseline value. For MRI scans, data obtained within 12 weeks prior to the date of informed consent can be used.

However, if an image used as the baseline shows findings suggestive of inflammation in the spinal cord, a retest must be performed within 12 weeks prior to the secondary enrollment for that site only. In addition, if data obtained within 12 weeks prior to the date of informed consent at another hospital is available and indicates no findings of inflammation in the spinal cord, a retest is not required.

*19: To be performed only in subjects with glaucoma at week 8, 12, 24, 36, and 48

*20: Unscheduled visit is allowed to confirm the extent of disease progression. At the unscheduled visit, only the Osame's Motor Disability Score, the walk test (10-meter timed walk), and the use of walking aids will be performed.

*A：Waling tests (10 meter Timed Walk, 6 minute Walk Distance, 2 minute Walk Distance, Timed Up and Go Test)

*B：Ambulatory aid used at the clinic and home

*C：VAS（Overall, Walking, Lower extremity pain）

*D：QOL (Modified IPEC2, N-QOL, Sexual Health Inventory for Men）

*E：Urinary dysfunction (OABSS, ICIQ-SF, IPSS)

*F：Blood tests： Complete blood count with differential, Urea nitrogen and electrolytes (BUN, Cre, Na, K), Liver function (AST, ALT, ALP, T-Bil),
Glucose metabolism (HbA1c (NGSP), Fasting blood glucose), Bone metablism (Ca, P), Lipids (T-Cho, LDL-C, HDL-C, TG)

*G：Urinalysis: glucose, protein

*H：Virus tests: Hepatitis B virus (HBc antibody ((at screening only), HBs antibody (at screening only), HBs antigen (at screening only), HBV-DNA)
HCV antibody (at screening only), HIV-1 antibody･HIV-2 antibody (at screening only)
Anti-HTLV-1 antibody for HAM diagnosis (at screening only)

*I：Cerebrospinal fluid tests: Anti-HTLV-1 antibody for HAM diagnosis (at screening only)
Safety: Glucose
Efficacy: Cell counts/ Cell fractionation, Toral protein, neopterin

*J：Blood tests (Accompanying research): β2-microgloblin, B- and T-cell subsets/markers、sIL-2R、HTLV-1 proviral load, sVCAM-1、SPARC

*K：Cerebrospinal fluid tests (Accompanying research): HTLV-1 anribody, HTLV-1 proviral load, CXCL10, sVCAM-

Copyright © 2022 Translational Research Center for Medical Innovation, Foundation for Biomedical Research and Innovation at Kobe All Rights Reserved.

1.4 Storage location of data

Data provided by the data manager (DM) and analysis data set (ADS) used for the analysis are stored below.

DM Dataset: (After secondary enrollment)	Z:¥¥01_Project¥¥TRIProject 2¥¥259_TRINEU1604(Yamano) ¥¥09_Biostatistics¥¥03_Final analysis¥¥2_ANALYSIS¥¥EXECUTE1¥¥01_DATA ¥¥DM_RAW¥¥20161209_Dummy¥¥SASDS
DM Dataset: (Before secondary enrollment)	Z:¥¥01_Project¥¥TRIProject2¥¥260_TRINEU1605(Yamano) ¥¥09_Biostatistics¥¥03_Final analysis¥¥2_ANALYSIS¥¥EXECUTE1¥¥01_DATA ¥¥DM_RAW¥¥20161209_Dummy¥¥SASDS
ADS :	Z:¥¥01_Project¥¥TRIProject 2¥¥259_TRINEU1604(Yamano) ¥¥09_Biostatistics¥¥03_Final analysis¥¥2_ANALYSIS¥¥EXECUTE1¥¥01_DATA ¥¥ADS¥¥Main

1.5 Analysis software

Analysis will be performed using SAS Version 9.4 (SAS Institute Inc., Cary, NC, USA).

Copyright © 2022 Translational Research Center for Medical Innovation, Foundation for Biomedical Research and Innovation at Kobe All Rights Reserved.

2 Objectives of Analysis

This Statistical Analysis Plan (hereinafter referred to as the “SAP”) provides details of the final analyses as defined in the Protocol [1)] (Version 1.12).

2.1 Changes to the analysis method from the protocol

Changes to the analysis method from the Protocol[1)] (Version 1.12) are shown below.

Details before change	Details after change	Reason for change
8. Study design and evaluation (Slow)	Added C-X-C motif chemokine ligand 10 (CXCL10) concentration not subject to electronic data capture (EDC) to the secondary efficacy endpoints of <comparison of active treatment group and placebo> and <comparison in placebo group>.	Because, in consultation with the coordinating investigator, it was considered that CXCL10 concentration should be evaluated in the same way as cerebrospinal fluid concentration of neopterin.
8. Study design and evaluation (Slow)	Added the change from baseline to timepoints after Day 169 (Week 24) in 10-meter timed walk, 2-minute walk distance, 6-minute walk distance, cerebrospinal fluid concentration of neopterin and CXCL10 concentration.	Since it was considered that the timepoints after Day 169 (Week 24) should also be assessed.

Copyright © 2022 Translational Research Center for Medical Innovation, Foundation for Biomedical Research and Innovation at Kobe All Rights Reserved.

3 Analysis Set

3.1 Efficacy Analysis Population

The FAS and the per protocol set (PPS) will be defined as the population for the analysis of efficacy. The FAS will be used for analyses of the primary efficacy results, and the PPS will be used for analyses of the secondary efficacy results.

3.1.1 FAS

A population of all subjects secondary enrolled as a slow progressor and randomized, excluding the subjects listed below. It will be used for analyses of the primary efficacy results.

- Subjects who have never received study treatment
- Subjects who do not have a pre-treatment value of the primary endpoint (10-meter timed walk)
- Subjects who do not have at least one value of the primary endpoint (10-meter timed walk) during the study period (through Week 24)
- Subject who is subsequently found to have violated the eligibility criteria

3.1.2 PPS

Subjects in the FAS who have no major protocol deviations. It will be used for analyses of the secondary efficacy results. Serious deviations will be determined by the time the database is locked.

3.2 Safety Analysis Population

The Safety Set 1 (SS1) and Safety Set 2 (SS2) will be defined as the safety analysis sets. The SS1 will be used for analyses of the primary safety results, and the SS2 will be used for the secondary safety results.

3.2.1 SS1

A population of all subjects secondary enrolled as a slow progressor who have received at least one dose of study treatment. It will be used for analyses of the primary safety results

3.2.2 SS2

A population of subjects included in the SS1 excluding those who is found not to meet any of the inclusion criteria or to meet any of the exclusion criteria after secondary enrollment as a slow progressor. It will be used for analyses of the secondary safety results.

Copyright © 2022 Translational Research Center for Medical Innovation, Foundation for Biomedical Research and Innovation at Kobe All Rights Reserved.

4 Endpoint Definitions

4.1 Efficacy Endpoint

4.1.1 Primary Endpoint

Change in 10-meter walking test at Day169 (Week 24) from baseline

4.1.2 Secondary Endpoints

4.1.2.1 10-meter timed walk

- Change at Day29 (Week 4)/ Day85 (Week 12) compared to baseline
- Change at Day169 (Week 24) compared to baseline and at Day337 (Week 48) compared to Day169 (Week 24) in the placebo arm

4.1.2.2 2-minute walk distance

- Change at Day29 (Week 4) / Day85 (Week 12)/ Day169 (Week 24) compared to baseline
- Change at Day169 (Week 24) compared to baseline and at Day337 (Week 48) compared to Day169 (Week 24) in the placebo arm

4.1.2.3 6-minute walk distance

- Change at Day29 (Week 4) / Day85 (Week 12)/ Day169 (Week 24) compared to baseline
- Change at Day169 (Week 24) compared to baseline and at Day337 (Week 48) compared to Day169 (Week 24) in the placebo arm

4.1.2.4 Cerebrospinal fluid concentration of neopterin

- Change at Day169 (Week 24) compared to baseline
- Change at Day169 (Week 24) compared to baseline and at Day337 (Week 48) compared to Day169 (Week 24) in the placebo arm

4.1.2.5 Subjects who discontinued the study drug

- Proportion of subjects who discontinued the study drug during the study drug treatment priod (Day 1 ~ Day169 (Week 24))

4.1.2.6 Cerebrospinal fluid concentration of CXCL10

- Change at Day169 (Week 24) compared to baseline
- Change at Day169 (Week 24) compared to baseline and at Day337 (Week 48) compared to Day169 (Week 24) in the placebo arm

4.1.3 Other Efficacy Endpoints

4.1.3.1 10-meter timed walk

- Change at Day197 (Week 28)/ Day225 (Week 32)/ Day253 (Week 36)/ Day337 (Week 48) from

Copyright © 2022 Translational Research Center for Medical Innovation, Foundation for Biomedical Research and Innovation at Kobe All Rights Reserved.

baseline

4.1.3.2 2-minute walk distance

- Change at Day197 (Week 28)/ Day225 (Week 32)/ Day253 (Week 36)/ Day337 (Week 48) from baseline

4.1.3.3 6-minute walk distance

- Change at Day197 (Week 28)/ Day225 (Week 32)/ Day253 (Week 36)/ Day337 (Week 48) from baseline

4.1.3.4 Cerebrospinal fluid concentration of neopterin

- Change at Day337 (Week 48) from baseline

4.1.3.5 Cerebrospinal fluid concentration of CXCL10

- Change at Day337 (Week 48) from baseline

4.1.3.6 Use of walking aids

- Ambulatory aid used at the clinic
- Ambulatory aid used at home

4.1.3.7 IPEC1

- Total score

4.1.3.8 QOL：quality of life

4.1.3.8.1 Modified IPEC 2

- Total score

4.1.3.8.2 N-QOL：nocturia quality of life questionnaire

Since the response scale for each question ranges from 4 (lowest QOL) to 0 (highest QOL), the points are reversed so that the scale ranges from 0 (lowest QOL) to 4 (highest QOL). Subsequently, a score will be calculated using the formula below in a manner where the highest QOL scores 100 points out of 100. If the response to Q8 “Concerned that I am disturbing others in the house because of having to get up at night to urinate” is “There is no family or others in the house,” this will be handled as a missing value for the question.

- Overall score
  - ✓ If all items (12 questions) have been answered:
    Total points of the items × 100/48
  - ✓ When there is 1 missing value and only 11 questions have been answered:
    Total points of the items × 100/44
- Subscales

Copyright © 2022 Translational Research Center for Medical Innovation, Foundation for Biomedical Research and Innovation at Kobe All Rights Reserved.

- ✓ Sleep/Energy
  Total points of the subscale items × 100/24
- ✓ Bother/Concern
  Total points of the subscale items × 100/24
- Overall health status
  Q13: Overall disturbance of daily life

4.1.3.8.3 Sexual Health Inventory for Men
- Individual score of question items

4.1.3.9 MAS：modified Ashworth scale
- Scale

4.1.3.10 VAS：visual analog scale
- Global assessment
- Walking assessment
- Lower extremity pain assessment

4.1.3.11 Urinary dysfunction

4.1.3.11.1 OABSS：overactive bladder symptom score
- Total score

4.1.3.11.2 ICIQ-SF：international consultation on incontinence questionnaire-short form
- Individual score of question items

4.1.3.11.3 IPSS：international prostate symptom score
- Total score

4.1.3.12 Time up and go test
- Time (second)

4.1.3.13 OMDS：Osame's motor disability score
- Grade

4.2 Safety Evaluation

4.2.1 Secondary Endpoints

4.2.1.1 Adverse events
- Occurrence status (frequency, severity)

However, events that occurred more than 28 days after the final dose of the study drug in subjects

Copyright © 2022 Translational Research Center for Medical Innovation, Foundation for Biomedical Research and Innovation at Kobe All Rights Reserved.

who discontinued the study, or those occurred more than 28 days after the last observation day at Week 48 (the last day if the 48-week visit measurement occurred for more than one day) in subjects who completed the study will not be included in the analysis.

4.2.2 Other Safety Endpoints

4.2.2.1 Clinical laboratory examination

- Complete blood count
  - ✓ WBC：white blood cell
  - ✓ WBC differential
  - ✓ RBC：red blood cell
  - ✓ Hb：hemoglobin
  - ✓ Hct：hematocrit
  - ✓ MCV：mean corpuscular volume
  - ✓ MCH：mean corpuscular hemoglobin
  - ✓ MCHC：mean corpuscular hemoglobin concentration
  - ✓ PLT：platelet
- Blood chemistry
  - ✓ BUN：blood urea nitrogen
  - ✓ Cr：creatinine
  - ✓ Na：natrium
  - ✓ K：kalium
  - ✓ AST：aspartate aminotransferase
  - ✓ ALT：alanine aminotranferase
  - ✓ ALP：alkaline phosphatase
  - ✓ T.BIL：total bilirubin
  - ✓ HbA1c：hemoglobin A1c
  - ✓ Fasting blood glucose
  - ✓ Ca：calcium
  - ✓ IP：inorganic phosphorus
  - ✓ T-Cho：total cholesterol
  - ✓ LDL-C：low-density lipoprotein cholesterol
  - ✓ HDL-C：high-density lipoprotein cholesterol
  - ✓ TG：triglyceride
- Urinalysis (Urine glucose, Urine protein)

4.2.2.2 Vital signs

- Body weight

Copyright © 2022 Translational Research Center for Medical Innovation, Foundation for Biomedical Research and Innovation at Kobe All Rights Reserved.

- Blood pressure (systolic, diastolic)
- Pulse rate
- Body temperature

Copyright © 2022 Translational Research Center for Medical Innovation, Foundation for Biomedical Research and Innovation at Kobe All Rights Reserved.

5 Data Handling

5.1 Handling of timepoint allowable range (allowance)

For data at scheduled visits, the FAS will, in principle, accept the any range of timepoints, but if data is outside the range or if there are multiple values within the range, the handling will be determined after discussion with the coordinating investigator and the principal investigator. Data handling in the PPS, SS1, and SS2 will also be determined after discussion with the coordinating investigator and the principal investigator.

5.2 Data handling at discontinuation

Data will be included in lists. If data is included in the timepoint allowable range, then it may be used for analysis.

5.3 Handling of missing value

All efficacy data after study treatment discontinuation will be treated as missing. Missing value will remain missing for items not listed below.

5.3.1 10-meter timed walk

- In principle, the mean of two measurements will be used as the value at the timepoint; however, if only one measurement has been taken (missing for one measurement), the measurement will be used as is for evaluation.
- Sensitivity analyses for missing value will be performed for the primary analysis of the primary endpoint (see Section 6.5.1 Primary Endpoint). For this sensitivity analysis, data up to Day 169 (Week 24) will be imputed by the LOCF. However, baseline data will not be used to impute missing values.

5.3.2 6-minute walk distance

- If the test is suspended within 2 minutes, the 6-minute walk distance will be missing on the EDC data. In this case, the 2-minute walk distance value will be imputed as the 6-minute walk distance.

5.3.3 IPEC1

- If at least 1 of all the questions (11 questions) of “motor score”, “spasm score”, “perceptive score”, and “sphincter muscle score” is missing, the overall score will be missing.

5.3.4 Modified IPEC 2

- If at least 1 of all the questions (8 questions) of “body movement” and “bladder and bowel movements” is missing, the overall score will be missing.

Copyright © 2022 Translational Research Center for Medical Innovation, Foundation for Biomedical Research and Innovation at Kobe All Rights Reserved.

5.3.5 N-QOL

- If the answer in Q8 is "No family members or cohabiters", Q8 will be missing.
- The overall score will be calculated only if all 12 questions have been answered from Q1 through Q12 (not considering Q13) or if only 11 questions have been answered with one missing question. Thus, if 2 or more questions from Q1 to Q12 are missing, the overall score will be missing.
- The subscale "sleep/energy" will only be calculated if all 6 questions in Q1, Q2, Q3, Q4, Q5, and Q7 have been answered. Thus, if at least 1 of the 6 questions in Q1, Q2, Q3, Q4, Q5, and Q7 is missing, the subscale "sleep/energy" will be missing.
- The subscale of "worry/concern" will be only calculated if all 6 questions in Q6, Q8, Q9, Q10, Q11, and Q12 have been answered. In other words, if at least 1 of the 6 questions in Q6, Q8, Q9, Q10, Q11, and Q12 is missing, the subscale of "worry/concern" will be missing.

5.3.6 OABSS

- The overall score will only be calculated if all Q1 through Q4 are answered. Thus, if at least one of Q1 through Q4 is missing, the overall score will be missing.

5.3.7 ICIQ-SF

- For Q4 (When do you have urine leakage?), if the first item of (None - No urine leakage) is checked (data shows a score of 1), all items 2 through 8 of Q4 will be imputed with 0 (None) for the analysis.

5.3.8 IPSS

- The IPSS (sum of the scores in Q1 to Q7) will only be calculated if all Q1 to Q7 responses have been made. Thus, if at least 1 question is missing from Q1 to Q7, IPSS will be missing.

5.3.9 Month of diagnosis

- When calculating disease duration, if the month of diagnosis is missing, 'January' will be imputed. However, the data will be considered as missing in lists.

5.4 Handling of limit of detection in laboratory values

For summary statistics of laboratory values, the site's limit will be used as the measurement value for laboratory values that are considered to be the limit of detection. However, the data will be considered as missing in lists, and will be shown so that it can be identified as below the quantification limit.

5.5 Handling of outliers or abnormal values

As a general rule, outliers or abnormal values will not be processed. However, if there is any

Copyright © 2022 Translational Research Center for Medical Innovation, Foundation for Biomedical Research and Innovation at Kobe All Rights Reserved.

problem, the handling of such problem shall be determined through consultation with the coordinating investigator and the principal investigator.

5.6 Definition of the baseline

5.6.1 10-meter timed walk

- This will be the final assessment (assessment at the time of the last progressor assessment).

5.6.2 Cerebrospinal fluid concentration of neopterin

- In principle, the final assessment is made before the start of treatment, but if a subject rolls over from a non-progressor and it is considered that there is a problem with the period from the final assessment, the handling should be determined after discussion with the coordinating investigator and the principal investigator, including data obtained within 12 weeks prior to obtaining consent.

5.6.3 CXCL10 concentration

- Same as "5.6.2 Cerebrospinal fluid concentration of neopterin".

5.6.4 Other items

- In principle, the final assessment is made before the start of treatment, but if a subject rolls over from the non-progressor and it is considered that there is a problem with the period from the final assessment, the handling should be determined after discussion with the coordinating investigator and the principal investigator.

Copyright © 2022 Translational Research Center for Medical Innovation, Foundation for Biomedical Research and Innovation at Kobe All Rights Reserved.

6 Analysis methods

General handling of statistical analyses will be described. If specific handling is defined, it should be prioritized.

- In principle, in calculation of summary statistics (sample size, mean, standard deviation, minimum, first quartile, median, third quartile, and maximum values), the number of decimal places for values of mean, standard deviation, first quartile, median, and third quartile shall be 1 digit more than the maximum number of digits of data. Similarly, the number of decimal places for values of differences of mean values between groups, 95% confidence interval other than percentage, least square mean (LSMean), standard error (SE) for LSMean, and 95% confidence interval for LSMean shall also be 1 digit more than the maximum number of digits of data. The number of decimal places for minimum and maximum values shall be the same as the maximum number of digits of data.
- Summary statistics for each timepoint for 10-meter timed walk, cerebrospinal fluid concentration of neopterin, and CXCL10 concentration will be derived for both the original measurement and the natural-logarithmically transformed value. Mean, standard deviation, first quartile, median and third quartile of 10-meter timed walk shall be displayed to three decimal places. Similarly, differences of mean values between groups, 95% confidence interval other than percentage, LSMean, SE for LSMean, and 95% confidence interval for LSMean shall also be displayed to three decimal places. Minimum and maximum shall be displayed to two decimal places.
- For variables that may be circulating decimals or irrational numbers, mean, standard deviation, first quartile, median, third quartile, LSMean, SE of LSMean, differences of mean values between groups, and 95% confidence interval other than percentage shall be displayed to three decimal places and minimum and maximum values shall be displayed to two decimal places.
- The Kenward-Roger method will be used as the method for adjusting the degree of freedom for repeated measure (MMRM) using mixed effect models. The variance-covariance structure of errors assumes an unstructured (UN) covariance based on the data, but if it does not converge, the assuming conditions shall be more strict in the order of heterogeneous TOEP (TOEPH), heterogeneous AR (1) (ARH [1]), heterogeneous CS (CSH), toeplitz (TOEP), autoregressive (1) (AR [1]), and composite symmetry (CS).
- Sample size, number of adverse events occurred and number of subjects with adverse events will be displayed as integers, and percentage (%) and its 95% confidence interval shall be displayed to one decimal place. The 95% confidence interval for percentage (%) will be calculated as an exact confidence interval using the Clopper-Pearson method.
- Difference of percentage (%) between groups and its 95% confidence interval shall be one decimal place. The 95% confidence interval for difference in percentage (%) will be calculated as an exact confidence interval using the Santerner and Snell method.
- For calculations of confidence interval of difference between groups based on the 2-sample t-test

Copyright © 2022 Translational Research Center for Medical Innovation, Foundation for Biomedical Research and Innovation at Kobe All Rights Reserved.

method, if a test for equality of variance provides $p<0.05$, the 95% confidence interval by the Satterthwaite method will be used assuming that the variance is not equal, and if a test for equality of variance provides $p\geq0.05$, the 95% confidence interval by the pooled estimate will be used assuming that the variance is equal.

- All calculations of the confidence interval shall be 2-sided 95% confidence interval.
- The significance level in tests shall be two-sided 5%. In addition, handling of problems of multiple testing shall not be considered.
- Adverse events, complications, and medical history shall be assigned the codes of system organ class (SOC), preferred term (PT), and lower level term (LLT) using the Medical Dictionary for Regulatory Activities/J dictionary (MedDRA/J). The version of MedDRA/J to be used in the analysis shall be the most recent version at the time of database lock.
- The severity of adverse events shall be determined using the Common Terminology Criteria for Adverse Events v4.0 (CTCAE v4.0) and the JCOG version translated into Japanese.
- Drug codes in the Iyakuhinmei Data File will be assigned to concomitant medications and post-treatment drugs.
- Among the adverse events, those with the relationship with investigational drug of “possible”, “probable” and “definite”, exluding “not related” and “unlikely”, shall be considered as adverse drug reactions.
- Start date for period until onset of an adverse event shall be the start date of treatment.

  Time to adverse event = start date of adverse event - start date of treatment + 1
- In tabulation of adverse events, the results will be displayed in the order of SOC code and PT code. If the same event occurs multiple times in the same subject, the number of events shall be counted multiple times, and the number of subjects shall be counted as one subject. In tabulation by severity, if the same event occurs with different severities, the number of events shall be all counted for each severity, while the number of subjects shall be counted as one subject with the most severe event. Similarly, in tabulation by seriousness, if the same event occurred as both serious and non-serious events, the number of events shall be all counted for both serious and non-serious, while the number of subjects shall be counted as one subject with the serious event.

6.1 Subject composition

- A diagram showing the number of subjects who were secondarily enrolled as slow progressors will be created for secondarily enrolled subjects, randomized subjects, untreated subjects, treated subjects, subjects who discontinued the study prior to Day 29 (Week 4), subjects who continues the study on Day 29 (Week 4), subjects who discontinued the study prior to Day 85 (Week 12), subjects who continues the study on Day 85 (Week 12), subjects who discontinued the study prior to Day 169 (Week 24), subjects who continues the study on Day 169 (Week 24), subjects

Copyright © 2022 Translational Research Center for Medical Innovation, Foundation for Biomedical Research and Innovation at Kobe All Rights Reserved.

who discontinued the study prior to Day 197 (Week 28), subjects who continues the study on Day 197 (Week 28), subjects who discontinued the study prior to Day 225 (Week 32), subjects who continues the study on Day 225 (Week 32), subjects who discontinued the study prior to Day 253 (Week 36), subjects who continues the study on Day 253 (Week 36), subjects who discontinued the study prior to Day 337 (Week 48), and subjects who continues the study on Day 337 (Week 48). Subjects who continues the study on Day X are defines as those who have at least one record of any specified test on Day X.

6.2 Subjects for analysis

- A diagram showing the sample size of the SS1, SS2, FAS and PPS for subjects who were secondarily enrolled as slow progressors will be created by treatment group. In the diagram, the number of subjects who were excluded from the FAS will be determined by reason for exclusion.

6.3 Patient demographics

- For patient demographics in the FAS, PPS, SS1 and SS2, summary statistics will be determined for quantitative variables, and sample size and percentage (%) will be determined for qualitative variables by treatment group and in overall.

Disease duration (months) = 12 × (year at the time of consent obtained – year at the time of diagnosis) + (month at the time of consent obtained – month at the time of diagnosis) + 1

- For the SS1, the number and percentage of subjects with medical history and complications will be calculated by SOC and PT.

6.4 Treatment status

- For the SS1 and FAS, the following parameters will be analyzed for the 2 periods from Day1 to Week 24 and Week 25 and thereafter, as well as for the entire treatment period. Summary statistics will be aggregated for quantitative variables and the number and percentage (%) of subjects will be summarized for qualitative variables by treatment group and in overall. For body weight at start of treatment, in principle, body weight at the initial test (screening test) shall be used; for subjects who roll over from non-progressors to slow progressors, the most recent body weight shall be used.
  - ✓ Prednisolone compliance (based on number of days) (%), total dose (mg), total dose/body weight at start of treatment (mg/kg)
  - ✓ Placebo compliance (based on number of days) (%)
  - ✓ Presence/absence of dose increase

    *For dose increase, subjects who continue up to Week 24 will be included.

Copyright © 2022 Translational Research Center for Medical Innovation, Foundation for Biomedical Research and Innovation at Kobe All Rights Reserved.

6.5 Efficacy endpoints

The following analyses will be performed in the FAS and PPS.
Summaries will be analyzed by treatment group and in overall.

6.5.1 Primary Endpoint

- (Primary analysis) Using the MMRM including the response variables of changes in 10-meter timed walk from baseline to Day 29 (Week 4), Day 85 (Week 12) and Day 169 (Week 24), and the fixed effects of natural-logarithmically transformed values of 10-meter timed walk at baseline, treatment group, timepoints (Day 29 [Week 4]/Day 85 [Week 12]/Day 169 [Week 24]) and interactions between treatment groups and timepoints, LSMean, SE of LSMean and 95% confidence interval for LSMean will be calculated for each timepoint and the difference between groups shall be tested on Day 169 (Week 24).

SAS Coding for MMRM analysis is as follows. The response variable shall be the change (CHG) in 10-meter timed walk from baseline to Day 29 (Week 4)/Day 85 (Week 12)/Day 169 (Week 24) (natural-logarithmically transformed values), and the explanatory variables shall be the natural logarithmically transformed values of 10-meter timed walk at baseline (BASE), treatment group (TRT), timepoint (AVISITN), and interactions between treatment groups and timepoints (TRT*AVISITN).

```
proc mixed data=DATASET method=reml;
    class USUBJID TRT AVISITN;
    model CHG=BASE TRT AVISITN TRT*AVISITN / ddfm=KR solution chisq;
    repeated AVISITN / type=un subject=USUBJID r rcorr;
    lsmeans TRT*AVISITN / pdiff=all cl;
run;
```

- A graph showing time course of LSMean ± SE up to Day 169 (Week 24) will be created with the vertical axis of LSMean at each timepoint obtained by the MMRM described above and a horizontal axis of timepoints.
- (Sensitivity analysis) As a sensitivity analysis of missing values in the primary analysis, analysis of covariance (ANCOVA) will be performed using changes in 10-meter timed walk from baseline to Day 169 (Week 24) (natural-logarithmically transformed values) as responsible variables and natural-logarithmically transformed values of 10-meter timed walk at baseline and randomized group as explanatory variables to calculate LSMean, SE of LSMean and 95% confidence interval for LSMean and perform a test of differences between groups.

Copyright © 2022 Translational Research Center for Medical Innovation, Foundation for Biomedical Research and Innovation at Kobe All Rights Reserved.

SAS Coding for ANCOVA is as follows: The response variable shall be the change (CHG) in 10-meter timed walk from baseline to Day 169 (Week 24) (natural-logarithmically transformed values), and the explanatory variables shall be the natural logarithmically transformed values of 10-meter timed walk at baseline (BASE) and treatment group (TRT) which are the fixed effect.

```
proc glm data=DATASET;
    class TRT;
    model CHG=BASE TRT / solution;
    lsmeans TRT / pdiff=all cl stderr;
    estimate “label” TRT 1 -1;
run;
```

- (Secondary analysis) Using the MMRM used for the primary analysis with additional factors of two randomized group adjustment factors (sex [male/femal] and number of canes [one or less/two or more]), LSMean, SE of LSMean and 95% confidence interval for LSMean will be calculated for each timepoint and the difference between groups shall be tested on Day 169 (Week 24). Site, that is also a randomized group adjustment factor, will not be added because it has not been included for balance between treatment groups.
- (Subgroup analysis) Summary statistics will be calculated by the following categories for changes in 10-meter timed walk from baseline to Day 169 (Week 24) (natural-logarithmically transformed values) to perform for a subgroup analysis. In addition, a Forest plot will be created for the difference between treatment groups (active treatment group – placebo group) and its 95% confidence interval (based on a 2-sample t-test).
  - ✓ Sex (Male/Female)
  - ✓ Concomitant medications (Yes/No)
  - ✓ Disease duration (Less than median value/Not less than median value)
    Median values will be calculated for the FAS and PPS, respectively.
  - ✓ HAL study enrollment prior to enrollment to this study (Yes/No)

6.5.2 Secondary Endpoints

6.5.2.1 10-meter timed walk

- Differences between treatment groups will be tested on Day 29 (Week 4)/Day 85 (Week 12) using MMRM, which was used for the primary analysis of the primary endpoint.
- For changes from baseline on Day 169 (Week 24) and on Day 337 (Week 48) from Day 169 (Week 24) in the placebo group (natural-logarithmically transformed values), summary statistics and 95% confidence interval of the mean will be calculated, summary statistics and 95%

Copyright © 2022 Translational Research Center for Medical Innovation, Foundation for Biomedical Research and Innovation at Kobe All Rights Reserved.

confidence interval of the mean will be calculated for difference between both changes, and a one-sample t-test will be performed.

6.5.2.2 2 minute walk distance

- Using the MMRM including the response variables of changes from baseline to Day 29 (Week 4)/Day 85 (Week 12)/Day 169 (Week 24) and the explanatory variables of values at baseline, randomized group group, timepoints (Day 29 [Week 4]/Day 85 [Week 12]/Day 169 [Week 24]) and interactions between randomized group groups and timepoints, LSMean, SE of LSMean and 95% confidence interval for LSMean will be calculated for each timepoint and the difference between groups shall be tested on Day 29 (Week 4)/Day 85 (Week 12)/Day 169 (Week 24).
- A graph showing time course of LSMean ± SE up to Day 169 (Week 24) will be created with the vertical axis of LSMean at each timepoint obtained by the MMRM described above and a horizontal axis of timepoints.
- For changes from baseline on Day 169 (Week 24) and on Day 337 (Week 48) from Day 169 (Week 24) in the placebo group, summary statistics and 95% confidence interval of mean will be calculated, summary statistics and 95% confidence interval of mean will be calculated for differences between both changes, and a one-sample t-test will be performed.

6.5.2.3 6 minute walk distance

The same analyses as in “6.5.2.2 2 minute walk distance” will be performed.

6.5.2.4 Cerebrospinal fluid concentration of neopterin

- ANCOVA will be performed using changes from baseline to Day 169 (Week 24) (natural-logarithmically transformed values) as responsible variables and baseline value and randomized group group as explanatory variables to calculate LSMean, SE of LSMean and 95% confidence interval for LSMean and perform a test of differences between groups.
- A graph showing time course of LSMean ± SE up to Day 169 (Week 24) will be created with the vertical axis of LSMean at each timepoint obtained by the ANCOVA described above and a horizontal axis of timepoints by treatment groups.
- For changes from baseline on Day 169 (Week 24) and on Day 337 (Week 48) from Day 169 (Week 24) in the placebo group (natural-logarithmically transformed values), summary statistics and 95% confidence interval of mean will be calculated, summary statistics and 95% confidence interval of mean will be calculated for differences between both changes, and a one-sample t-test will be performed.
- On Day169 (Week 24), the number of subjects with “≥30% of decrease (improvement)” compared with the baseline, its percentage (%) and its 95% confidence interval will be calculated. In addition, difference in percentage (%) between treatment groups (pulse group – p.o. group) and its 95% confidence interval will be calculated, and differences between treatment

Copyright © 2022 Translational Research Center for Medical Innovation, Foundation for Biomedical Research and Innovation at Kobe All Rights Reserved.

groups will be tested using Fisher's exact test. A similar analysis will be performed for subjects with "≥50% of decrease (improvement)".

6.5.2.5 Percentage of subjects who discontinued the study drug during the treatment period (Day1 to Day169 [Week 24])

- The number and percentage of subjects who discontinued the study drug during the period from Day1 (start date of treatment) to Day 169 (Week 24) and its 95% confidence interval will be calculated. In addition, difference in percentage (%) between treatment groups (pulse group – p.o. group) and its 95% confidence interval will be calculated, and difference between treatment groups will be tested using Fisher's exact test.

6.5.2.6 CXCL10 concentration

- The same analysis as for "6.5.2.4 Cerebrospinal fluid concentration of neopterin" will be performed.

6.5.3 Other Efficacy Endpoints

6.5.3.1 10-meter timed walk

- Summary statistics will be calculated for measured values at baseline/Day 29 (Week 4)/Day 85 (Week 12)/Day 169 (Week 24)/Day 197 (Week 28)/Day 225 (Week 32)/Day 253 (Week 36)/Day 337 (Week 48) and changes from baseline.
- A graph showing time course of measured values and changes from baseline will be created at each timepoint up to Day 337 (Week 48). A graph will be created using median (first quartile - third quartile) for the original measurement values and mean ± SE for the natural-logarithmically transformed values.

6.5.3.2 2 minute walk distance

- Summary statistics will be calculated for measured values at baseline/Day 29 (Week 4)/Day 85 (Week 12)/Day 169 (Week 24)/Day 197 (Week 28)/Day 225 (Week 32)/Day 253 (Week 36)/Day 337 (Week 48) and changes from baseline.
- A graph showing time course of measured values and changes from baseline (mean ± SE) will be created at each timepoint up to Day 337 (Week 48).

6.5.3.3 6 minute walk distance

- The same analyses as in "6.5.3.2 2 minute walk distance" will be performed.

6.5.3.4 Cerebrospinal fluid concentration of neopterin

- Summary statistics will be calculated for the measured value at baseline/Day 169 (Week 24)/Day 337 (Week 48) and changes from baseline.
- A graph showing time course of measured values and changes from baseline will be created at

Copyright © 2022 Translational Research Center for Medical Innovation, Foundation for Biomedical Research and Innovation at Kobe All Rights Reserved.

each timepoint up to Day 337 (Week 48). A graph will be created using median (first quartile - third quartile) for the original measurement values and mean ± SE for the natural-logarithmically transformed values.

6.5.3.5 CXCL10 concentration

- The same analysis as for "6.5.3.4 Cerebrospinal fluid concentration of neopterin" will be performed.

6.5.3.6 Use of walking aids

- Shift tables will be created for use of walking aids in the clinic on Day 29 (Week 4)/Day 85 (Week 12)/Day 169 (Week 24)/Day 197 (Week 28)/Day 225 (Week 32)/Day 253 (Week 36)/Day 337 (Week 48) compared with baseline.
- Shift tables will be created for use of walking aids at home on Day 29 (Week 4)/Day 85 (Week 12)/Day 169 (Week 24)/Day 197 (Week 28)/Day 225 (Week 32)/Day 253 (Week 36)/Day 337 (Week 48) compared with baseline.

6.5.3.7 IPEC1

- Summary statistics will be calculated for overall score at baseline/Day 29 (Week 4)/Day 85 (Week 12)/Day 169 (Week 24)/Day 197 (Week 28)/Day 225 (Week 32)/Day 253 (Week 36)/Day 337 (Week 48) and changes from baseline.
- Graphs showing time course of total scores and changes from baseline (mean ± SD) by treatment group will be created at each timepoint from baseline to Day 337 (Week 48).

6.5.3.8 QOL

6.5.3.8.1 Modified IPEC 2

- Summary statistics will be calculated for overall score at baseline/Day 29 (Week 4)/Day 85 (Week 12)/Day 169 (Week 24)/Day 197 (Week 28)/Day 225 (Week 32)/Day 253 (Week 36)/Day 337 (Week 48) and changes from baseline.
- Graphs showing time course of total scores and changes from baseline (mean ± SD) by treatment group will be created at each timepoint from baseline to Day 337 (Week 48).

6.5.3.8.2 N-QOL

- Summary statistics will be calculated for overall score at baseline/Day 29 (Week 4)/Day 85 (Week 12)/Day 169 (Week 24)/Day 197 (Week 28)/Day 225 (Week 32)/Day 253 (Week 36)/Day 337 (Week 48) and changes from baseline. The same analysis will be performed for the 2 subscales (sleep/energy and worry/concern) and overall disturbance of daily life.
- Graphs showing time course of total scores and changes from baseline (mean ± SD) by treatment group will be created at each timepoint from baseline to Day 337 (Week 48).
- The 2 subscales (sleep/energy and worry/concern) and overall disturbance of daily life will also

Copyright © 2022 Translational Research Center for Medical Innovation, Foundation for Biomedical Research and Innovation at Kobe All Rights Reserved.

be analyzed in the same manner as the overall score.

6.5.3.8.3 Sexual Health Inventory for Men

- Shift tables will be created for individual scores in the questionnaires on Day 29 (Week 4)/Day 85 (Week 12)/Day 169 (Week 24)/Day 197 (Week 28)/Day 225 (Week 32)/Day 253 (Week 36)/Day 337 (Week 48) compared with baseline.

6.5.3.9 MAS

- Shift tables will be created for MAS on Day 29 (Week 4)/Day 85 (Week 12)/Day 169 (Week 24)/Day 197 (Week 28)/Day 225 (Week 32)/Day 253 (Week 36)/Day 337 (Week 48) compared with baseline.

6.5.3.10 VAS

- Summary statistics will be calculated for the VAS scores in "Global Assessment", "Walking Assessment" and "Lower Limb Pain Assessment" at baseline/Day 29 (Week 4)/Day 85 (Week 12)/Day 169 (Week 24)/Day 197 (Week 28)/Day 225 (Week 32)/Day 253 (Week 36)/Day 337 (Week 48) and change from baseline.
- Graphs showing time course of the VAS scores in "Global Assessment", "Walking Assessment" and "Lower Limb Pain Assessment" and changes from baseline (mean ± SE) by treatment group will be created at each timepoint from baseline to Day 337 (Week 48).

6.5.3.11 Assessment of Urinary dysfunction

6.5.3.11.1 OABSS

- The overall score will be classified into categories of "≤5 (mild)", "6 to 11 (moderate)", or "≥12 (severe)" and shift tables will be created on Day 29 (Week 4)/Day 85 (Week 12)/Day 169 (Week 24)/Day 197 (Week 28)/Day 225 (Week 32)/Day 253 (Week 36)/Day 337 (Week 48) compared with baseline.

6.5.3.11.2 ICIQ-SF

- Shift tables will be created for individual scores in the questionnaires on Day 29 (Week 4)/Day 85 (Week 12)/Day 169 (Week 24)/Day 197 (Week 28)/Day 225 (Week 32)/Day 253 (Week 36)/Day 337 (Week 48) compared with baseline.

6.5.3.11.3 IPSS

- The IPSS will be classified into categories of "≤ 7 (mild)", "8 to 19 (moderate)", or "≥20 (severe)" and shift tables will be created on Day 29 (Week 4)/Day 85 (Week 12)/Day 169 (Week 24)/Day 197 (Week 28)/Day 225 (Week 32)/Day 253 (Week 36)/Day 337 (Week 48) compared with baseline.

Copyright © 2022 Translational Research Center for Medical Innovation, Foundation for Biomedical Research and Innovation at Kobe All Rights Reserved.

6.5.3.12 Timed up-and-go test

- Summary statistics will be calculated for the measured values at baseline/Day 29 (Week 4)/Day 85 (Week 12)/Day 169 (Week 24)/Day 197 (Week 28)/Day 225 (Week 32)/Day 253 (Week 36)/Day 337 (Week 48) and change from baseline,
- A graph showing time course of measured values and changes from baseline (mean ± SD) will be created at each timepoint from baseline to Day 337 (Week 48).

6.5.3.13 OMDS

- Shift tables will be created for the ODMS on Day 29 (Week 4)/Day 85 (Week 12)/Day 169 (Week 24)/Day 197 (Week 28)/Day 225 (Week 32)/Day 253 (Week 36)/Day 337 (Week 48) compared with baseline.

6.6 Safety evaluation

The following analyses will be performed in the SS1 and SS2:
Summaries will be analyzed by treatment group and in overall.

6.6.1 Secondary Endpoints

6.6.1.1 Adverse events

- For all adverse events occurred, adverse drug reactions, adverse events of Grade 2 or higher, serious adverse events, adverse events leading to discontinuation, and fetal adverse events, number of events, number of subjects and incidence rate (%) with 95% confidence interval will be calculated and summarized by treatment group. In addition, Fisher’s exact test will be used to test differences between treatment groups. Summaries will also be performed by timing of occurrence from Day 1 to Day 169 (Week 24) and Week 25 onwards.
- Number of events, number of subjects and incidence rate (%) of all adverse events occurred and adverse drug reactions by SOC and PT will be calculated by treatment group. Summaries will also be performed by timing of occurrence from Day 1 to Day 169 (Week 24) and Week 25 onwards.
- Number of events, number of subjects and incidence rate (%) of all adverse events occurred and adverse drug reactions by SOC and PT will be calculated for each Grade by treatment group. Similarly, analysis will be performed for each seriousness category.

6.6.2 Other Safety Endpoints

6.6.2.1 Clinical laboratory examination

- For quantitative variables, summary statistics at baseline/Day 29 (Week 4)/Day 85 (Week 12)/Day 169 (Week 24)/Day 197 (Week 28)/Day 337 (Week 48) will be calculated by laboratory parameter. Similarly, change from baseline will also be calculated.

Copyright © 2022 Translational Research Center for Medical Innovation, Foundation for Biomedical Research and Innovation at Kobe All Rights Reserved.

- For qualitative variables, shift tables will be created on Day 29 (Week 4)/Day 85 (Week 12)/Day 169 (Week 24)/Day 197 (Week 28)/Day 337 (Week 48) compared with baseline.
- Number of subjects and percentage (%) with decrease, increase, or no increase and decrease for individual clinically significant abnormalities (ICSA) on Day 29 (Week 4)/Day 85 (Week 12)/Day 169 (Week 24)/Day 197 (Week 28)/Day 337 (Week 48) will be calculated by laboratory parameter.
  ICSA shall be determined according to the criteria by the Japanese Society of Chemotherapy [1].
  Table 1 shows the confirmation criteria for clinical laboratory test items in this study. Items not specified in Table 1 are not determined because there are no criteria.

Copyright © 2022 Translational Research Center for Medical Innovation, Foundation for Biomedical Research and Innovation at Kobe All Rights Reserved.

Table 1 ICSA Confirmation Criteria

Items		Unit	Criteria by Japanese Society of Chemotherapy
Complete blood count	WBC	x10^3/µL	<3,000/mm^3<br>. An increase shall not be considered as an AE unless there is any special reason.<br>It can be reported as neutrophil count decreased (<1,500/mm^3) or lymphocyte decreased (<800/mm^3).
	EO	%	≥500/mm3 or ≥10%<br>In addition, this should be considered if there is any associated allergic disease.
	RBC	x10 ^{6}/µL	Males: <3.5 million/mm^3<br>; females: <3.2 million/mm^3
	Hb	g/dL	Less than 10 g/dL
	Hct	%	Males: <35%,<br>females: <30%
	PLT	x10^3 /µL	Decrease: <75,000/mm^3<br>Increase: ≥600,000/mm^3 with any symptom, or<br>In case of 1 million/mm^3 or more
Blood chemistry	BUN	mg/dL	≥1.5 times the upper limit of normal in the site
	Cr	mg/dL	
	T.BIL	mg/dL	
	Na	mEq/L	Decrease: ≤125 mEq/L Increase: ≥155 mEq/L
	K	mEq/L	Decrease: ≤3.2 mEq/L Increase: ≥5.5 mEq/L
	AST	U/L	>2.5 times the upper limit of normal in the site<br>Even if it does not exceed 2.5 times, the following cases should also be considered as adverse events.<br>・ It does not exceed 2.5 times the site reference value, but the contribution of the study drug is considered large based on the range of variation.<br>・ It does not exceed 2.5 times the site reference value, but there is an increasing trend during treatment,<br>and the subject recovers when the effect of the study drug is no longer observed.
	ALT	U/L	
	ALP	U/L	
	Fasting blood glucose	mg/dL	Decrease: <55 mg/dL Increase: >160 mg/dL<br>(A decrease is considered as an abnormal change when it changes to <55 mg/dL with or without food.)
Urine	Urine glucose	N/A	Variation of 2 levels or more (if ± is included in qualitative value, ± is also included in 1 level)
	Urine protein		

6.6.2.2 Vital signs

- Summary statistics at baseline/Day 29 (Week 4)/Day 85 (Week 12)/Day 169 (Week 24)/Day 197 (Week 28)/Day 225 (Week 32)/Day 253 (Week 36)/Day 337 (Week 48) will be calculated by test item. Similarly, changes from baseline will also be calculated.

Copyright © 2022 Translational Research Center for Medical Innovation, Foundation for Biomedical Research and Innovation at Kobe All Rights Reserved.

6.7 Lists

The following lists will be created for the subjects secondary enrolled as slow progressors.

- A list of subjects who discontinued the study will be created including the reason for discontinuation.
- A list of subjects who are excluded from either of the SS1, SS2, FAS or PPS will be created including the reason for exclusion
- A list of demographic information will be created.
- A list of changes in the OMDS from the onset to the date of informed consent will be created.
- A list of prior treatment for HAM will be created.
- A list of history of blood transfusion will be created.
- For the SS1, a list will be created including reported adverse event terms, SOCs, PTs, seriousness, severity, causal relationship and outcome. Similarly, lists of serious adverse events and fatal adverse events will also be created.
- A list of complications and medical history will be created.
- A list of concomitant medications and treatments will be created.
- A list of post-treatment and post-treatment regimens will be created.
- A list of treatment status table including administration status will be created for theSS1.
- A list of antibody analysis will be created.
- A list of laboratory values will be created.
- For the SS1, a list of individual clinically significant abnormal laboratory values based on ICSA determination will be created.
- A list of cerebrospinal fluid test will be created.
- A list of walking aids will be created.
- A list of 10-meter timed walk will be created.
- A list of 2-minute walk distance will be created.
- A list of 6-minute walk distance will be created.
- A list of timed up-and-go test will be created.
- A list of the OMDS will be created.
- A list of the VAS will be created.
- A list of IPEC1 will be created.
- A list of Modified IPEC 2 will be created.
- A list of MAS will be created.
- A list of OABSS will be created.
- A list of ICIQ-SF will be created.
- A list of N-QOL will be created.
- A list of IPSS will be created.
- A list of sexual function assessment will be created.

Copyright © 2022 Translational Research Center for Medical Innovation, Foundation for Biomedical Research and Innovation at Kobe All Rights Reserved.

- A list of vital signs will be created.
- A list of intraocular pressure measurement will be created.
- A list of CXCL10 will be created.

Copyright © 2022 Translational Research Center for Medical Innovation, Foundation for Biomedical Research and Innovation at Kobe All Rights Reserved.

6.8 Sample size calculation and power justification

Twenty subjects in the Prednisolone group and 20 subjects in the placebo group

Data analysis of Daily clinical practice in patients with HAM was performed to estimate the change and standard deviation in the logarithmically transformed 10-meter timed walk (log 10mWT) with prednisone treatment. The data assessed are Data Set A consisting of 14 Japanese patients with HAM who received prednisolone treatment and Data Set B consisting of 71 patients with HAM who were observed for 6 months. Data Set B is composed of 41 patients in Japan, 27 patients in the UK, and 3 patients in Brazil, and includes the period during which the patients in Japan are receiving steroid treatment. Steroid treatment was not administered to the patients in the UK and Brazil.
First, the effect of prednisolone treatment was estimated. In Data Set A, log 10mWT decreased by 0.165 in 6 months compared to pre-treatment (improved by 15%). On the other hand, in the patient group (n=30) in which steroid treatment was not performed in Data Set B, the disease worsened by 0.0575 over 6 months (worsened by 6%). Next, the standard deviation for log 10mWT was estimated. A log 10mWT regression model at 6 months adjusted for the values at the beginning of observation was created using Japanese data from Data Set B to estimate the standard deviation for the error section and the estimated value was 0.21. Therefore, the estimated difference between groups due to this study intervention is around 0.223, and the estimated standard deviation of log 10mWT when performing analysis of covariance is around 0.21.
Under the above estimate, when the significance level is set at 0.05 (two-sided), and the group comparison is performed with an analysis of covariance adjusted for the baseline values, the statistical power of 0.8 or more can be obtained with the number of patients of greater than or equal to 30. Tables 2, 3, and 4 show the number of patients required for the statistical power of 0.8, 0.85, and 0.9, respectively. It is expected that approximately 40 subjects will be enrolled at sites participating in this study during the enrollment period. Given the differences between the groups and standard deviations as estimated above, the statistical power of 0.9 can be obtained with 40 patients. In addition, even if the standard deviation is estimated to be about 15% larger, 0.24, the statistical power of 0.8 can be maintained, indicating a somewhat conservative setting. Therefore, the target number of patients for this study is 40.

Copyright © 2022 Translational Research Center for Medical Innovation, Foundation for Biomedical Research and Innovation at Kobe All Rights Reserved.

Table 2. Required sample size (statistical power of 80%)

Treatment effect	SD										
	0.17	0.18	0.19	0.20	0.21	0.22	0.23	0.24	0.25	0.26	0.27
−0.248（22%減少）	16	18	20	22	24	26	28	32	34	36	40
−0.236（21%減少）	18	20	22	24	26	30	32	34	38	40	44
−0.223（20%減少）	20	22	24	28	30	32	36	38	42	44	48
−0.211（19%減少）	22	24	28	30	34	36	40	42	46	50	54
−0.198（18%減少）	26	28	30	34	38	40	44	48	52	56	60
−0.186（17%減少）	28	32	34	38	42	46	50	54	58	64	68
−0.174（16%減少）	32	36	40	44	48	52	56	62	66	72	78
−0.163（15%減少）	36	40	44	50	54	60	64	70	76	82	88
−0.151（14%減少）	42	46	52	58	62	68	74	82	88	96	102
−0.139（13%減少）	48	54	60	66	74	80	88	96	104	112	120

Table 3. Required sample size (statistical power of 85%)

Treatment effect	SD										
	0.17	0.18	0.19	0.20	0.21	0.22	0.23	0.24	0.25	0.26	0.27
−0.248（22%減少）	18	20	24	26	28	30	32	36	38	42	44
−0.236（21%減少）	20	24	26	28	30	34	36	40	42	46	50
−0.223（20%減少）	22	26	28	30	34	36	40	44	48	50	54
−0.211（19%減少）	26	28	32	34	38	42	44	48	52	56	60
−0.198（18%減少）	28	32	34	38	42	46	50	54	58	64	68
−0.186（17%減少）	32	36	40	44	48	52	56	62	66	72	78
−0.174（16%減少）	36	40	44	50	54	60	64	70	76	82	88
−0.163（15%減少）	42	46	52	56	62	68	74	80	86	94	102
−0.151（14%減少）	48	54	58	66	72	78	86	92	100	108	118
−0.139（13%減少）	56	62	68	76	84	92	100	108	118	128	136

Table 4. Required sample size (statistical power of 90%)

Treatment effect	SD										
	0.17	0.18	0.19	0.20	0.21	0.22	0.23	0.24	0.25	0.26	0.27
−0.248（22%減少）	22	24	26	30	32	34	38	42	44	48	52
−0.236（21%減少）	24	26	30	32	36	38	42	46	50	54	58
−0.223（20%減少）	26	30	32	36	40	42	46	50	54	60	64
−0.211（19%減少）	30	32	36	40	44	48	52	56	62	66	70
−0.198（18%減少）	32	36	40	44	50	54	58	64	68	74	80
−0.186（17%減少）	38	42	46	50	56	60	66	72	78	84	90
−0.174（16%減少）	42	46	52	58	62	68	76	82	88	96	102
−0.163（15%減少）	48	54	60	66	72	78	86	94	102	110	118
−0.151（14%減少）	56	62	68	76	84	92	100	108	118	126	136
−0.139（13%減少）	64	72	80	88	98	106	116	126	138	148	160

Copyright © 2022 Translational Research Center for Medical Innovation, Foundation for Biomedical Research and Innovation at Kobe All Rights Reserved.

7 Appendix

None

8 Reference Material

1) Protocol Version 1.12 (October 08, 2020)

9 References

1. “Antimicrobial Drug Safety Evaluation Criteria” Final Report (final version) by the Japanese Society of Chemotherapy, Committee for Criteria of Safety Evaluation of Antimicrobial Drugs. Journal of the Japanese Society of Chemotherapy Vol. 58, 4/2010 (July): pp. 484 to 493

Copyright © 2022 Translational Research Center for Medical Innovation, Foundation for Biomedical Research and Innovation at Kobe All Rights Reserved.

10 Revision History

Version	Date prepared	Main content	Author
1.0	November 18, 2020	Analysis plan for the final analysis	Kenichiro Tanabe

Copyright © 2022 Translational Research Center for Medical Innovation, Foundation for Biomedical Research and Innovation at Kobe All Rights Reserved.

Any transfer, copying, publication, or other unauthorized reproduction of all or part of this material is prohibited without the prior consent of the Translational Research Center for Medical Innovation (TRI).

Translational Research Center for Medical Innovation.
Address: 1-5-4, Minatojimaminamimachi, Chuo-ku, Kobe, 650-0047, Japan.
TEL: +81-78-303-9107　FAX: +81-303-9094
URL: http://www.tri-kobe.org

Copyright © 2022 Translational Research Center for Medical Innovation, Foundation for Biomedical Research and Innovation at Kobe All Rights Reserved.
SOP-STAT-002-RD-01 Statistical analysis plan Template 2019.08.23
