# Supplementary material for: Efficacy of Corticosteroid Therapy for HTLV-1-Associated Myelopathy: A Randomized Controlled Trial (HAMLET-P)
Source: Viruses. 2022 Jan 12;14(1):136. doi: 10.3390/v14010136 (PMC8780460; doi:10.3390/v14010136)
Supplement: Supplementary file 1 [file viruses-14-00136-s001.zip › SuppelementaryMaterial.pdf]

Supplementary Materials

1. **SUPPLEMENTARY METHODS**

   1) Laboratory analysis

   2) Statistical analysis

2. **SUPPLEMENTARY TABLES**

Table S1. Changes in motor function and CSF marker concentrations in the placebo group during the placebo and prednisolone periods

Table S2. Summary of exploratory assessment in slow progressors

Table S3. Changes in OMDS and the Modified Ashworth Scale score at 24 and 48 weeks from baseline in slow progressors

3. **SUPPLEMENTARY FIGURES**

Figure S1. Trial overview

Figure S2. Treatment flow chart of rapid progressors

Figure S3. Treatment flow chart of slow progressors

Figure S4. Changes in motor function and CSF marker concentrations in rapid progressors

Figure S5. Exploratory assessment in rapid progressors

Figure S6. Changes in motor function and CSF marker concentrations in slow progressors

Figure S7. Exploratory assessment in slow progressors

1. SUPPLEMENTARY METHODS

1) Laboratory analysis

CXCL10 concentration in the cerebrospinal fluid (CSF) was assessed using the cytometric bead array method (BD Biosciences, San Diego, CA) in the laboratory according to the manufacturer's instructions. CSF neopterin concentration was evaluated using high-performance liquid chromatography (SRL, Inc. Tokyo, Japan). For HTLV-1 proviral load measurements in peripheral blood mononuclear cells (PBMCs), PBMCs were separated from the patients' peripheral blood using Ficoll-based density gradient centrifugation. DNA was extracted from the PBMCs. DNA was also extracted from CSF cells to investigate the HTLV-1 proviral load in the CSF. The HTLV-1 proviral load was measured by real-time PCR, as reported in a previous study.[1] The HTLV-1 proviral load in PBMCs and CSF cells was expressed as the HTLV-1 proviral copy number per 100 cells (copies/100 cells). That in the CSF (mL) was calculated using the equation below.

HTLV-1 proviral copy number per milliliter of CSF (copies/mL) = HTLV-1 PVL (copies/100 CSF cells) × CSF cell count (/μl) × 10

1. Yamano Y, Nagai M, Brennan M, et al. Correlation of human T-cell lymphotropic virus type 1 (HTLV-1) mRNA with proviral DNA load, virus-specific CD8(+) T cells, and disease severity in HTLV-1-associated myelopathy (HAM/TSP). Blood 2002;99:88-94.

2) Statistical analysis

For between-group comparisons of secondary outcomes in rapid progressors, the Fisher's exact test or the 2-sample Wilcoxon test (exact method) was used. For between-group comparisons of disease evaluations other than the primary and secondary outcomes in slow progressors, the 2-sample Wilcoxon test was applied. The 1-sample *t*-test was utilized to compare 10-meter walking time (10mWT), 2-minute walk distance (2MWD), 6-minute walk distance (6MWD), and CSF neopterin and CXCL10 concentrations in the placebo group between baseline and week-24 (placebo period) and between week-24 and week-48 (prednisolone period). Changes in these parameters in the placebo and prednisolone periods were also compared using the 1-sample t-test. Log-transformation

was applied to 10mWT and CSF marker concentrations to produce a normal distribution for analysis. Statistical analysis was performed using SAS version 9.4 (SAS Institute Inc., Cary, NC). *P* values were 2-sided, and a significance level of 0.05 was applied for all tests.

2. SUPPLEMENTARY TABLES

Table S1. Changes in motor function and CSF marker concentrations in the placebo group during the placebo and prednisolone periods

Measurement		Baseline to Week 24 (Placebo period, n = 14)	Week 24 to Week 48 (Prednisolone period, n = 13)	*p* value[a]
10mWT	% change	−4.0 (−10.1–2.3)	−9.9 (−15.7–−3.7)	0.095
	p value[b]	0.19	0.005	
2MWD	% change	1.2 (−2.5–4.8)	8.5 (0.3–16.7)	0.13
	p value[b]	0.50	0.043	
6MWD	% change	2.6 (−1.8–7.1)	8.4 (0.8−15.9)	0.14
	p value[b]	0.23	0.033	
CSF neopterin	% change	−3.6 (−20.0–16.1)	−32.2 (−43.5–−18.5)	0.059
	p value[b]	0.68	< 0.001	
CSF CXCL10	% change	−0.9 (−21.9–25.7)	−54.1 (−66.7–−36.7)	0.006
	p value[b]	0.94	< 0.001	

Data about 10mWT and CSF neopterin and CXCL10 concentrations are expressed as median percent change (95% confidence interval), which were calculated using the exponential of the mean difference in the log-transformed measured values. Data about 2MWD and 6MWD are expressed as mean percent change (95% confidence interval).

[a] *p* value for comparison between the changes in the placebo and prednisolone periods analyzed using the 1-sample *t*-test

[b] *p* value for comparison between baseline and week-24, and between week-24 and week-48 analyzed using the 1-sample t-test

CSF, cerebrospinal fluid; 2MWD, 2-minute walk distance; 6MWD, 6-minute walk distance; 10mWT, 10-meter walking time.

Table S2. Summary of exploratory assessment in slow progressors

Measurement	Prednisolone	Placebo	p-value
Timed up-and-go test (seconds)	−0.7 (−2.3–−0.1)	−0.3 (−0.9–0.4)	0.14
IPEC1 score	−2.0 (−5.0–−1.0)	0.0 (0.0–0.0)	0.002
VAS score for global condition of HAM (mm)	8.0 (−8.0–17.0)	5.5 (−4.0–12.0)	0.43
VAS score for walking (mm)	0.0 (−5.0–16.0)	10.5 (−7.0–24.0)	0.54
VAS score for pain (mm)	3.0 (0.0–21.0)	−4.0 (−15.0–0.0)	0.025
IPSS score	−3.0 (−7.0–0.0)	−1.0 (−3.0–0.0)	0.42
OABSS score	0.0 (−1.0–2.0)	−1.0 (−3.0–0.0)	0.079
N-QOL score	6.8 (0.0–10.4)	4.2 (0.0–8.3)	0.35
ICIQ-SF score	1.0 (−1.0–3.0)	0.0 (−1.0–1.0)	0.35
HTLV-1 proviral load in PBMCs (copies/100 PBMCs)	−1.4 (−1.9–0.1)	−0.2 (−1.0–0.6)	0.10
HTLV-1 proviral load in CSF cells (copies/100 CSF cells)	−1.9 (−3.8–0.6)	0.3 (−2.6–1.8)	0.047
HTLV-1 proviral load in CSF (copies/mL CSF)	−129.0 (−271.5–8.2)	−39.9 (−175.8–1.7)	0.34

Values are presented as median (interquartile range) changes from baseline at week-24. P values show the significance of between-group differences analyzed using the 2-sample Wilcoxon test.

CSF, cerebrospinal fluid; ICIQ-SF, International Consultation on Incontinence Questionnaire-Short Form; IPEC 1, Insituto de Pesquisa Clinica Evandro Chagas disability score 1; IPSS, International Prostate Symptom Score; MAS, Modified Ashworth Scale; N-QOL, Nocturia–Quality of Life Questionnaire; OABSS, Overactive Bladder Symptom Score; OMDS, Osame Motor Disability Score; PBMCs, peripheral blood mononuclear cells; VAS, visual analog scale.

Table S3. Changes in OMDS and the Modified Ashworth Scale score at 24 and 48 weeks from baseline in slow progressors

Measurement		Prednisolone group (n = 15) [a]	Placebo group (n = 15) [b]	p-value
OMDS	Week 24			0.16
	Improved	2 (13.3%)	0 (0.0%)	
	No change	13 (86.7%)	14 (100.0%)	
	Worsened	0 (0.0%)	0 (0.0%)	
	Week 48			0.44
	Improved	5 (38.5%)	7 (53.8%)	
	No change	8 (61.5%)	6 (46.2%)	
	Worsened	0 (0.0%)	0 (0.0%)	
MAS	Week 24			0.71
	Improved	5 (33.3%)	3 (21.4%)	
	No change	9 (60.0%)	11 (78.6%)	
	Worsened	1 (6.7%)	0 (0.0%)	
	Week 48			0.24
	Improved	6 (46.2%)	9 (69.2%)	
	No change	7 (53.8%)	4 (30.8%)	
	Worsened	0 (0.0%)	0 (0.0%)	

The 2-sample Wilcoxon test was used to evaluate between-group differences.

[a] Two patients in the prednisolone group discontinued the trial between week-24 and week-48.

[b] Two patients in the placebo group discontinued the trial (one patient each before and after week-24).

Values are expressed as number (percentage).

MAS, Modified Ashworth Scale; OMDS, Osame Motor Disability Score.

3. SUPPLEMENTARY FIGURES

Figure S1. Trial overview

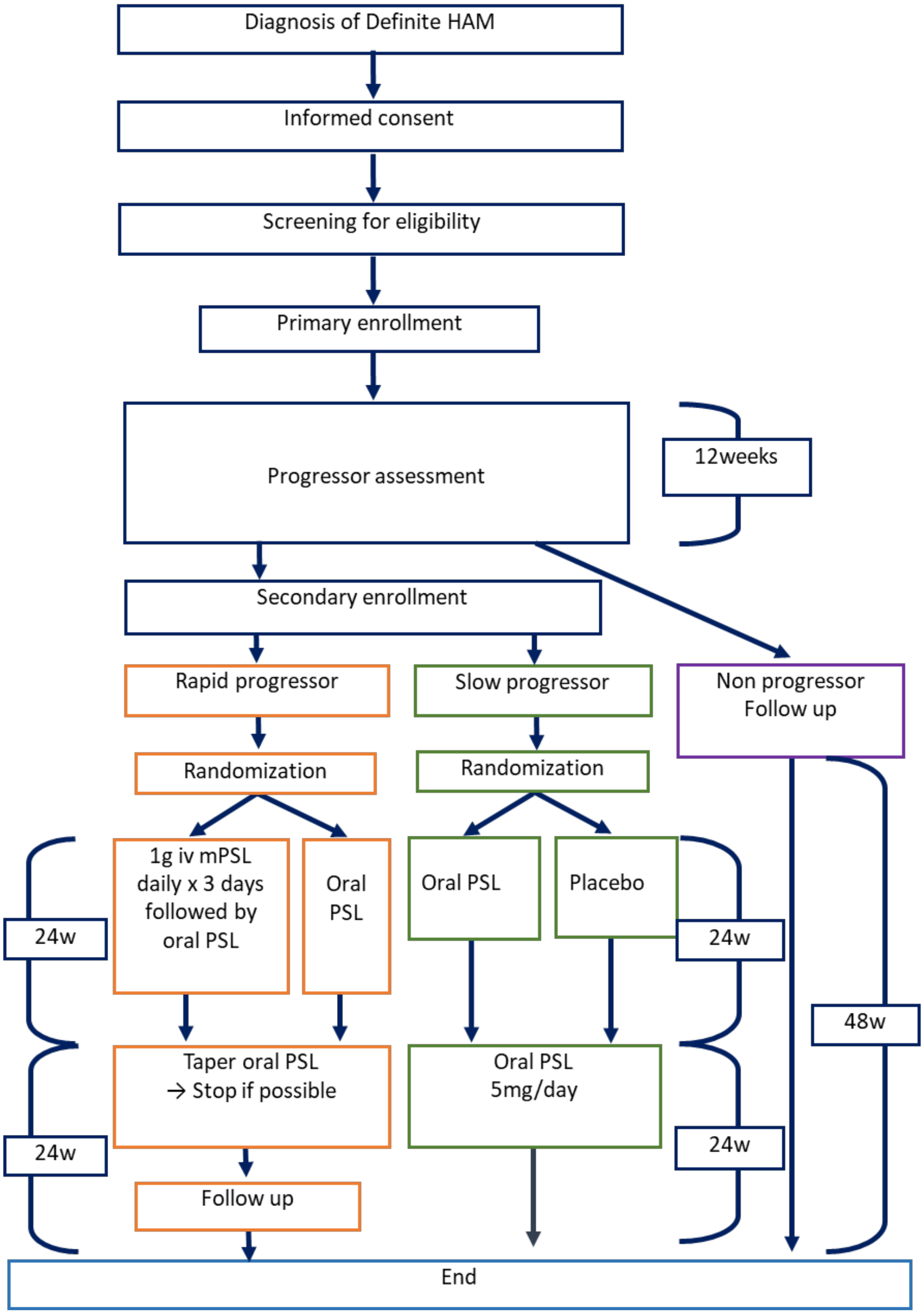

HAM, HTLV-1-associated myelopathy; iv mPSL, intravenous methylprednisolone; PSL, prednisolone

Figure S2. Treatment flow chart of rapid progressors

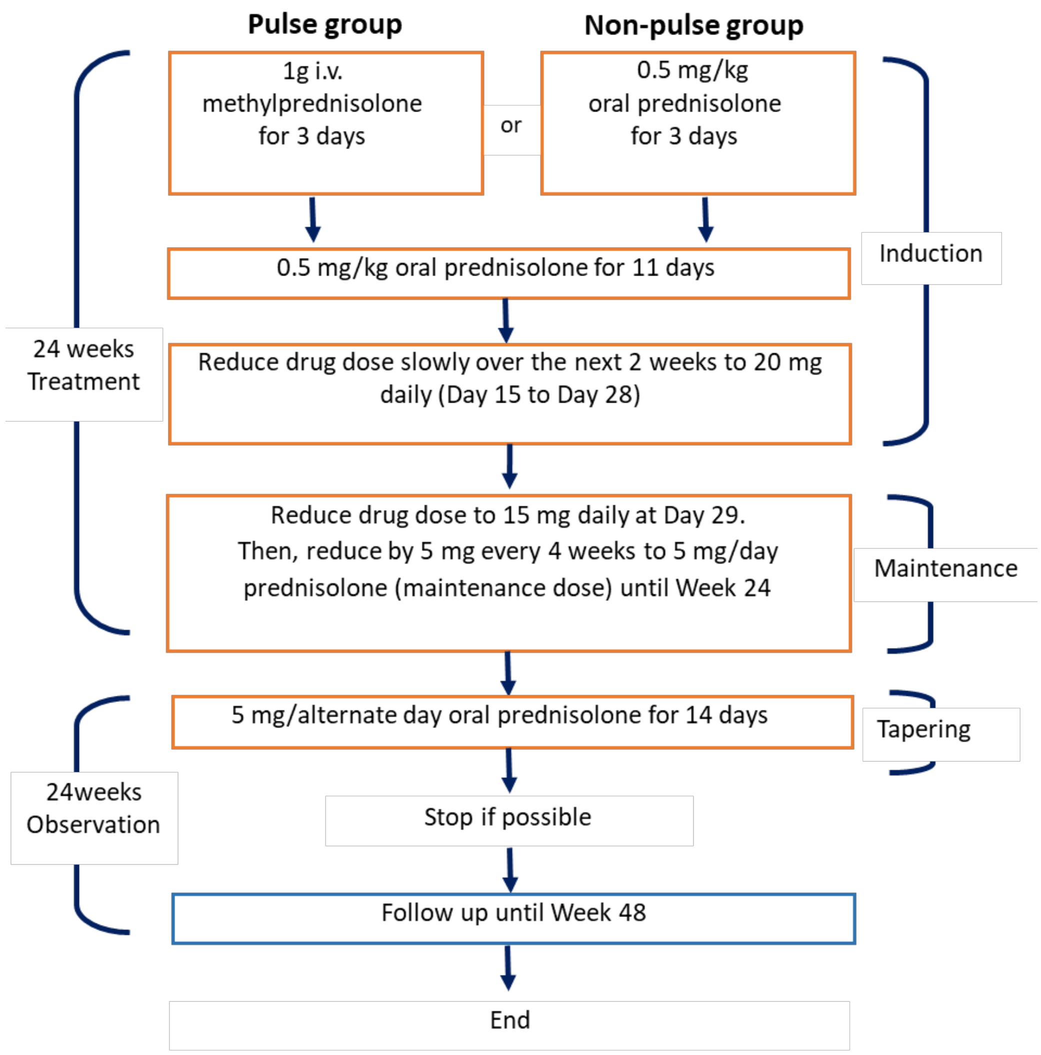

Additional treatment for deterioration after week 4

Additional treatment is not allowed until week 4. If a participant experienced progression of HAM symptoms and met the criteria for dose increase or resumption after week 4, additional treatment should be provided.

1) After evaluation at week 4 until evaluation at week 24

[Dose increase criteria 1] "≥30% worsening in the 10-meter walking test (10mWT)" or "≥1 grade worsening in the Osame Motor Disability Score (OMDS)" compared with baseline. **[Dose increase procedures 1]** Methylprednisolone 1 g/day was intravenously administered for 3 consecutive days. Subsequently, oral prednisolone was administered at a dose of 0.5 mg/kg/day (for 11 days) and gradually reduced to 20 mg/day over 2 weeks. The method of tapering should be determined based on the discretion of physician. Subsequently, the

dose was reduced from 2.5 mg/day (5 mg every other day) /4 weeks to 5 mg/day/4 weeks to find a maintenance dose that does not meet the dose increase criteria. Oral treatment was continued at the maintenance dose.

[Dose increase criteria 2] "≥10% worsening in 10mWT compared with the value at week 4 was observed at two visits (which can be apart from each other)." **[Dose increase procedures 2]** The prednisolone dose was increased by 2.5 mg/day (5 mg every other day)/4 weeks to maintain the dose that does not meet the increase criteria.

2) After evaluation at week 24

[Dose increase/resumption criteria 1] "≥30% worsening in 10mWT" or "≥1 grade of worsening in OMDS" compared with that at week 24 was observed. **[Dose increase/resumption procedures 1]** Methylprednisolone 1 g/day was administered intravenously for 3 consecutive days. Prednisolone was administered at a dose used immediately before worsening. If the dose increase criteria are subsequently met, the dose is increased by 2.5 mg/day (5 mg every other day)/4 weeks to identify a maintenance dose that does not meet the dose increase criteria. Oral treatment was continued at the maintenance dose. If the dose is reduced, the minimum maintenance dose should be 5 mg.

[Dose increase/resumption criteria 2] "≥10% worsening in 10mWT compared with that at week 24 was observed. **[Dose increase/resumption procedures 2]** Prednisolone was administered at a dose used immediately before worsening. If the dose increase criteria are subsequently met, the dose is increased by 2.5 mg/day (5 mg every other day)/4 weeks to identify the maintenance dose that does not meet the dose increase criteria. Oral treatment was continued at that maintenance dose. If the dose is reduced, the minimum maintenance dose should be 5 mg.

Figure S3. Treatment flow chart of slow progressors

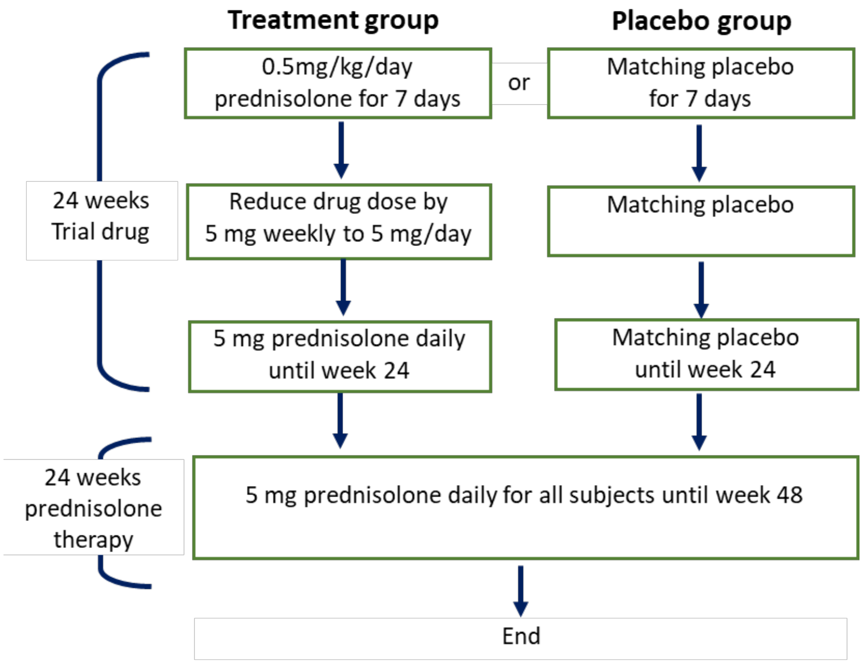

Additional treatment for deterioration after week 24

Additional treatment was not allowed until week 24. If a participant experienced worsening of HAM symptoms and met the criteria for dose increase or additional dose increase after the week 24 visit, additional treatment should be given (maximum dose of 10 mg/day).

1) Criteria based on the Osame Motor Disability Score (OMDS)

[Dose increase criteria] "≥1 grade worsening in the OMDS" compared with that at week 24. **[Dose increase procedures]** The prednisolone dose was increased by 2.5 mg/day (5 mg every other day)/4 weeks.

2) Criteria based on the 10-meter walking test (10mWT)

[Dose increase criteria] ≥10% worsening in the 10mWT compared with that at week 24 was observed during the two study visits (which can be apart) after week 24. **[Dose increase procedures]** The prednisolone dose should be increased to 7.5 mg/day (5 or 10 mg every other day), and oral treatment was continued at that dose.

[Additional increase criteria] If the 10mWT performed at the visit after a dose increase to 7.5 mg/day (5 mg/10 mg every other day) shows worsening by 10% or more worsening compared with that observed at the time of the increase. **[Additional increase procedures]** The prednisolone dose should be increased to 10.0 mg/day, and oral treatment was continued at that dose.

Figure S4. Changes in motor function and CSF marker concentrations in rapid progressors

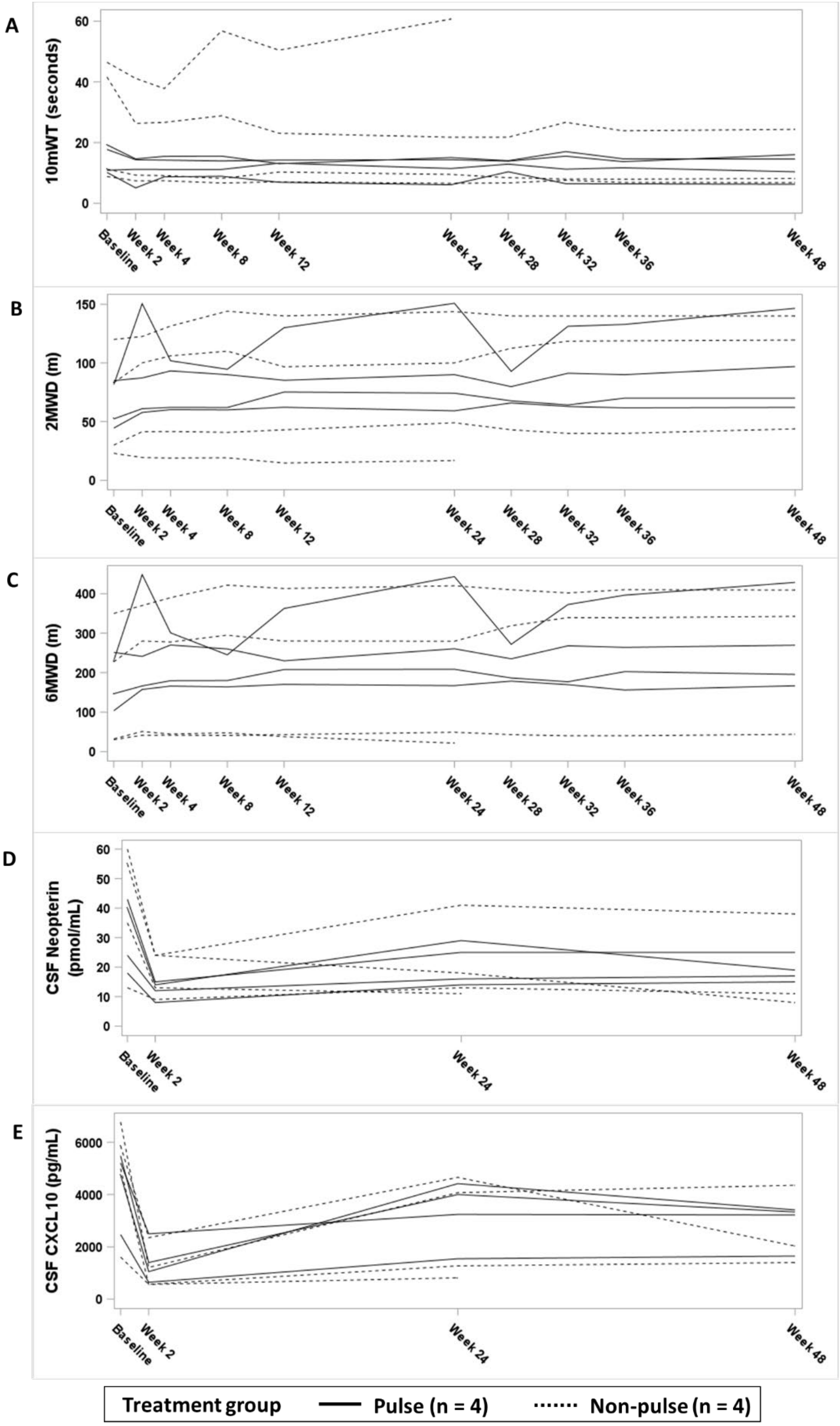

Figure S5. Exploratory assessment in rapid progressors

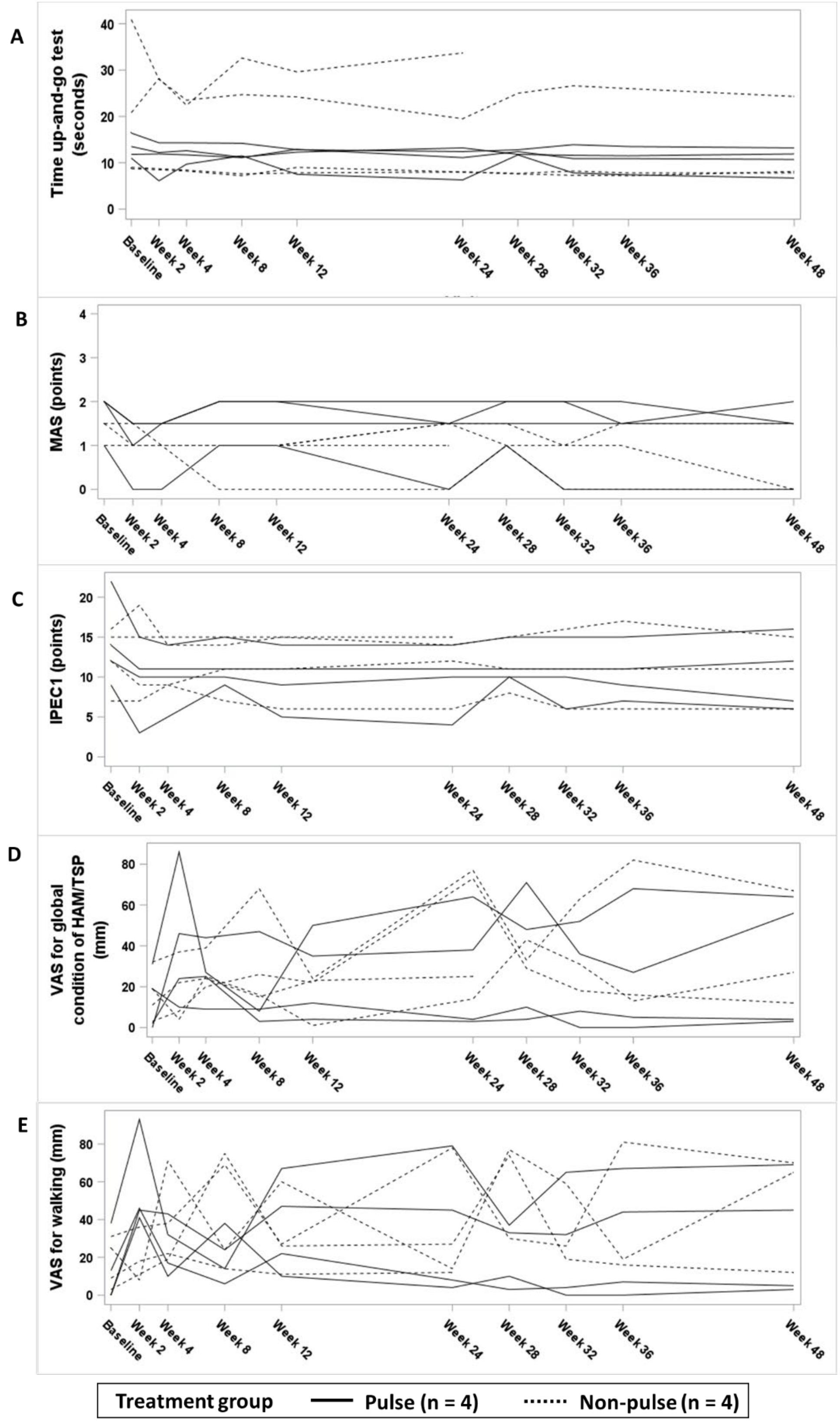

Figure S5. Exploratory assessment in rapid progressors (continued)

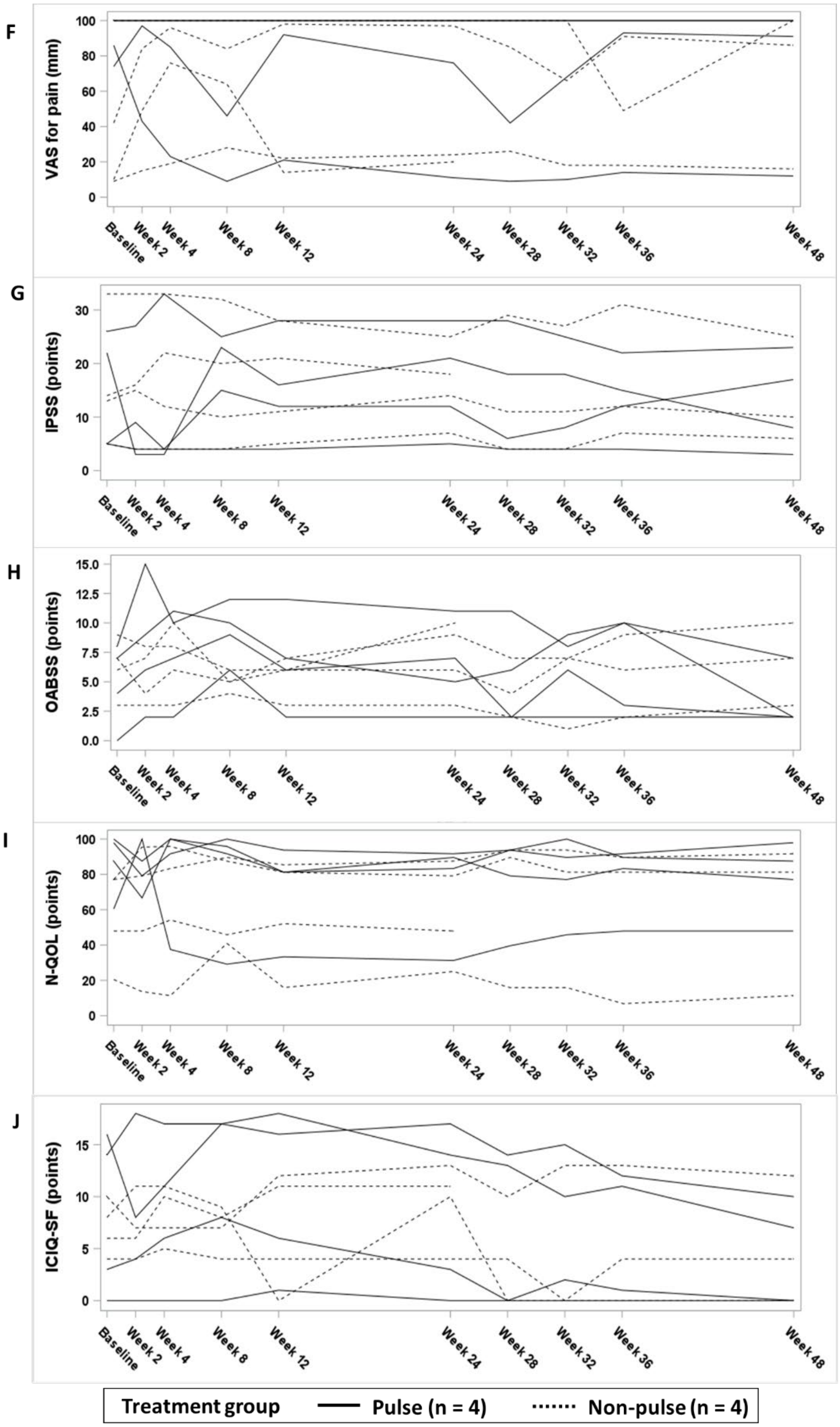

Figure S5. Exploratory assessment in rapid progressors (continued)

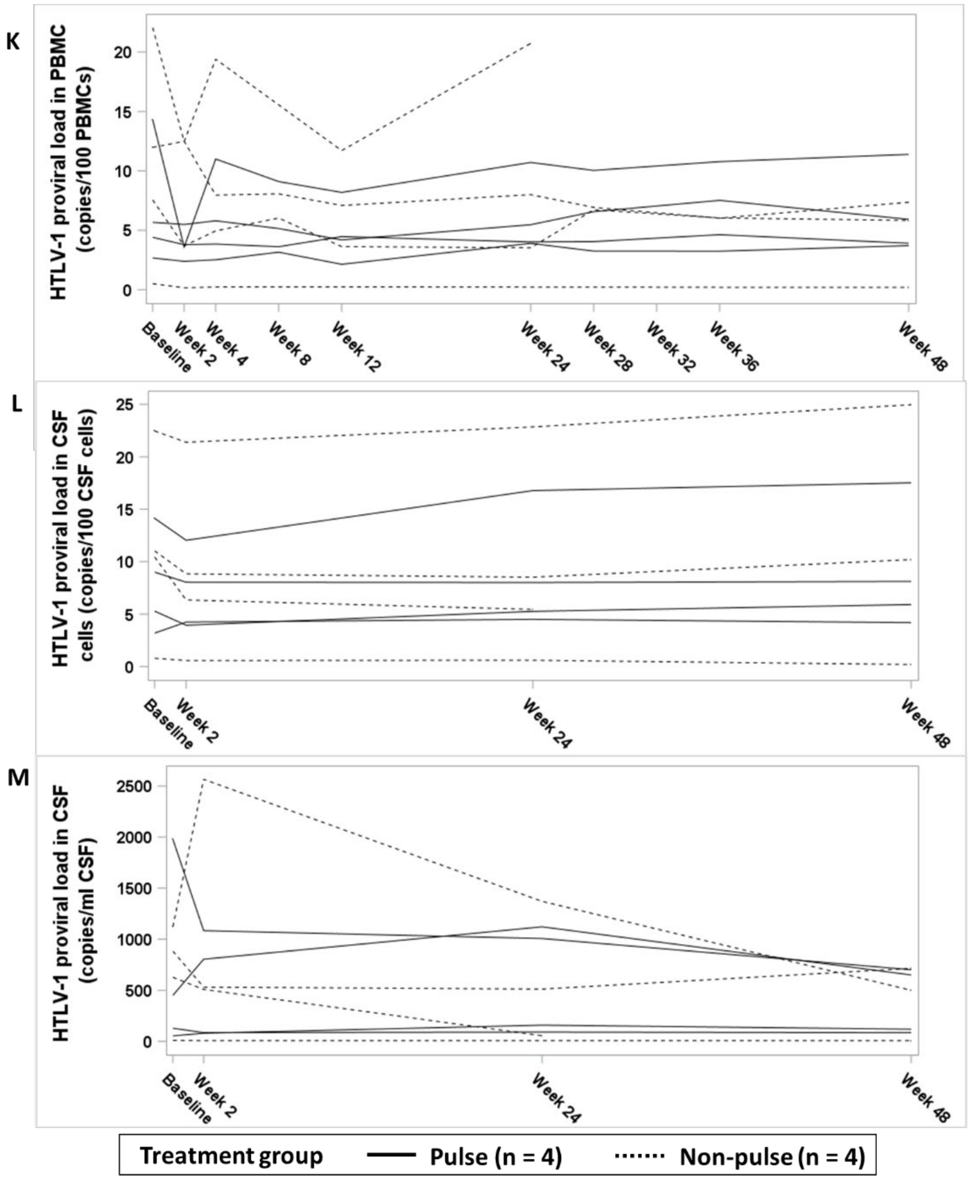

The value 1+ in the Modified Ashworth Scale (MAS) was treated as 1.5 points.

CSF, cerebrospinal fluid; ICIQ-SF, International Consultation on Incontinence Questionnaire-Short Form; IPEC 1, Insituto de Pesquisa Clinica Evandro Chagas disability score 1; IPSS, International Prostate Symptom Score; MAS, Modified Ashworth Scale; N-QOL, Nocturia–Quality of Life Questionnaire; OABSS, Overactive Bladder Symptom Score; PBMCs, peripheral blood mononuclear cells; VAS, visual analog scale.

Figure S6. Changes in motor function and CSF marker concentrations in slow progressors

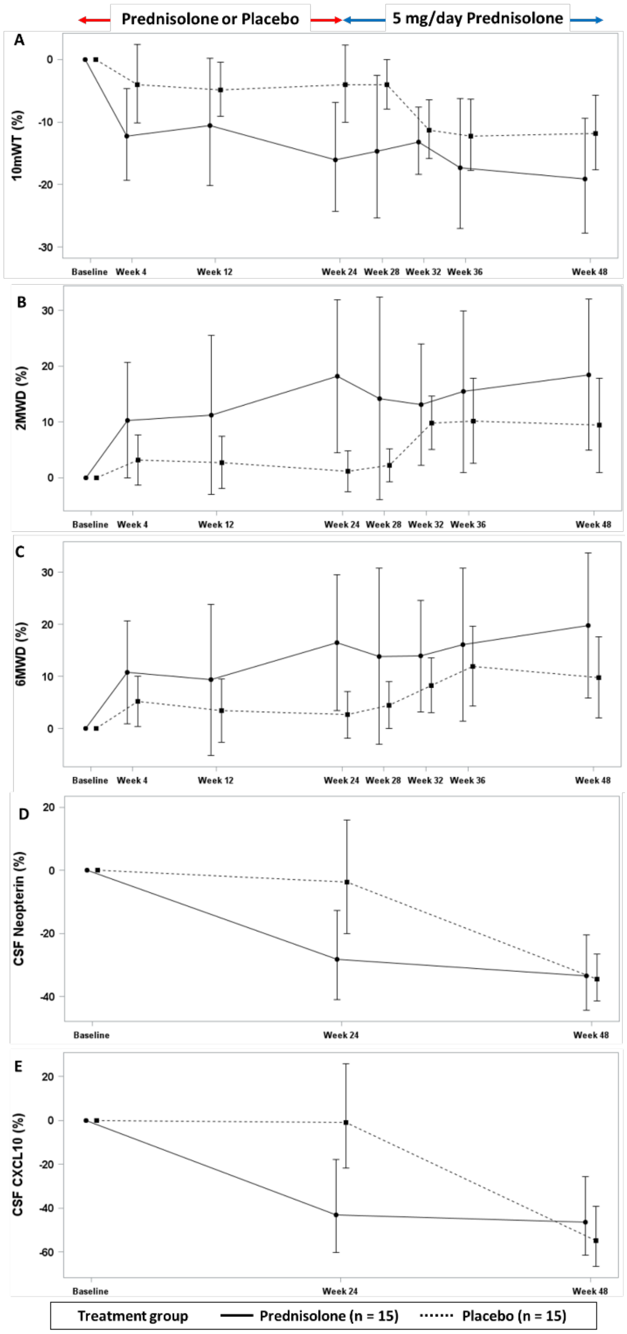

(A–E) Percent changes from baseline are shown. Data about 10mWT and CSF neopterin and CXCL10 concentrations are expressed as median and 95% confidence interval, which were calculated using the exponential of the mean difference in the log-transformed measured values. Data about 2MWD and 6MWD are expressed as mean and 95% confidence interval.

CSF, cerebrospinal fluid; 2MWD, 2-minute walk distance; 6MWD, 6-minute walk distance; 10mWT, 10-meter walking time.

Figure S7. Exploratory assessment in slow progressors

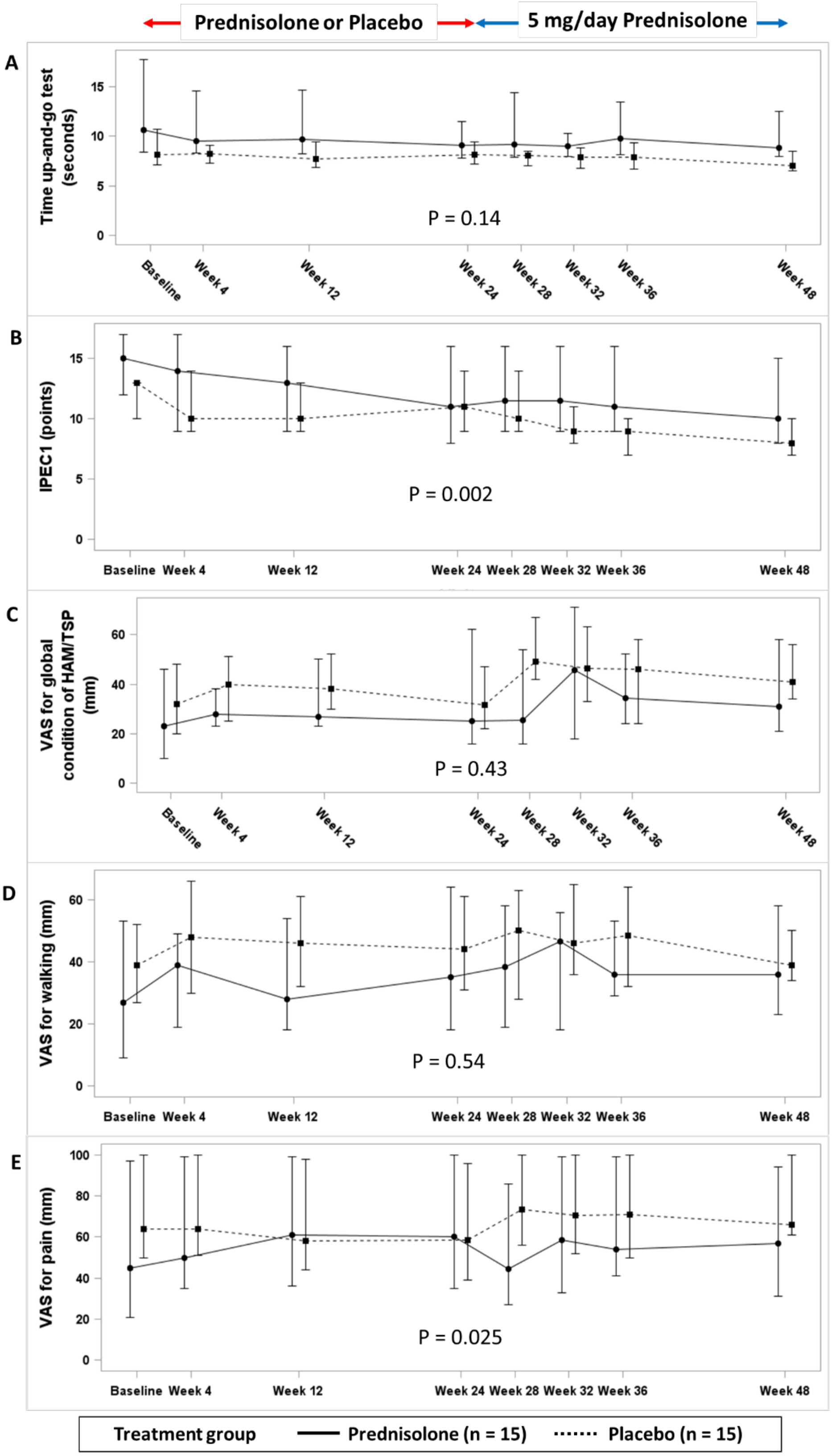

Figure S7. Exploratory assessment in slow progressors (continued)

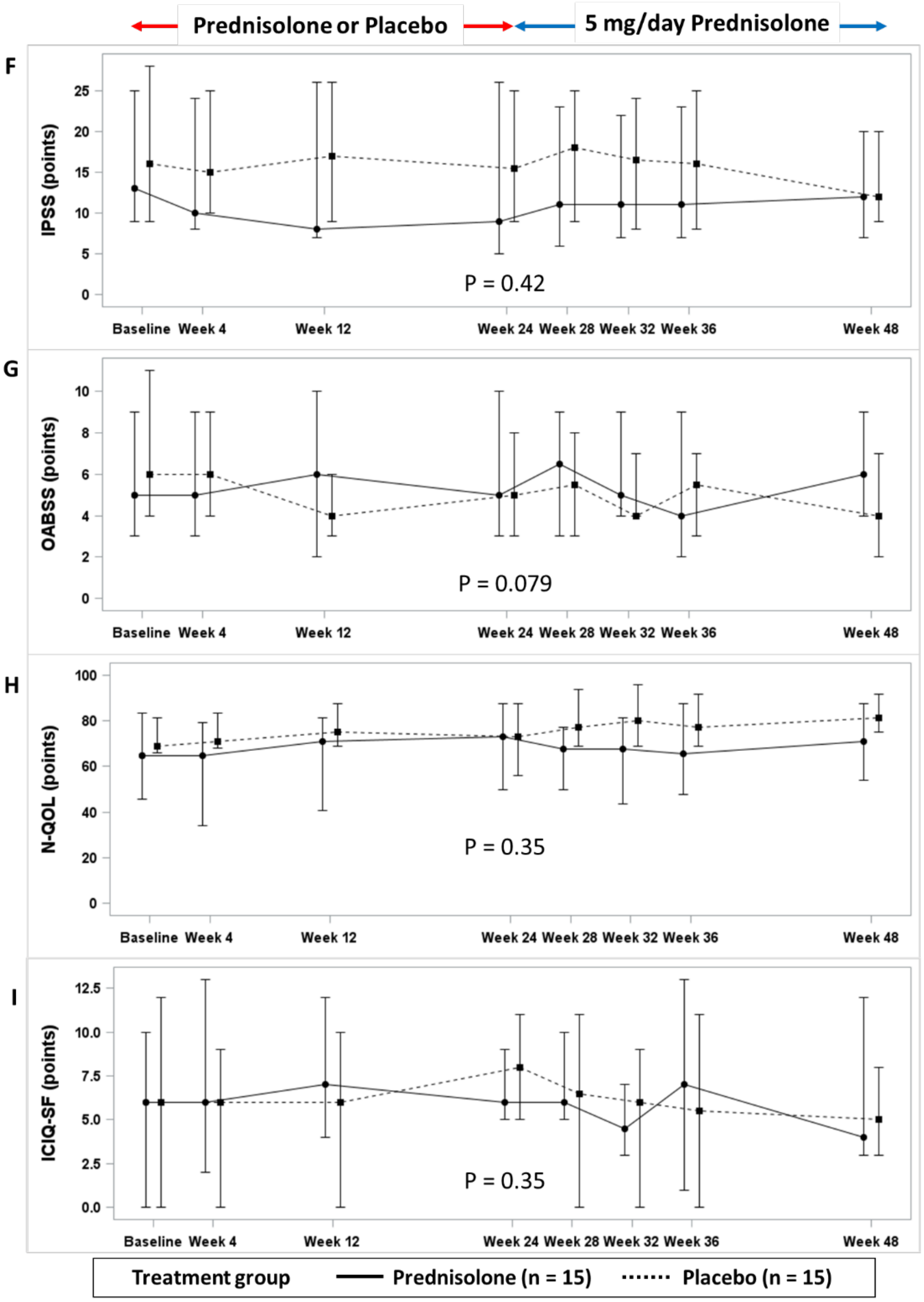

Figure S7. Exploratory assessment in slow progressors (continued)

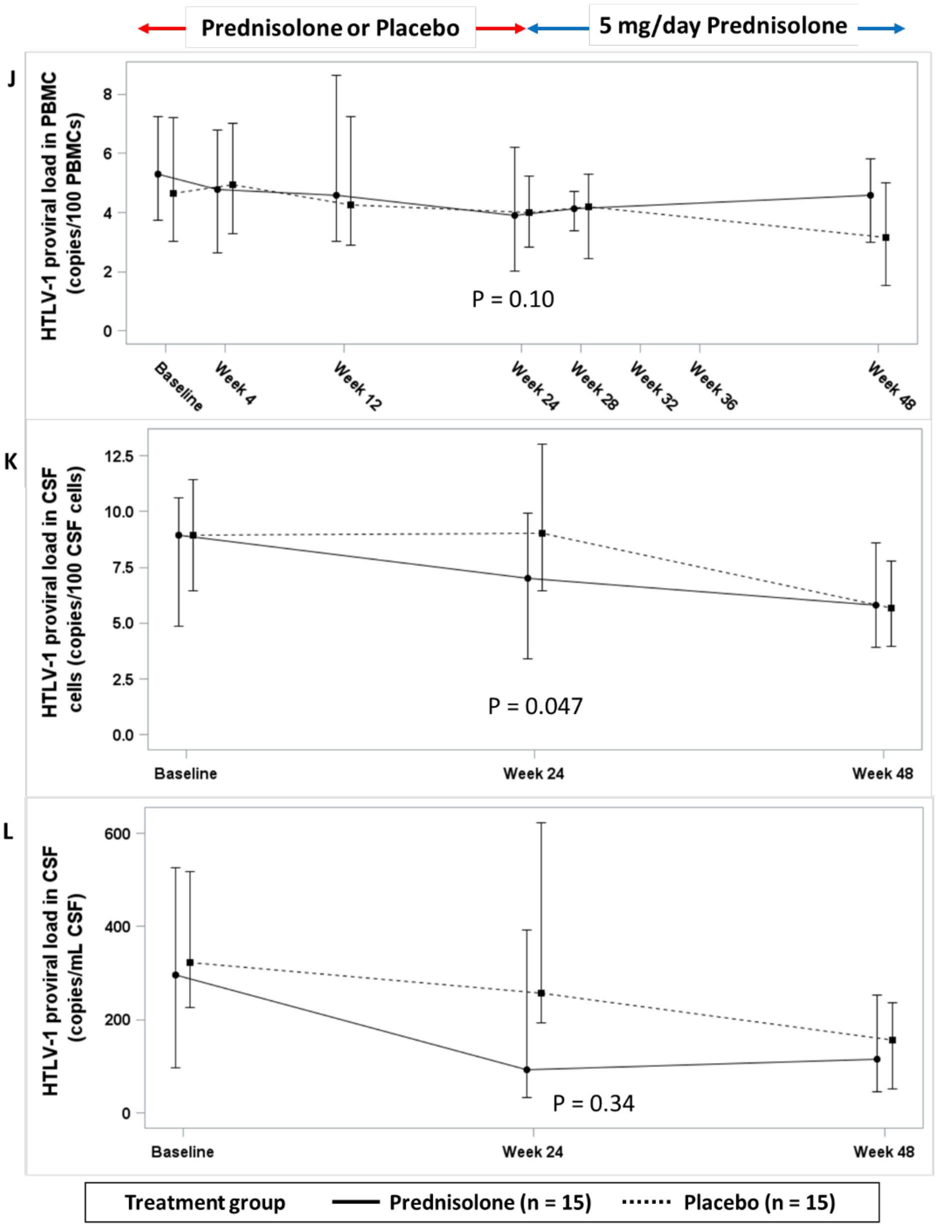

(A-L) Data are presented as median and interquartile range. *p*-values show the significance of between-group differences in changes from baseline at week 24 analyzed using the 2-sample Wilcoxon test.

CSF, cerebrospinal fluid; ICIQ-SF, International Consultation on Incontinence Questionnaire-Short Form; IPEC 1, Insituto de Pesquisa Clinica Evandro Chagas disability score 1; IPSS, International Prostate Symptom Score; N-QOL, Nocturia–Quality of Life Questionnaire; OABSS, Overactive Bladder Symptom Score; PBMCs, peripheral blood mononuclear cells; VAS, visual analog scale.
